# Caught in the Act of Substitution: Interadsorbate Effects on an Atomically Precise Fe/Co/Se Nanocluster

**DOI:** 10.1021/acscentsci.4c00210

**Published:** 2024-05-31

**Authors:** Jonathan A. Kephart, Daniel Y. Zhou, Jason Sandwisch, Nathalia Cajiao, Sebastian M. Krajewski, Paul Malinowski, Jiun-Haw Chu, Michael L. Neidig, Werner Kaminsky, Alexandra Velian

**Affiliations:** † Department of Chemistry, University of Washington, Seattle, Washington 98195, United States; ‡ Department of Chemistry, University of Rochester, Rochester, New York 14627, United States; § Department of Physics, University of Washington, Seattle, Washington 98195, United States; ∥ Inorganic Chemistry Laboratory, Department of Chemistry, University of Oxford, South Parks Road, Oxford OX1 3QR, United Kingdom

## Abstract

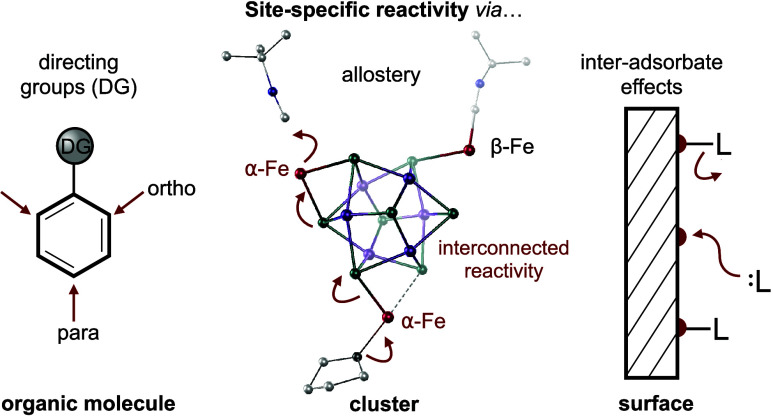

Directing groups guide substitution patterns in organic synthetic schemes, but little is known about pathways to control reactivity patterns, such as regioselectivity, in complex inorganic systems such as bioinorganic cofactors or extended surfaces. Interadsorbate effects are known to encode surface reactivity patterns in inorganic materials, modulating the location and binding strength of ligands. However, owing to limited experimental resolution into complex inorganic structures, there is little opportunity to resolve these effects on the atomic scale. Here, we utilize an atomically precise Fe/Co/Se nanocluster platform, [Fe_3_(L)_2_Co_6_Se_8_L′_6_]^+^ ([**1**(L)_2_]^+^; L = CN^*t*^Bu, THF; L′ = Ph_2_PN^(−)^Tol), in which allosteric interadsorbate effects give rise to pronounced site-differentiation. Using a combination of spectroscopic techniques and single-crystal X-ray diffractometry, we discover that coordination of THF at the ligand-free Fe site in [**1**(CN^*t*^Bu)_2_]^+^ sets off a domino effect wherein allosteric through-cluster interactions promote the regioselective dissociation of CN^*t*^Bu at a neighboring Fe site. Computational analysis reveals that this active site correlation is a result of delocalized Fe···Se···Co···Se covalent interactions that intertwine edge sites on the same cluster face. This study provides an unprecedented atom-scale glimpse into how interfacial metal–support interactions mediate a collective and regiospecific path for substrate exchange across multiple active sites.

## Abstract 2

Akin to directing groups in organic chemistry, allostery directs reaction chemistry across multiple active sites at the surface of an inorganic cluster.

## Introduction

Substrate adsorption is an elementary step in any surface-based chemical transformation^1^ and can dramatically alter electronic properties^2,3^ or invoke surface restructuring within the lattice.^4−9^ But even the simplest coordinative process is complex when considered at an inorganic surface, where an ensemble of interconnected active sites compete for substrate binding.^10^ For example, ligand binding differentiates chemically degenerate surface sites as steric^11^ and inductive^12^ effects give rise to interadsorbate interactions. Although scarcely understood at the atomic level, interadsorbate effects have macroscopic outcomes, modulating catalytic activity,^13−15^ and even promoting long-range substrate ordering.^16,17^ From a molecular point of view, interadsorbate forces guide the reactivity of neighboring surface sites in a manner similar to how directing groups guide substitution patterns in organic synthesis, activating or deactivating neighboring sites at specific locations. Atomistic insights into how interadsorbate effects manifest to facilitate active site speciation and guide surface reactivity are fundamental to the design and implementation of new catalysts.^18^ However, these multi-active site dynamics are difficult to resolve experimentally in heterogeneous systems, requiring advanced surface analysis techniques,^8,16,19−22^ and relying primarily on computational modeling.^23^

Molecular nanoclusters provide suitable models to study the electronic properties and reactivity of solid-state surfaces,^24^ for example those of transition metal oxides^25−27^ and chalcogenides.^28,29^ In these atomically defined systems, ligand binding at a substrate-accessible metal site has been shown to lead to a redistribution of charge within a polymetallic support.^30−33^ Nanoclusters also provide a platform to study the regiochemical aspects of surface substitution reactions when there are multiple equivalent reactive sites available, for example in metal carbonyl clusters^34−37^ or in C_60_ fullerene.^38^ Several instances of site-specific ligand exchange reactivity have also been reported in metal chalcogenide (M_6_Se_8_, M = Co, Re)^39,40^ and metallic Au nanoclusters.^41,42^ Still, nanocluster frameworks designed to capture the interactions between multiple well-defined active sites through a supporting lattice remain scarce, competent examples having only been developed recently by our group.^43,44^

Our group has introduced a family of atomically precise ternary nanoclusters, M_3_Co_6_Se_8_L′_6_ (M = Cr, Fe, Co, Zn; L′ = Ph_2_PN^(−)^Tol; Ph = phenyl, Tol = *p*-tolyl), that model the interplay of multiple active sites dispersed on an inorganic support.^43−47^ These clusters feature three low-coordinate edge metals that interact directly with a Co_6_Se_8_ support via hemilabile M–Se bonds. This edge/support M/Co_6_Se_8_ construct is reminiscent of Fe-doped CoSe_2_ and other widely studied mixed transition metal chalcogenides, such as edge-doped MoS_2_.^22,48−53^ With its well-defined metal–support architecture, this M/Co/Se cluster system has furnished key insights into the electronic and coordinative aspects of the metal–support interaction, and enabled the direct observation of multi-active site interactions.^45,54^ Notably, we have shown the reactivity of neighboring active sites is intertwined via through-cluster allosteric effects that regulate catalytic activity or guide self-assembly.^43,44,46^

**Figure 1:**
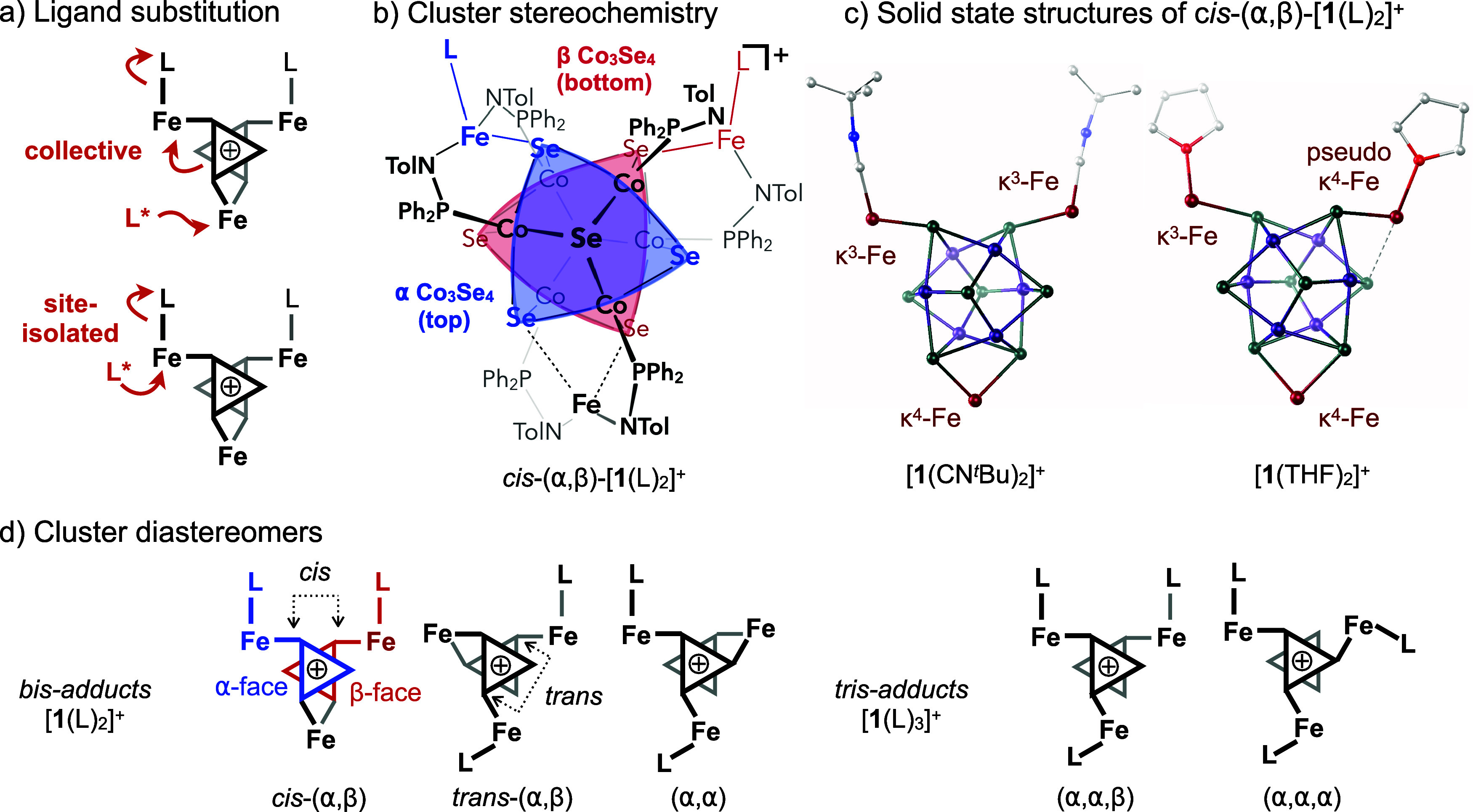
(a) Multi- vs single-site substitution mechanisms at site-differentiated bis-adducts, *cis*-(α,β)-[**1**(L)_2_]^+^. (b) Connectivity and stereochemistry of *cis*-(α,β)-[**1**(L)_2_]^+^. (c) Bis-adducts [**1**(CN^*t*^Bu)_2_][PF_6_] and [**1**(THF)_2_][PF_6_] crystallize exclusively as *cis*-(α,β) stereoisomers. Solid-state structures acquired from single-crystal X-ray diffraction data are depicted as ball and stick models. Amidophosphine ligands, disorder, and solvent molecules are omitted for clarity. (d) All possible diastereomers for bis- and tris-adducts [**1**(L)_2_]^+^ and [**1**(L)_3_]^+^, respectively.

Here, we uncover how allostery facilitates a collective pathway for ligand substitution on the surface of a family of triiron clusters, [Fe_3_(L)_2_Co_6_Se_8_L′_6_][PF_6_] ([**1**(L)_2_][PF_6_], L = THF, CN^*t*^Bu; Figure 1a,b).^45^ In this platform, ligand binding at one Fe edge site induces ligand dissociation at a distal Fe edge (Figure 1a). Experimental and computational analyses trace the allostery and observed regioselectivity of the active sites to covalent Fe···Se···Co···Se interactions that specifically intertwine the reactivity of two Fe centers located on the same cluster face.

## Results and Discussion

### Site-Differentiation and Stereochemistry of [**1**(L)_2_]^+^ Clusters

The triiron clusters [**1**(L)_2_][PF_6_] are uniquely suited for this investigation because of their prominent allostery in the +1-oxidation manifold. The three Fe edge sites in [**1**(L)_2_]^+^ have significantly differentiated affinities for exogenous ligands, favoring isolation as bis-ligated adducts. This is best visualized in their solid-state structures, which feature two types of edges: a κ^4^-Fe site, bound only by the Co/Se cluster-ligand, and two κ^3^-Fe(L) sites, bound also to an exogenous ligand (Figure 1c). In contrast to the neutral triiron clusters which can be isolated completely ligand-free, mono-oxidation creates a regime wherein two monodentate exogenous ligands are necessary and sufficient for isolation. For example, [**1**(THF)_2_][PF_6_] and **1**(MeCN)(OTf) exhibit one edge free of surface ligands (κ^4^-Fe) despite crystallization in the presence of a large excess of THF or MeCN, respectively (>800 equiv; Figure 1c and Figure S26). Similarly, the neutral tris(isocyanide) cluster **1**(CN^*t*^Bu)_3_ was found to liberate isocyanide from one of the Fe edges upon chemical oxidation to afford the site-differentiated product [**1**(CN^*t*^Bu)_2_][PF_6_].^45^ Despite the preference for isolation as a site-differentiated bis-adduct, the κ^4^-Fe site in [**1**(CN^*t*^Bu)_2_][PF_6_] exhibits a measurable, albeit low, CN^*t*^Bu binding affinity (*K*_a_ ≃ 24 M^–1^)^45^ and can in fact be crystallized as a tris(isocyanide) adduct, [**1**(CN^*t*^Bu)_3_][PF_6_], in the presence of at least 5 additional equivalents of CN^*t*^Bu (Figure S43). This preference for site-differentiation with weak ligation at a single Fe center distinguishes the [**1**(L)_2_]^+^ clusters as an ideal platform to study the allosteric ligand exchange reactivity.

To uncover the rules of collective ligand substitution on these multi-active site clusters, it is important to understand their stereochemistry. Like the stereoisomers of di- and trisubstituted cyclopropanes, different diastereomers can form upon ligand binding at each cluster edge. Coordination of a monodentate ligand at a κ^4^-Fe desymmetrizes the cluster such that the Fe–Se bonds become α or β stereogenic, where α and β denote the equatorial Se atoms located on the top (α) or bottom (β) Co_3_Se_4_ hemispheres of the Co_6_Se_8_ cube (Figure 1b). Therefore, three unique diastereomers are possible for bis-adduct [**1**(L)_2_]^+^, (α,α), *cis*-(α,β), and *trans*-(α,β), while two are possible for tris-adducts [**1**(L)_3_]^+^, (α,α,α) and (α,α,β) (Figure 1d). These clusters are isolated exclusively as the *C*_2_-symmetric *cis*-(α,β) and *C*_1_-symmetric (α,α,β) diastereomers, respectively, when the M–Se interactions are of appreciable strength.^43−46^ Interestingly, the mono-oxidized [**1**(L)_2_]^+^ clusters all adopt a *cis*-(α,β) conformation in the solid-state (Figure 1c). We propose that the *cis*-(α,β) conformation is electronically favored because it maximizes the separation of the κ^3^-Fe(L)–Se bonds away from those of the tightly bound κ^4^-Fe site. This empirical observation is supported by density functional theory (DFT) calculations, which indicate a slight thermodynamic preference (∼1.2–1.3 kcal/mol) for *cis*-(α,β)-[**1**(CNMe)_2_]^+^ compared to the (α,α) or *trans*-(α,β) congeners (Section S7).

### Caught in the Act: Single Crystal X-ray Diffraction Studies Resolve Ligand Substitution

Ligand exchange on the cluster surface occurs rapidly in solution for L-type ligands (Section S2.4). Whereas isocyanide rapidly displaces THF when [**1**(THF)_2_][PF_6_] is treated with CN^*t*^Bu (Figure S16), the reverse process (i.e., the solvolysis of [**1**(CN^*t*^Bu)_2_][PF_6_] in THF) is thermodynamically unfavorable (Figure S56), and is therefore positioned to provide a fertile space for the resolution of discrete intermediates en route to the bis(THF) adduct.

**Figure 2:**
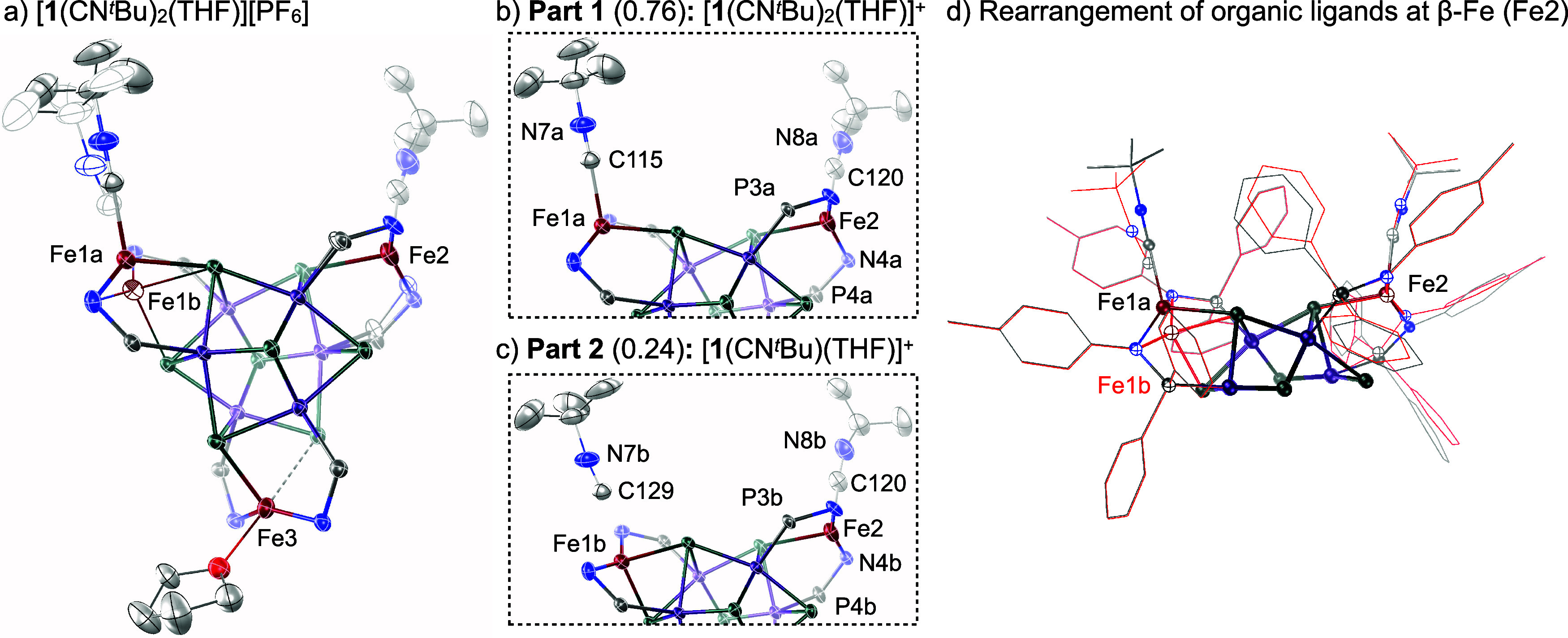
(a) Representative solid-state structure of the cocrystallized intermediates (α,α,β)-[**1**(CN^*t*^Bu)_2_(THF)][PF_6_] and *trans*-(α,β)-[**1**(CN^*t*^Bu)(THF)][PF_6_]. Thermal ellipsoids are plotted at 50% probability. Insets included to highlight the (b) major and (c) minor components of the disordered crystal. (d) Overlay of the organic ligands at the α-Fe1 and β-Fe2 edge sites in the major (gray) and minor (red) components of the crystal structure detailed in (a)–(c).

Indeed, the substitution of isocyanide with a THF molecule is caught in the act when single crystals of [**1**(CN^*t*^Bu)_2_][PF_6_] are grown in the presence of excess THF. Single-crystal X-ray diffraction analysis of the isolated crystals reveals the presence of two species. The major component is the tris-adduct (α,α,β)-[**1**(CN^*t*^Bu)_2_(THF)][PF_6_], which features a THF molecule bound at the previously ligand-free κ^4^-Fe site (Figure 2b). The minor component is the bis-adduct *trans*-(α,β)-[**1**(CN^*t*^Bu)(THF)][PF_6_] captured with a dissociated molecule of isocyanide near a newly formed κ^4^-Fe site, previously the α-Fe(CN^*t*^Bu) edge (Figure 2c). The bis-adduct is ostensibly formed upon dissociation of isocyanide from the α-Fe(CN^*t*^Bu) site of the parent tris-adduct, (α,α,β)-[**1**(CN^*t*^Bu)_2_(THF)][PF_6_]. While THF binding does not perturb the Fe–Se bonding interactions at the β-Fe, the ancillary organic ligands undergo a notable reorganization at this edge, as illustrated in Figure 2d. Together, the bis- and tris-adducts illustrate the first and second steps of a collective ligand exchange process and capture how ligand binding at one site promotes dissociation at a neighboring one (Figure 4).

Three independent crystallizations have given rise reproducibly to disorder at the α-Fe(CN^*t*^Bu) site, in ratios of 76:24, 85:15, and 95:5, for major and minor components, respectively. We hypothesize that the variation in the relative populations of the two components across the three samples is due to differences in ambient lighting or crystal size. Whereas in the first two crystals the disorder is identical, with the isocyanide dissociating from the α-Fe site as depicted in Figure 2c, in the latter, the data were modeled better if the entire Fe(CN^*t*^Bu) unit migrates to accommodate the formation of a second Fe–Se bond, giving rise to a pseudo-κ^4^-Fe(CN^*t*^Bu) edge site (Figure S34). To probe the impact of temperature on the isocyanide dissociation, X-ray diffraction data for the single crystal featuring the 95:5 disorder were collected at 100, 150, 200, and 250 K, then once more at 100 K. Upon warming over this range, the structure is gradually enriched in the minor component at the Fe(CN^*t*^Bu) site from 5% at 100 K to 16% at 250 K, reverting to 5% once more upon cooling down to 100 K (Section S6.5). This demonstrates that the dynamics interconnecting the two α-Fe sites are reversible *in crystallo* and suggests this process is thermally activated.

### Solution-Phase Ligand Exchange

**Figure 3:**
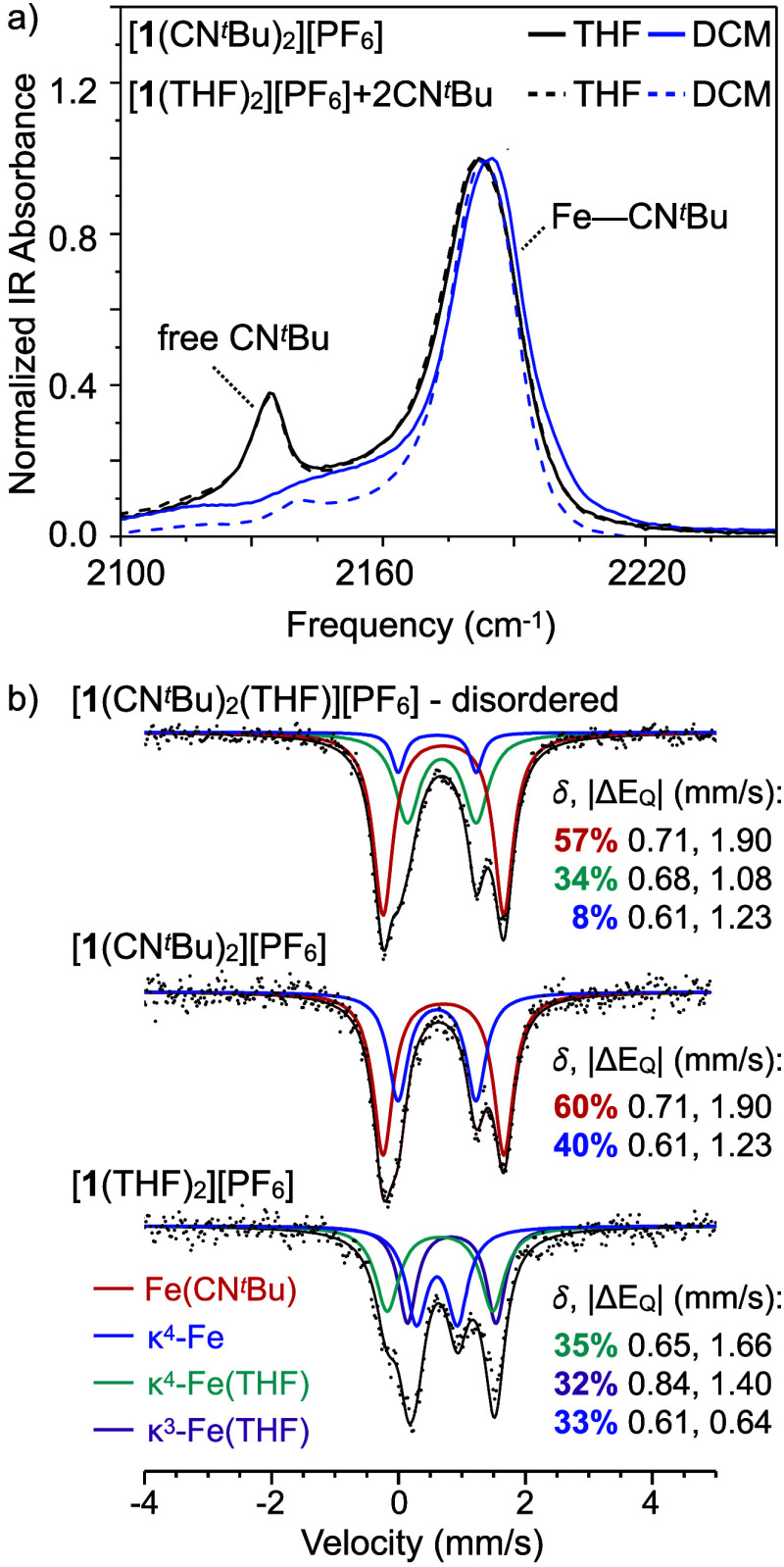
(a) Infrared spectra of equilibrium state reached upon dissolving [**1**(CN^*t*^Bu)_2_][PF_6_] or [**1**(THF)_2_][PF_6_] + 2 equiv of CN^*t*^Bu in neat THF or DCM. (b) Zero-field ^57^Fe Mössbauer spectra (80 K) collected on bulk solid samples of disordered [**1**(CN^*t*^Bu)_2_(THF)][PF_6_], [**1**(CN^*t*^Bu)_2_][PF_6_], and [**1**(THF)_2_][PF_6_], with quadrupole doublets assigned to distinct Fe environments.

Solution-phase infrared spectroscopy reveals that a ligand substitution equilibrium favoring isocyanide coordination is rapidly established when [**1**(CN^*t*^Bu)_2_][PF_6_] is dissolved in neat THF (Figure 3a). Two CN vibrational frequencies corresponding to bound (2184 cm^–1^) and free (2135 cm^–1^) CN^*t*^Bu are observed in a 7:1 ratio. The same equilibrium ratio is reached upon titrating [**1**(THF)_2_][PF_6_] with CN^*t*^Bu (0.1–2.0 equiv; Section S3) in neat THF, but no free CN^*t*^Bu is detected when [**1**(CN^*t*^Bu)_2_][PF_6_] is dissolved in a noncoordinating solvent (DCM), indicating that dissociation of isocyanide occurs only when another ligand is present to coordinate to the cluster. When [**1**(CN^*t*^Bu)_2_][PF_6_] is dissolved in neat THF, the presence of free isocyanide suggests the formation of THF adducts in the equilibrium mixture of the solvolysis, such as [**1**(CN^*t*^Bu)(THF)]^+^, [**1**(CN^*t*^Bu)(THF)_2_]^+^, and [**1**(THF)_2_]^+^. However, the IR or NMR spectroscopy measurements do not distinguish between different types of bound Fe–L species (Section S2.4), and therefore cannot resolve the single-site exchange of CN^*t*^Bu and THF from the multisite mechanism that is implied crystallographically.

### ^57^Fe Mössbauer Spectroscopy

The dissociation of isocyanide from tris-adduct [**1**(CN^*t*^Bu)_2_(THF)][PF_6_] is a bulk phenomenon. The 80 K Mössbauer spectrum for a solid sample of disordered [**1**(CN^*t*^Bu)_2_(THF)][PF_6_] (Figure 3b) reveals three quadrupole doublets representative of high-spin Fe(II) environments. These are attributed to κ^3^-Fe(CN^*t*^Bu), κ^4^-Fe, and pseudo-κ^4^-Fe(THF) edges based on their similarity to their counterparts in [**1**(CN^*t*^Bu)_2_][PF_6_] and [**1**(THF)_2_][PF_6_]. The area ratio of the spectrum matches the expected distribution of Fe edges based on the maximum chemical disorder observed crystallographically ([**1**(CN^*t*^Bu)_2_(THF)][PF_6_]:[**1**(CN^*t*^Bu)(THF)][PF_6_] in a 76:24 ratio). The signal corresponding to an 8% area ratio can be identified as the contribution from the newly formed κ^4^-Fe edge of the minor component, [**1**(CN^*t*^Bu)(THF)]^+^. This area ratio is also consistent with the amount of free isocyanide detected by IR spectroscopy measurements in the solution-phase ligand exchange study. For the bis-adduct [**1**(THF)_2_][PF_6_], the 80 K Mössbauer spectrum of a solid sample features three unique high-spin Fe(II) environments in a 1:1:1 area ratio, reflecting the κ^4^-Fe, pseudo-κ^4^-Fe(THF), and κ^3^-Fe(THF) coordination of the edge sites observed crystallographically (Figure S25). The differences in quadrupole splitting values observed for the Fe(THF) edge sites are attributed to the variation in the Fe···Se bonding interactions at these sites.

### Rules of Substitution: Electronic Factors Guiding Regioselective Ligand Substitution

**Figure 4:**
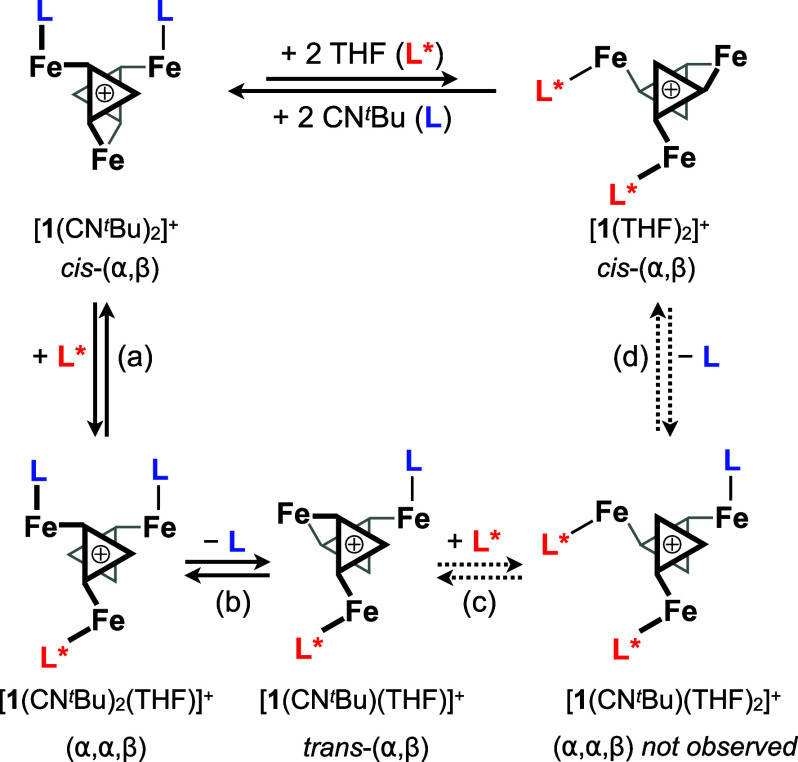
Proposed mechanism for the interconversion of [**1**(CN^t^Bu)_2_]^+^ and [**1**(THF)_2_]^+^ via a multisite ligand substitution pathway.

A multisite, regioselective substitution mechanism that interconverts *cis*-(α,β)-[**1**(CN^*t*^Bu)_2_]^+^ and *cis*-(α,β)-[**1**(THF)_2_]^+^, based on experimental observations, is illustrated in Figure 4. In the proposed mechanism, (a) THF binding at *cis*-(α,β)-[**1**(CN^*t*^Bu)_2_]^+^ is followed by (b) dissociation of the isocyanide oriented α with respect to the THF ligand. (c) Coordination of a second THF molecule to the κ^4^-Fe site of *trans*-(α,β)-[**1**(CN^*t*^Bu)(THF)]^+^ leads to the experimentally elusive tris-adduct (α,α,β)-[**1**(CN^*t*^Bu)(THF)_2_]^+^. Finally, (d) the tris-adduct undergoes isocyanide dissociation to generate *cis*-(α,β)-[**1**(THF)_2_]^+^ following the same α/α regioselective substitution pattern.

**Figure 5:**
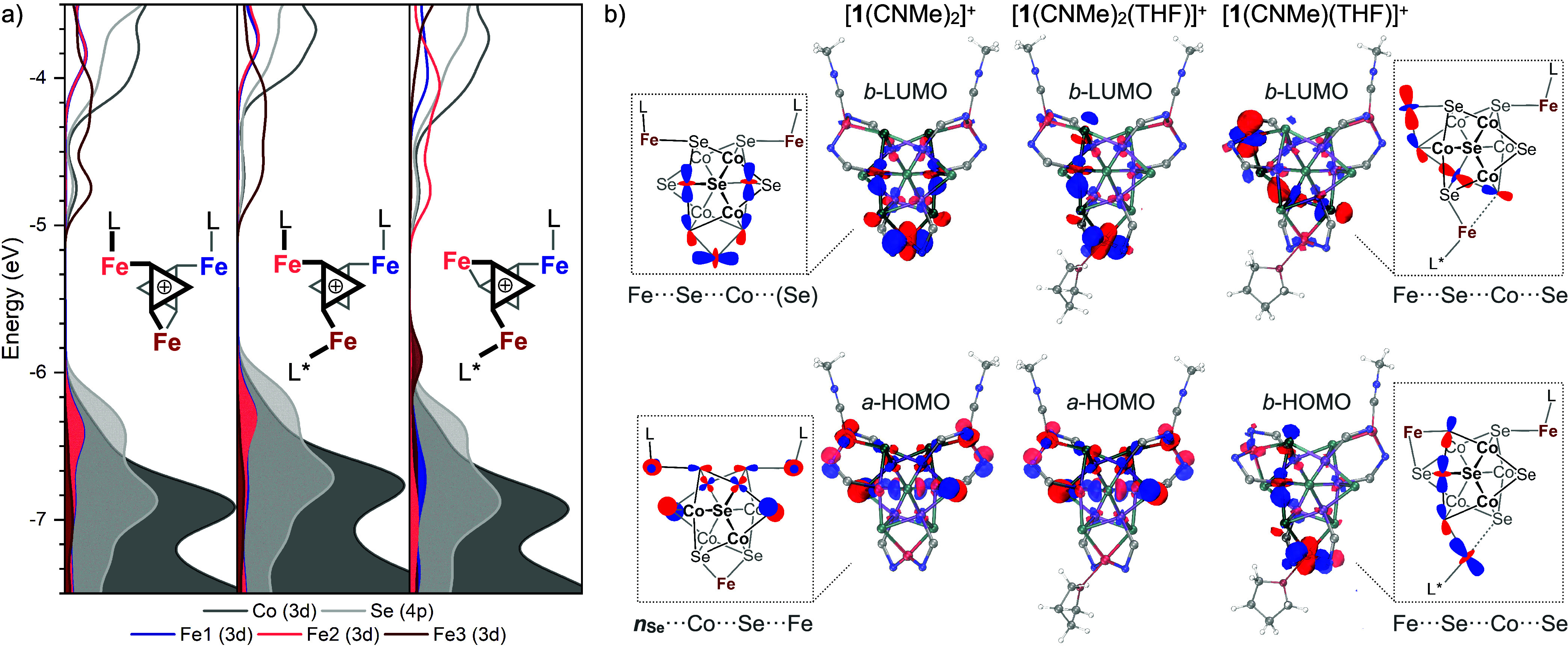
(a) Partial density of states for *cis*-(α,β)-[**1**(CNMe)_2_]^+^, (α,α,β)-[**1**(CNMe)_2_(THF)]^+^, and *trans*-(α,β)-[**1**(CNMe)(THF)]^+^, illustrating the individual contributions of each Fe edge to the frontier states and (b) isosurface plots (0.04 au) and schematic representations for select frontier orbitals. Calculations were run at the uB3LYP+//cc-PVTZ level of theory. Up and down spin manifolds are denoted as *a* or *b*, respectively.

To elucidate the electronic origins of the observed α/α regioselectivity, we turned to computational modeling. DFT calculations qualitatively confirm the experimental observations, such as the preference for isocyanide coordination over that of THF and the endergonic nature of the overall equilibrium (Figure S56). Spin-multiplicity assignments are informed by solid-state magnetic susceptibility measurements for [**1**(CN^*t*^Bu)_2_][PF_6_] (Section S4), and the CN^*t*^Bu ligand was approximated by CNMe *in silico*. Partial density of states (*p*DOS) analysis of the key intermediates *cis*-(α,β)-[**1**(CNMe)_2_]^+^, (α,α,β)-[**1**(CNMe)_2_(THF)]^+^, and *trans*-(α,β)-[**1**(CNMe)(THF)]^+^ reveals that multisite ligand substitution promotes a reshuffling of Fe-centered frontier electronic states (Figure 5a). The corresponding singly occupied molecular orbitals, whose topology sheds light on the correlated reactivity of neighboring sites, are depicted in Figure 5b. Associated molecular orbital energy diagrams and additional computational details are provided in Section S7.

In *cis*-(α,β)-[**1**(CNMe)_2_]^+^, two nearly degenerate, low-lying unoccupied orbitals (LUMOs) within the *b*-spin manifold extend symmetrically from the κ^4^-Fe edge into the Co_6_Se_8_ support along the α and β face. These -Fe···Co/Se antibonding orbitals span the Fe···Se···Co···Se vectors within the cluster revealing a high degree of covalency between the Fe edge and the Co/Se support, as is anticipated for metal-chalcogenide clusters (Figure S58).^28,55,56^ When THF coordinates to *cis-*(α,β)-[**1**(CNMe)_2_]^+^ to form the tris-adduct (α,α,β)-[**1**(CNMe)_2_(THF)]^+^, the near-degeneracy of the LUMO and LUMO+1 orbitals is disrupted. This leads to Fe···Se···Co···Se orbitals with primary contributions from Co/Se atoms on either the α or β cluster halves, respectively (Figure S59). This desymmetrization demonstrates how ligand binding at the κ^4^-Fe edge polarizes bonding within the Co/Se support to interconnect edge sites on the same cluster face. Meanwhile, the highest occupied molecular orbital (HOMO) maintains nonbonding Se 4p character at the two Fe(CNMe) sites, ostensibly ready to engage in Fe···Se bond formation with expulsion of isocyanide. Isocyanide dissociation from tris-adduct (α,α,β)-[**1**(CNMe)_2_(THF)]^+^ to form *trans-*(α,β)-[**1**(CNMe)(THF)]^+^ occurs with population of the antibonding Fe···Se···Co···Se orbital. Figure 5b illustrates the nearly identical topologies of the HOMO of *trans-*(α,β)-[**1**(CNMe)(THF)]^+^ and the LUMO of (α,α,β)-[**1**(CNMe)_2_(THF)]^+^, signifying that isocyanide displacement proceeds with an increase in the Co/Se electron density near the α-Fe(CNMe) edge. Meanwhile, the LUMO of [**1**(CNMe)(THF)]^+^ is primarily localized at the new κ^4^-Fe edge with antibonding Fe···Se···Co···Se character directed back toward the weak-field Fe(THF) edge site. Independent crystallographic measurements of [**1**(CN^*t*^Bu)(THF)]^+^ reveal a contraction of the newly formed Fe–Se bond, consistent with depopulation of this Fe···Se antibonding interaction. The topology of the calculated Fe···Se···Co···Se orbital suggests that the κ^4^-Fe edge will favor α-ligand binding and promote dissociation of the more labile THF ligand to regenerate *cis-*(α,β)-[**1**(CNMe)_2_]^+^.

Overall, the relative population/depopulation of the frontier Fe···Se···Co···Se orbitals dictates the strength of edge–support bonding (i.e., Fe–Se bonds) and, in turn, the edge site’s affinity for exogenous ligands. Indeed, mono-oxidation of the putative neutral *cis*-(α,β)-[**1**(CNMe)_2_] cluster increases Fe–Se bonding character at the κ^4^-Fe site leading to pronounced site-differentiation of the cluster, as indicated by DFT analysis (Figure S46). As THF associates at the κ^4^-Fe site of *cis*-(α,β)-[**1**(CNMe)_2_]^+^, the directionality of the responsive Fe···Se···Co···Se orbital encourages the migration of the strong edge–support interaction to the α-Fe(CN^*t*^Bu) edge site, driving dissociation of CN^*t*^Bu.

## Conclusions

At heterogeneous interfaces, chemically equivalent surface metals are distinguished as they compete for substrate binding and activation, with their reactivities intertwined through a supporting lattice. Here, we discover that interfacial metal–support interactions are responsive to substrate binding and promote regiospecific through-cluster ligand substitution dynamics at the active sites of an Fe/Co/Se nanocluster. This study provides an atom-scale glimpse into how interadsorbate forces may guide the reactivity of heterogeneous surfaces. This combined experimental and computational investigation into the nanocluster allostery enables us to uncover the electronic factors that guide the α/α-regioselectivity in a multisite ligand substitution sequence. Ultimately, this work introduces a molecular formalism to investigate the interadsorbate effect and its consequences for reactivity. Extrapolating to heterogeneous systems, we expect that the impact of collective substrate activation pathways on reactivity to be dependent on the strength of interadsorbate effects, which vary with chemical composition and bonding within the active site/support construct.

## Supporting Information Available

Synthetic protocols and experimental characterization for all compounds, including solution-phase IR spectra, solid-state magnetometry, crystallographic data, and computational details (PDF)
Crystallographic data for [**1**(CN^*t*^Bu)_2_(THF)][PF_6_], [**1**(THF)_2_][PF_6_], [**1**(CN^*t*^Bu)_3_][PF_6_], and **1**(MeCN)(OTf) (TXT)
CIF files (ZIP)
