# Supplementary material for: Caught in the Act of Substitution: Interadsorbate Effects on an Atomically Precise Fe/Co/Se Nanocluster
Source: ACS Cent Sci. 2024 May 31;10(6):1276–82. doi: 10.1021/acscentsci.4c00210 (PMC11212139; doi:10.1021/acscentsci.4c00210)
Supplement: Supplementary file 1 — oc4c00210_si_001.pdf [file oc4c00210_si_001.pdf]

Supporting information for:

Caught in the Act of Substitution: Inter-adsorbate Effects on an Atomically Precise Fe/Co/Se Nanocluster

Jonathan A. Kephart[1], Daniel Y. Zhou[1], Jason Sandwisch[1], Sebastian Krajewski[1], Nathalia Cajiao[2], Paul Malinowski[3], Jiun-Haw Chu[3], Michael L. Neidig[4], Werner Kaminsky,[1] Alexandra Velian[1]*

[1]Department of Chemistry, University of Washington, Seattle, Washington 98195, United States; [2]Department of Chemistry, University of Rochester, Rochester, New York 14627, United States; [3]Department of Physics, University of Washington, Seattle, Washington 98195, United States; [4]Inorganic Chemistry Laboratory, Department of Chemistry, University of Oxford, South Parks Road, Oxford OX1 3QR, United Kingdom. *Email: avelian@uw.edu.

Table of Contents

S1 General Information 2
S2 Synthetic Details and Characterization of Products 3
S2.1 Adapted Synthesis of $[Fe_3(CN^tBu)_2Co_6Se_8L_6][PF_6]$ ($[1(CN^tBu)_2][PF_6]$) 3
S2.2 Synthesis and Isolation of $[Fe_3(THF)_2Co_6Se_8L_6][PF_6]$ ($[1(THF)_2][PF_6]$) 6
S2.3 Synthesis and Isolation of $[Fe_3(MeCN)Co_6Se_8L_6][OTf]$ (1(MeCN)(OTf)) 8
S2.4 NMR Titration of $[1(CN^tBu)_2][PF_6]$ with THF 11
S3 Fourier Transform Infrared Spectroscopy 12
S4 Magnetic Characterization of $[1(CN^tBu)_2][PF_6]$ 17
S5 ^{57}Fe Mössbauer Spectroscopy 20
S6 X-ray Diffraction Studies 20
S6.1 $[Fe_3(THF)_2Co_6Se_8L_6][PF_6]$ ($[1(THF)_2][PF_6]$) 20
S6.2 $Fe_3(MeCN)(OTf)Co_6Se_8L_6$ (1(MeCN)(OTf)) 21
S6.3 $[Fe_3(CN^tBu)_2(THF)Co_6Se_8L_6][PF_6]$ (Disordered $[1(CN^tBu)_2(THF)][PF_6]$) – sample 1 22
S6.4 $[Fe_3(CN^tBu)_2(THF)Co_6Se_8L_6][PF_6]$ (Disordered $[1(CN^tBu)_2(THF)][PF_6]$) – sample 2 24
S6.5 $[Fe_3(CN^tBu)_2(THF)Co_6Se_8L_6][PF_6]$ (Disordered $[1(CN^tBu)_2(THF)][PF_6]$) – sample 3 26
S6.6 $[Fe_3(CN^tBu)_3Co_6Se_8L_6][PF_6]$ ($[1(CN^tBu)_3][PF_6]$) 31
S6.7 X-ray Tables 33
S7 DFT Calculations 40
S7.1 DFT Input Files 65
S8 References 109

S1 General Information

All syntheses were conducted under a dinitrogen atmosphere using a standard Schlenk line or an LC Technology Solutions glovebox equipped with a freezer set to −35 °C. All glassware was dried at 160 °C for a minimum of 12 h prior to use.

Solvents were purchased from Fischer Scientific and degassed, dried and purified using solvent purification columns housed in a stainless-steel cabinet and dispensed by a stainless-steel Schlenk line manufactured by JC Meyer Solvent Systems. Tetrahydrofuran (THF), diethyl ether (Et_2O), acetonitrile (MeCN), and methylene chloride (DCM) are passed through two packed columns of neutral alumina. *n*-Pentane and toluene are passed through a column packed with alumina, and one containing Q5 reactant, a copper(II) oxide oxygen scavenger. All solvents were passed through an in-line, 2 µm filter, then stored over activated 3 Å molecular sieves within the glovebox. 3 Å molecular sieves were purchased from Sigma Aldrich and activated under vacuum at 300 °C for 48 h.

Deuterated solvents (C_6D_6, CD_2Cl_2, CD_3CN) were purchased from Cambridge Isotope Laboratories, Inc., degassed and dried over activated 3Å molecular sieves in the glovebox for a minimum of 72 h. $FeCl_2$ (anhydrous, 98+%) was purchased from Strem Chemicals, Inc. and dried under vacuum at 120 °C for 24 h prior to use. $Co_2(CO)_8$ (stabilized with 2-5% hexanes; Strem Chemicals, Inc.) was stored in the glovebox freezer and used as received. The aminophosphine $Ph_2PN(H)Tol$ (L^H, Ph = phenyl, Tol = 4-tolyl), $Co_6Se_8L^H{}_6$, $Li_6(py)_6Co_6Se_8L_6$, and $Fe_3Co_6Se_8L_6$ were prepared according to methods described in the literature.[1,2] Chlorodiphenylphosphine (97%) and *p*-toluidine (99+%) were purchased from Alfa Aesar and used without further purification. Selenium (99.5%, powder, 200 mesh) and *n*-butyl lithium (2.5 M in hexanes) were purchased from Sigma Aldrich and used without further purification. *Tert*-butyl isocyanide (CN^tBu, 98%) was purchased from Sigma Aldrich and stored over activated 3Å molecular sieves in the glovebox for 72 h prior to use. Ferrocenium hexafluorophosphate ($FcPF_6$, 97%) was purchased from Sigma Aldrich and recrystallized from dry acetone and diethyl ether prior to use. Silver triflate (≥99%) was purchased from Sigma Aldrich and used without further purification.

Solution-phase FTIR spectra were recorded at 298 K on a Perkin Elmer Frontier FTIR spectrometer with a spectral resolution of 0.5 cm^{-1} equipped with a solution-cell holder. UV-vis-*n*IR absorption spectra were acquired using a Varian Cary 5000 UV-vis-*n*IR spectrophotometer in *n*IR quartz cuvettes (Spectrocell Inc., 10 mm path length, 220–3500 nm spectral window). Extinction coefficients were collected in triplicate for each compound and reported as an average. NMR spectra were acquired at 25 °C on Bruker 300 or 500 MHz spectrometers, as indicated. ^1H spectra were referenced to residual deuterated solvent peaks. ^{19}F NMR spectra were referenced externally to CCl_3F.

Samples were prepared for elemental analysis by crushing single-crystalline material into a powder and then removing volatiles under reduced pressure for 12 h. Co, Se, P elemental analysis was conducted using a Perkin-Elmer Nexion 2000B inductively-coupled plasma mass spectrometer (ICP-MS). Prior to ICP-MS analysis, samples were digested in neat nitric acid (Fisher Scientific, ICP-MS grade) at 50 °C, and serial dilutions were performed with Milli-Q reference water (18.2 MΩ).

Safety Statement: No unexpected or unusually high safety hazards were encountered.

S2 Synthetic Details and Characterization of Products

S2.1 Adapted Synthesis of $[Fe_3(CN^tBu)_2Co_6Se_8L_6][PF_6]$ ($[\mathbf{1}(CN^tBu)_2][PF_6]$)

A 50 mL Schlenk flask equipped with a Teflon valve and magnetic stir bar was loaded with **1** (1.000 g, 0.345 mmol, 1 equiv) and DCM (25 mL). The dark red-brown solution was stirred at room temperature until the mixture was homogeneous. The solution was then spiked with neat CN^tBu (78 μL, 0.69 mmol, 2 equiv) and cooled to –35 °C before solid ferrocenium hexafluorophosphate (0.115 g, 0.345 mmol, 1 equiv) was added portion wise with rapid stirring. This mixture was warmed to room temperature and stirred for 1 h. Over the course of this time the solution adopted a dark red-purple color. The solution was passed through a plug of Celite on a fritted glass funnel and the filtrate was layered with *n*-pentane (~120 mL) for bulk crystallization. The layered mixture was kept still at room temperature for 16 hours, affording a crop of large, dark red prismatic crystals, which were collected on a fritted glass funnel and washed with additional pentane (3 x 6 mL). This material was recrystallized once more to remove trace paramagnetic impurities. Volatiles were removed from these solids *in vacuo* to afford $[\mathbf{1}(CN^tBu)_2][PF_6]$ as an analytically pure, crystalline solid (0.952 g, 0.297 mmol, 86%).

UV-vis-*n*IR (DCM, 25 °C) λ: 437 (ε = 38,290 M^{-1} cm^{-1}). ^1H NMR (CD_3CN, 300 MHz) δ: 49.82 (s, 18H, $\nu_{1/2}$ = 98 Hz, tolyl-C$\boldsymbol{H}_3$); 33.22 (s, $\nu_{1/2}$ = 56 Hz); 21.52 (s, $\nu_{1/2}$ = 226 Hz); 16.90 (s, $\nu_{1/2}$ = 185 Hz); 14.52 (s, $\nu_{1/2}$ = 33 Hz); 10.89 (s, $\nu_{1/2}$ = 28 Hz); 3.42 (s, $\nu_{1/2}$ = 33 Hz); 0.54 (s, 18H, $\nu_{1/2}$ = 26 Hz, CN^tBu-C$\boldsymbol{H}_3$); −35.22 (s, $\nu_{1/2}$ = 550 Hz) ppm. ^1H NMR (CD_2Cl_2, 300 MHz) δ: 57.70 (s, $\nu_{1/2}$ = ~6000 Hz); 36.46 (s, $\nu_{1/2}$ = 2500 Hz); 21.28 (s, $\nu_{1/2}$ = 2430 Hz), 15.52 (s, $\nu_{1/2}$ = 660 Hz); 14.64 (s, $\nu_{1/2}$ = 65 Hz); 11.23 (s, 6H, $\nu_{1/2}$ = 112 Hz); 10.86 (s, $\nu_{1/2}$ = 99 Hz); 3.24 (s, $\nu_{1/2}$ = 85 Hz); −0.58 (s, $\nu_{1/2}$ = 41 Hz); −37.85 (s, $\nu_{1/2}$ = ~8000 Hz) ppm. ^{31}P NMR (CD_2Cl_2, 202 MHz) δ: −144.57 (*h*, $^1J_{PF}$ = 722 Hz) ppm. Note: no other ^{31}P resonances observed within the range of ±2000 ppm. ^{19}F NMR (CD_2Cl_2, 471 MHz) δ: −72.96 (*d*, $^1J_{PF}$ = 718 Hz) ppm. ^{13}C NMR (CD_2Cl_2, 125 MHz): no peaks observed between ±400 ppm. μ_{eff} (Evans Method, CD_2Cl_2, 298K): 9.4(3) μ_B. Elemental analysis found (calc.) for $[\mathbf{1}(CN^tBu)_2][PF_6]$ (Formula: $C_{124}H_{120}Co_6F_6Fe_3N_8P_7Se_8$): Co 11.06 (11.03), Se 19.89 (19.71), P 6.90 (6.76).

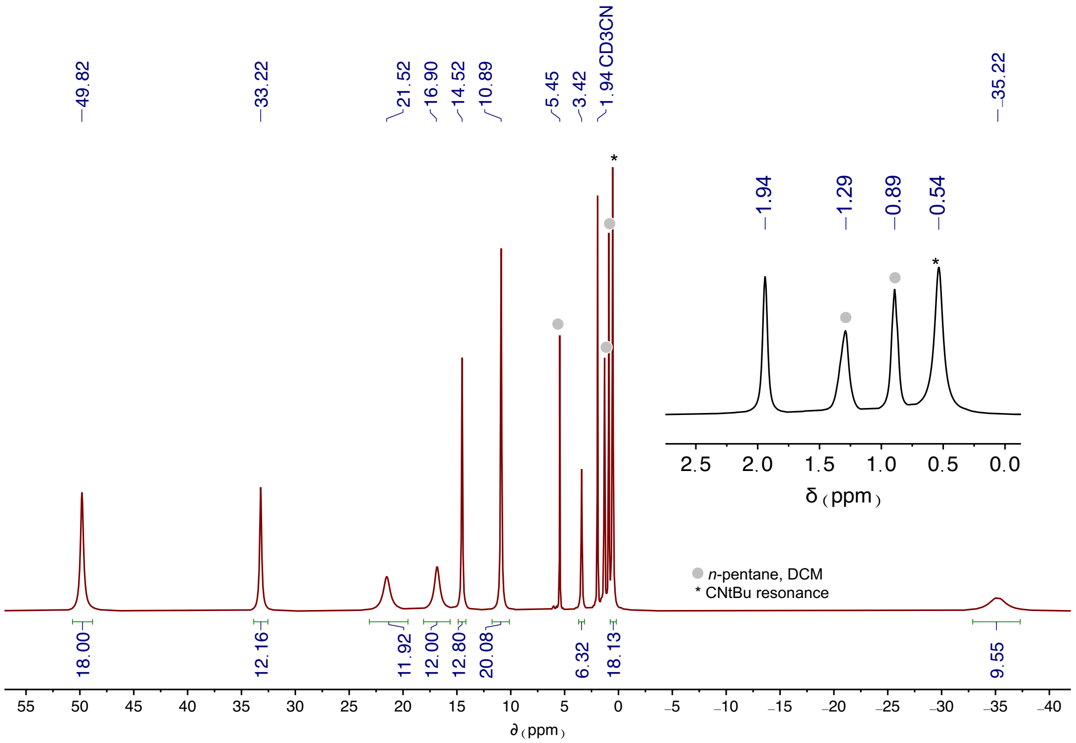

Figure S1. ^{1}H NMR (CD_3CN, 25 °C, 300 MHz) spectrum of [**1**(CN^{t}Bu)$_2$][PF_6].

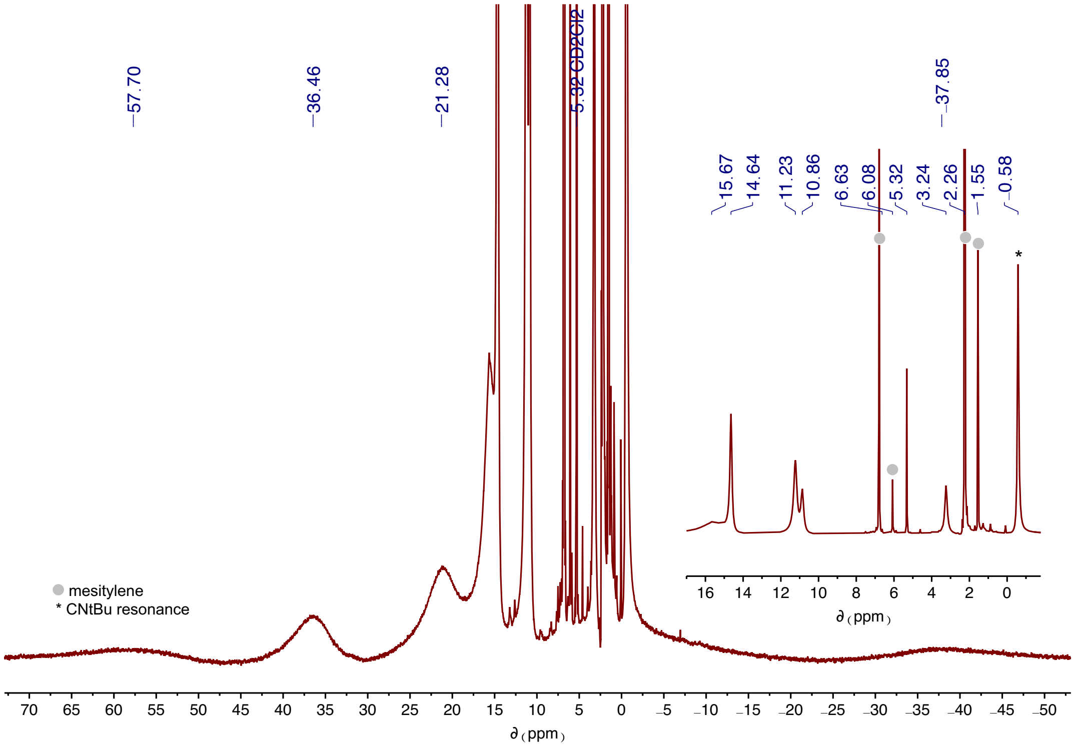

Figure S2. ^{1}H NMR (CD_2Cl_2, 25 °C, 300 MHz) spectrum of [**1**(CN^{t}Bu)$_2$][PF_6].

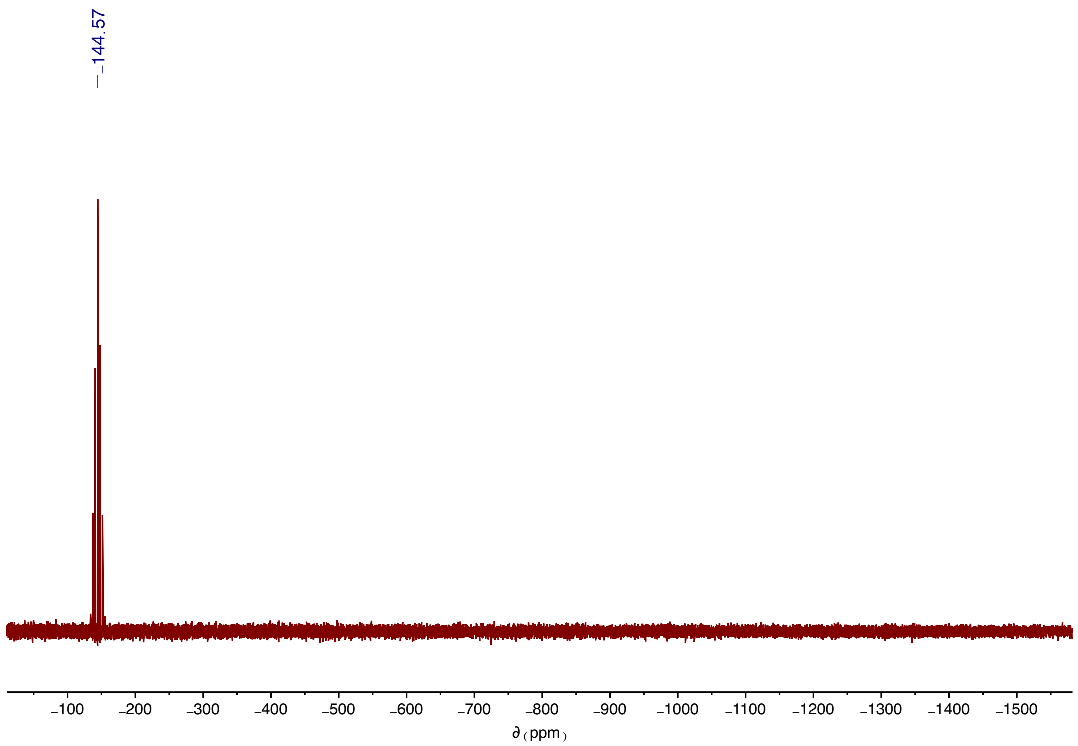

Figure S3. ^{31}P NMR (CD_2Cl_2, 25 °C, 202 MHz) spectrum of [**1**(CN^{t}Bu)$_2$][PF_6].

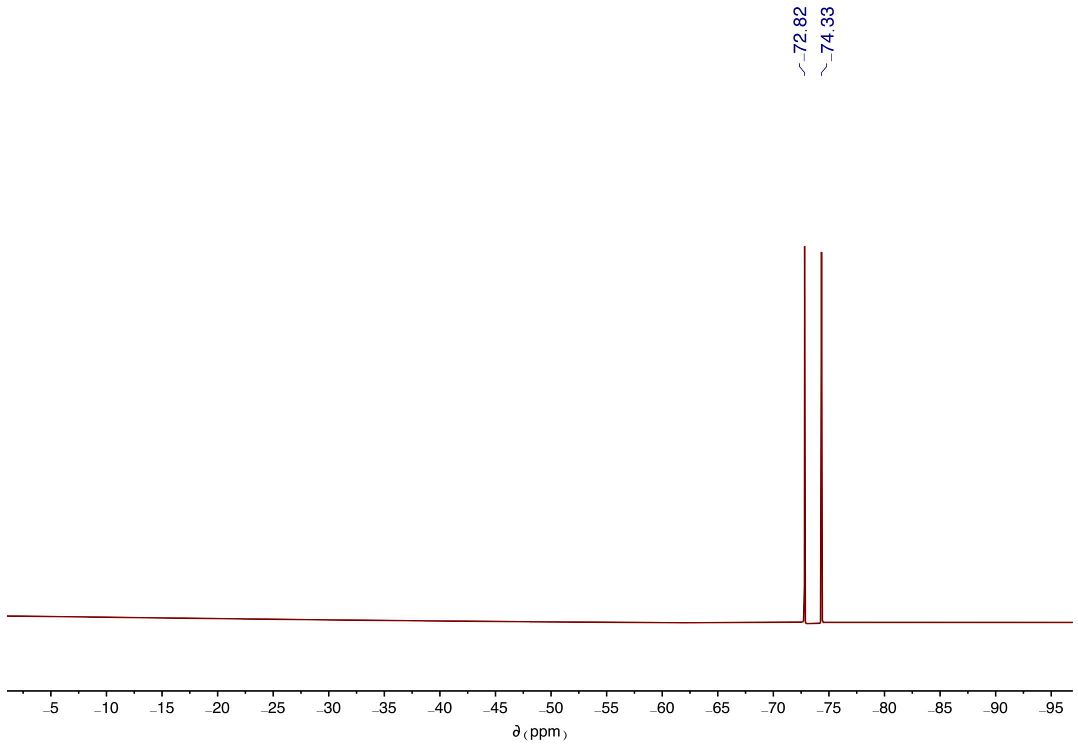

Figure S4. ^{19}F NMR (CD_2Cl_2, 25 °C, 471 MHz) spectrum of [**1**(CN^{t}Bu)$_2$][PF_6].

S2.2 Synthesis and Isolation of $[Fe_3(THF)_2Co_6Se_8L_6][PF_6]$ ($[\mathbf{1}(THF)_2][PF_6]$)

A 50 mL Schlenk flask equipped with a Teflon valve and magnetic stir bar was loaded with **1** (1.000 g, 0.345 mmol, 1 equiv) and THF (25 mL). The dark red-brown slurry was stirred at room temperature and DCM (8 mL) was added until the mixture was homogeneous. The solution was then cooled to −35 °C and ferrocenium hexafluorophosphate (0.115 g, 0.345 mmol, 1 equiv) was added portion wise with rapid stirring. The solution was brought to room temperature and stirred for 1 h, over the course of this time the solution adopted a dark red-purple color. The solution was passed through a plug of Celite on a fritted glass funnel and the filtrate was layered with *n*-pentane for bulk crystallization. The solution was kept still at room temperature for 16 hours, affording a crop of large, dark red prismatic crystals which collected on a fritted glass funnel and washed with additional *n*-pentane (3 x 6 mL). After removal of volatiles from these solids *in vacuo*, this process afforded [**1**(THF)$_2$][PF$_6$] as an analytically pure, crystalline solid (0.977 g, 0.307 mmol, 89%).

UV-vis-*n*IR (DCM, 25 °C) λ: 445 (ε = 39,420 M^{-1} cm^{-1}). ^{1}H NMR (CD_3CN, 300 MHz) δ: 50.16 (s, 18 H, $\nu_{1/2}$ = 65 Hz, tolyl-C***H***$_3$); 33.27 (s, 12H, $\nu_{1/2}$ = 69 Hz); 22.94 (s, 12H, $\nu_{1/2}$ = 247 Hz), 17.45 (s, 12H, $\nu_{1/2}$ = 208 Hz); 15.03 (s, 12H, $\nu_{1/2}$ = 55 Hz); 11.75 (s, 6H, $\nu_{1/2}$ = 40 Hz); 10.42 (s, 12H, $\nu_{1/2}$ = 62 Hz); 3.64 (s, 6H, $\nu_{1/2}$ = 36 Hz); 13.08 (s, 8H, $\nu_{1/2}$ = 44 Hz); 1.80 (s, 8H, $\nu_{1/2}$ = 40 Hz); −38.25 (s, $\nu_{1/2}$ = 550 Hz) ppm. ^{1}H NMR (CD_2Cl_2, 500 MHz) δ: 63.21 (s, $\nu_{1/2}$ = 161 Hz); 39.38 (s, $\nu_{1/2}$ = 119 Hz); 15.71 (s, $\nu_{1/2}$ = 158 Hz); 13.70 (s, $\nu_{1/2}$ = 31 Hz); 13.29 (s, $\nu_{1/2}$ = 146 Hz); 11.09 (s, $\nu_{1/2}$ = 23 Hz); 9.52 (s, $\nu_{1/2}$ = 47 Hz); 2.85 (s, $\nu_{1/2}$ = 57 Hz); 2.58 (s, $\nu_{1/2}$ = 23 Hz); 1.65 (s, $\nu_{1/2}$ = 31 Hz); −49.95 (s, $\nu_{1/2}$ = 388 Hz ppm. ^{31}P NMR (CD_2Cl_2, 202 MHz) δ: −144.37 (*h*, $^1J_{PF}$ = 722 Hz) ppm. Note: no other ^{31}P resonances observed within the range of ±2000 ppm. ^{19}F NMR (CD_2Cl_2, 471 MHz) δ: −73.12 (d, $^1J_{PF}$ = 712 Hz, $\nu_{1/2}$ = 130 Hz) ppm. ^{13}C NMR (CD_2Cl_2, 125 MHz): no peaks observed between ±400 ppm. μ_{eff} (Evans Method, CD_2Cl_2, 298K): 8.7(3) μ_B. Elemental analysis found (calc.) for [**1**(THF)$_2$][PF$_6$] (Formula: $C_{122}H_{118}Co_6F_6Fe_3N_6O_2P_7Se_8$): Co 11.05 (11.11), Se 20.01 (19.84), P 6.85 (6.81).

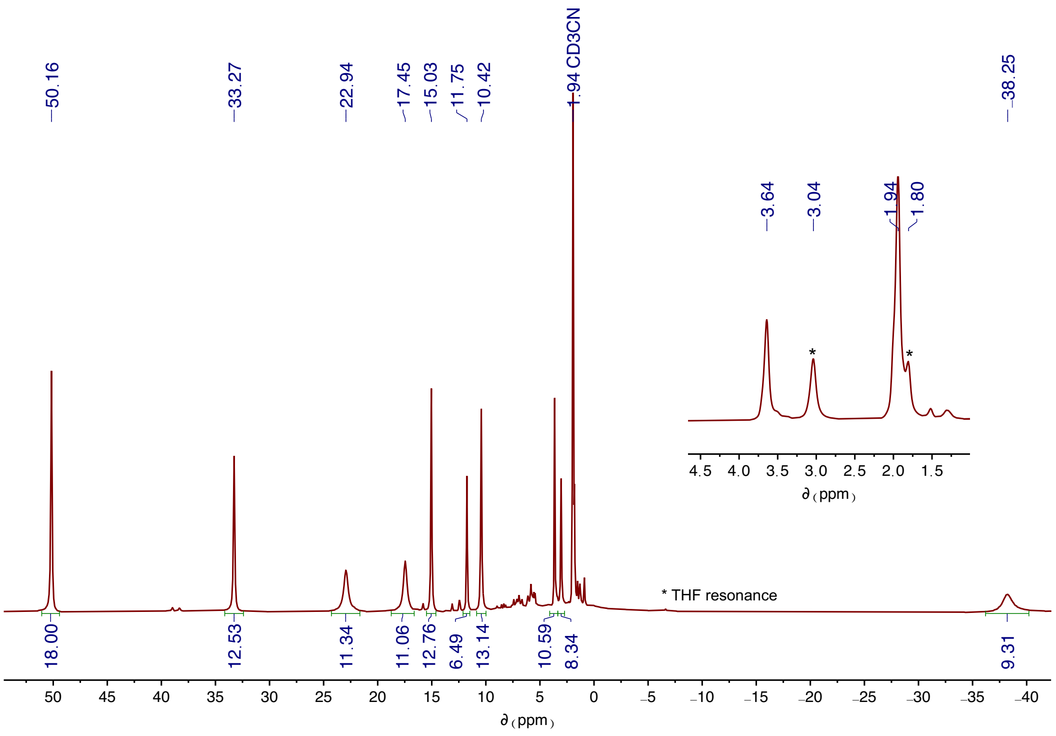

Figure S5. ^{1}H NMR (CD_3CN, 25 °C, 300 MHz) spectrum of [**1**(THF)$_2$][PF$_6$].

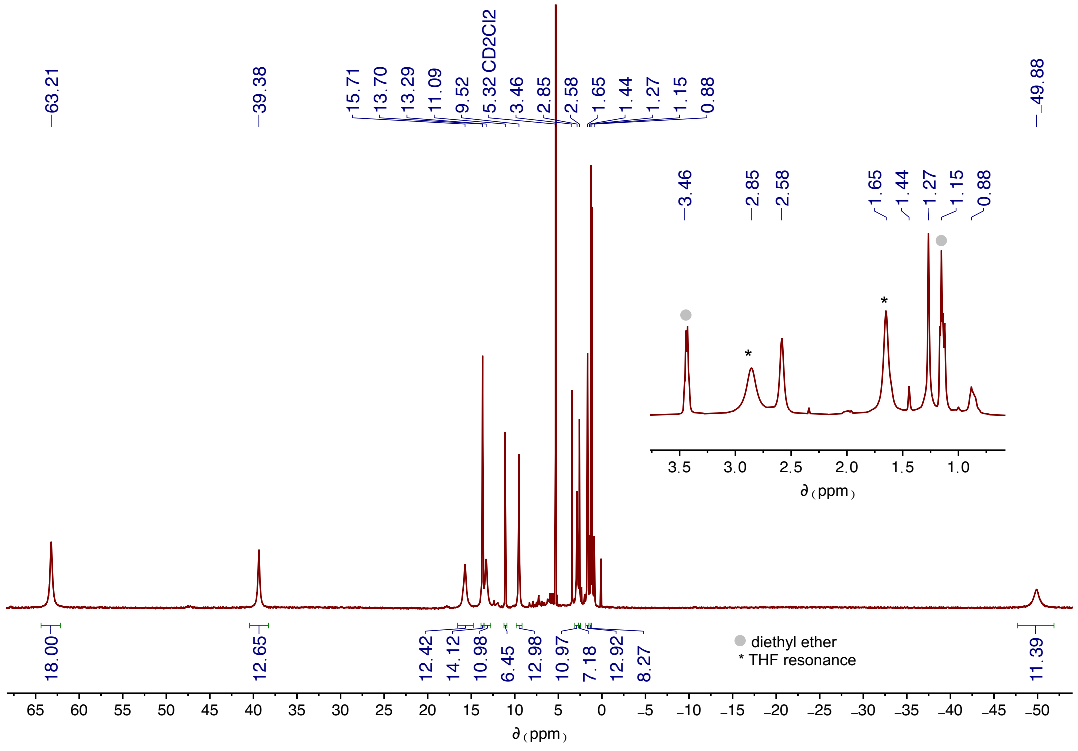

Figure S6. ^{1}H NMR (CD_2Cl_2, 25 °C, 500 MHz) spectrum of [**1**(THF)$_2$][PF_6].

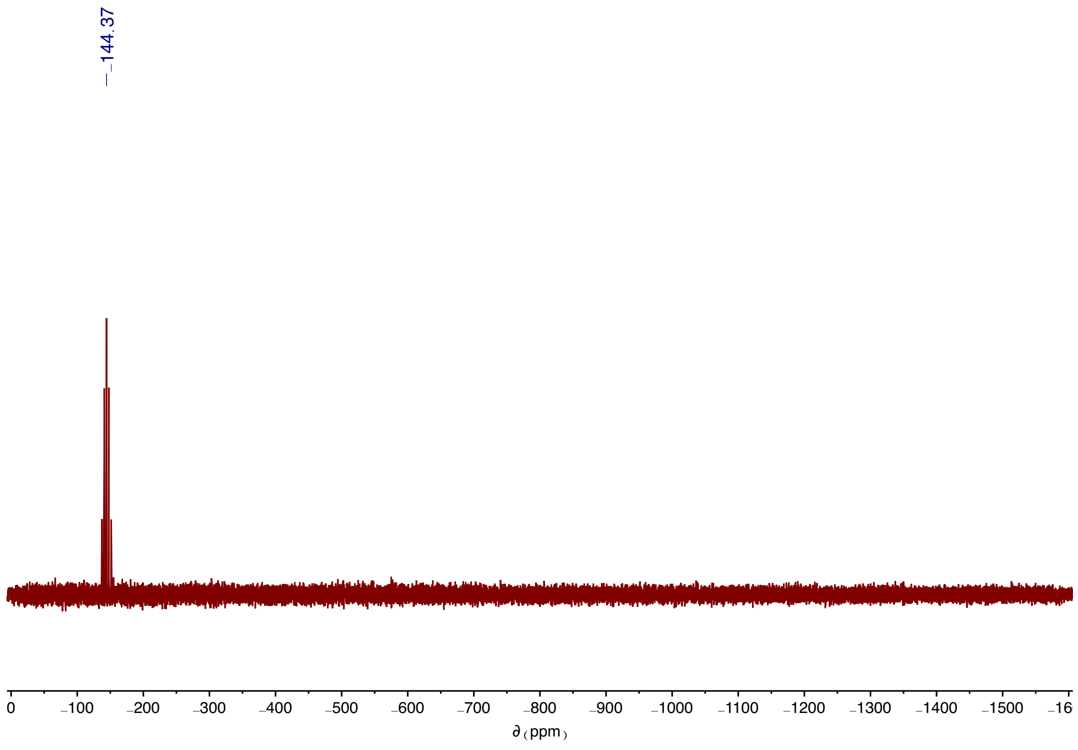

Figure S7. ^{31}P NMR (CD_2Cl_2, 25 °C, 202 MHz) spectrum of [**1**(THF)$_2$][PF_6].

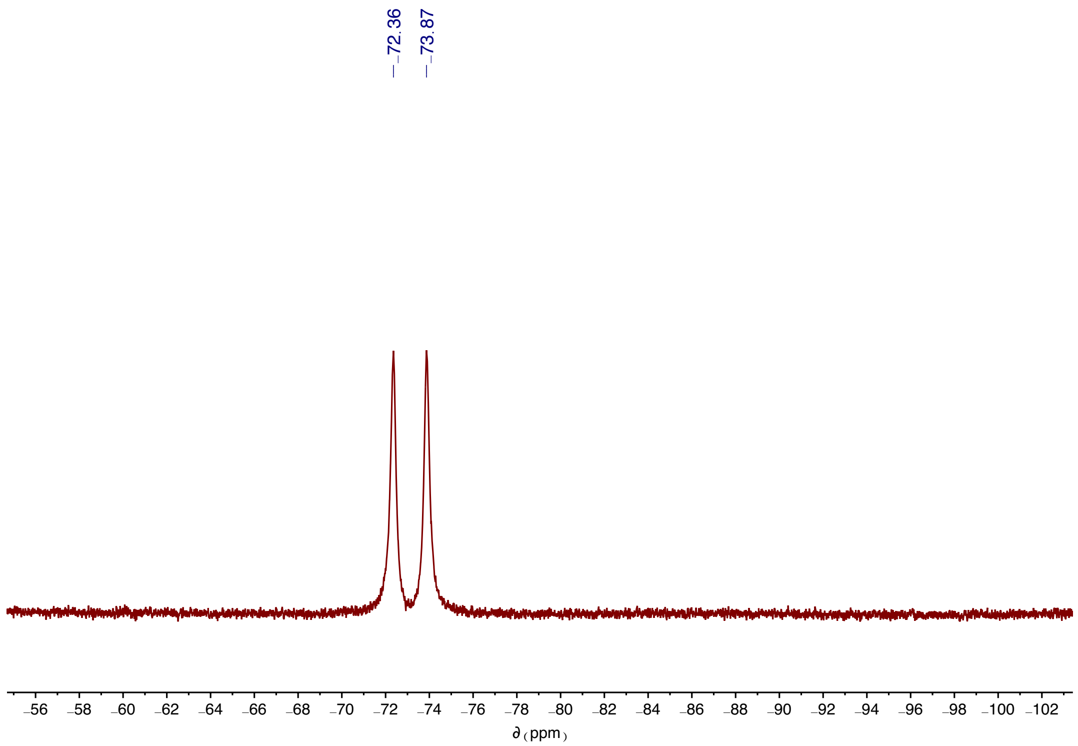

Figure S8. ^{19}F NMR (CD_2Cl_2, 25 °C, 470 MHz) spectrum of [**1**(THF)$_2$][PF_6].

S2.3 Synthesis and Isolation of [$Fe_3(MeCN)Co_6Se_8L_6$][OTf] (1(MeCN)(OTf))

A 100 mL round bottom flask equipped with a magnetic stir bar was loaded with **1** (0.600 g, 0.207 mmol, 1 equiv), AgOTf (0.053 g, 0.207 mmol, 1 equiv), acetonitrile (2 mL) and DCM (50 mL). The mixture was stirred at 25 °C for 2 h, and then passed through a plug of Celite in a fritted-glass funnel to remove silver triflate and silver metal. The filtrate was evaporated to dryness under reduced pressure, and the product was extracted in acetonitrile (10 mL). The acetonitrile extracts were layered with diethyl ether and stored at –35 °C for 24 h to yield black needles. These were collected on a fritted-glass filter and volatiles were removed under reduced pressure to yield **1**(MeCN)(OTf) (0.331 g, 0.107 mmol, 48%).

UV-vis-*n*IR (DCM, 25 °C) λ: 439 (ε = 38,380 M^{-1} cm^{-1}). ^{1}H NMR (CD_3CN, 300 MHz) δ: 50.07 (s, 18 H, $\nu_{1/2}$ = 31 Hz, tolyl-C$\boldsymbol{H}_3$); 32.23 (s, 12H, $\nu_{1/2}$ = 51 Hz); 22.76 (s, 12H, $\nu_{1/2}$ = 225 Hz), 17.38 (s, 12H, $\nu_{1/2}$ = 183 Hz); 15.01 (s, 12H, $\nu_{1/2}$ = 39 Hz); 11.72 (s, 6H, $\nu_{1/2}$ = 27 Hz); 10.41 (s, 12H, $\nu_{1/2}$ =42 Hz); 3.05 (s, 6H, $\nu_{1/2}$ = 31.5 Hz); −38.12 (s, 12H, $\nu_{1/2}$ = 471 Hz) ppm. ^{1}H NMR (CD_2Cl_2, 300 MHz) δ: 61.41 (s, $\nu_{1/2}$ = 1140 Hz, tolyl-C$\boldsymbol{H}_3$); 38.66 (s, $\nu_{1/2}$ = 474 Hz); 19.33 (s, $\nu_{1/2}$ = 902 Hz), 14.66 (s, 12H, $\nu_{1/2}$ = 55 Hz); 12.10 (s, $\nu_{1/2}$ = 390 Hz); 11.73 (s, $\nu_{1/2}$ = 59 Hz); 8.84 (s, $\nu_{1/2}$ = 59 Hz); 1.91 (s, $\nu_{1/2}$ = 37 Hz); 1.82 (s, $\nu_{1/2}$ = 52 Hz, MeCN-C$\boldsymbol{H}_3$); −48.56 (s, $\nu_{1/2}$ = 1414 Hz) ppm. ^{19}F NMR (CD_3CN, 471 MHz) δ: −79.11 (s, $\nu_{1/2}$ = 272 Hz) ppm; (C_6D_6, 471 MHz) δ: +6.47 (s, $\nu_{1/2}$ = 258 Hz) ppm; (CD_2Cl_2, 471 MHz) δ: −63.00 (s, $\nu_{1/2}$ = 787 Hz) ppm. ^{31}P NMR (CD_2Cl_2, 202 MHz) δ: no peaks observed between ±2000 ppm. ^{13}C NMR (CD_2Cl_2, 125 MHz): no peaks observed between ±400 ppm. μ_{eff}(Evans Method, CD_2Cl_2, 298K): 8.8(3) μ_B. Elemental analysis found (calc.) for **1**(MeCN)(OTf) (Formula: $C_{117}H_{105}Co_6F_3Fe_3N_7O_3P_6SSe_8$): Co 11.32 (11.46), Se 20.30 (20.48), P 5.91 (6.02).

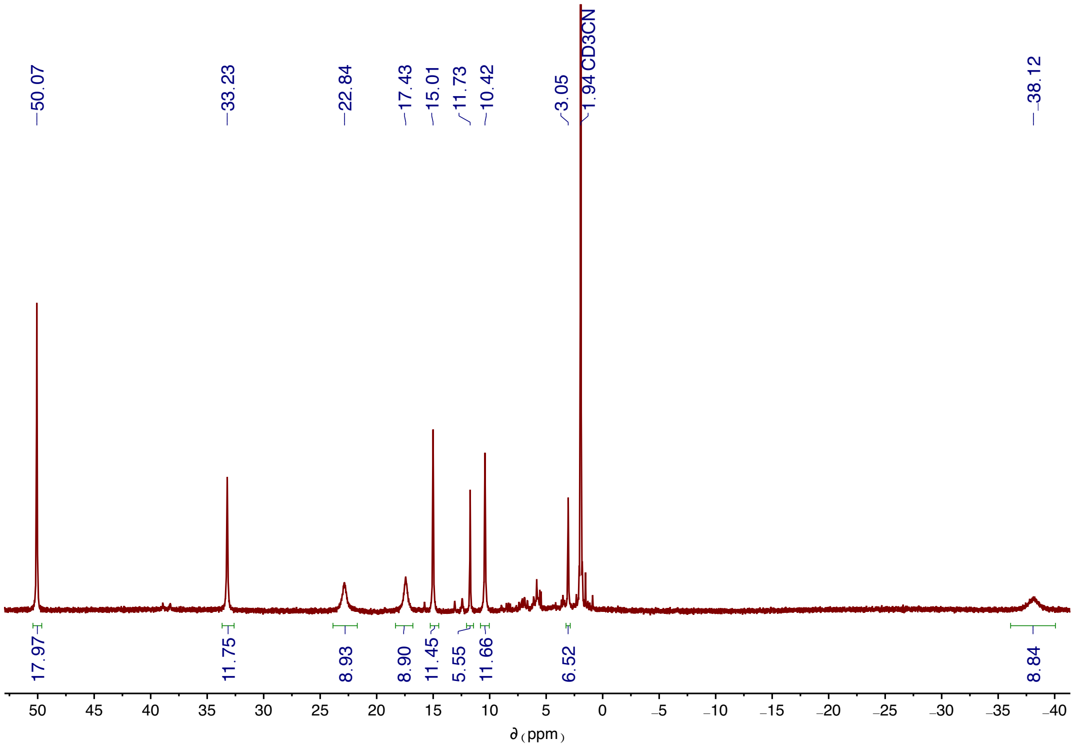

Figure S9. ^{1}H NMR (CD_3CN, 25 °C, 300 MHz) spectrum of **1**(MeCN)(OTf).

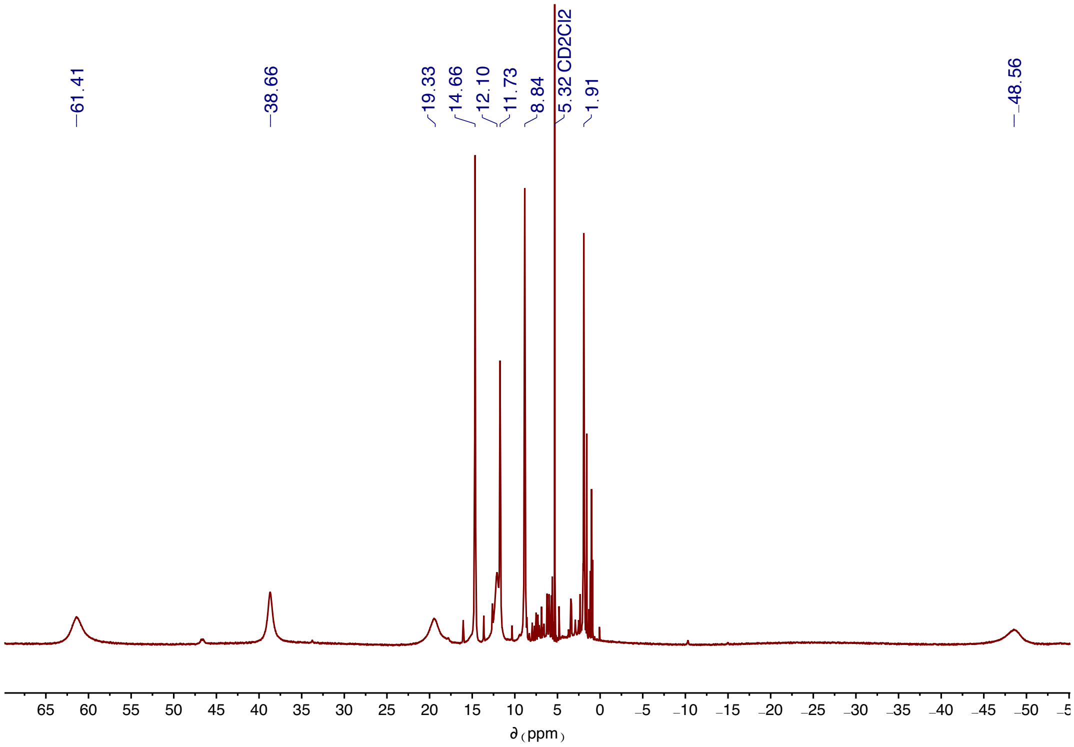

Figure S10. ^{1}H NMR (CD_2Cl_2, 25 °C, 300 MHz) spectrum of **1**(MeCN)(OTf).

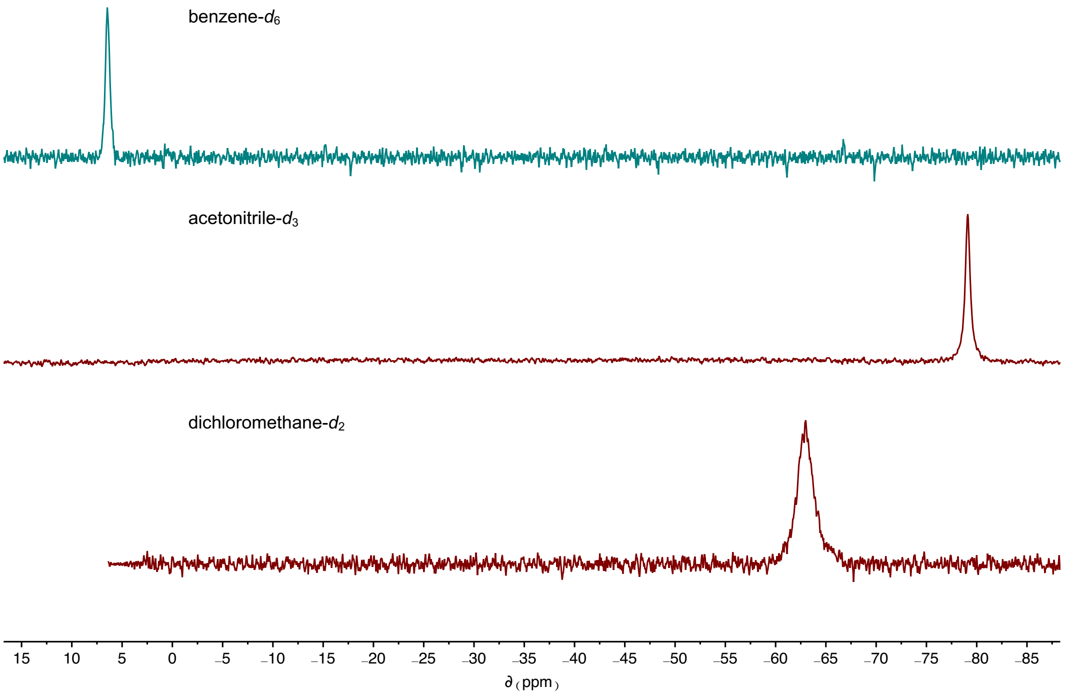

Figure S11. ^{19}F NMR (25 °C, 470 MHz) spectra of **1**(MeCN)(OTf) recorded in C_6D_6 (top), CD_3CN (middle), and CD_2Cl_2 (bottom).

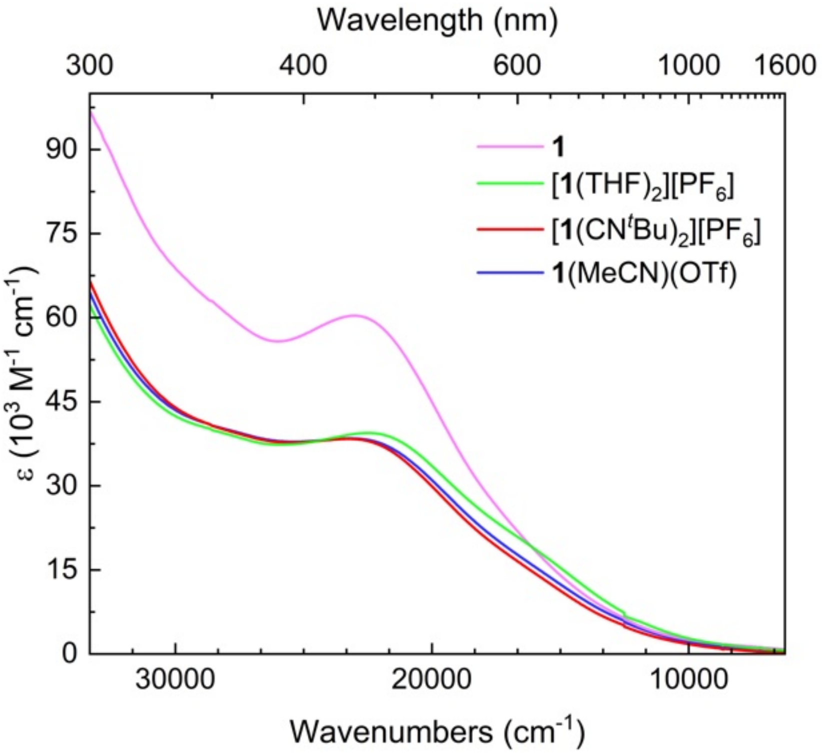

Figure S12. UV-vis-*n*IR absorption spectra of **1**, [**1**(THF)$_2$][PF_6], [**1**(CN^{t}Bu)$_2$][PF_6], and **1**(MeCN)(OTf) recorded in DCM.

S2.4 NMR Titration of [1(CN^{t}Bu)$_2$][PF$_6$] with THF

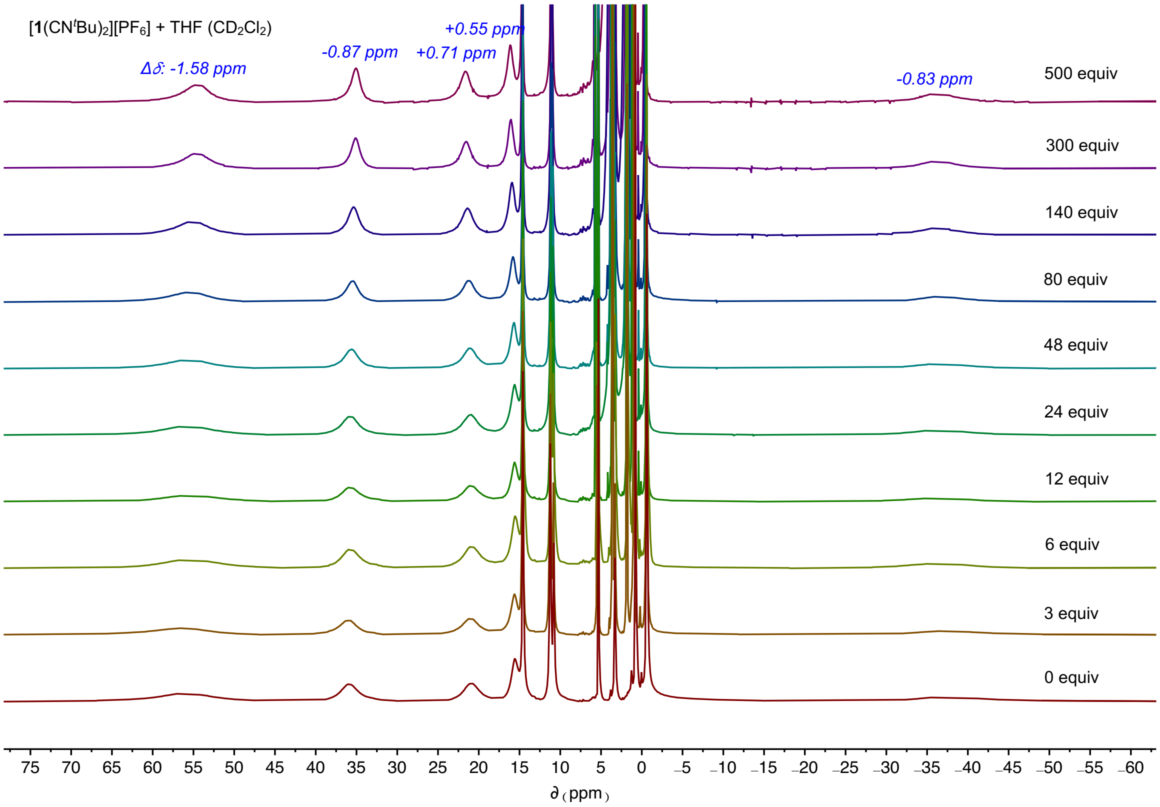

Figure S13. ^{1}H NMR (CD_2Cl_2, 25 °C, 500 MHz) spectra of [**1**(CN^{t}Bu)$_2$][PF$_6$] treating with varying equivalents (0-500 equiv) of THF at 25 °C. The variation in chemical shift ($\Delta\delta$) for several paramagnetic resonances upon increasing THF amounts from 0 to 500 equivalents are denoted in blue. The minimal perturbation of observed NMR profile indicates both THF and CN^{t}Bu remain in rapid exchange for each measurement.

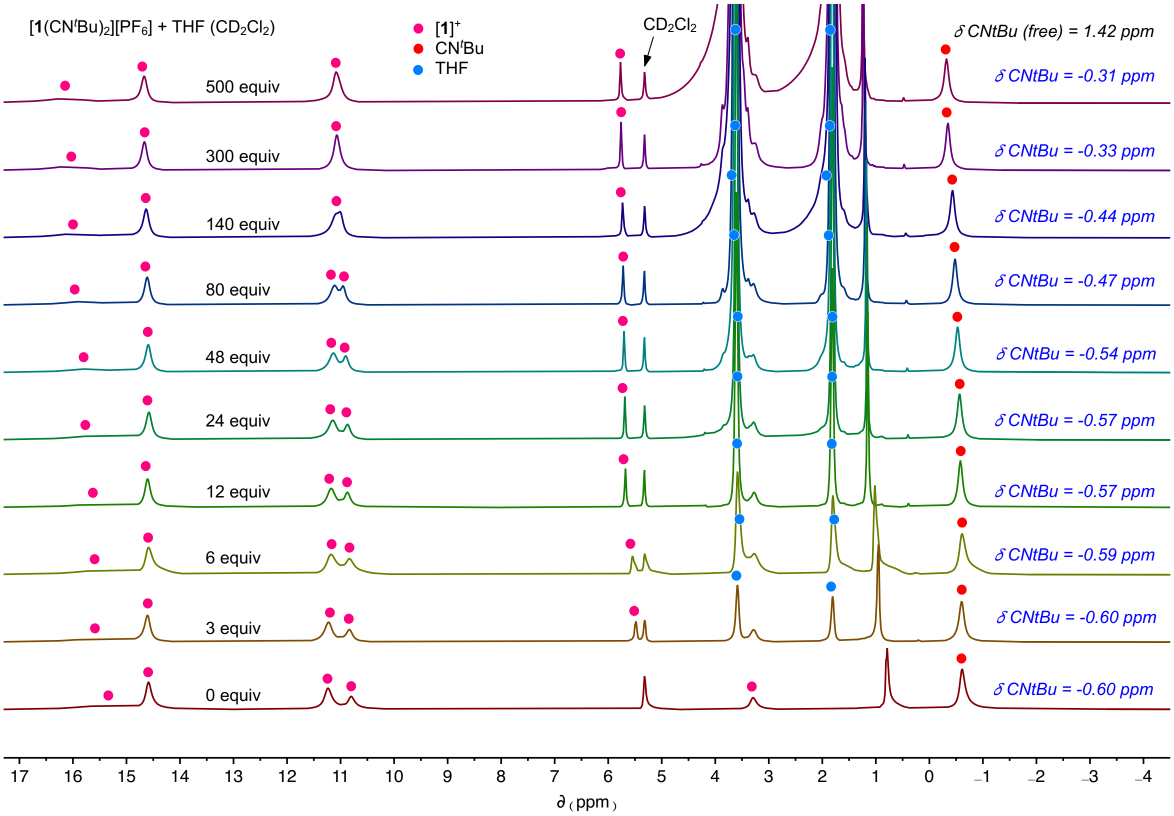

Figure S14. ^{1}H NMR (CD_2Cl_2, 25 °C, 500 MHz) spectra of [**1**(CN^{t}Bu)$_2$][PF_6] treating with varying equivalents (0-500 equiv) of THF at 25 °C, highlighting the diamagnetic region. A singular, paramagnetically broadened singlet is observed for CN^{t}Bu even in the presence of 500 equivalents of THF, indicating ligand exchange more rapidly than can be resolved in the NMR timescale.

S3 Fourier Transform Infrared Spectroscopy

The solution-phase ligand substitution dynamics of [**1**]$^+$ with *tert*-butyl isocyanide (CN^{t}Bu) were studied by monitoring the isocyanide stretching frequency (ν_{CN}) using a Fourier transform infrared spectroscopy (FTIR). Solutions of [**1**(THF)$_2$][PF_6] (7.8 mM in THF or DCM) were treated with varying amounts of CN^{t}Bu (0.1–2.0 equiv; added via microsyringe from a 0.5 M THF or DCM stock solution). Separate spectra were recorded for [**1**(CN^{t}Bu)$_2$][PF_6] dissolved in neat THF or DCM, and for neat THF or DCM for solvent subtraction. All samples were stirred until homogeneous and then transferred to an air-free 2 mm pathlength solution IR cell equipped with PTFE caps and CaF_2 windows. All FTIR spectra were collected at room temperature (~298 K). Each spectrum was background corrected to account for atmospheric contributions to the IR absorption pattern followed by solvent subtraction.

In neat THF, two major CN stretches are resolved at 2184 and 2135 cm^{-1}, which correspond to bound Fe(CN^{t}Bu) and free CN^{t}Bu, respectively. These CN stretching frequencies are slightly blue-shifted in DCM solutions (2185 and 2140 cm^{-1}), and analysis of [**1**(CN^{t}Bu)$_2$][PF_6] in DCM reveals a single major peak at 2185 cm^{-1}, with no appreciable amount of free CN^{t}Bu. To assess the relative concentrations of free and bound CN^{t}Bu, these peaks were fit to a series of four gaussian curves, accounting for the contribution of minor overtone bands at ~2160 and 2118 cm^{-1}, which reliably reproduced the recorded line shape with

a low residual. The major peaks for bound Fe(CN^{t}Bu) and free CN^{t}Bu were integrated, and the ratio of their areas was used to calculate concentrations via relation to the known total concentrations of CN^{t}Bu and [**1**]$^+$.

In neat coordinating solvent, the displacement of CN^{t}Bu by THF at an isolated Fe center is described by the equilibrium constant:

$$Fe(CNtBu) + THF \rightleftarrows Fe(THF) + CNtBu$$

$$K_{eq} = \frac{[Fe(THF)][CNtBu]}{[Fe(CNtBu)]}$$

Under the assumption that in neat THF all Fe edges are bound by either CN^{t}Bu or THF, the concentration of Fe(THF) can be treated as [Fe(THF)] = [Fe_{Tot}] – [Fe(CN^{t}Bu)]. Each equivalent of [**1**]$^+$ introduces three Fe edges, allowing for the determination of total Fe edge concentration, [Fe_{Tot}].

To solve for the remaining unknowns, [CN^{t}Bu] and [Fe(CN^{t}Bu)], isocyanide stretching (ν_{CN}) peaks are fit to gaussian curves and their peak area is integrated. The unknown concentrations are then determined by multiplying a known total volume of isocyanide ([CN^{t}Bu]$_{Tot}$) by the area ratio for the respective ν_{CN} peak:

$$[Fe(CNtBu)] = [CNtBu]_{Tot}\ x\ \frac{Area_{Fe(CNtBu)}}{Area_{CNtBu} + Area_{Fe(CNtBu)}}$$

$$[CNtBu] = [CNtBu]_{Tot}\ x\ \frac{Area_{CNtBu}}{Area_{CNtBu} + Area_{Fe(CNtBu)}}$$

Table S1. Equilibrium constants for the displacement of CN^{t}Bu by THF at an isolated Fe edge of [**1**]$^+$, as determined from gaussian fitting of solution-phase IR data.

CN^{t}Bu Added (equiv)	K_{eq} (M)
0.5	1.67
0.75	2.01
1.0	1.66
1.5	1.68
2	1.50
Average	1.70 ± 0.18

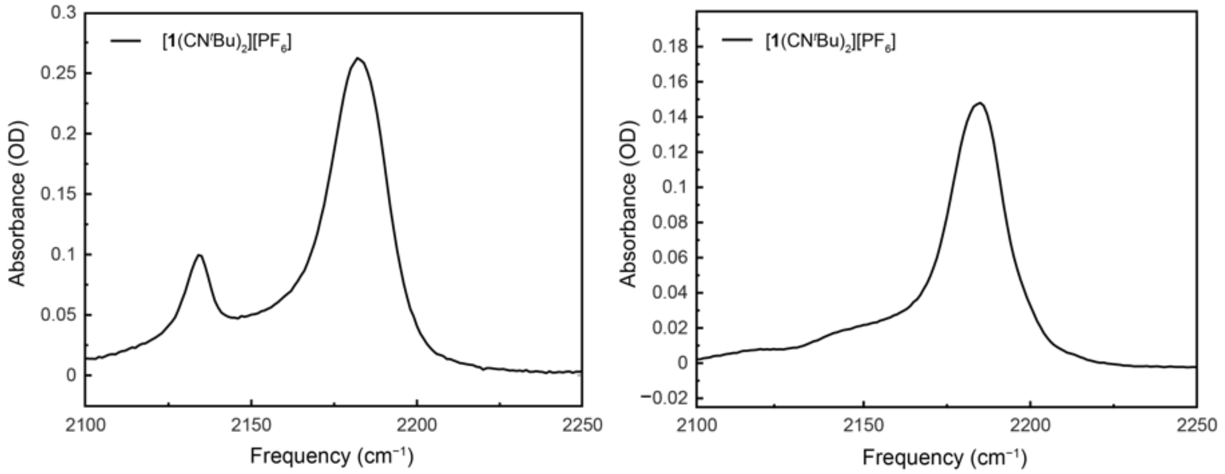

Figure S15. FTIR absorption spectra for [**1**(CN^{t}Bu)$_2$][PF$_6$] collected in neat THF (left) and DCM (right).

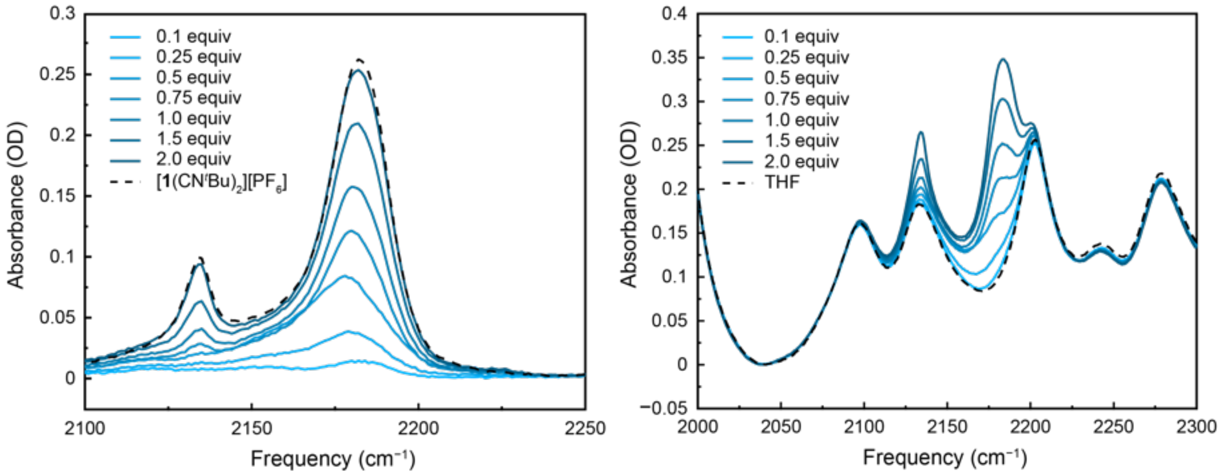

Figure S16. FTIR absorption spectra recorded for [**1**(THF)$_2$][PF$_6$] upon titration with varying equivalents of CN^{t}Bu in neat THF both after (left) and before solvent subtraction (right). Close overlap of the spectra obtained for [**1**(THF)$_2$][PF$_6$] in the presence of 2 equiv CN^{t}Bu with that of pure [**1**(CN^{t}Bu)$_2$][PF$_6$] in THF suggests an equilibrium is quickly established upon addition of CN^{t}Bu.

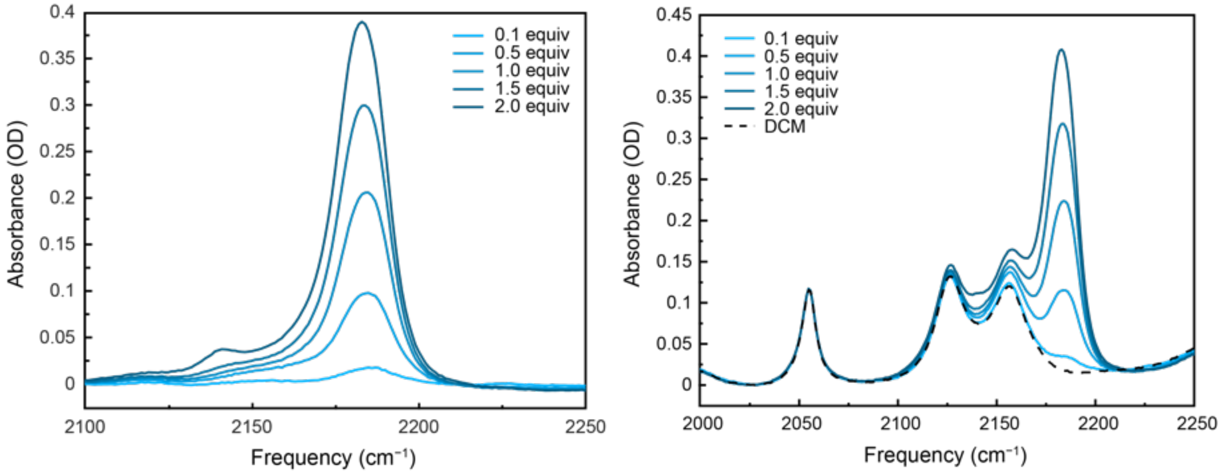

Figure S17. FTIR absorption spectra recorded for [**1**(THF)$_2$][PF$_6$] upon titration with varying equivalents of CN^{t}Bu in neat DCM after (left) and before solvent subtraction (right).

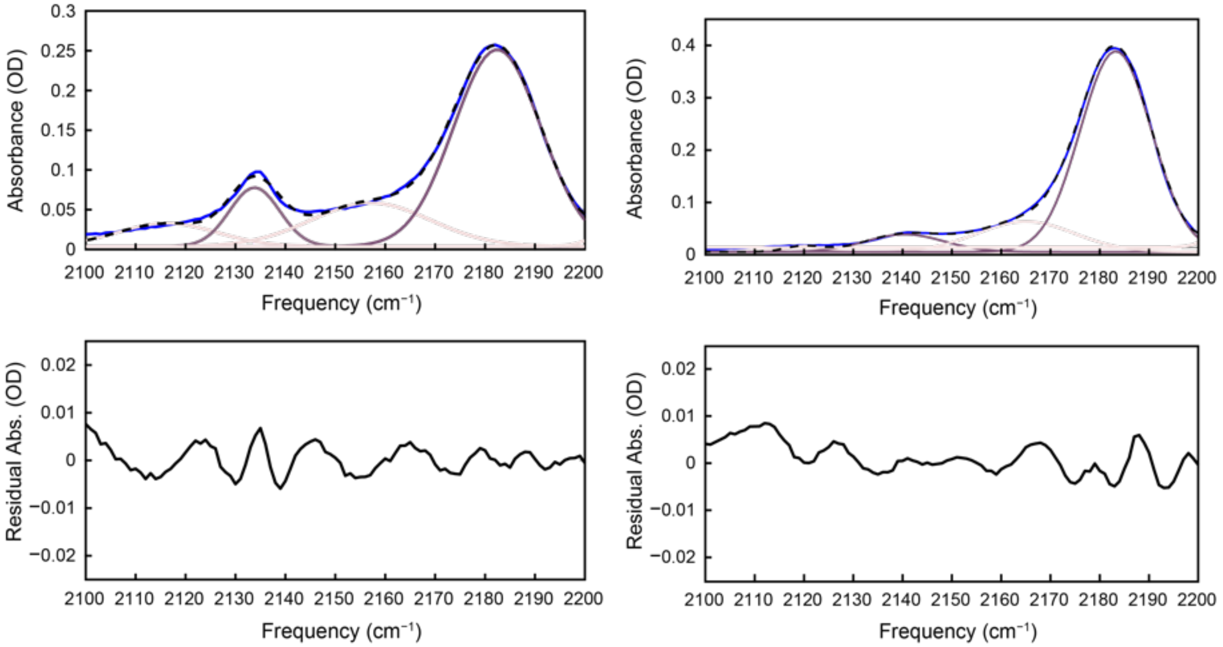

Figure S18. Representative gaussian fits used to assess the concentrations of bound and free CN^{t}Bu (dark gray curves) in both THF (left) and DCM (right). Light grey curves are attributed to overtone bands and were necessary to achieve a good fit to the line shape for the major CN^{t}Bu stretches.

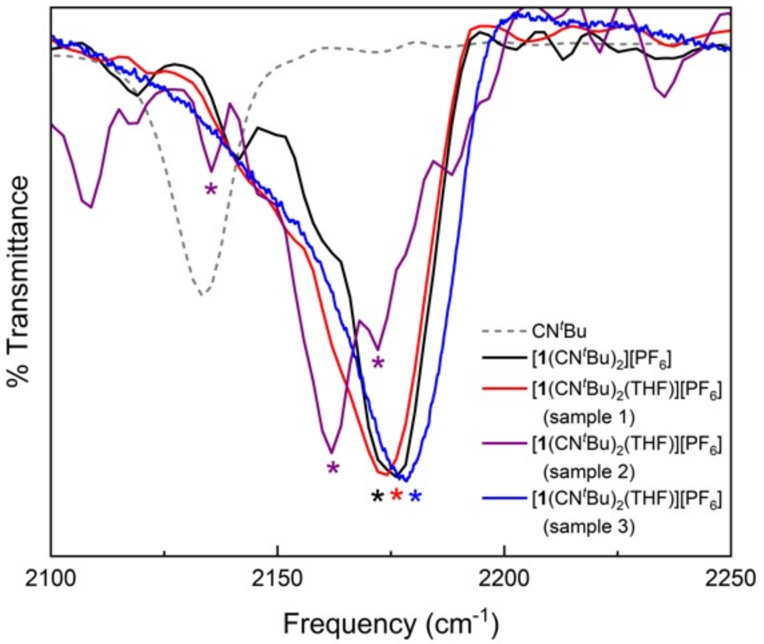

Figure S19. **Infrared spectra of [1(CN^{t}Bu)$_2$(THF)][PF$_6$]** (Samples 1-3), plotted in comparison to [**1**(CN^{t}Bu)$_2$][PF$_6$] (black trace; ATR-FTIR, with anvil press, solid sample) and CN^{t}Bu (dashed trace; ATR-FTIR, dropcast neat). Three different batches of fresh crystals of [**1**(CN^{t}Bu)$_2$(THF)][PF$_6$] were measured to capture any dissociated CN^{t}Bu: Sample 1 (red trace; ATR-FTIR; crystals crushed and measured under anvil press); Sample 2 (purple trace; ATR-FTIR; crystals not crushed and measured without anvil press); Sample 3 (blue trace; transmittance IR; crystals dispersed in Nujol and sandwiched between NaCl plates). Select CN stretching frequencies (cm^{-1}): 2135, 2161, 2172 (purple); 2174 (red); 2178 (blue); 2176 (black); 2133 (gray). When ATR-FTIR spectra are recorded without crushing the crystals (i.e., Sample 2), a peak is observed close to that expected for free isocyanide. However, the ATR diamond exhibits significant noise in this region, so this peak at 2135 cm^{-1} may not be of statistical significance.

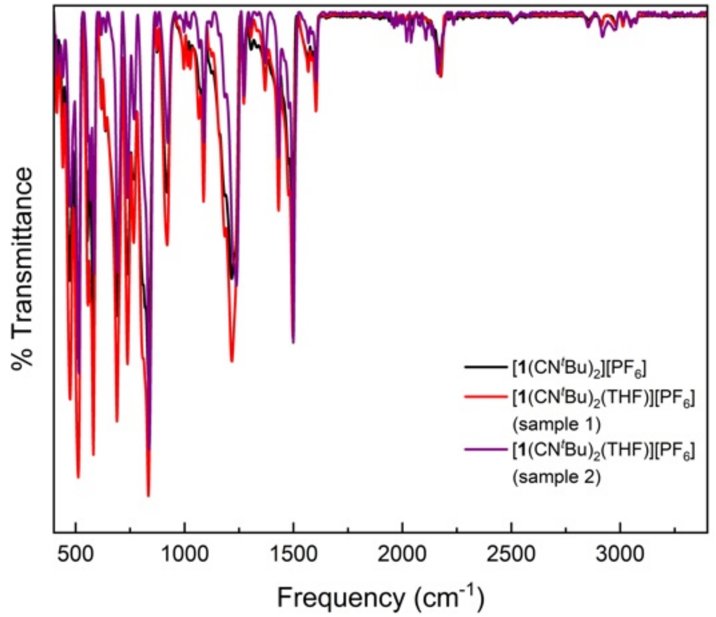

Figure S20. ATR-FTIR spectra of polycrystalline [**1**(CN^{t}Bu)$_2$][PF$_6$] plotted in comparison to two samples of [**1**(CN^{t}Bu)$_2$(THF)][PF$_6$]: Sample 1 (red trace; ATR-FTIR; crystals crushed and measured under anvil press); Sample 2 (purple trace; ATR-FTIR; crystals not crushed and measured without anvil press). This illustrates that many of the same peaks are retained for [**1**(CN^{t}Bu)$_2$(THF)][PF$_6$] both with and without the use of the anvil press. However, significant noise is observed near the CN-stretching region (~1950-2200 cm^{-1}).

S4 Magnetic Characterization of [1(CN^{t}Bu)$_2$][PF$_6$]

dc Magnetic susceptibility data for [**1**(CN^{t}Bu)$_2$][PF$_6$] were collected on warming from 5 to 300 K under applied fields of 0.1, 0.5, and 1 T using a Quantum Design Inc. PPMS DynaCool equipped with a 14 T magnet. To prepare these samples, polycrystalline material was loaded within a VSM powder sample holder (P125E, Quantum Design Inc.) that was carefully sealed with a thin loop of epoxy. Crushed crystalline material placed under vacuum at 60 °C for 2 hours prior to analysis. All magnetic susceptibility data was corrected for diamagnetic contributions from the analyte calculated using Pascal's constants,[3] as well as for the susceptibility of the capsule and sample holder. Magnetization data was recorded at 50 K between –14 and 14 T to confirm the absence of any ferromagnetic impurities. Further, variable-temperature *dc* magnetic susceptibility does not decrease with increasing field strength, providing further support that the samples are free of ferromagnetic impurities. Susceptibility data (0.5 T applied field) for [**1**(CN^{t}Bu)$_2$][PF$_6$] provides a reasonable value for a 3 x Fe(II) (s = 2), 1 x $Co_6Se_8^{1+}$ (s = ½) spin system, with S_{Tot} = 13. This data was modeled using PHI in order to extract the g-value (g = 2.03, R^2 = 1.18), but a quantitative model was not developed due to the complexity of the system.[4] Variable-temperature variable-field reduced magnetization data supports the presence of a high-spin ground state, but magnetic saturation is not reached under applied fields of up to 7 T and temperatures down to 2.5 K. Accordingly, the selected spin multiplicity for DFT analysis was assigned based on the observed magnetic susceptibility at 298 K.

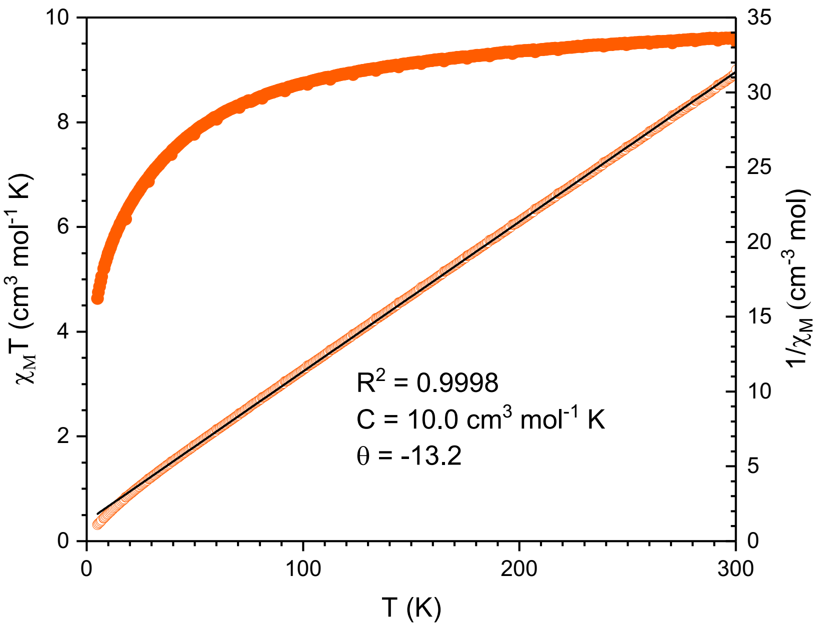

Figure S21. Variable-temperature *dc* magnetic susceptibility ($\chi_M T$, filled circles) and inverse molar susceptibility ($1/\chi_M$, hollow circles) data recorded for [**1**(CN^{t}Bu)$_2$][PF_6] upon warming from 5 to 300K with an applied field of 0.5 T. A linear fit to the data collected between 25 and 150 K was used to calculate Curie (C) and Weiss (θ) constants.

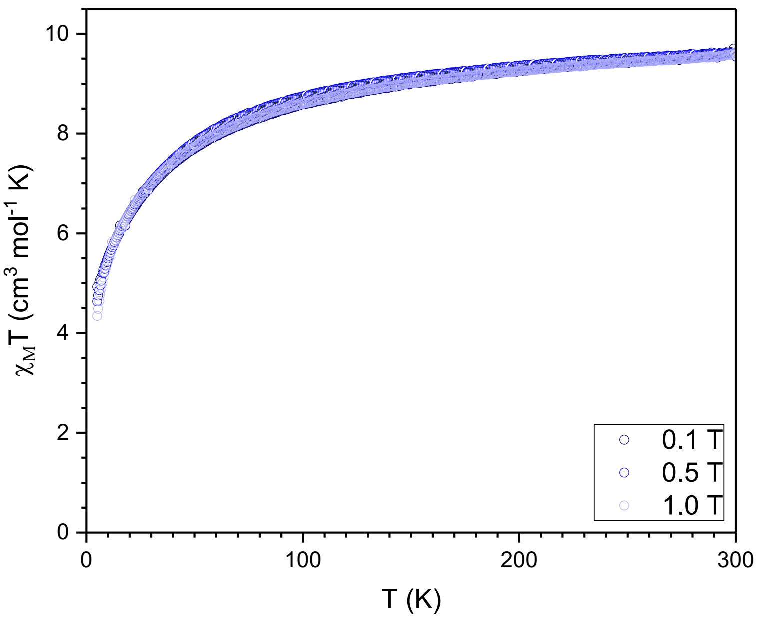

Figure S22. Variable-temperature *dc* magnetic susceptibility ($\chi_M T$, filled circles) data recorded for [**1**(CN^{t}Bu)$_2$][PF_6] upon warming from 5 to 300K under applied fields of 0.1, 0.5, and 1.0 T.

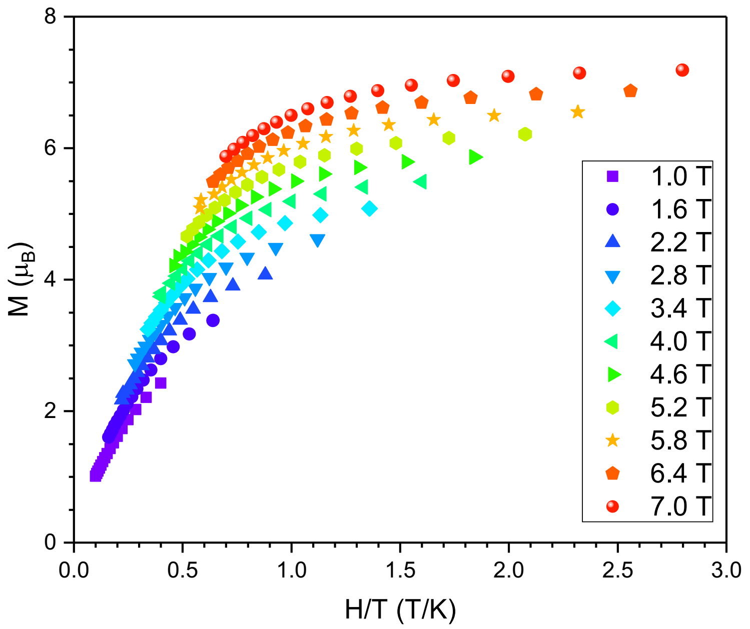

Figure S23. Variable-temperature, variable-field reduced magnetization data for [**1**(CN^{t}Bu)$_2$][PF$_6$], collected upon warming from 2.5 to 10 K at each applied field. This plot confirms the presence of a high-spin ground state, but the sample resists magnetic saturation under applied magnetic fields up to 7.0 T.

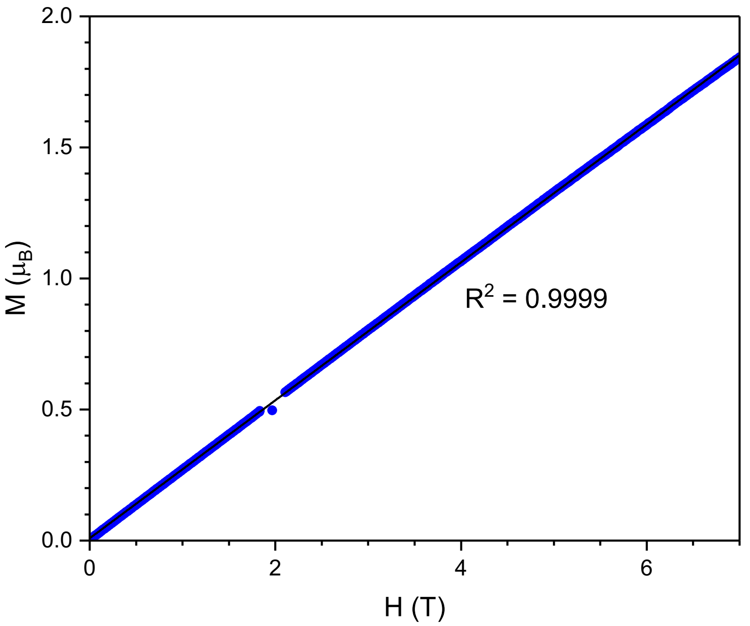

Figure S24. Magnetization of [**1**(CN^{t}Bu)$_2$][PF$_6$] recorded on sweeping the applied field from 0 and 7 T at 50 K. This plot confirms the absence of ferromagnetic impurities.

S5 ^{57}Fe Mössbauer Spectroscopy

Zero-field low temperature ^{57}Fe Mössbauer measurements were performed using a See Co. MS4 Mössbauer spectrometer integrated with a Janis SVT-400T He/N2 cryostat for measurements at 80 K. Isomer shifts were determined relative to α-Fe at 298 K. All Mössbauer spectra were fit using the program WMoss (SeeCo).

Table S2. Zero-field ^{57}Fe Mössbauer parameters for [**1**(CN^{t}Bu)$_2$][PF$_6$], [**1**(THF)$_2$][PF$_6$] and [**1**(CN^{t}Bu)$_2$(THF)][PF$_6$] collected at 80 K.

**Compound**	**δ (mm/s)**	**|ΔE$_Q$| (mm/s)**
[**1**(CN^{t}Bu)$_2$][PF$_6$][a]		
component 1 (60%)	0.71	1.90
component 2 (40%)	0.61	1.23
[**1**(THF)$_2$][PF$_6$]		
component 1 (35%)	0.65	1.66
component 2 (32%)	0.84	1.40
component 3 (33%)	0.61	0.64
[**1**(CN^{t}Bu)$_2$(THF)][PF$_6$]		
component 1 (57%)	0.71	1.90
component 2 (34%)	0.68	1.08
component 3 (8%)	0.61	1.23

[a]Collected at 90 K. [b]Component 4 stems from an unidentified ferrous impurity.

S6 X-ray Diffraction Studies

Single crystals suitable for X-ray analysis, grown according to methods detailed below, were coated in deoxygenated paratone oil and mounted on a 20 µm CryoLoop™ (Hampton Research, 18 mm mount, 0.2 to 0.3 mm loop diameter). Data was collected on a Bruker APEX II single crystal X-ray diffractometer, with a Mo source at 100 K (unless noted otherwise). Data was integrated and scaled using SAINT, SADABS within the APEX2 software package by Bruker.[5] Solution by direct methods (SHELXT[6] or SIR97[7,8]) produced a complete heavy atom phasing model consistent with the proposed structure. Structures were completed by difference Fourier synthesis with SHELXL.[9–11] Scattering factors are from Waasmair and Kirfel.[12] Hydrogen atoms were placed in geometrically idealized positions and constrained to ride on their parent atoms with C–H distances in the range 0.95–1.00 Å. Isotropic thermal parameters U_{eq} were fixed such that they were 1.2U_{eq} of their parent atom Ueq for CHs and 1.5U_{eq} of their parent atom U_{eq} in case of methyl groups. All non-hydrogen atoms were refined anisotropically by full-matrix least-squares.

S6.1 [Fe$_3$(THF)$_2$Co$_6$Se$_8$L$_6$][PF$_6$] ([1(THF)$_2$][PF$_6$])

Dark red, prismatic crystals of [**1**(THF)$_2$][PF$_6$] suitable for single-crystal X-ray analysis were grown by vapor diffusion of diethyl ether into a solution of **1** (1 equiv) and FcPF$_6$ (1 equiv) in THF at 25 °C. The compound crystallizes in the monoclinic space group P 21/c, with an asymmetric unit consisting of a singular [**1**(THF)$_2$]$^+$ cluster accompanied by a [PF$_6$]$^-$ counterion. One of the amidophosphine phenyl groups

as well as one *p*-tolyl substituent exhibit rotational disorder and as a result they were modeled over two positions.

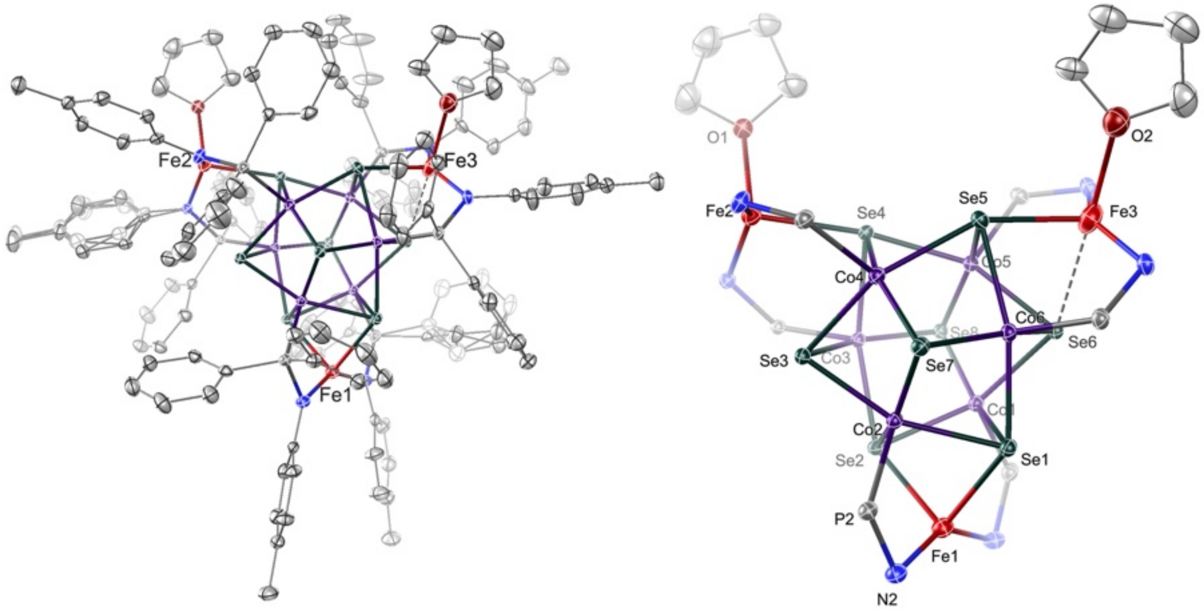

Figure S25. Single crystal X-ray structure of [**1**(THF)$_2$][PF$_6$] (thermal ellipsoids plotted at 50% probability). Disordered amidophosphine substituents are plotted over two positions. All hydrogen atoms and the [PF$_6$]$^-$ counterion have been omitted for clarity.

S6.2 Fe$_3$(MeCN)(OTf)Co$_6$Se$_8$L$_6$ (1(MeCN)(OTf))

Long, dark-red needles suitable for single crystal X-ray analysis were grown via vapor diffusion of diethyl ether into a concentrated acetonitrile solution at –35 °C over the course of 72 h. The compound crystallizes in the orthorhombic Pbca space group, and the asymmetric unit contains a single cluster, the triflate counterion, and two unbound diethyl ether molecules, one of which is disordered and was modeled over two positions. In the solid state, each of the three iron atoms exhibit a distinct coordination environment: one with a bound, rotationally disordered triflate anion (modeled over two positions), another with an acetonitrile molecule coordinated, and a third iron bound κ^4 to the [Co$_6$Se$_8$L$_6$]$^{6-}$ ligand framework.

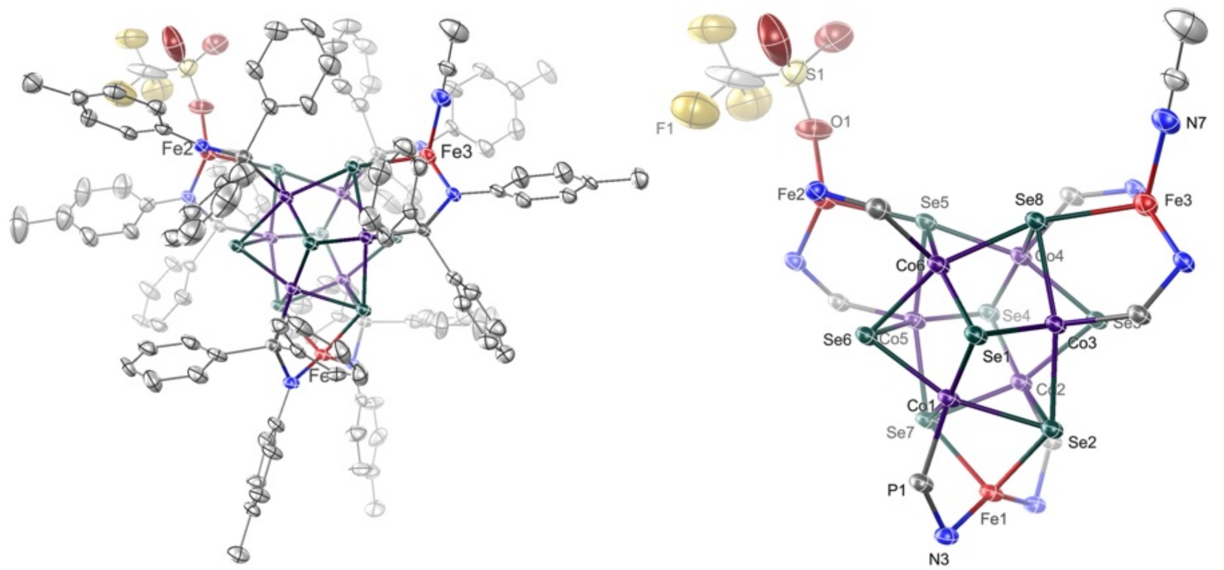

Figure S26. Single crystal X-ray structure of **1**(MeCN)(OTf) with thermal ellipsoids plotted at 50% probability. All hydrogen atoms, the disordered triflate fragment, and two co-crystallizing diethyl ether molecules have been omitted for clarity.

S6.3 [Fe_3(CN^{t}Bu)$_2$(THF)$Co_6Se_8L_6$][PF_6] (Disordered [1(CN^{t}Bu)$_2$(THF)][PF_6]) – sample 1

Dark red, prismatic crystals of [**1**(CN^{t}Bu)$_2$(THF)][PF_6] were grown via slow diffusion of a mixture of pentane and diethyl ether (~7:1) into a solution of [**1**(CN^{t}Bu)$_2$][PF_6] in THF at 25 °C. The compound crystallizes in the triclinic space group P$\overline{1}$, and the asymmetric unit contains a singular [**1**(CN^{t}Bu)$_2$(THF)]$^+$ cluster accompanied by a [PF_6]$^-$ counterion. Although the refinement is of high quality, the structure exhibits multiple instances of positional disorder, which necessitated the implementation of numerous restraints. The [PF_6]$^-$ counterion exhibits rotational disorder, and as a result it needed to be modeled over two positions. With a THF ligand bound to Fe3, the coordination of CN^{t}Bu to Fe1 is fluctional in the solid-state, causing Fe1 to oscillate between κ^3 and κ^4 binding. Further, both CN^{t}Bu ligands exhibit positional disorder and were modeled over two positions, one of which oscillates between a bound and unbound positions. Finally, two of the amidophosphines exhibit positional disorder and as a result were modeled over two positions. Taking all of these instances of disorder into consideration, the structure was modeled as a co-crystallizing mixture of [**1**(CN^{t}Bu)$_2$(THF)]$^+$ and [**1**(CN^{t}Bu)(THF)]$^+$ with 0.76 and 0.24 relative contributions, respectively.

Elemental analysis found (calc.) for crystalline [**1**(CN^{t}Bu)$_2$(THF)][PF_6] (Formula: $C_{128}H_{128}Co_6F_6Fe_3N_8OP_7Se_8$): Co 10.82 (10.79), Se 19.49 (19.27), P 6.63 (6.61).

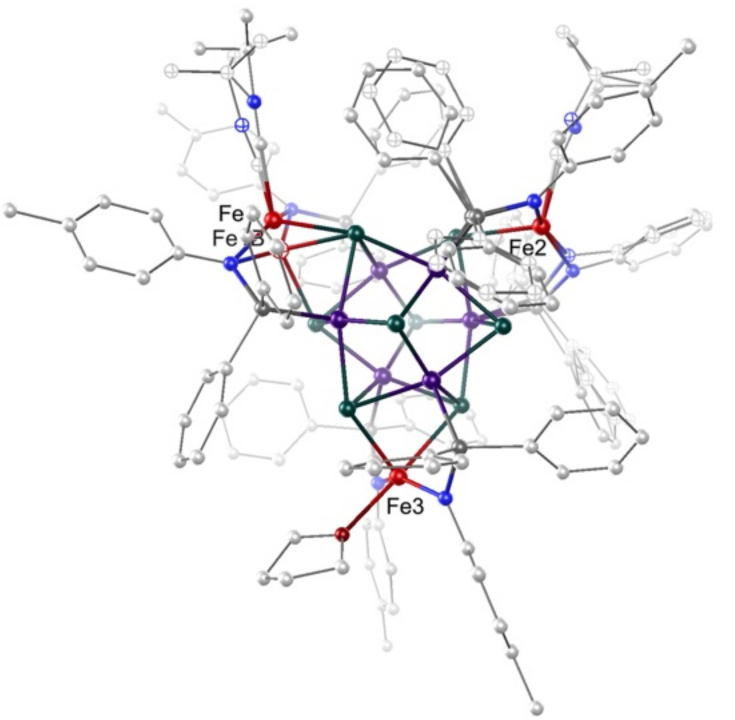

Figure S27. Solid-state structure of [**1**(CN^tBu)$_2$(THF)][PF_6] (sample 1) plotting both disordered components: (α,α,β)-[**1**(CN^tBu)$_2$(THF)]$^+$ and *trans*-(α,β)-[**1**(CN^tBu)(THF)]$^+$, which are present in a 76:24 ratio, respectively. Atoms associated only with the minor component, *trans*-(α,β)-[**1**(CN^tBu)(THF)]$^+$ are depicted as hollow spheres. All hydrogen atoms and the disordered [PF_6]$^-$ counterion have been omitted for clarity.

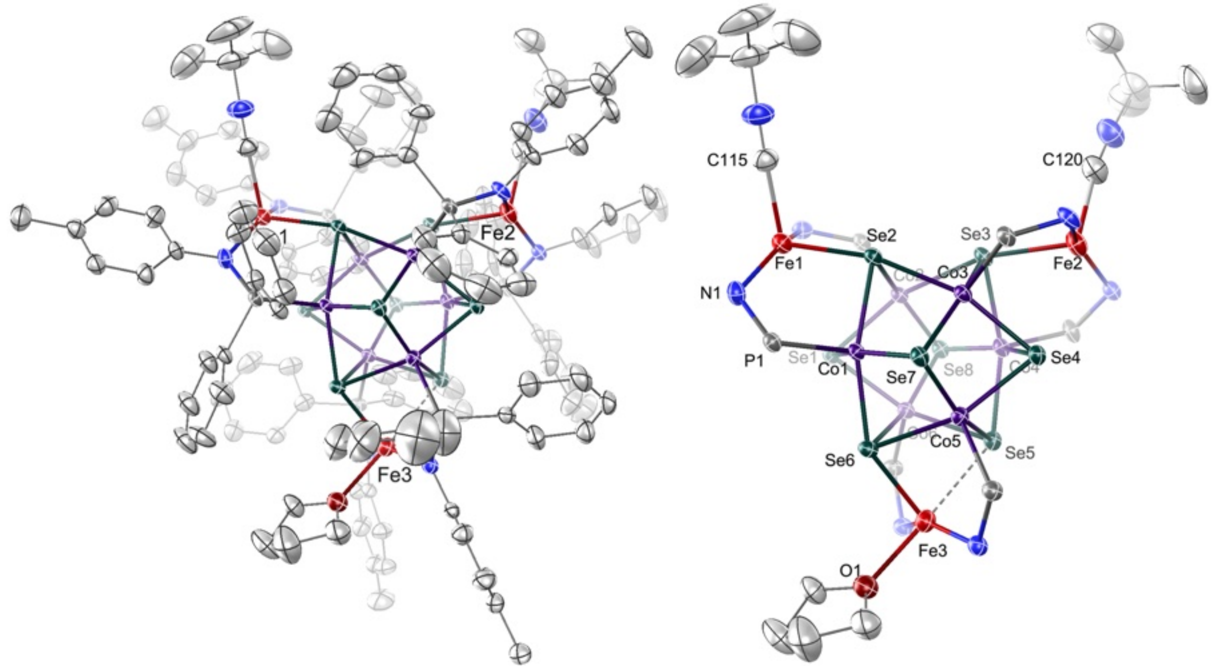

Figure S28. Single crystal X-ray structure of the major component (76% contribution), (α,α,β)-[**1**(CN^tBu)$_2$(THF)][PF_6], with thermal ellipsoids plotted at 50% probability. All hydrogen atoms and the rotationally disordered [PF_6]$^-$ counterion have been omitted for clarity.

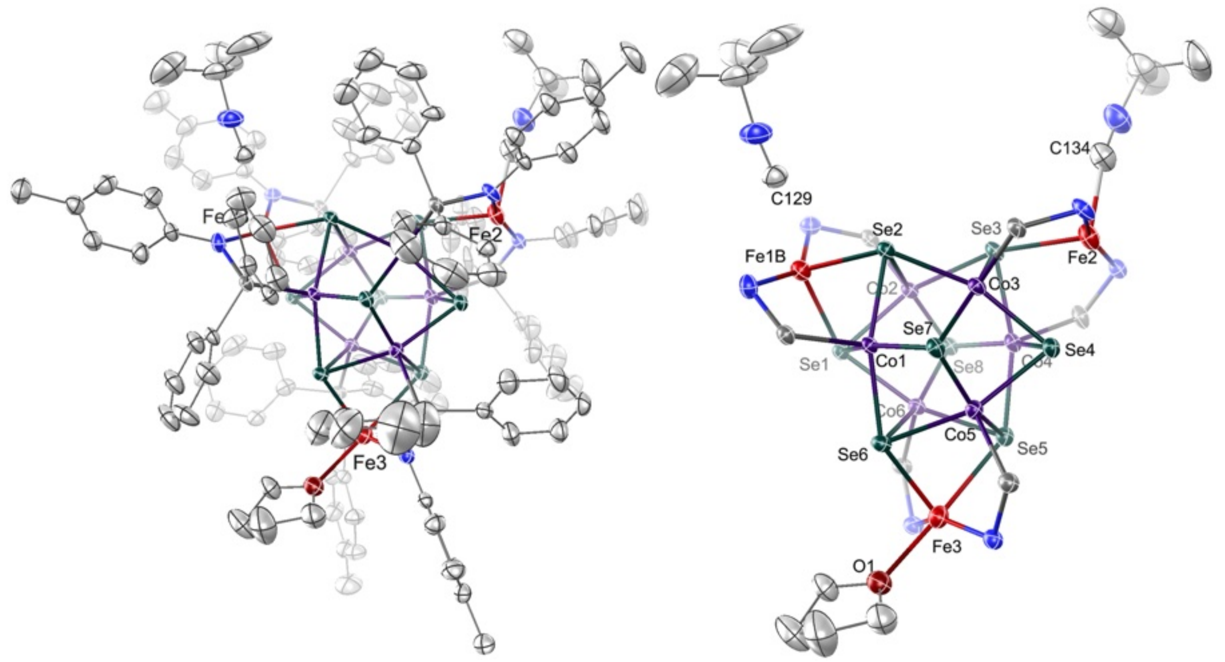

Figure S29. Single crystal X-ray structure of the minor component (24% contribution) *trans*-(α,β)-[**1**(CN^{t}Bu)(THF)]$^+$ including a dissociated CN^{t}Bu molecule proximal to Fe1B. Thermal ellipsoids are plotted at 50% probability. All hydrogen atoms and the rotationally disordered $[PF_6]^-$ counterion have been omitted for clarity.

S6.4 [Fe_3(CN^{t}Bu)$_2$(THF)$Co_6Se_8L_6$][PF_6] (Disordered [1(CN^{t}Bu)$_2$(THF)][PF_6]) – sample 2

A separate batch of [**1**(CN^{t}Bu)$_2$(THF)][PF_6], isolated independently of sample 1, was crystallized via slow diffusion of a 1:7 mixture of diethyl ether and *n*-pentane into a solution of [**1**(CN^{t}Bu)$_2$][PF_6] in THF at 25 °C to yield a crop of dark red, prismatic crystals. The compound crystallizes in the triclinic space group $P\bar{1}$, with the same unit cell parameters obtained for sample 1. The asymmetric unit contains a singular [**1**(CN^{t}Bu)$_2$(THF)]$^+$ cluster accompanied by a $[PF_6]^-$ counterion. Refinement is of high quality and the structure exhibits positional disorder similar to that of sample 1, although in this case the major and minor components are present in 85% and 15% contributions, respectively.

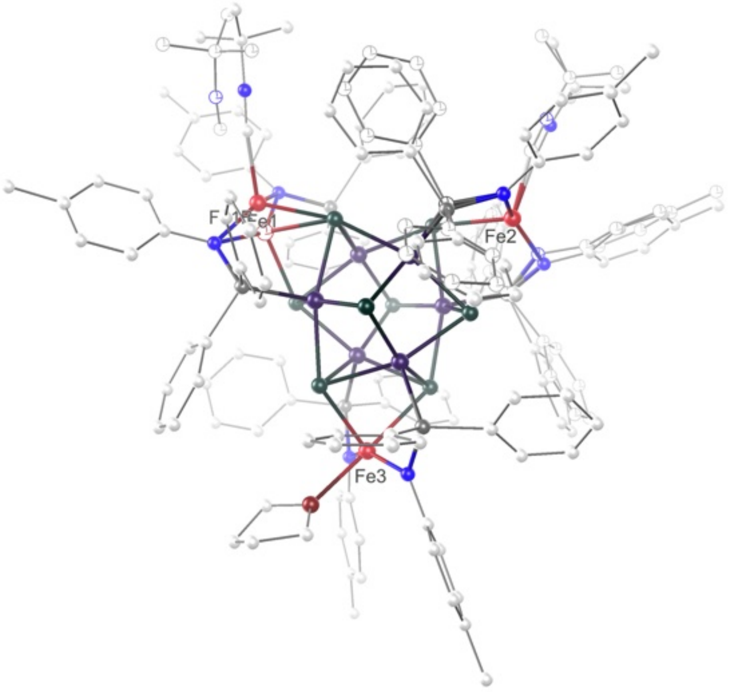

Figure S30. Solid-state structure of [**1**(CN^{t}Bu)$_2$(THF)][PF$_6$] (sample 2) plotting both disordered components: (α,α,β)-[**1**(CN^{t}Bu)$_2$(THF)]$^+$ and *trans*-(α,β)-[**1**(CN^{t}Bu)(THF)]$^+$, which are present in a 85:15 ratio. Atoms associated only with the minor component, *trans*-(α,β)-[**1**(CN^{t}Bu)(THF)]$^+$ are depicted as hollow spheres. All hydrogen atoms and the disordered [PF$_6$]$^-$ counterion have been omitted for clarity.

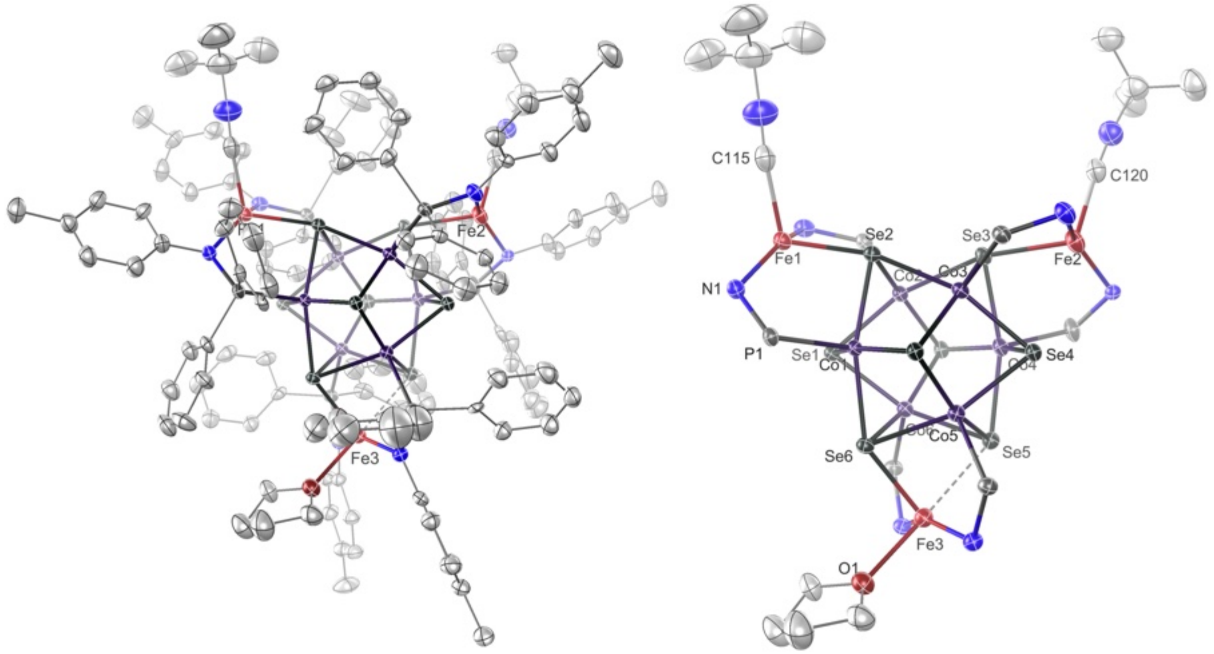

Figure S31. Single crystal X-ray structure of the major component, (α,α,β)-[**1**(CN^{t}Bu)$_2$(THF)][PF$_6$] (85% contribution), with thermal ellipsoids plotted at 50% probability. All hydrogen atoms and the rotationally disordered [PF$_6$]$^-$ counterion have been omitted for clarity.

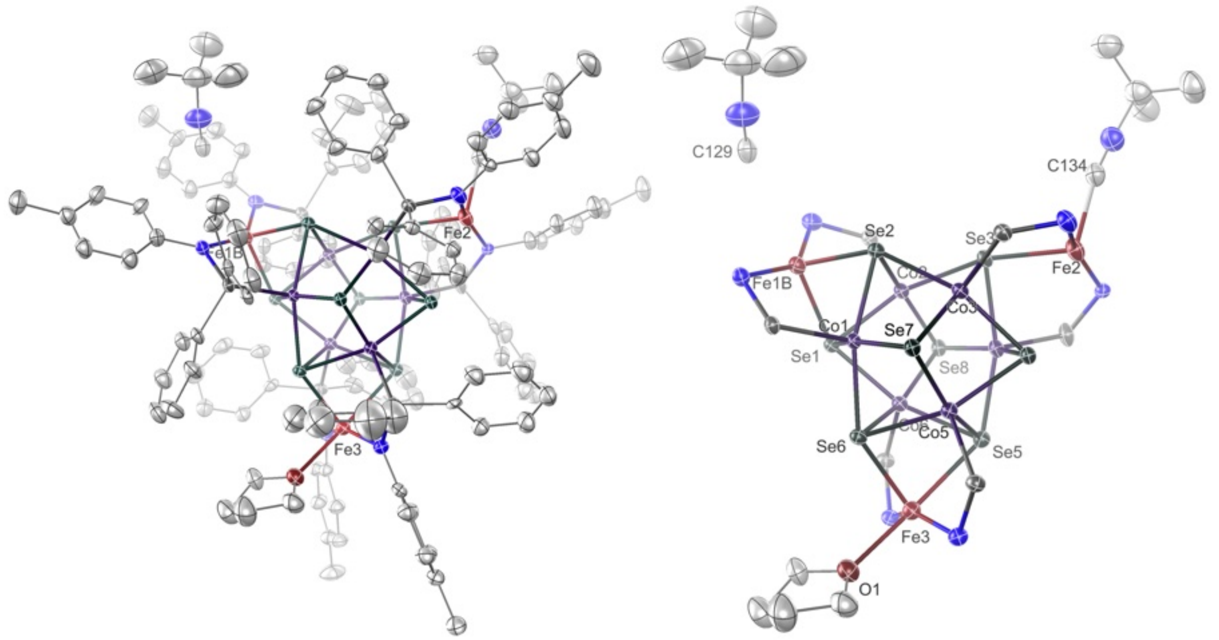

Figure S32. Single crystal X-ray structure of the minor component, *trans*-(α,β)-[**1**(CN^{t}Bu)(THF)]$^+$ (15% contribution), including a dissociated CN^{t}Bu molecule proximal to Fe1B. Thermal ellipsoids are plotted at 50% probability. All hydrogen atoms and the [PF$_6$]$^-$ counterion have been omitted for clarity.

S6.5 [Fe$_3$(CN^{t}Bu)$_2$(THF)Co$_6$Se$_8$L$_6$][PF$_6$] (Disordered [1(CN^{t}Bu)$_2$(THF)][PF$_6$]) – sample 3

Dark red, prismatic crystals of [**1**(CN^{t}Bu)$_2$(THF)][PF$_6$] were grown via slow diffusion of a 1:7 mixture of diethyl ether and pentane into a solution of [**1**(CN^{t}Bu)$_2$][PF$_6$] in THF at 25 °C. Aluminum foil was placed on the glass doors of the diffractometer to limit the exposure of ambient light to the crystal. Complete data sets were recorded on the same crystal with increasing temperature at 100 K, 150 K, 200 K, and 250 K, before the instrument was cooled back down to 100 K for a final measurement. The compound crystallizes in the triclinic space group P$\overline{1}$, and the asymmetric unit contains a singular disordered [**1**(CN^{t}Bu)$_2$(THF)]$^+$ cluster accompanied by a [PF$_6$]$^-$ counterion. The structure exhibits the same unit cell as the previously recorded samples of disordered [**1**(CN^{t}Bu)$_2$(THF)][PF$_6$], but the disorder observed here is slightly different. Most notably, we do not observe clear dissociation of isocyanide at Fe1B in the minor component. Instead, the optimal refinement indicates CN^{t}Bu remains loosely bound to this site.

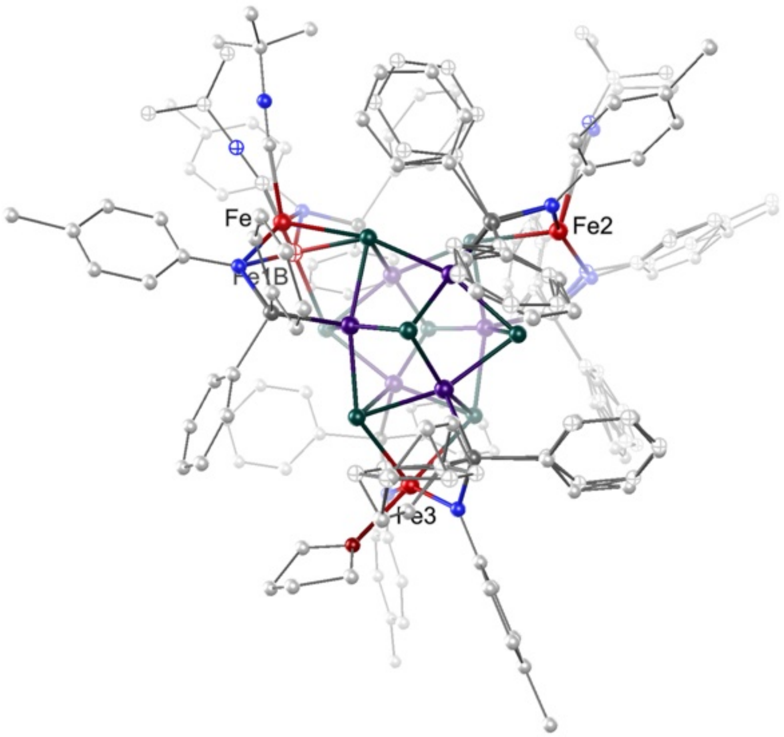

Figure S33. Single crystal X-ray structure of [**1**(CN^{t}Bu)$_2$(THF)][PF_6] (sample 3) for the first measurement collected at 100 K. Atoms corresponding to minor component are depicted as hollow spheres. All hydrogen atoms and the rotationally disordered $[PF_6]^-$ counterion have been omitted for clarity.

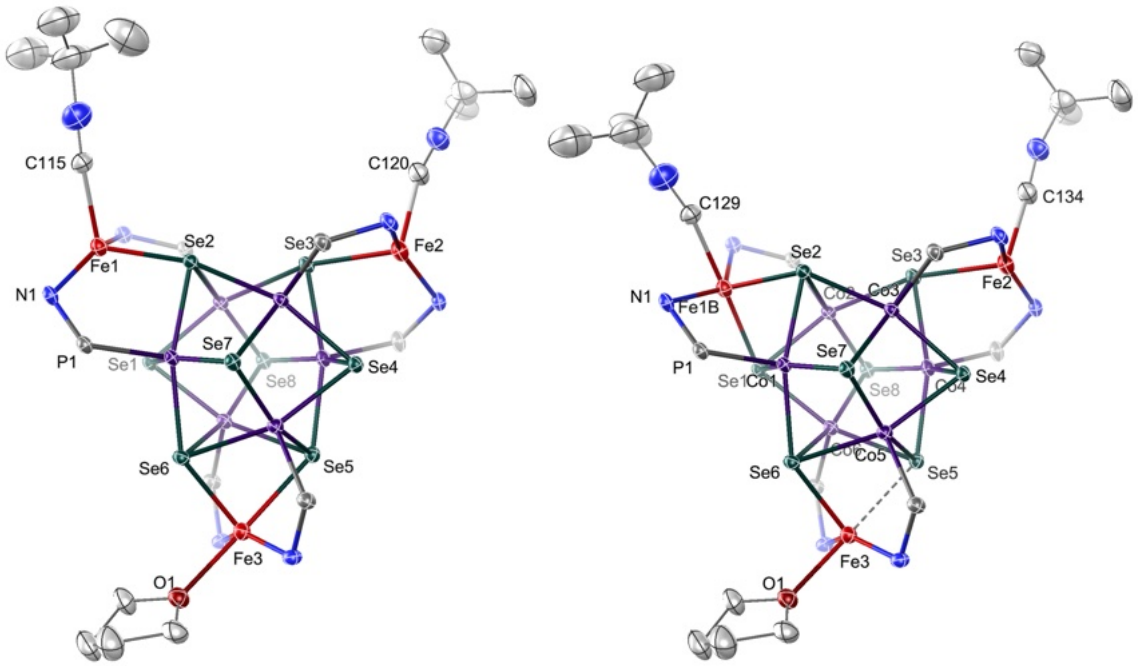

Figure S34. Single crystal X-ray structures of the major (left) and minor (right) of [**1**(CN^{t}Bu)$_2$(THF)][PF_6] (variable temperature) for the first measurement collected at 100 K. Thermal ellipsoids plotted at 50% probability. All hydrogen atoms and the disordered $[PF_6]^-$ counterion have been omitted for clarity.

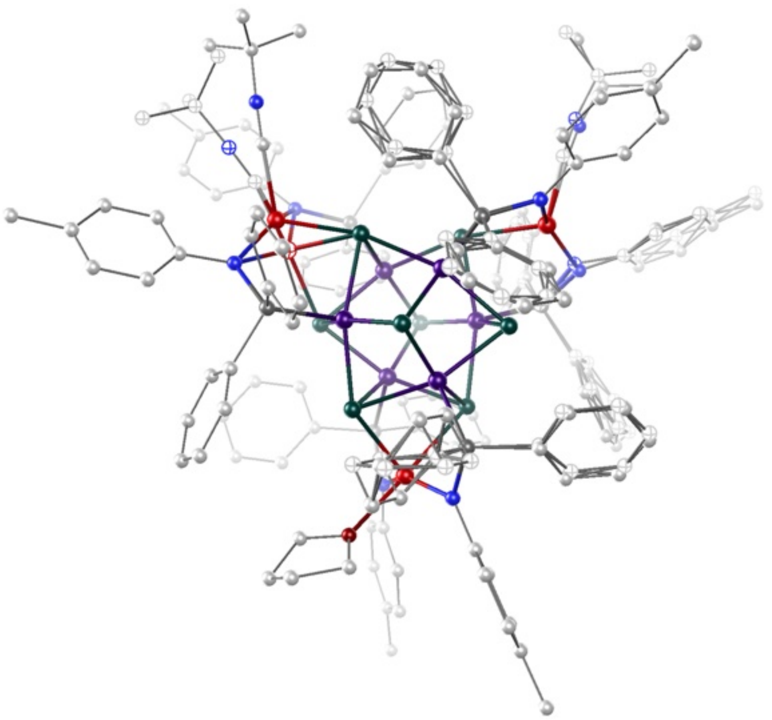

Figure S35. Single crystal X-ray structure of [**1**(CN^{t}Bu)$_2$(THF)][PF$_6$] (sample 3) for the measurement collected at 150 K. Atoms corresponding to minor component are depicted as hollow spheres. All hydrogen atoms and the rotationally disordered [PF$_6$]$^-$ counterion have been omitted for clarity.

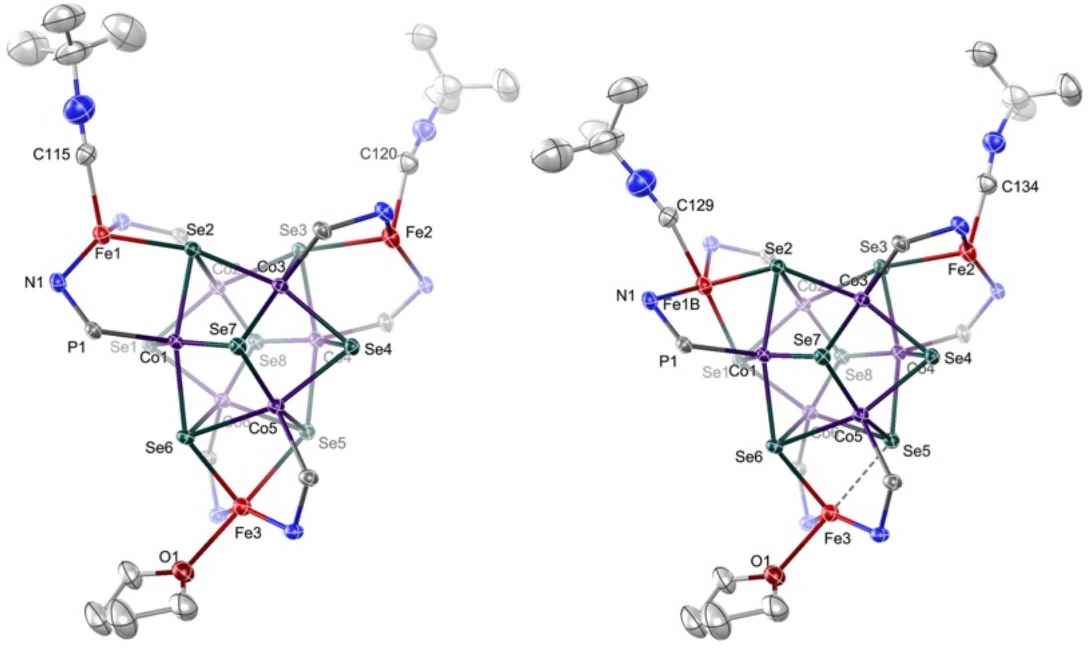

Figure S36. Single crystal X-ray structures of the major (left) and minor (right) of [**1**(CN^{t}Bu)$_2$(THF)][PF$_6$] (sample 3) for the measurement collected at 150 K. Thermal ellipsoids plotted at 50% probability. All hydrogen atoms and the rotationally disordered [PF$_6$]$^-$ counterion have been omitted for clarity.

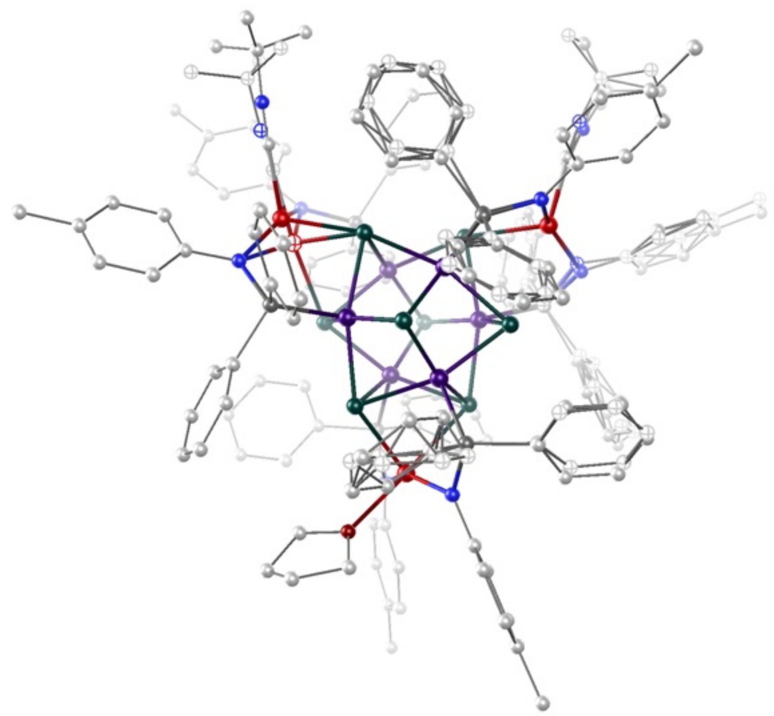

Figure S37. Single crystal X-ray structure of [**1**(CN^{t}Bu)$_2$(THF)][PF_6] (sample 3) for the measurement collected at 200 K. Atoms corresponding to minor component are depicted as hollow spheres. All hydrogen atoms and the rotationally disordered [PF_6]$^-$ counterion have been omitted for clarity.

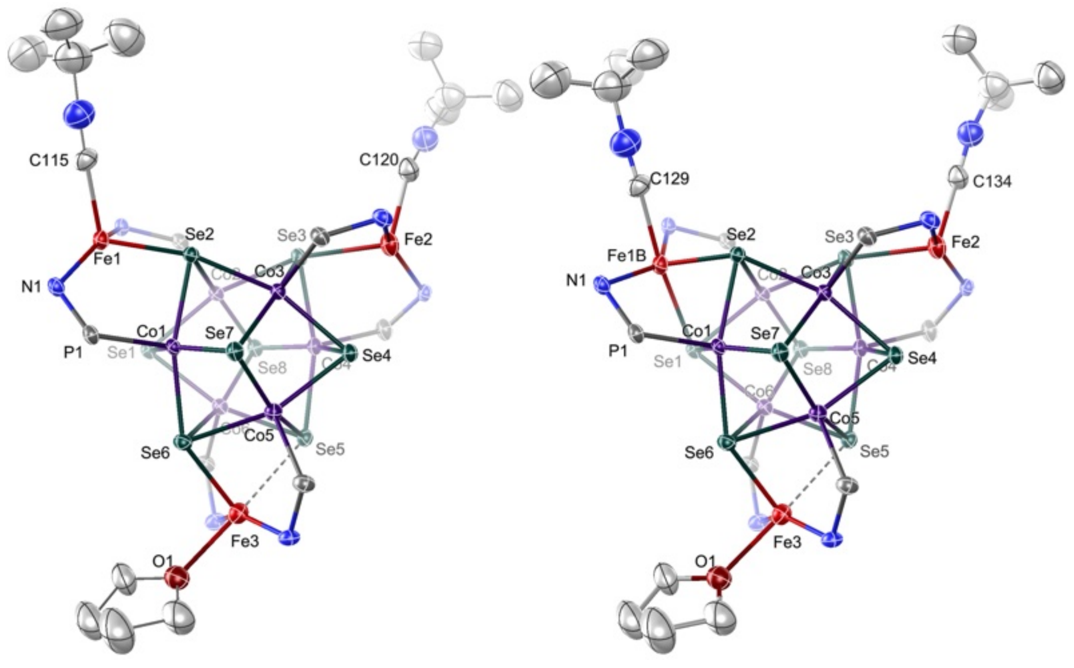

Figure S38. Single crystal X-ray structures of the major (left) and minor (right) of [**1**(CN^{t}Bu)$_2$(THF)][PF_6] (sample 3) for the measurement collected at 200 K. Thermal ellipsoids plotted at 50% probability. All hydrogen atoms and the disordered [PF_6]$^-$ counterion have been omitted for clarity.

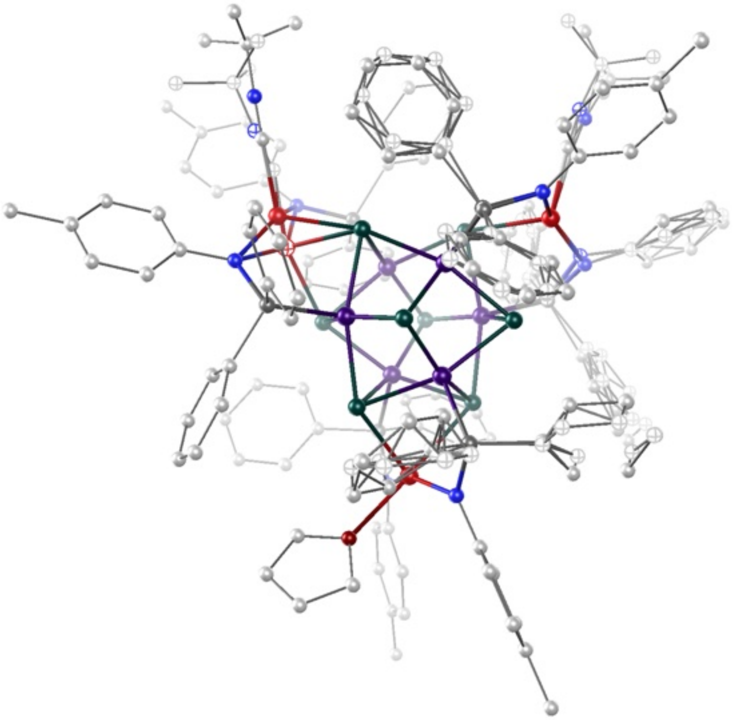

Figure S39. Single crystal X-ray structure of [**1**(CN^{t}Bu)$_2$(THF)][PF$_6$] (sample 3) for the measurement collected at 250 K. Atoms corresponding to minor component are depicted as hollow spheres. All hydrogen atoms and the disordered [PF$_6$]$^-$ counterion have been omitted for clarity.

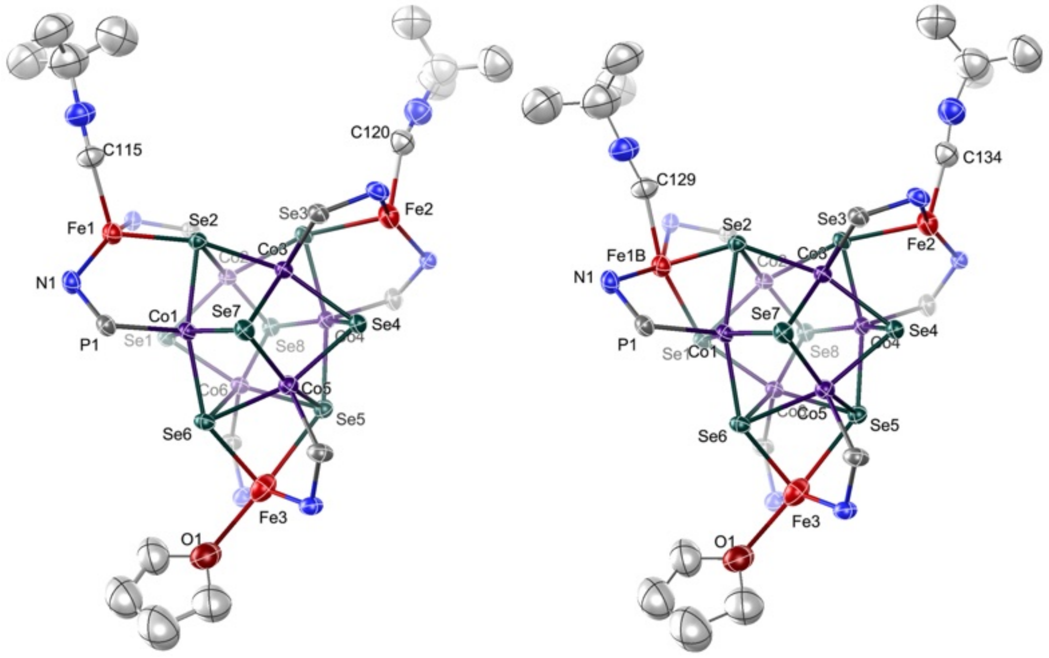

Figure S40. Single crystal X-ray structures of the major (left) and minor (right) of [**1**(CN^{t}Bu)$_2$(THF)][PF$_6$] (sample 3) for the measurement collected at 250 K. Thermal ellipsoids plotted at 50% probability. All hydrogen atoms and the disordered [PF$_6$]$^-$ counterion have been omitted for clarity.

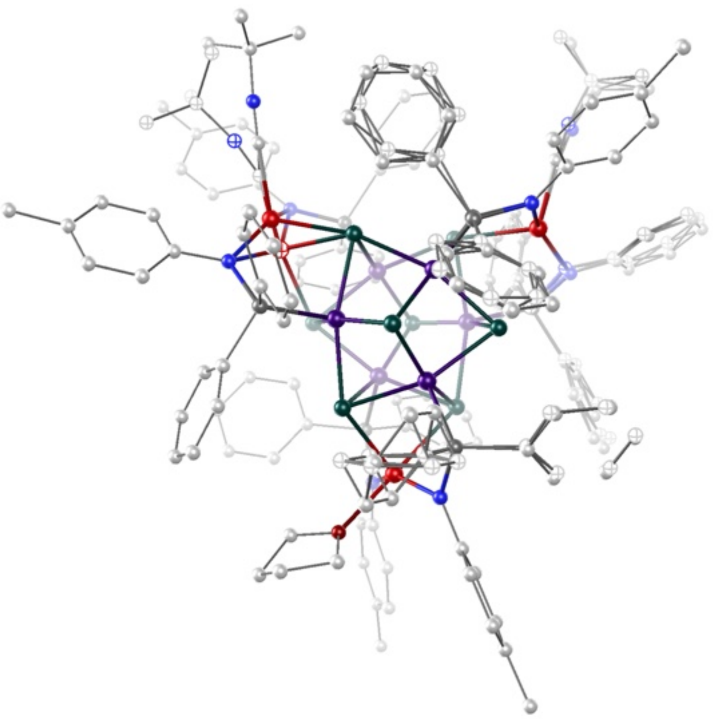

Figure S41. Single crystal X-ray structure of [**1**(CN^tBu)$_2$(THF)][PF_6] (sample 3) for the final measurement collected at 100 K. Atoms corresponding to minor component are depicted as hollow spheres. All hydrogen atoms and the disordered $[PF_6]^-$ counterion have been omitted for clarity.

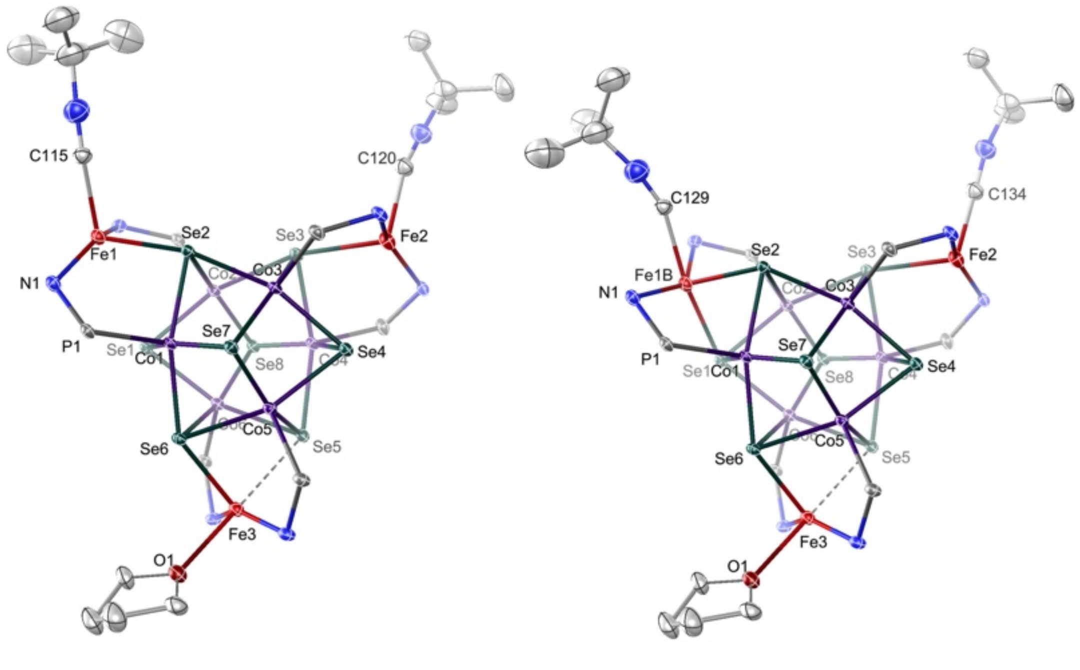

Figure S42. Single crystal X-ray structures of the major (left) and minor (right) of [**1**(CN^tBu)$_2$(THF)][PF_6] (sample 3) for the final measurement collected at 100 K. Thermal ellipsoids plotted at 50% probability. All hydrogen atoms and the rotationally disordered $[PF_6]^-$ counterion have been omitted for clarity.

S6.6 [$Fe_3(CN^tBu)_3Co_6Se_8L_6$][$PF_6$] ([**1**($CN^tBu$)$_3$][$PF_6$])

Dark red, prismatic crystals of [**1**(CN^{t}Bu)$_3$][PF_6] were grown via slow diffusion of pentane into a solution of [**1**(CN^{t}Bu)$_2$][PF_6] and 5 equiv. of CN^{t}Bu in DCM at 25 °C. The compound crystallizes in the triclinic space group P$\bar{1}$, and the asymmetric unit contains a singular [**1**(CN^{t}Bu)$_3$]$^+$ cluster accompanied by a [PF_6]$^-$ counterion. The [PF_6]$^-$ counterion exhibits rotational disorder while one phenyl group on an amidophosphine exhibits positional disorder. As such, both components were modeled over two positions. The weighting scheme was adjusted manually to limit rest electron density and to move the goodness of fit parameter close to 1.

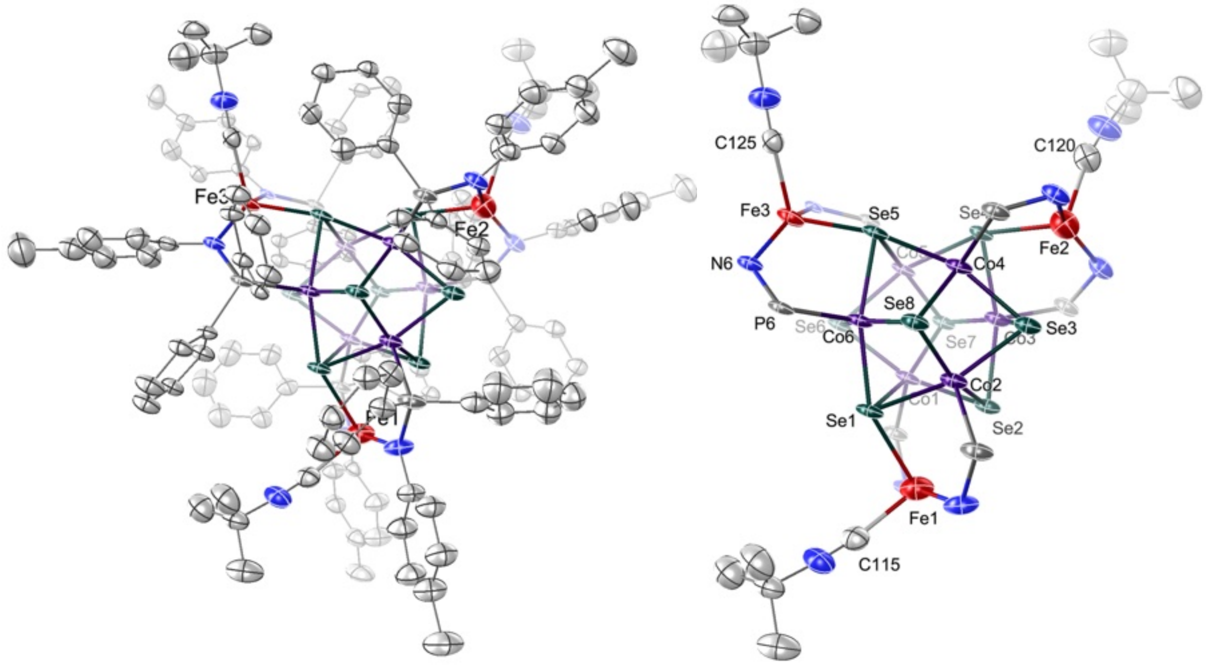

Figure S43. Single crystal X-ray structure of [**1**(CN^{t}Bu)$_3$][PF_6] with thermal ellipsoids plotted at 50% probability. All hydrogen atoms, the disordered phenyl substituent, and the [PF_6]$^-$ counterion have been omitted for clarity.

S6.7 X-ray Tables

Table S3. Select interatomic distances (Å) for repeat measurements of disordered crystals of [**1**(CN^{t}Bu)$_2$(THF)][PF$_6$].

	**(α) Fe-THF edge**			**(α) Fe(CN^{t}Bu) disordered**			**(β) Fe(CN^{t}Bu)**		
**Structure**	Fe—O	Fe—Se		Fe—C	Fe—Se		Fe—C	Fe—Se	
#1; 0.76	2.237(5)	2.500(2)	2.699(3)	2.13(1)	2.498(7)	3.258(3)	2.11(3)	2.541(7)	3.042(3)
#1; 0.24				**2.41(3)**	2.471(8)	**2.403(5)**	2.1(1)		
#2; 0.85	2.243(8)	2.436(2)	2.648(3)	2.12(2)	2.512(7)	3.248(4)	2.11(3)	2.537(7)	3.056(4)
#2; 0.15				**3.42(8)**	2.46(2)	**2.37(1)**	2.11(4)		
#3; 0.95 (100 K-*i*)	2.242(5)	2.428(2)	2.623(4)	2.094(9)	2.525(9)	3.251(4)	2.10(4)	2.53(1)	3.058(4)
#3; 0.5 (100 K-*i*)				2.10(7)	2.43(2)	**2.34(2)**	2.10(7)		
#3; 0.95 (150 K)	2.260(7)	2.430(3)	2.631(7)	2.09(1)	2.53(2)	3.252(7)	2.11(4)	2.53(2)	3.055(8)
#3; 0.05 (150 K)				2.09(4)	2.43(2)	2.33(2)	2.11(7)		
#3; 0.90 (200 K)	**2.30(1)**	2.442(3)	2.662(5)	2.09(2)	2.526(9)	3.258(5)	2.10(4)	2.53(1)	3.051(6)
#3; 0.10 (200 K)				2.1(1)	2.39(2)	2.47(2)	2.10(5)		
#3; 0.84 (250 K)	**2.37(1)**	2.445(4)	2.709(7)	2.11(2)	2.51(2)	3.272(8)	2.11(5)	2.53(2)	3.038(8)
#3; 0.16 (250 K)				2.11(5)	2.49(2)	**2.37(1)**	2.10(7)		
#3; 0.95 (100 K-*f*)	2.240(5)	2.426(2)	2.620(4)	2.11(1)	2.522(9)	3.251(4)	2.11(3)	2.53(1)	3.051(4)
#3; 0.95 (100 K-*f*)				2.11(6)	2.45(2)	2.34(2)	2.11(5)		

Table S4. Select interatomic distances for **1**(MeCN)(OTf), [**1**(CN^{t}Bu)$_2$][PF$_6$], and [**1**(CN^{t}Bu)$_3$][PF$_6$]. Bonding parameters involving Fe centers as well as τ_4 values are listed for Fe1, Fe2, and Fe3 from top to bottom.

**Compound**	**Interatomic Distance (Å)**						
	Fe…Se	Fe…Co	Co–P	*trans*-Co…Co	Co–Se	Fe–N^{Tol}	τ_4
**1**(MeCN)(OTf)	2.412(1)<br>2.414(1)<br>2.495(2)<br>3.483(2)<br>2.574(1)<br>3.194(2)	2.766(1)<br>2.785(2)<br>3.426(2)<br>3.652(2)<br>3.429(2)<br>3.453(2)	2.169(2) –<br>2.188(2)	4.151(1)<br>4.082(1)<br>4.132(2)	2.315(1) –<br>2.435(1)<br>Avg. 2.35<br>∑ 56.34	1.945(6)<br>1.956(6)<br>1.982(6)<br>1.980(6)<br>1.955(6)<br>1.980(6)<br>Avg. 1.97	0.79<br>0.80<br>0.82
[**1**(CN^{t}Bu)$_2$][PF$_6$]	2.488(7)<br>3.288(6)<br>2.546(7)<br>3.254(8)<br>2.397(6)<br>2.433(5)	3.372(8)<br>3.458(6)<br>3.321(5)<br>3.551(9)<br>2.661(8)<br>2.853(7)	2.164(5) –<br>2.178(5)	4.044(9)<br>4.11(1)<br>4.18(1)	2.304(3) –<br>2.424(3)<br>Avg. 2.35<br>∑ 56.43	1.996(12)<br>1.972(13)<br>2.003(12)<br>1.996(12)<br>1.960(12)<br>1.914(12)<br>Avg. 2.01	0.80<br>0.81<br>0.77
[**1**(CN^{t}Bu)$_3$][PF$_6$]	2.531(3)<br>2.969(5)<br>2.480(3)<br>2.978(5)<br>2.542(3)<br>3.32(6)	3.159(6)<br>3.281(6)<br>3.303(6)<br>3.115(9)<br>3.45(4)<br>3.520(4)	2.160(3) –<br>2.176(4)	4.111(8)<br>4.088(7)<br>4.05(1)	2.311(2) –<br>2.395(2)<br>Avg. 2.35<br>∑ 56.33	1.998(7)<br>2.03(1)<br>2.018(8)<br>2.006(7)<br>2.002(7)<br>1.988(9)<br>Avg. 2.01	0.71<br>0.71<br>0.75

Table S5. Select interatomic distances for [**1**(THF)$_2$][PF$_6$] and [**1**(CN^{t}Bu)$_2$(THF)][PF$_6$]/trans-(α,β)-[**1**(CN^{t}Bu)(THF)][PF$_6$]. Bonding parameters involving Fe centers as well as τ_4 values are listed for Fe1, Fe2, and Fe3 from top to bottom.

Compound	Interatomic Distance (Å)						
	Fe…Se	Fe…Co	Co–P	*trans*-Co…Co	Co–Se	Fe–N^{Tol}	τ_4
[**1**(THF)$_2$][PF$_6$]	2.4410(7)<br>2.4566(7)<br>2.5172(7)<br>3.388(8)<br>2.4441(8)<br>2.7294(8)	2.880(2)<br>2.755(3)<br>3.594(4)<br>3.395(2)<br>2.997(1)<br>3.049(4)	2.168(1) – 2.186(1)	4.130(3)<br>4.204(4)<br>4.0900(6)	2.3027(6) – 2.4274(6)<br>Avg. 2.36<br>∑ 56.55	1.970(3)<br>1.955(3)<br>1.982(3)<br>1.980(3)<br>1.964(3)<br>1.949(3)<br>Avg. 1.97	0.75<br>0.81<br>0.75<br>0.58[a]
[**1**(CN^{t}Bu)$_2$(THF)]$^+$/*trans*-(α,β)-[**1**(CN^{t}Bu)(THF)]$^+$ (sample 1)	2.403(4)[b]<br>2.471(4)[b]<br>2.498(1)<br>3.258(3)<br>2.541(1)<br>3.042(4)<br>2.450(1)<br>2.699(1)	2.853(4)[b]<br>2.888(4)[b]<br>3.370(4)<br>3.427(6)<br>3.216(6)<br>3.411(4)<br>2.981(1)<br>3.031(3)	2.162(2) – 2.194(9)	4.101(7)<br>4.113(8)<br>4.113(6)	2.3146(9) – 2.3928(9)<br>Avg. 2.35<br>∑ 56.45	1.966(6)[b]<br>2.023(5)[b]<br>2.010(5)<br>1.965(4)<br>2.011(5)<br>2.054(8)<br>2.002(4)<br>2.020(5)<br>Avg. 1.99 Å	0.76<br>0.76[b]<br>0.73<br>0.74<br>0.46[a]
[**1**(CN^{t}Bu)$_2$(THF)]$^+$/*trans*-(α,β)-[**1**(CN^{t}Bu)(THF)]$^+$ (sample 2)	2.37(1)[b]<br>2.46(1)[b]<br>2.512(7)<br>3.248(4)<br>2.537(7)<br>3.056(4)<br>2.436(3)<br>2.648(3)	2.84(1)[b]<br>2.86(1)[b]<br>3.374(5)<br>3.442(6)<br>3.216(6)<br>3.430(5)<br>2.963(6)<br>3.005(4)	2.15(2) – 2.18(1)	4.077(8)<br>4.117(8)<br>4.122(6)	2.309(2) – 2.396(2)<br>Avg. 2.35<br>∑ 56.43	1.99(1)[b]<br>2.04(1)[b]<br>2.015(6)<br>1.969(6)<br>2.033(7)<br>2.11(1)<br>2.012(6)<br>2.030(6)<br>Avg. 2.02 Å	0.75<br>0.78[b]<br>0.73<br>0.74<br>0.24[a]

[a]Calculated τ_5 value for Fe3 taking the weakly associated Se atom into account for the κ^4-bound Fe sites.

[b]Parameters corresponding to the minor component, the κ^4-bound Fe1b of trans-(α,β)-[**1**(CN^{t}Bu)(THF)][PF$_6$].

Table S6. Crystallographic data for [**1**($THF)_2$][PF_6], **1**(MeCN)(OTf), and [**1**($CN^tBu)_3$][PF_6].

Compound	[**1**($THF)_2$][PF_6]	**1**(MeCN)(OTf)	[**1**($CN^tBu)_3$][PF_6]
**Empirical formula**	$C_{122}H_{118}Co_6F_6Fe_3N_6O_2P_7Se_8$	$C_{123.32}H_{120.79}Co_6F_3Fe_3N_7O_{4.58}P_6SSe_8$	$C_{129}H_{129}Co_6F_6Fe_3N_9P_7Se_8$
**CCDC Number**	2097608	2090142	2320345
**Formula weight**	3183.82	3201.83	3289.00
**Temperature (K)**	100(2)	100(2)	100(2)
**Wavelength (Å)**	0.71073	0.71073	0.71073
**Crystal system**	Monoclinic	Orthorhombic	Triclinic
**Space group**	P 21/c	Pbca	$P\bar{1}$
**a (Å)**	22.4240(12)	19.4968(13)	18.6251(14)
**b (Å)**	19.6456(9)	29.070(2)	19.3562(14)
**c (Å)**	28.5568(15)	43.890(3)	20.5647(14)
**α (°)**	90	90	89.231(4)
**β (°)**	100.843(3)	90	86.037(4)
**γ (°)**	90	90	66.926(4)
**Volume (Å^3)**	12355.6(11)	24875(3)	6803.6(9)
**Z**	4	8	2
**ρ^{calc} (g cm^{-1})**	1.712	1.710	1.605
**Absorption coefficient (mm^{-1})**	3.634	3.613	3.302
**F(000)**	6316	12731	3274
**Crystal size (mm^3)**	0.770 x 0.080 x 0.070	0.320 x 0.050 x 0.040	0.150 x 0.090 x 0.020
**Theta range for data collection (°)**	1.389 to 28.333	1.340 to 28.343	0.993 to 25.027
**Index ranges**	−29≤h≤29, −26≤k≤26, −38≤l≤38	−26≤h≤26, −38≤k≤38, −58≤l≤58	−22≤h≤22, −23≤k≤23, −24≤l≤24
**Reflections collected**	60651	59854	47405
**Independent reflections**	30778 [R(int) = 0.0465]	30874 [R(int) = 0.1428]	23921 [R(int) = 0.1505]
**Completeness to theta = 25.000°**	100.0%	99.9%	99.6%
**Data / restraints / parameters**	30778 / 45 / 1489	30874 / 130 / 1561	23921 / 2243 / 1617
**Goodness-of-fit on F^2**	1.000	1.005	0.991
**Final R indices [I>2sigma(I)]**	R1 = 0.0373, wR2 = 0.0736	R1 = 0.0654, wR2 = 0.1222	R1 = 0.0843, wR2 = 0.1489
**R indices (all data)**	R1 = 0.0802, wR2 = 0.0882	R1 = 0.1963, wR2 = 0.1625	R1 = 0.2066, wR2 = 0.1765
**Largest diff. peak and hole (e$^-$Å^{-3})**	2.190 and −2.013	1.212 and −1.055	2.466 and −1.426

Table S7. Crystallographic data for [**1**(CN^{t}Bu)$_2$(THF)][PF$_6$] (1), [**1**(CN^{t}Bu)$_2$(THF)][PF$_6$] (2), and [**1**(CN^{t}Bu)$_2$(THF)][PF$_6$] (3) at 100K (initial).

**Compound**	[**1**(CN^{t}Bu)$_2$(THF)][PF$_6$] (1)	[**1**(CN^{t}Bu)$_2$(THF)][PF$_6$] (2)	[**1**(CN^{t}Bu)$_2$(THF)][PF$_6$] (3) 100 K-*i*
**Empirical formula**	$C_{128}H_{128}Co_6F_6Fe_3N_8OP_7Se_8$	$C_{128}H_{128}Co_6F_6Fe_3N_8OP_7Se_8$	$C_{128}H_{128}Co_6F_6Fe_3N_8OP_7Se_8$
**CCDC Number**	2320591	2097609	2320349
**Formula weight**	3277.98	3277.98	3277.98
**Temperature (K)**	100(2)	100(2)	100(2)
**Wavelength (Å)**	0.71073	0.71073	0.71073
**Crystal system**	Triclinic	Triclinic	Triclinic
**Space group**	$P\bar{1}$	$P\bar{1}$	$P\bar{1}$
**a (Å)**	19.480(2)	19.480(2)	19.5061(15)
**b (Å)**	19.724(2)	19.724(2)	19.6529(15)
**c (Å)**	20.296(2)	20.296(2)	20.2439(15)
**α (°)**	89.548(3)	89.548(3)	89.931(4)
**β (°)**	71.860(3)	71.860(3)	71.512(4)
**γ (°)**	72.506(3)	72.506(3)	73.044(4)
**Volume (Å^3)**	7036.4(13)	7036.4(13)	7004.3(9)
**Z**	2	2	2
**ρ^{calc} (g cm^{-1})**	1.547	1.547	1.554
**Absorption coefficient (mm^{-1})**	3.193	3.193	3.207
**F(000)**	3262	3262	3262
**Crystal size (mm^3)**	0.600 x 0.420 x 0.090	0.600 x 0.420 x 0.090	0.480 x 0.210 x 0.040
**Theta range for data collection (°)**	1.445 to 28.339	1.293 to 28.282	1.157 to 27.484
**Index ranges**	$-26\leq h\leq 25$, $-26\leq k\leq 26$, $-27\leq l\leq 27$	$-25\leq h\leq 25$, $-26\leq k\leq 26$, $-27\leq l\leq 27$	$-25\leq h\leq 25$, $-25\leq k\leq 25$, $-26\leq l\leq 26$
**Reflections collected**	69531	228082	239791
**Independent reflections**	34926 [R(int) = 0.0472]	60498 [R(int) = 0.0579]	31916 [R(int) = 0.0594]
**Completeness to theta = 25.000°**	99.8%	96.0%	99.8%
**Data / restraints / parameters**	34926 / 441 / 1671	60498 / 2829 / 1631	31916 / 3201 / 1445
**Goodness-of-fit on F^2**	1.019	1.063	1.033
**Final R indices [I>2sigma(I)]**	R1 = 0.0605, wR2 = 0.1452	R1 = 0.0836, wR2 = 0.1546	R1 = 0.0580, wR2 = 0.1106
**R indices (all data)**	R1 = 0.0964, wR2 = 0.1621	R1 = 0.1587, wR2 = 0.1887	R1 = 0.0852, wR2 = 0.1259
**Largest diff. peak and hole (e$^-$Å^{-3})**	3.033 and −1.036	2.345 and −1.265	2.312 and −1.308

Table S8. Crystallographic data for [**1**(CN^tBu)$_2$(THF)][PF_6] (3) at 150, 200, and 250 K.

Compound	[**1**(CN^tBu)$_2$(THF)][PF_6] (3) 150 K	[**1**(CN^tBu)$_2$(THF)][PF_6] (3) 200K	[**1**(CN^tBu)$_2$(THF)][PF_6] (3) 250K
**Empirical formula**	$C_{128}H_{128}Co_6F_6Fe_3N_8OP_7Se_8$	$C_{128}H_{128}Co_6F_6Fe_3N_8OP_7Se_8$	$C_{128}H_{128}Co_6F_6Fe_3N_8OP_7Se_8$
**CCDC Number**	2320347	2320346	2320348
**Formula weight**	3277.98	3277.98	3277.98
**Temperature (K)**	150(2)	200(2)	250(2)
**Wavelength (Å)**	0.71073	0.71073	0.71073
**Crystal system**	Triclinic	Triclinic	Triclinic
**Space group**	$P\bar{1}$	$P\bar{1}$	$P\bar{1}$
**a (Å)**	19.584(2)	19.6947(16)	19.840(2)
**b (Å)**	19.675(2)	19.7617(16)	19.804(2)
**c (Å)**	20.326(2)	20.3585(16)	20.400(2)
**α (°)**	89.897(7)	89.974(5)	90.076(7)
**β (°)**	71.677(7)	71.901(4)	72.287(7)
**γ (°)**	73.059(7)	73.071(4)	72.990(7)
**Volume (Å^3)**	7077.5(15)	7169.7(10)	7265.6(13)
**Z**	2	2	2
**ρ^{calc} (g cm^{-1})**	1.538	1.518	1.498
**Absorption coefficient (mm^{-1})**	3.174	3.133	3.092
**F(000)**	3262	3262	3262
**Crystal size (mm^3)**	0.480 x 0.210 x 0.040	0.480 x 0.210 x 0.040	0.480 x 0.210 x 0.040
**Theta range for data collection (°)**	1.151 to 27.485	1.143 to 27.485	1.132 to 27.485
**Index ranges**	−25≤h≤25, −25≤k≤25, −26≤l≤26	−25≤h≤25, −25≤k≤25, −26≤l≤26	−25≤h≤25, −25≤k≤25, −26≤l≤26
**Reflections collected**	244489	214939	236747
**Independent reflections**	32345 [R(int) = 0.0878]	62427 [R(int) = 0.0899]	32705 [R(int) = 0.1079]
**Completeness to theta = 25.000°**	99.8%	99.4%	99.1%
**Data / restraints / parameters**	32345 / 3201 / 1445	32427 / 3201 / 1445	32705 / 3201 / 1445
**Goodness-of-fit on F^2**	1.019	1.080	1.021
**Final R indices [I>2sigma(I)]**	R1 = 0.0599, wR2 = 0.1277	R1 = 0.1169, wR2 = 0.2109	R1 = 0.1034, wR2 = 0.1936
**R indices (all data)**	R1 = 0.0991, wR2 = 0.1487	R1 = 0.1654, wR2 = 0.2384	R1 = 0.1686, wR2 = 0.2285
**Largest diff. peak and hole (e$^-$Å^{-3})**	2.072 and −1.259	2.533 and −2.412	2.150 and −1.648

Table S9. Crystallographic data for [**1**($CN^tBu)_2$(THF)][PF_6] (3) at 100 K (final).

Compound	[**1**($CN^tBu)_2$(THF)][PF_6] (3) 100 K-*f*
**Empirical formula**	$C_{128}H_{128}Co_6F_6Fe_3N_8OP_7Se_8$
**CCDC Number**	2320350
**Formula weight**	3277.98
**Temperature (K)**	100(2)
**Wavelength (Å)**	0.71073
**Crystal system**	Triclinic
**Space group**	$P\bar{1}$
**a (Å)**	19.5288(16)
**b (Å)**	19.6098(16)
**c (Å)**	20.2366(16)
**α (°)**	89.811(4)
**β (°)**	71.407(4)
**γ (°)**	72.961(4)
**Volume (Å^3)**	6988.0(10)
**Z**	2
**ρ^{calc} (g cm^{-1})**	1.558
**Absorption coefficient (mm^{-1})**	3.215
**F(000)**	3262
**Crystal size (mm^3)**	0.480 x 0.210 x 0.040
**Theta range for data collection (°)**	1.156 to 27.484
**Index ranges**	$-25\leq h\leq 25$, $-25\leq k\leq 25$, $-26\leq l\leq 26$
**Reflections collected**	237264
**Independent reflections**	31792 [R(int) = 0.0799]
**Completeness to theta = 25.000°**	99.7%
**Data / restraints / parameters**	31792 / 3201 / 1445
**Goodness-of-fit on F^2**	1.019
**Final R indices [I>2sigma(I)]**	R1 = 0.0633, wR2 = 0.1322
**R indices (all data)**	R1 = 0.0966, wR2 = 0.1498
**Largest diff. peak and hole (e$^-$Å^{-3})**	2.027 and −1.348

S7 DFT Calculations

The $[\mathbf{1}]^{+}$ clusters were optimized at the uBPV86/def2SVP level of theory using a minimally adapted phosphinoamide ligand platform, $Ph_2PN^{(-)}Ph$, and methyl isocyanide, CNMe. These changes were made to simplify the computational problem without significantly altering the electronic or steric traits of our system. Analytical frequency calculations for these optimized structures reveal isocyanide stretching frequencies ($\nu_{CN,calc} = 2169 - 2179$ cm^{-1}) close to that observed experimentally ($\nu_{CN} = 2184$ cm^{-1}). Individual values for $\nu_{CN,calc}$ are provided in Table S13. Single-point electronics were then calculated at the B3LYP+//cc-pVTZ level of theory. Spin-multiplicity assignments for these calculations were informed by solid-state magnetometry data collected for $[\mathbf{1}(CN^{t}Bu)_2][PF_6]$, which exhibits a magnetic susceptibility of 9.5 cm^3 mol^{-1} K at 298 K (Section S4). When taken into consideration with its corresponding ^{57}Fe Mössbauer spectrum, these data are indicative of a magnetic ground state with three $s = 2$ Fe(II) edge metals and a $s = 1/2$ $[Co_6Se_8]^{+}$ ($S_{Tot} = 13$; 9.4 cm^3 mol^{-1} K, $g = 2$). For neutral *cis*-(α,β)-**1**$(CNMe)_2$, a $S_{Tot} = 12$ spin state was applied on the basis of prior Evans method magnetic susceptibility measurements and ^{57}Fe Mössbauer spectra.

All calculations were performed using the Gaussian 16, Revision B.01 quantum chemistry program package for the Linux operating system.[13] The initial starting point geometries were adapted from the corresponding crystallographically obtained structures and optimized to a stationary point, followed by analytical frequency calculations (Hessian) to confirm that no imaginary frequencies were present. The geometry optimizations were performed using DFT calculations at a pure GGA functional level using Becke's 1988 gradient-corrected exchange functional[14] and Perdew's 1986 electron correlation functional[15] (BPV86) and def2SVP[16,17] as a basis set. Using the optimized geometries, single point calculations were conducted using B3LYP+[18–21] as a functional and the correlation consistent basis set, cc-pVTZ.[22–26] These single point calculations were used to generate density of states plots, orbital energy diagrams, and spin density calculations. Multiwfn was used to generate partial density of states and molecular orbital isosurface plots, and to perform Hirshfeld spin density and charge analysis.[27] To reduce calculation time, geometry optimizations and frequency calculations were run over multiple nodes using an interactive batch file, which necessitates the use of %LindaWorker and %usessh. Further, to aid in the identification of a stationary point, a quadratic convergence algorithm was applied to the self-consistent field should the first-order SCF convergence fail (e.g., scf=(xqc,maxconventional=256)). Optimization of *trans*-(α,β)-$[\mathbf{1}(CNMe)(THF)]^{+}$, (α,α,β)-$[\mathbf{1}(CNMe)_2(THF)]^{+}$, and *cis*-(α,β)-$[\mathbf{1}(THF)_2]^{+}$ proved to be challenging and required the implementation of a fragmented initial guess (see input below). Despite fragmentation of the initial guess, these calculations yielded structural and electronic parameters highly similar to the non-fragmented counterpart, *cis*-(α,β)-$[\mathbf{1}(CNMe)_2]^{+}$, as illustrated by Hirshfeld spin density and Hirshfeld charge values provided below. Interestingly, free optimization of the experimentally unobserved intermediate (α,α,β)-$[\mathbf{1}(CNMe)(THF)_2]^{+}$ resulted in dissociation of the β-oriented THF molecule to yield *trans*-(α,β)-$[\mathbf{1}(CNMe)(THF)]^{+}$. Accordingly, this species was optimized as a transition state using the input file detailed below, with frequency calculations confirming the presence of a singular negative eigenvalue.

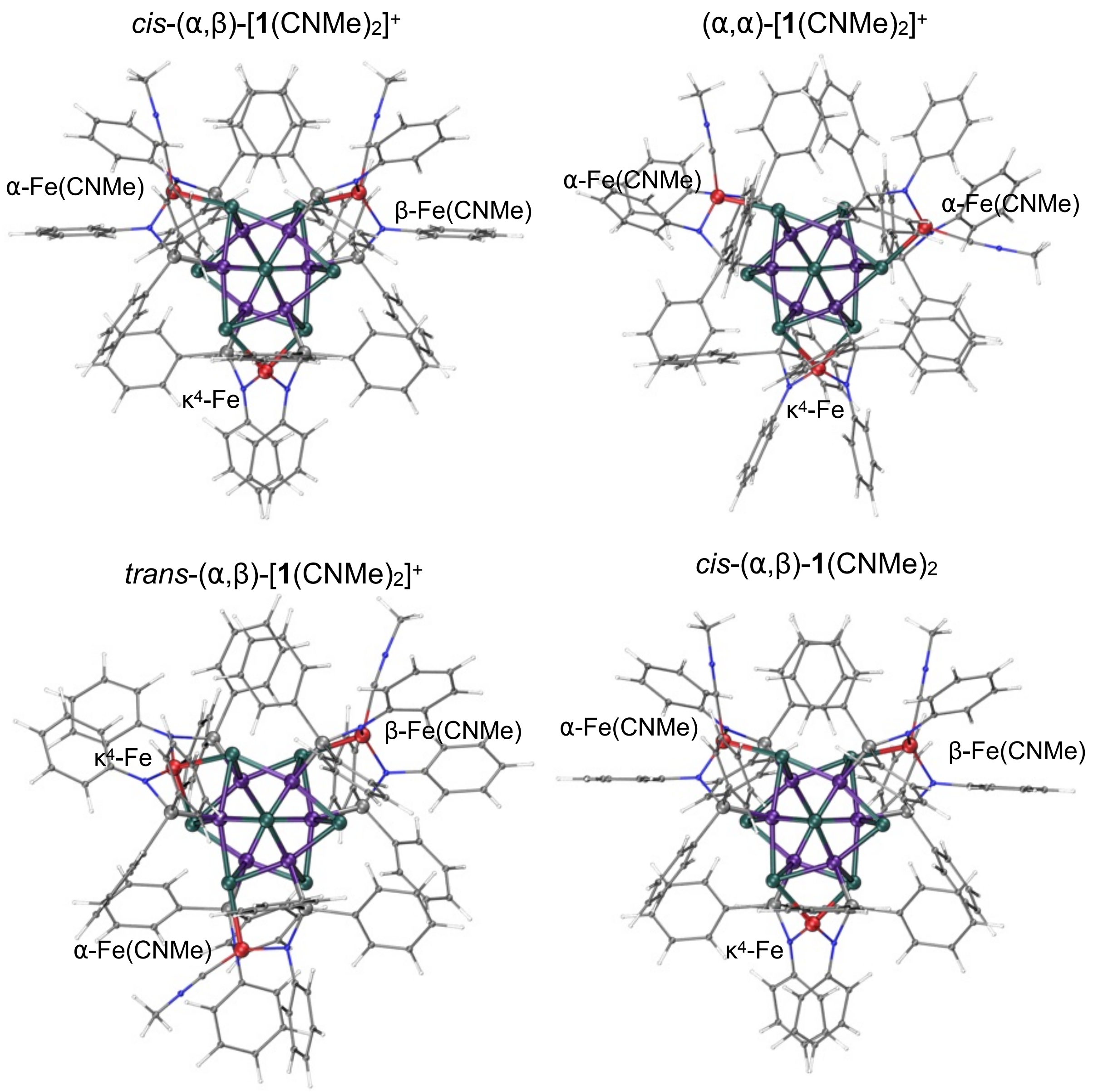

Figure S44. Ground state structures of *cis*-(α,β)-[**1**$(CNMe)_2$]$^+$, (α,α)-[**1**$(CNMe)_2$]$^+$, *trans*-(α,β)-[**1**$(CNMe)_2$]$^+$, and *cis*-(α,β)-**1**$(CNMe)_2$. Optimized at the uBPV86//def2SVP level of theory.

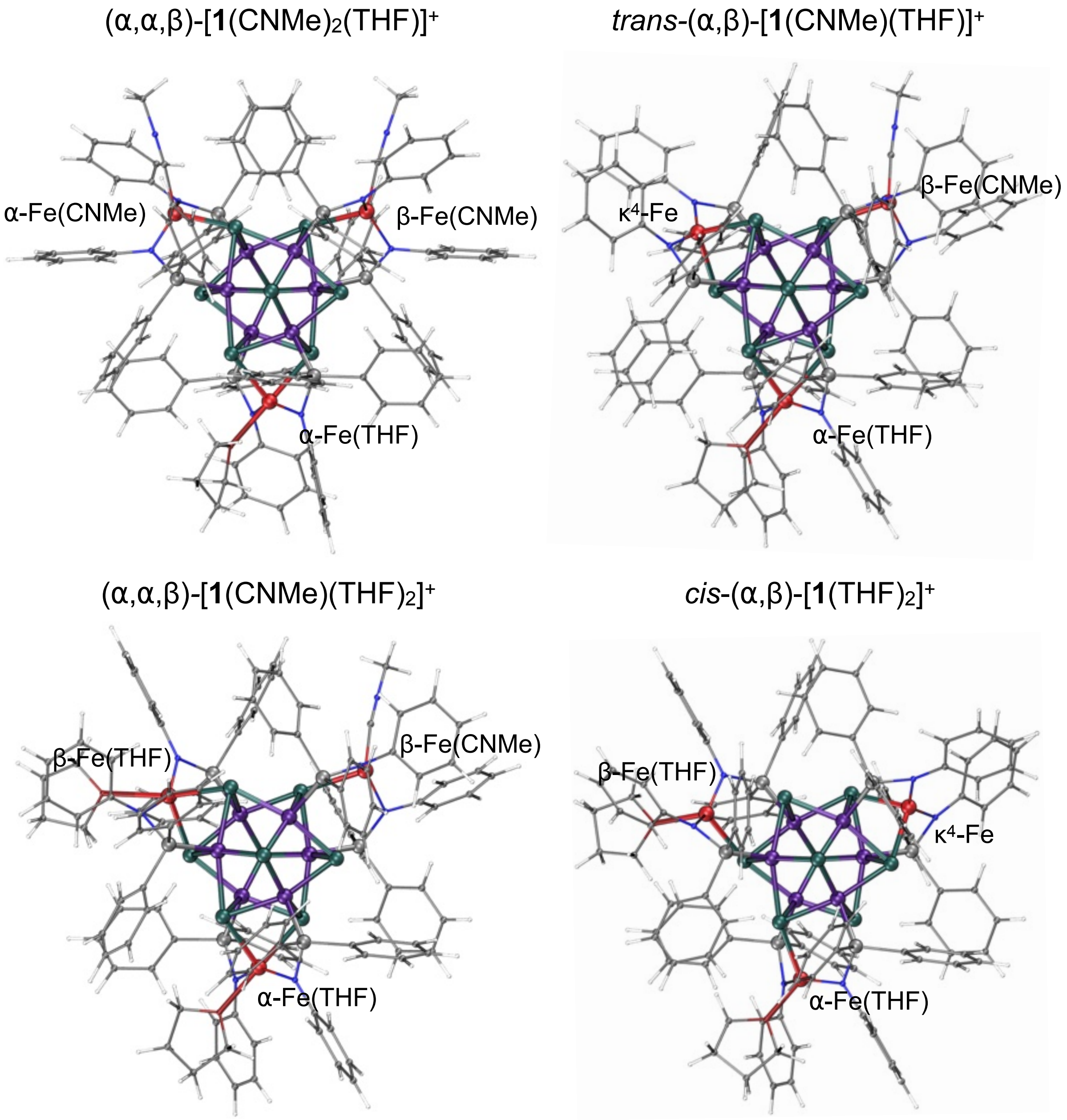

Figure S45. Ground state structures of (α,α,β)-[**1**(CNMe)$_2$(THF)]$^+$, *trans*-(α,β)-[**1**(CNMe)(THF)]$^+$, and (α,α,β)-[**1**(CNMe)(THF)$_2$]$^+$, and *cis*-(α,β)-[**1**(THF)$_2$]$^+$. Optimized at the uBPV86//def2SVP level of theory.

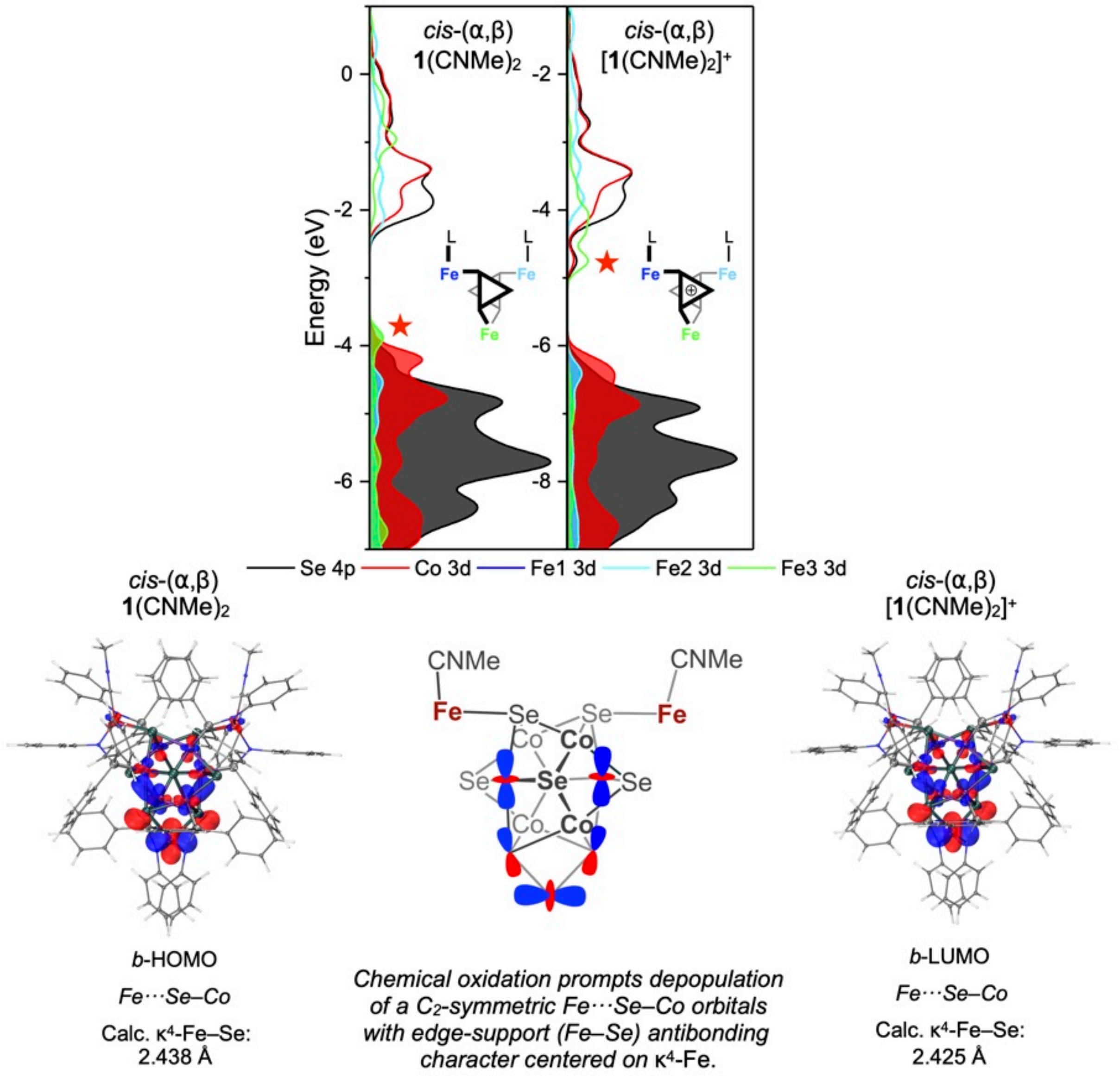

Figure S46. **Impact of mono-oxidation on the electronic structure.** *p*-DOS plots and corresponding frontier MO isosurface plots (0.03 a.u.) for *cis*-(α,β)-**1**$(CNMe)_2$ and *cis*-(α,β)-[**1**$(CNMe)_2]^+$, revealing how high-lying Fe 3d states centered on κ^4-Fe are depleted upon chemical oxidation, which reinforces edge-support bonding. Calculations were run at the uB3LYP+//cc-PVTZ level of theory.

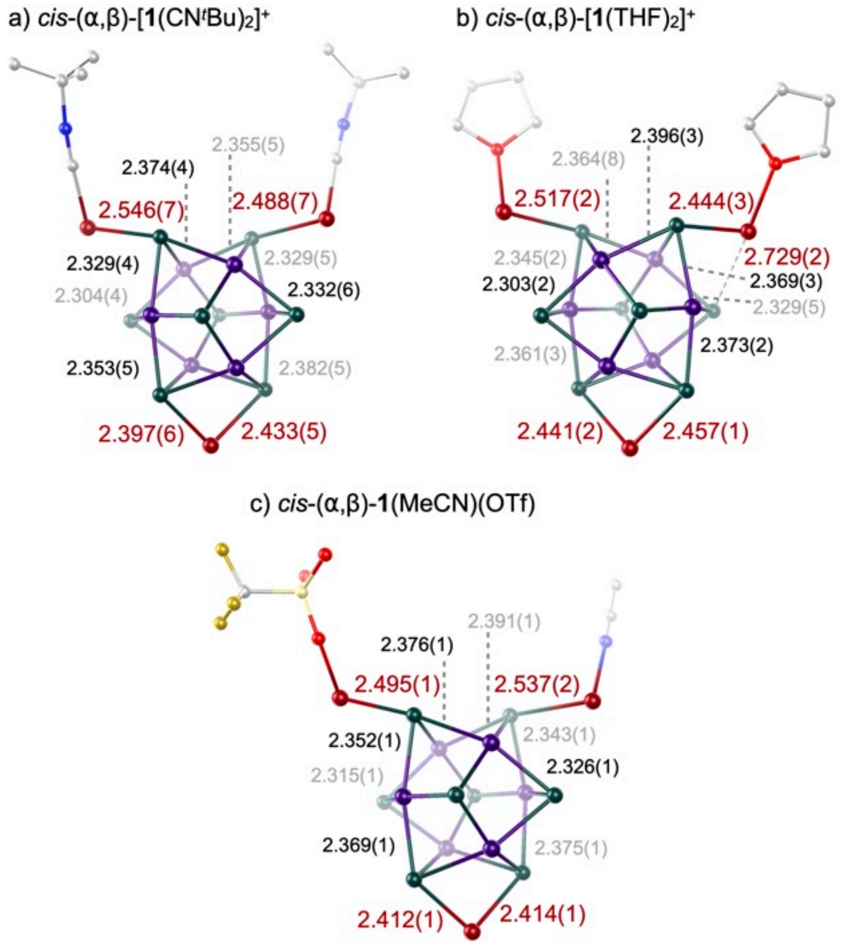

Figure S47. **Experimental bond metrics are aligned with computational evidence that mono-oxidation reinforces edge-support binding at the *κ*⁴-Fe site.** Single crystal X-ray structures of the [**1**(L)$_2$]$^+$ series, [**1**(CN^{t}Bu)$_2$][PF$_6$], [**1**(THF)$_2$][PF$_6$], and **1**(MeCN)(OTf) including select Fe–Se and Co–Se interatomic distances (Å). All hydrogen atoms, the phosphino-amide ligand framework, PF_6^- counterion, and any instances of disorder are omitted for clarity.

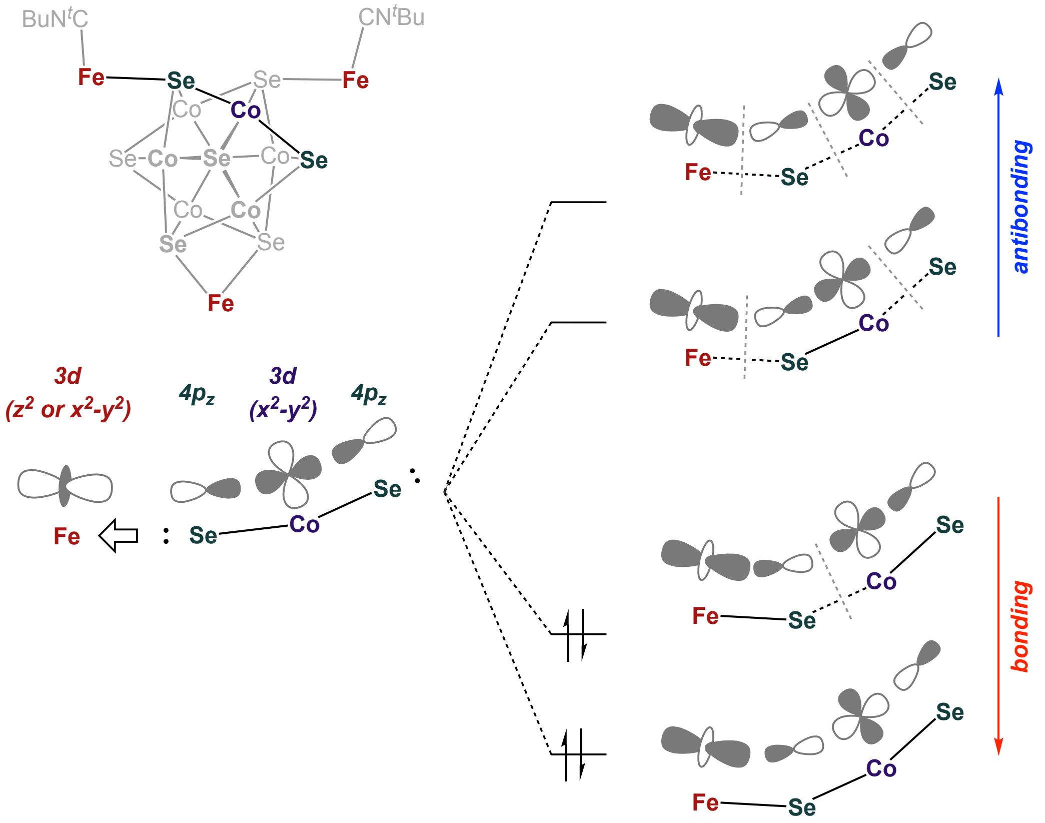

Figure S48. **Fe···Se···Co···Se delocalized orbitals are approximated as symmetry adapted linear combinations** (SALCs) of Fe ($3d_{z2}$ or $3d_{x2-y2}$), Se_a $4p_z$, Co $3d_{x2-y2}$, and Se_b $4p_z$ orbitals.

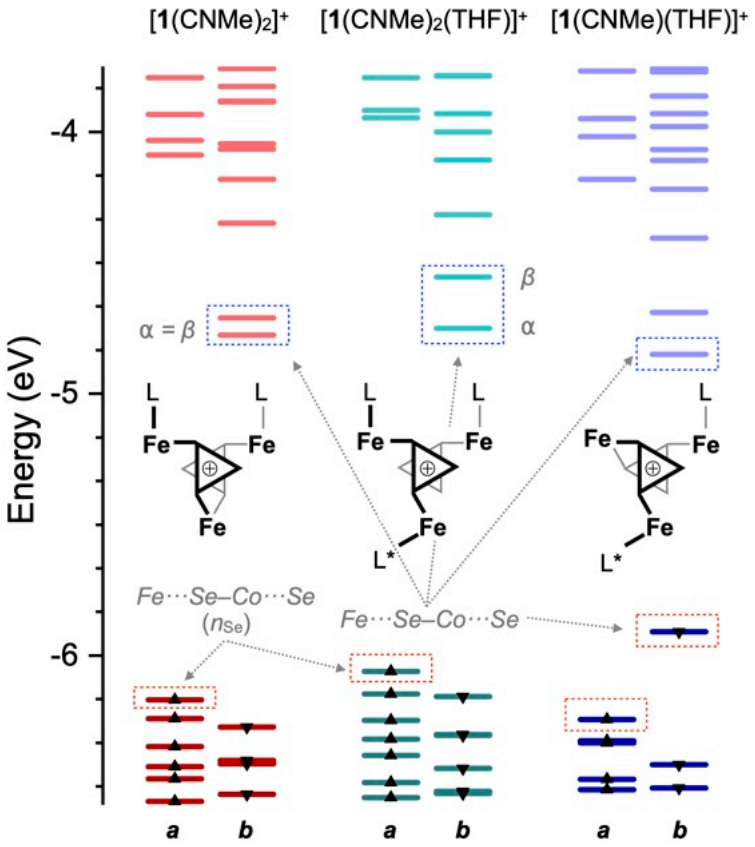

Figure S49. **Frontier molecular orbital energy diagrams of the crystallographically resolved [1]$^+$ ligand exchange series**, *cis*-(α,β)-[**1**($CNMe)_2$]$^+$, (α,α,β)-[**1**($CNMe)_2$(THF)]$^+$ and *trans*-(α,β)-[**1**(CNMe)(THF)]$^+$. The *a*-HOMO and *b*-HOMO orbitals for all three compounds project along the Fe(L)–Se bond axis with Fe⋯Se–Co⋯Se antibonding character. Isosurface plots for select frontier molecular orbitals for [**1**]$^+$ clusters and neutral *cis*-(α,β)-**1**($CNMe)_2$ are provided in Figures S52-S59.Here, *a* and *b* denote the up and down spin manifolds, respectively. Calculations were run at the uB3LYP+//cc-PVTZ level of theory.

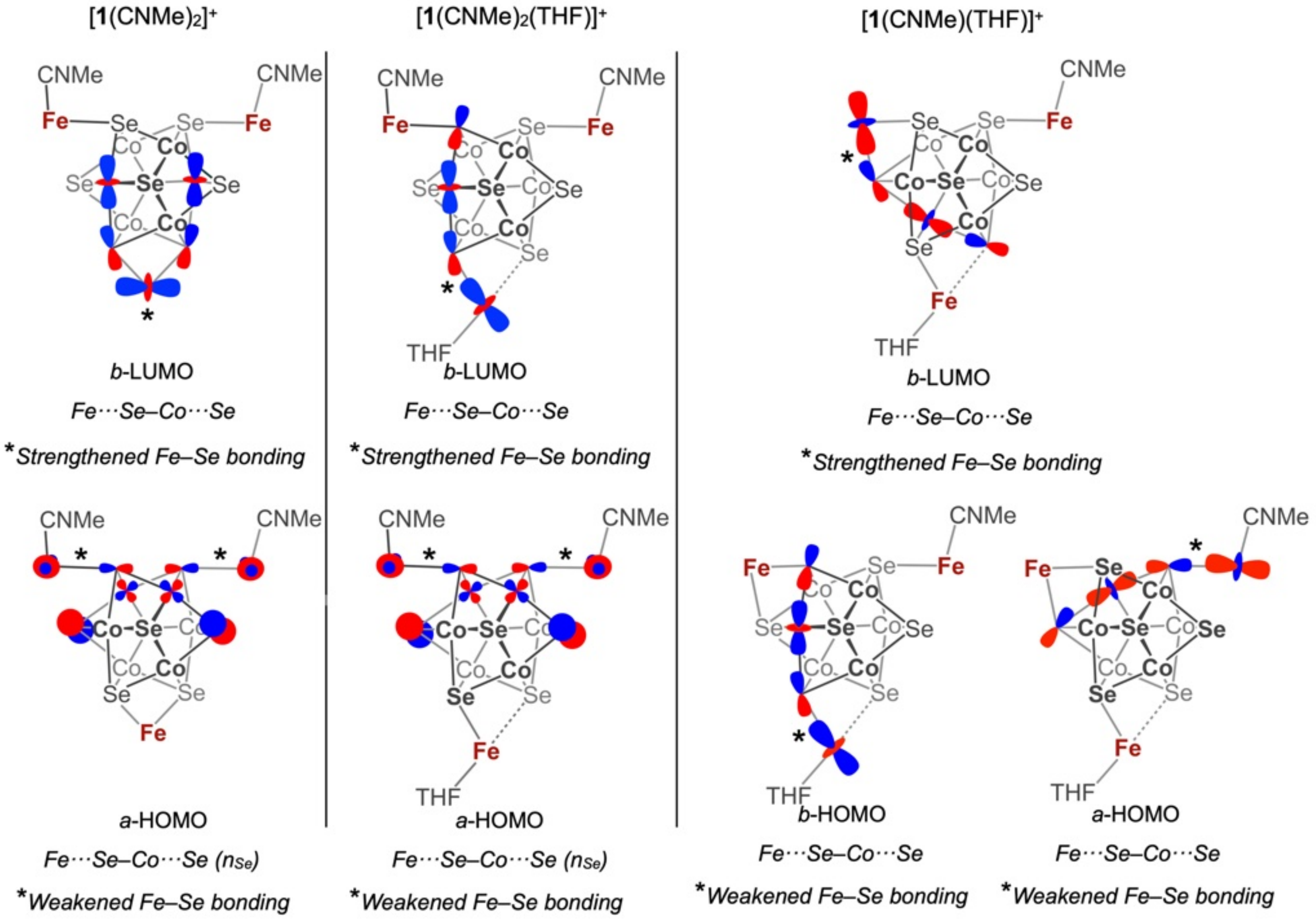

Figure S50. **SALC representation of key frontier orbitals.** Qualitative representations of frontier molecular orbitals for the crystallographically-resolved ligand exchange participants, *cis*-(α,β)-[**1**($CNMe)_2$]$^+$, (α,α,β)-[**1**($CNMe)_2$(THF)]$^+$ and *trans*-(α,β)-[**1**(CNMe)(THF)]$^+$. Asterisks denote the Fe–Se bonds that we anticipate being impacted by the population or depopulation of the corresponding orbital.

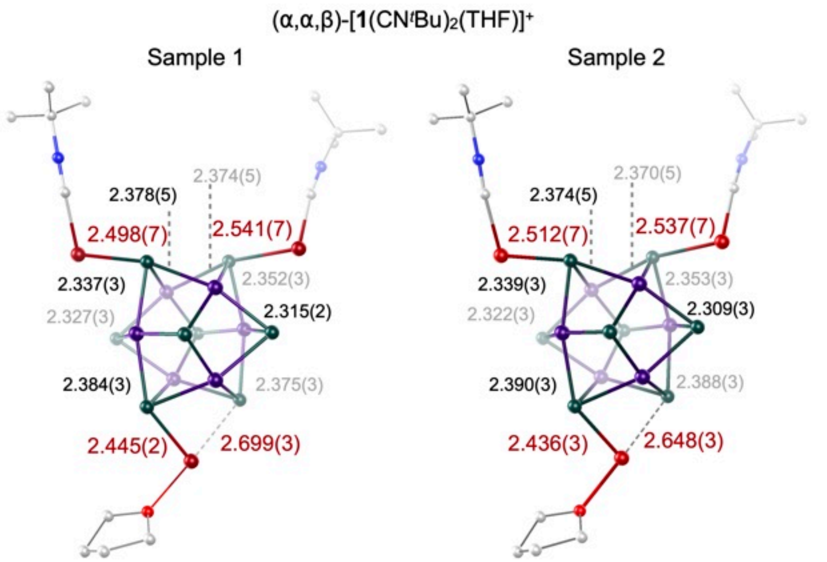

Figure S51. **Experimental bond metrics for [1(CN^{t}Bu)$_2$(THF)][PF$_6$], revealing long Fe…Se bonds at the Fe(CN^{t}Bu) edges, in alignment with computational predictions.** Single crystal X-ray structures of samples 1 (left) and 2 (right) of [**1**(CN^{t}Bu)$_2$(THF)][PF$_6$], including select Fe–Se and Co–Se interatomic distances (Å). In (α,α,β)-[**1**(CN^{t}Bu)$_2$(THF)][PF$_6$], the Fe(CN^{t}Bu) edges exhibit long Fe–Se bond lengths comparable to those in [**1**(CN^{t}Bu)$_2$][PF$_6$], consistent with the populated *a*-HOMO. All hydrogen atoms, the phosphino-amide ligand framework, PF_6^- counterion, and any instances of disorder are omitted for clarity.

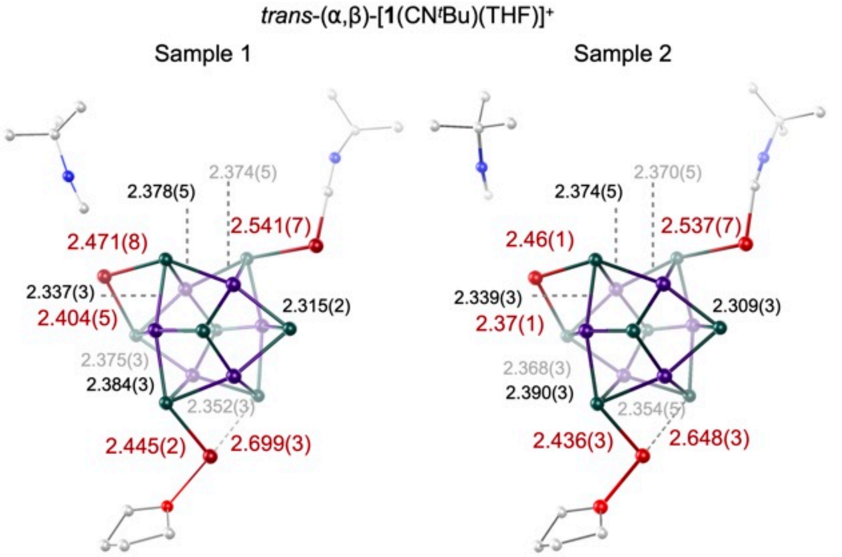

Figure S52. **Experimental bond metrics reveal a contraction of the Fe…Se bond, aligned with computational prediction.** Single crystal X-ray structures of samples 1 (left) and 2 (right) of *trans*-(α,β)-[1(CN^{t}Bu)(THF)][PF$_6$], including select Fe–Se and Co–Se interatomic distances (Å). In *trans*-(α,β)-[**1**(CN^{t}Bu)(THF)][PF$_6$], the newly formed κ^4-Fe site exhibits one markedly contracted Fe–Se bond that coincides with the Fe···Se–Co···Se *b*-LUMO. All hydrogen atoms, the phosphino-amide ligand framework, PF_6^- counterion, and any instances of disorder are omitted for clarity.

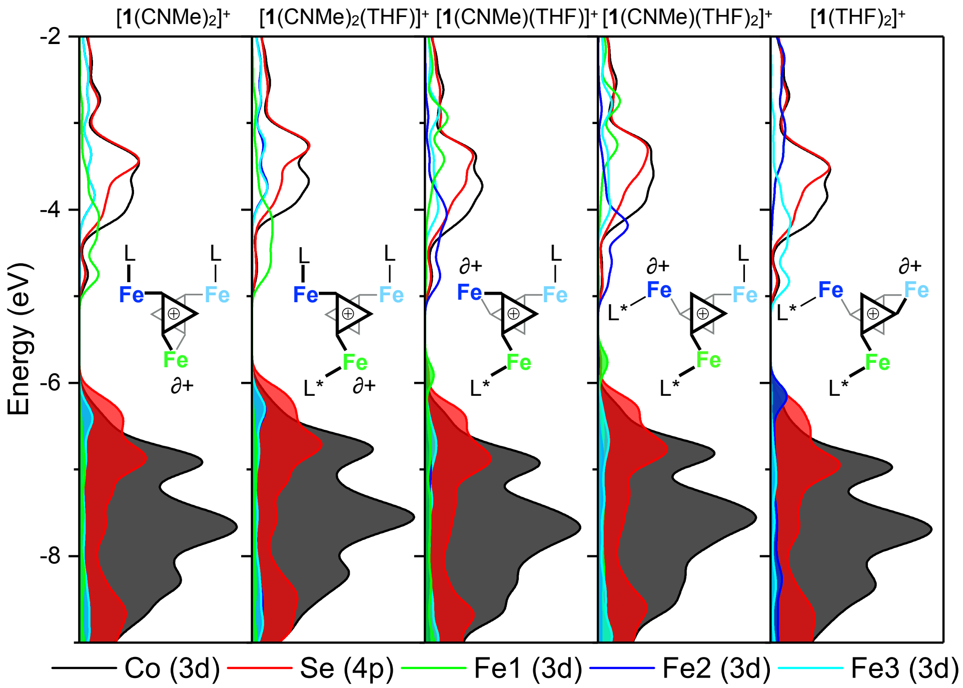

Figure S53. **Individual Fe contributions to the frontier electronic states.** Partial density of states plots for the proposed step-wise ligand exchange reaction including those of *cis*-(α,β)-[**1**(CNMe)$_2$]$^+$, (α,α,β)-[**1**(CNMe)$_2$(THF)]$^+$, *trans*-(α,β)-[**1**(CNMe)(THF)]$^+$, (α,α,β)-[**1**(CNMe)(THF)$_2$]$^+$, and *cis*-(α,β)-[**1**(THF)$_2$]$^+$. Calculations were run at the uB3LYP+//cc-PVTZ level of theory. Migrating partial positive charge assigned on the basis of calculated Hirshfeld charge density (values provided in Table S15).

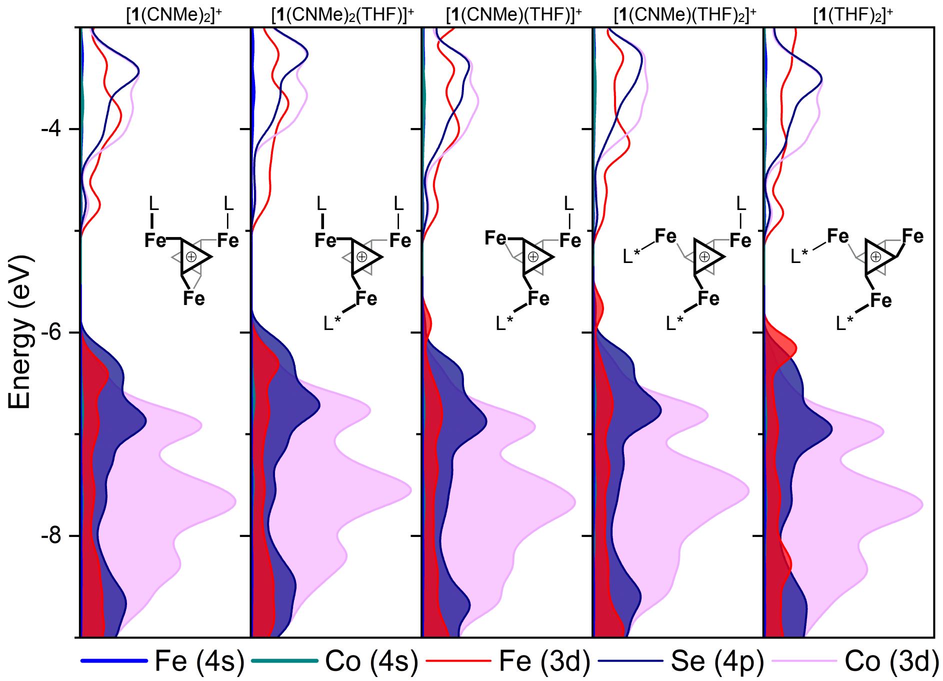

Figure S54. **Fe and Co 4s provide minimal contribution to the frontier electronic states.** Partial density of states (*a* + *b*) plots calculated for *cis*-(α,β)-[**1**(CNMe)$_2$]$^+$, (α,α,β)-[**1**(CNMe)$_2$(THF)]$^+$, *trans*-(α,β)-[**1**(CNMe)(THF)]$^+$, (α,α,β)-[**1**(CNMe)(THF)$_2$]$^+$, and *cis*-(α,β)-[**1**(THF)$_2$]$^+$. Calculations were run at the uB3LYP+//cc-PVTZ level of theory.

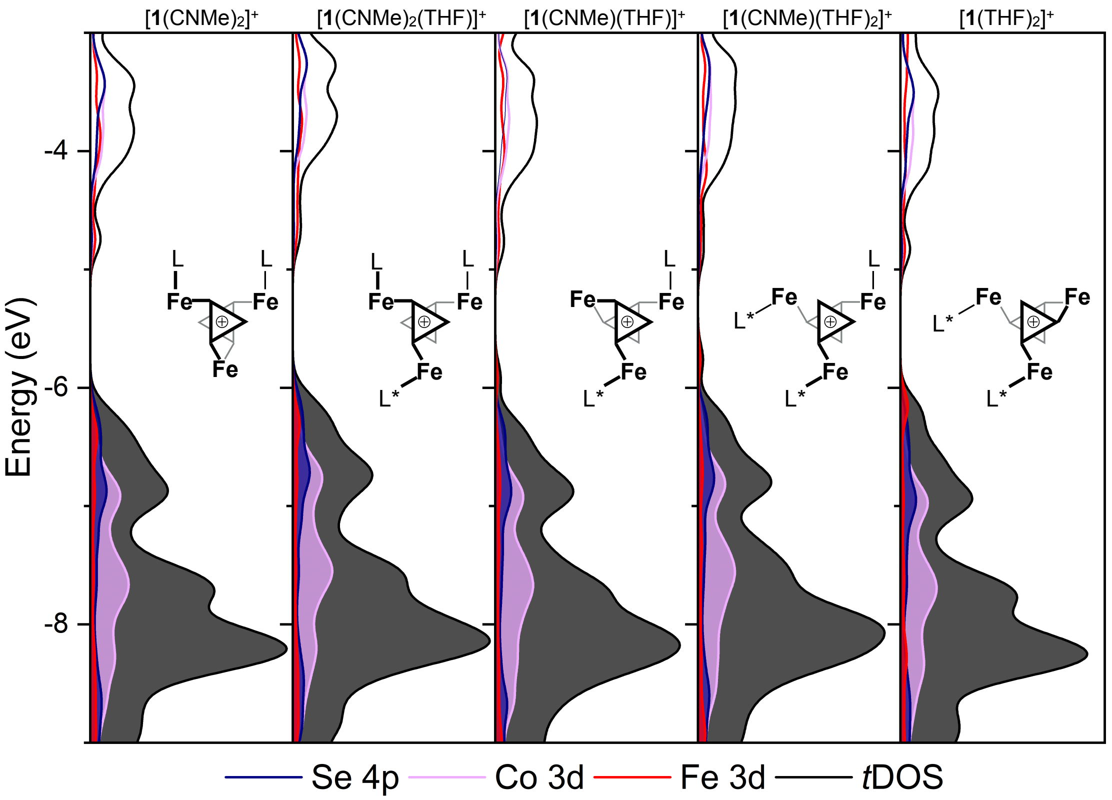

Figure S55. **Total density of states** (*a + b)* including Co 3d, Fe 3d and Se 4p contributions for *cis*-(α,β)-[**1**(CNMe)$_2$]$^+$, (α,α,β)-[**1**(CNMe)$_2$(THF)]$^+$, *trans*-(α,β)-[**1**(CNMe)(THF)]$^+$, (α,α,β)-[**1**(CNMe)(THF)$_2$]$^+$, and *cis*-(α,β)-[**1**(THF)$_2$]$^+$. Calculations were run at the uB3LYP+//cc-PVTZ level of theory.

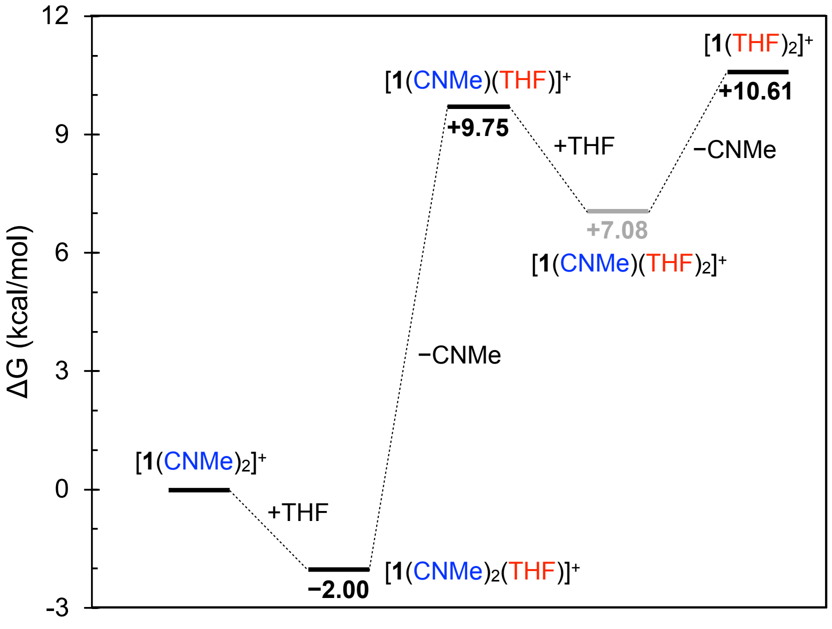

Figure S56. **Calculated energetics for the proposed step-wise ligand exchange reaction coordinate.** Calculations were run at the uB3LYP+//cc-PVTZ level of theory. The experimentally unobserved intermediate (α,α,β)-[**1**(CNMe)(THF)$_2$]$^+$ resulted in dissociation of the β-oriented THF molecule to yield *trans*-(α,β)-[**1**(CNMe)(THF)]$^+$. Accordingly, this species was optimized as a transition state using the input file detailed below, with frequency calculations confirming the presence of a singular negative eigenvalue.

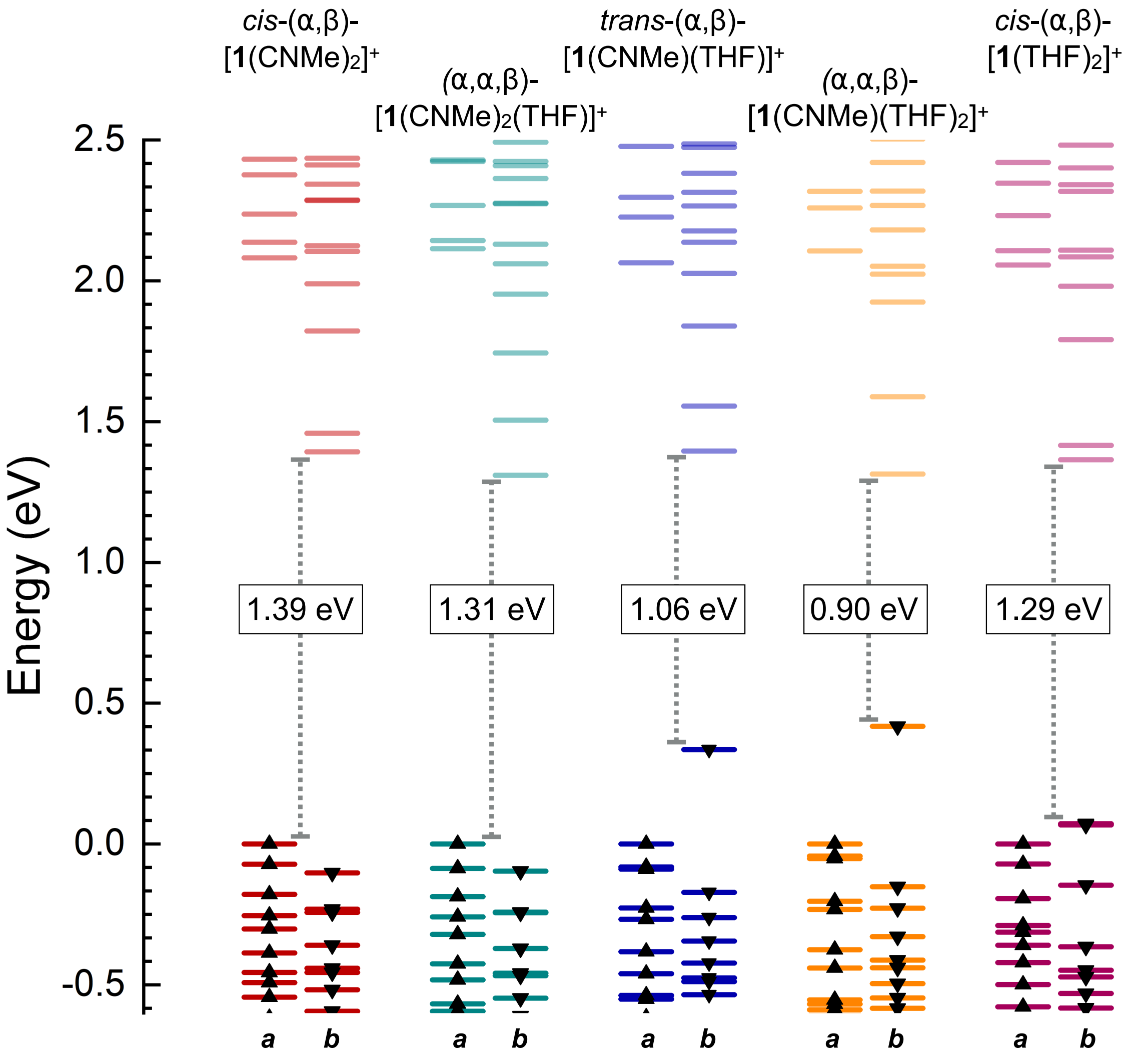

Figure S57. **Frontier molecular orbital energy diagrams for all proposed intermediates in the ligand exchange series.** The orbitals are normalized to the *a*-HOMO, which maintains the same Fe⋯Se–Co⋯Se topology, projecting along the κ^3-Fe(L)–Se bond and exhibiting primary contributions from the distal Se 4p, throughout this series of compounds. Here, *a* and *b* are used to abbreviate the up and down spin manifolds. Calculations were run at the uB3LYP+//cc-PVTZ level of theory.

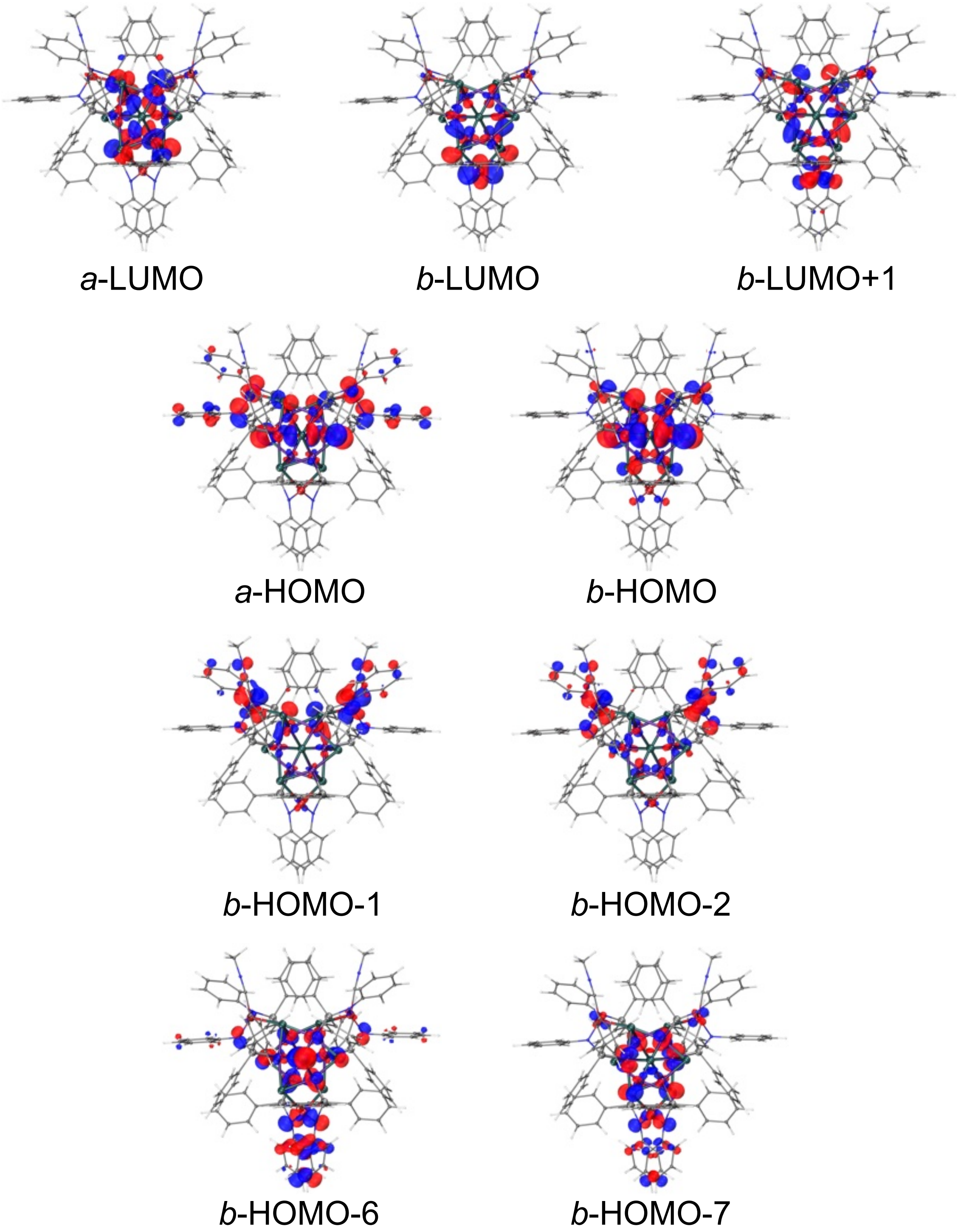

Figure S58. **Isosurface plots (0.03 au) for select molecular orbitals of *cis*-(α,β)-[1(CNMe)$_2$]$^+$**. Orbitals *b*-LUMO and *b*-LUMO+1 exhibit C_2-symmetric Fe···Se–Co···Se topology projecting along the Fe–Se bonds of the κ^4-Fe edge. Both *a*-HOMO and *b*-HOMO also exhibit Fe···Se–Co···Se topology, however in this case they project along the Fe–Se bonds of the Fe(CNMe) edges and exhibit predominantly Se 4p character. *b*-HOMO-1, *b*-HOMO-2, *b*-HOMO-6, and *b*-HOMO-7 roughly C_2-symmetric in the case of *cis*-(α,β)-[**1**(CNMe)$_2$]$^+$, but are polarized toward the α/β face upon coordination of THF in (α,α,β)-[**1**(CNMe)$_2$(THF)]$^+$.

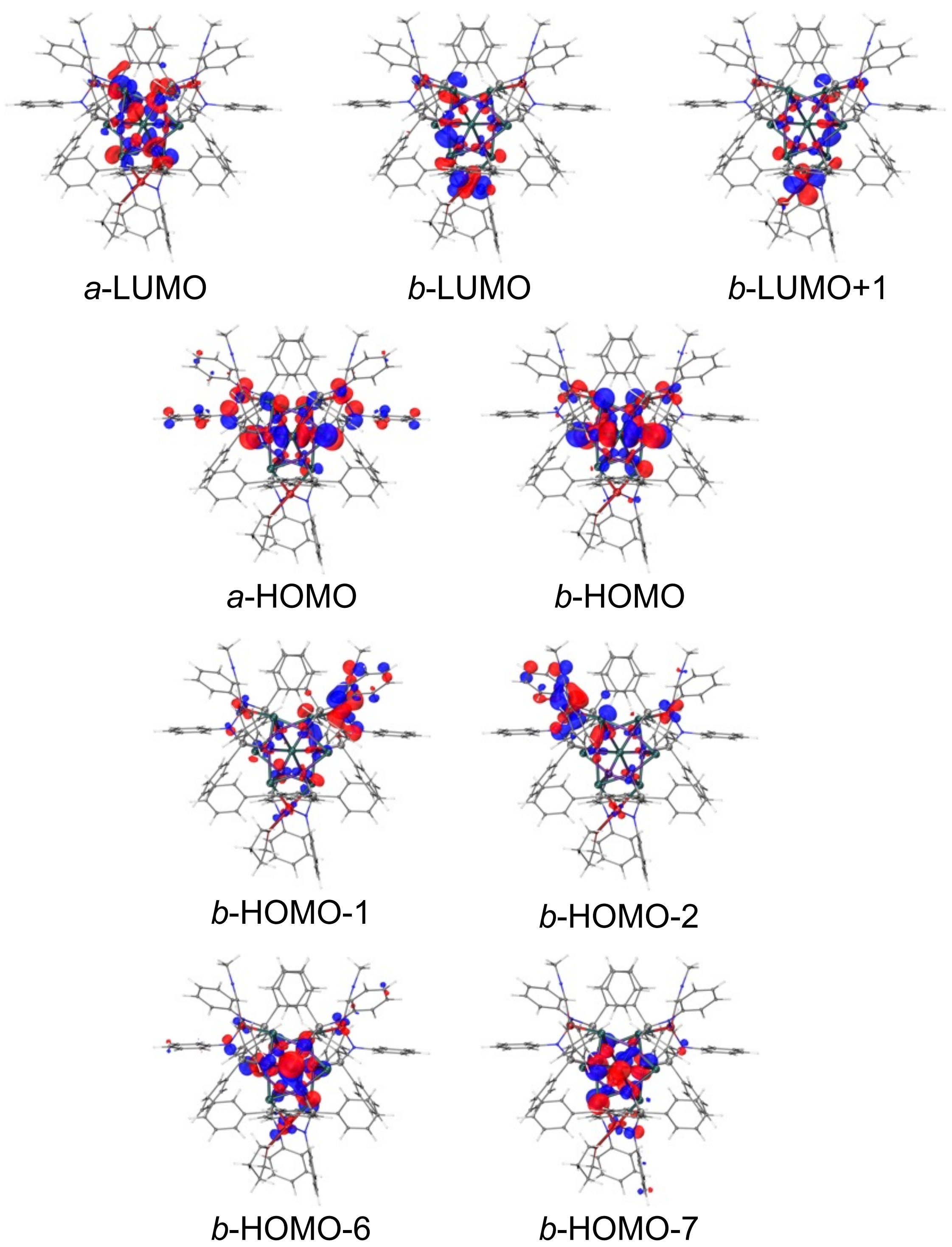

Figure S59. **Isosurface plots (0.03 au) for select molecular orbitals of (α,α,β)-[1(CNMe)$_2$(THF)]$^+$.** Orbitals *b*-LUMO and *b*-LUMO+1 exhibit α/β polarized Fe⋯Se–Co⋯Se topology projecting along the Fe–Se bonds of the pseudo-κ^4-Fe(THF) edge. Both *a*-HOMO and *b*-HOMO also exhibit Fe⋯Se–Co⋯Se topology, however in this case they project along the Fe–Se bonds of the Fe(CNMe) edges and exhibit predominantly Se 4p character. *b*-HOMO-1, *b*-HOMO-2, *b*-HOMO-6, and *b*-HOMO-7 are polarized toward the α/β Fe edges upon coordination of THF roughly, relative to those of *cis*-(α,β)-[**1**(CNMe)$_2$]$^+$. These orbitals illustrate how THF coordination polarizes Co-Se bonding within the Co_6Se_8 support and Fe-CNMe bonding at neighboring edge sites.

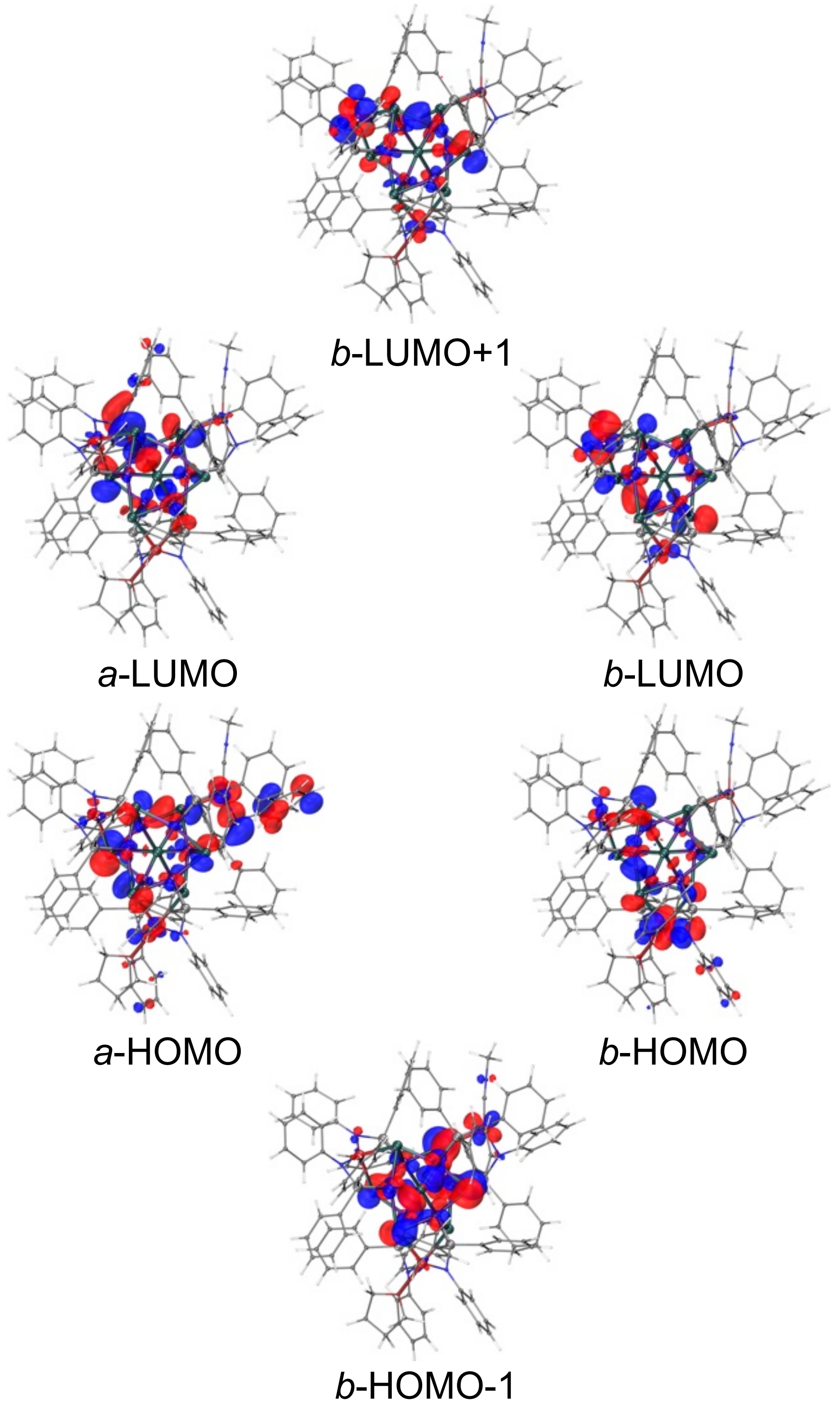

Figure S60. **Isosurface plots (0.03 au) for select molecular orbitals of *trans*-(α,β)-[1(CNMe)(THF)]$^{+}$.**

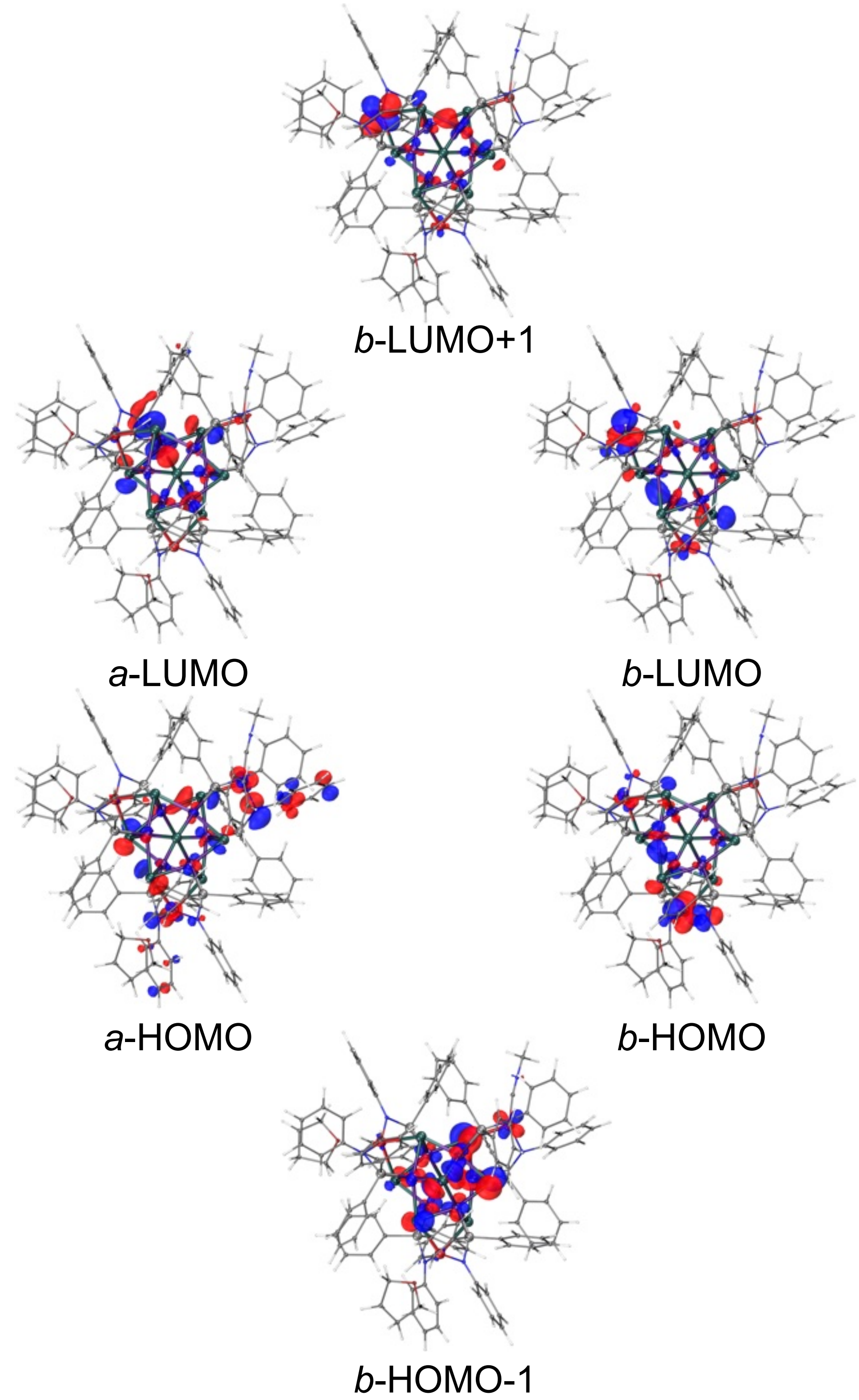

Figure S61. **Isosurface plots (0.03 au) for select molecular orbitals of (α,α,β)-$[1(CNMe)(THF)_2]^+$.**

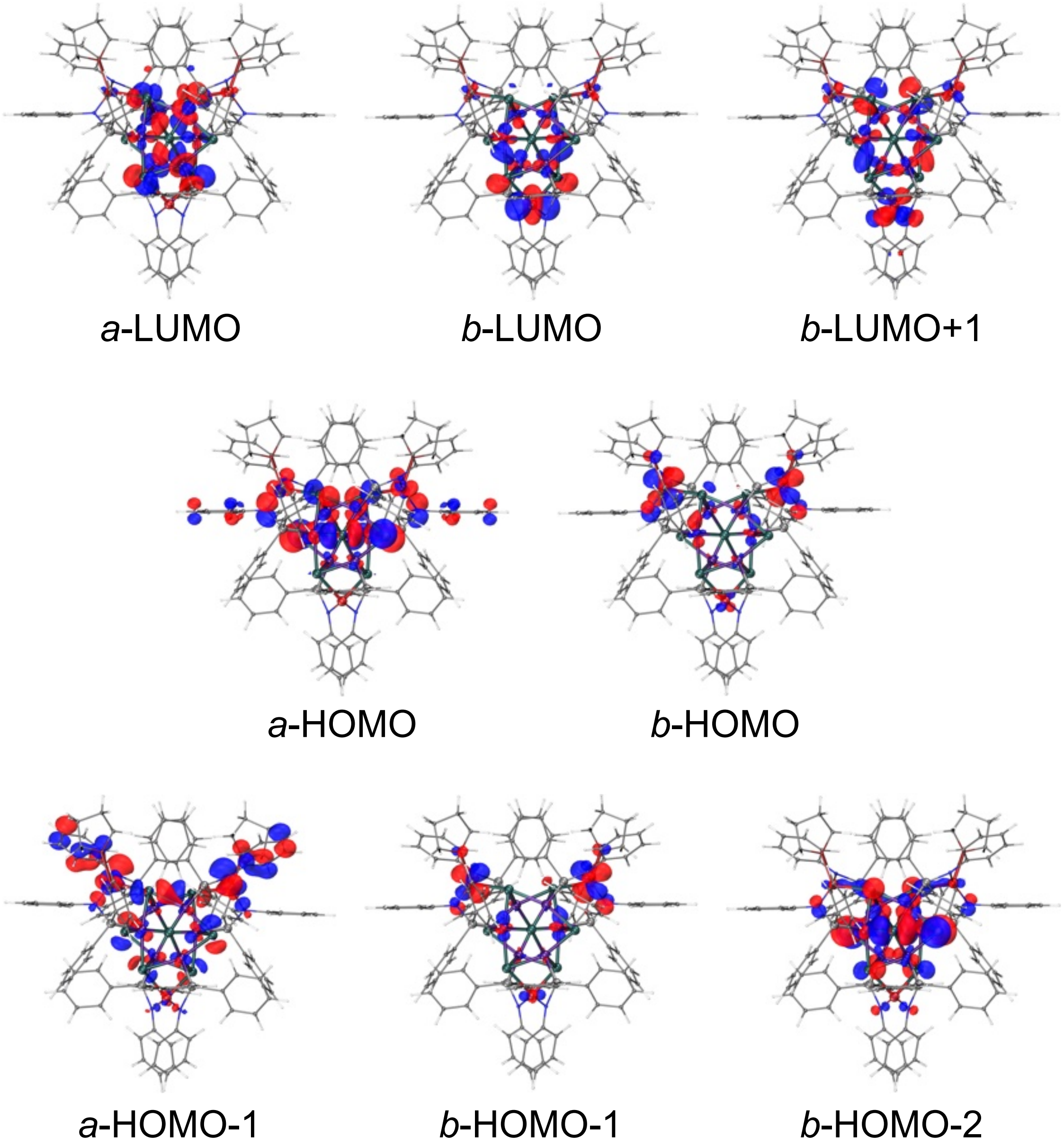

Figure S62. **Isosurface plots (0.03 au) for select molecular orbitals of *cis*-(α,β)-$[1(THF)_2]^+$.**

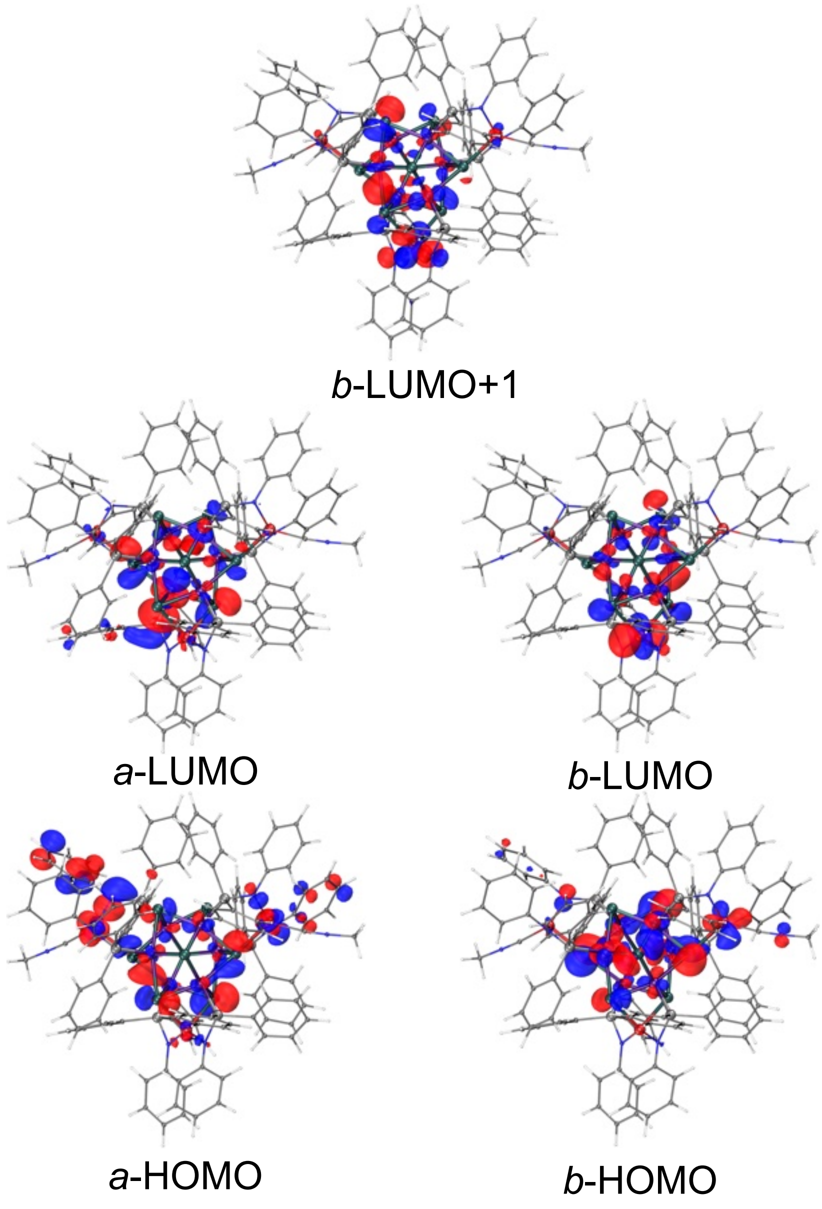

Figure S63. I**sosurface plots (0.03 au) for select molecular orbitals of *trans*-(α,β)-[1(CNMe)$_2$]$^+$.**

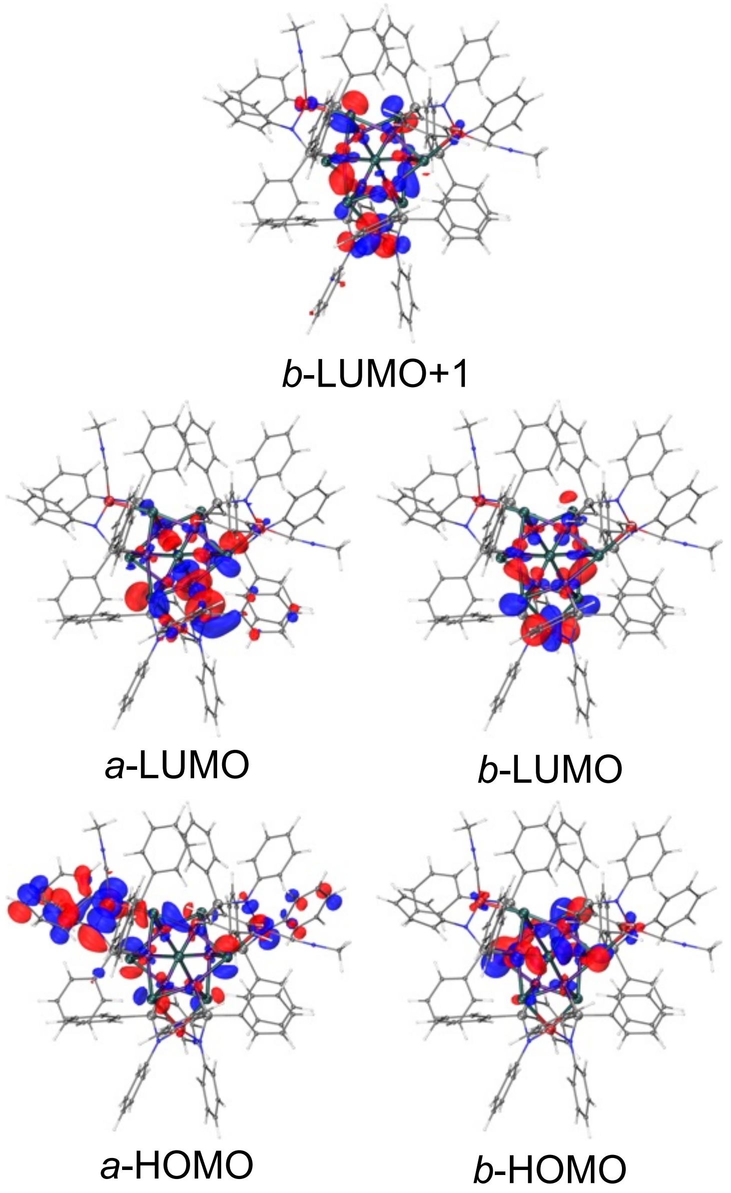

Figure S64. **Isosurface plots (0.03 au) for select molecular orbitals of (α,α)-[1(CNMe)$_2$]$^+$.**

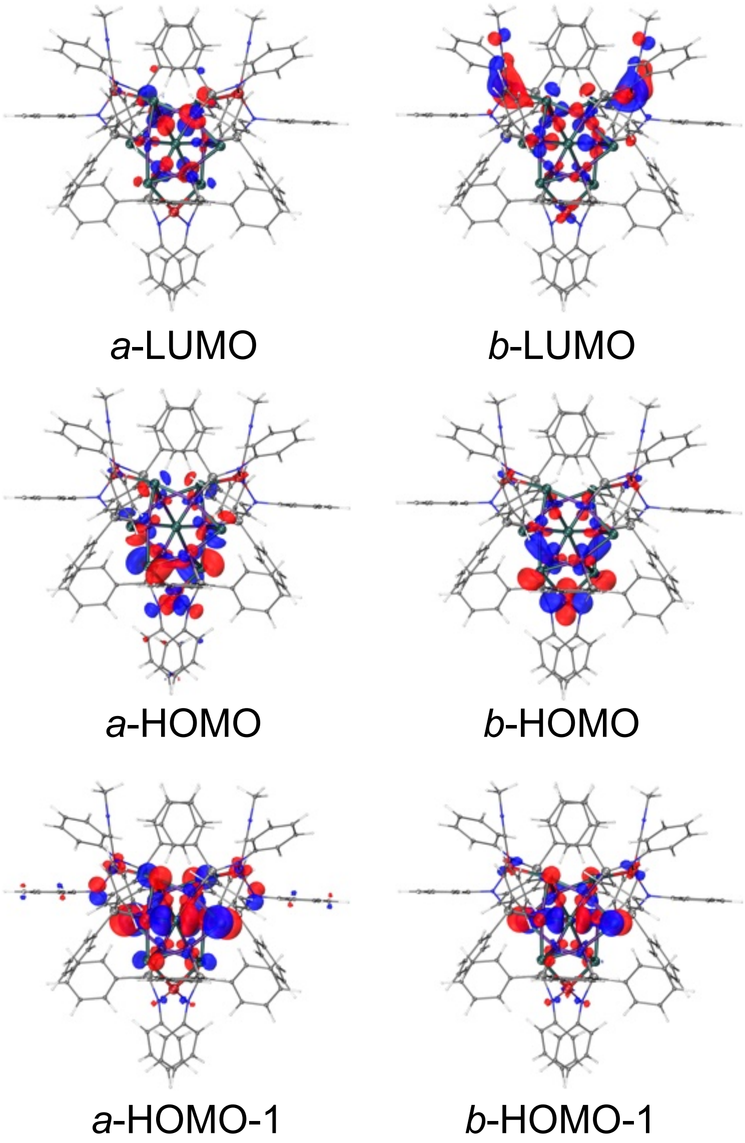

Figure S65. **Isosurface plots (0.03 au) for select molecular orbitals of *cis*-(α,β)-1(CNMe)$_2$.** The *a*-HOMO and *b*-HOMO calculated for neutral *cis*-(α,β)-**1**(CNMe)$_2$ are similar in composition to the *a*-LUMO and *a*-LUMO+1 of oxidized *cis*-(α,β)-[**1**(CNMe)$_2$]$^+$, exhibiting pseudo-C_2 symmetric Fe···Se–Co···Se character. Depletion of these states upon chemical oxidation would localize partial positive charge character and reinforce Fe–Se bonding at the κ^4-Fe.

Table S10. Comparison of bond metrics of the DFT optimized structures of *cis*-(α,β)-[**1**$(CNMe)_2]^+$ and [**1**$(CNMe)_2(THF)]^+$ and those of the related single crystal X-ray diffraction structures of [**1**$(CN^tBu)_2][PF_6]$ and [**1**$(CN^tBu)_2(THF)][PF_6]$.

Compound	Interatomic Distance (Å)				
	Fe…Se	Fe–L	Co–Se (avg.)	Co–P (avg.)	$Fe–N^{Tol}$ (avg.)
[**1**$(CN^tBu)_2][PF_6]$ (crystallographic)	2.488(7)[a]<br>2.546(7)[a]<br>2.397(6)[c]<br>2.433(5)[c]	2.04(2)[a]<br>2.08(2)[a]<br>--	2.35	2.17	2.01
cis-(α,β)-[**1**$(CNMe)_2]^+$ (calculated)	2.441[a]<br>2.441[a]<br>2.426[c]<br>2.426[c]	1.998[a]<br>1.998[a]<br>--	2.36	2.18	1.98
[**1**$(CN^tBu)_2(THF)][PF_6]$ (crystallographic)	2.498(1)[a]<br>2.541(1)[a]<br>2.450(1)[b]<br>2.699(3)[b]	2.12(1)[a]<br>2.11(3)[a]<br>2.237(5)[b]	2.35	2.16	1.99
[**1**$(CNMe)_2(THF)]^+$ (calculated)	2.439[a]<br>2.453[a]<br>2.449[b]<br>2.588[b]	2.001[a]<br>1.993[a]<br>2.355[b]	2.36	2.17	1.99

[a]Fe(CNMe/CN^tBu) edge site; [b]Fe(THF) edge site; [c]κ^4-Fe edge site.

Table S11. Comparison of bond metrics of the DFT optimized structures of *trans*-(α,β)-[**1**(CNMe)(THF)]$^+$ and *cis*-(α,β)-[**1**(THF)$_2$] and those of the related single crystal X-ray diffraction structures of *trans*-(α,β)-[**1**(CN^{t}Bu)(THF)][PF$_6$] and *cis*-(α,β)-[**1**(THF)$_2$][PF$_6$].

**Compound**	**Interatomic Distance (Å)**				
	Fe…Se	Fe–L	Co–Se (avg.)	Co–P (avg.)	Fe–N^{Tol} (avg.)
[**1**(CN^{t}Bu)(THF)][PF$_6$]<br>(crystallographic)	2.541(7)[a]<br>2.450(2)[b]<br>2.699(3)[b]<br>2.404(5)[c]<br>2.471(7)[c]	2.1(1)[a]<br>2.237(5)[b]<br>--	2.35	2.15	1.97
[**1**(CNMe)(THF)]$^+$<br>(calculated)	2.5082[a]<br>2.489[b]<br>2.594[b]<br>2.407[c]<br>2.458[c]	1.966[a]<br>2.391[b]<br>--	2.37	2.18	1.97
[**1**(THF)$_2$][PF$_6$]<br>(crystallographic)	2.4441(8)[b]<br>2.7294(8)[b]<br>2.5172(7)[b]<br>2.4410(7)[c]<br>2.4566(7)[c]	2.201(4)[b]<br>2.081(3)[b]<br>--	2.36	2.18	1.97
[**1**(THF)$_2$]$^+$<br>(calculated)	2.422[b]<br>3.197[b]<br>2.423[b]<br>3.198[b]<br>2.425[c]<br>2.425[c]	2.182[b]<br>2.182[b]<br>--	2.37	2.18	1.97
[**1**(CNMe)(THF)$_2$]$^+$<br>(calculated)	2.479[b]<br>2.582[b]<br>2.504[a]<br>2.485[b]<br>2.515[b]	2.418[b]<br>1.969[a]<br>2.666[b]	2.37	2.17	1.98

[a]Fe(CNMe/CN^{t}Bu) edge site; [b]Fe(THF) edge site; [c]κ^4-Fe edge site.

Table S12. Total energies calculated for *cis*-(α,β)-[**1**$(CNMe)_2$]$^+$, [**1**$(CNMe)_2$(THF)]$^+$, [**1**(CNMe)(THF)]$^+$, [**1**(CNMe)$(THF)_2$]$^+$, [**1**$(THF)_2$]$^+$, *trans*-(α,β)-[**1**$(CNMe)_2$]$^+$, and (α,α)-[**1**$(CNMe)_2$]$^+$ at the B3LYP+/cc-pVTZ level of theory.

**Compound**	**Total Energy (eV)**
cis-(α,β)-[**1**$(CNMe)_2$]$^+$	−1034471.565
[**1**$(CNMe)_2$(THF)]$^+$	−1040756.003
[**1**(CNMe)(THF)]$^+$	−1037167.030
[a][**1**(CNMe)$(THF)_2$]$^+$	−1043452.074
[**1**$(THF)_2$]$^+$	−1039862.882
trans-(α,β)-[**1**$(CNMe)_2$]$^+$	−1034472.083
(α,α)-[**1**$(CNMe)_2$]$^+$	−1034472.081
cis-(α,β)-**1**$(CNMe)_2$	−1034476.493

[a]Global minimum not found for [**1**(CNMe)$(THF)_2$]$^+$.

Table S13. Calculated ν_{CN} frequencies for *cis*-(α,β)-[**1**(CNMe)$_2$]$^+$, [**1**(CNMe)$_2$(THF)]$^+$, [**1**(CNMe)(THF)]$^+$, [**1**(CNMe)(THF)$_2$]$^+$, *trans*-(α,β)-[**1**(CNMe)$_2$]$^+$, and (α,α)-[**1**(CNMe)$_2$]$^+$ at the BPV86/def2SVP level of theory.

**Compound**	**ν_{CN} (cm^{-1})**
cis-(α,β)-[**1**(CNMe)$_2$]$^+$	2175.72 (major); 2171.80 (minor)
(α,α,β)-[**1**(CNMe)$_2$(THF)]$^+$	2173.58 (major); 2168.92 (minor)
trans-(α,β)-[**1**(CNMe)(THF)]$^+$	2178.93
[a][**1**(CNMe)(THF)$_2$]$^+$	2179.05
trans-(α,β)-[**1**(CNMe)$_2$]$^+$	2167.49 (major); 2178.80 (minor)
(α,α)-[**1**(CNMe)$_2$]$^+$	2172.64 (major); 2178.84 (minor)
cis-(α,β)-**1**(CNMe)$_2$	2157.73 (major); 2149.08 (minor)

[a]Global minimum not found for [**1**(CNMe)(THF)$_2$]$^+$.

Table S14. Hirshfeld spin-density values calculated for [**1**(CNMe)$_2$]$^+$, [**1**(CNMe)$_2$(THF)]$^+$, [**1**(CNMe)(THF)]$^+$, [**1**(CNMe)(THF)$_2$]$^+$, and [**1**(THF)$_2$]$^+$ at the B3LYP+/cc-pVTZ level of theory.

**Compound**	**Hirshfeld Spin-density**						
	Fe1	Fe2	Fe3	Co_6Se_8 core	Total Spin	% on Fe edges	% on Co_6Se_8
cis-(α,β)-[**1**(CNMe)$_2$]$^+$	3.37[a]	3.37[a]	3.65[c]	1.15	13	79.9	8.8
[**1**(CNMe)$_2$(THF)]$^+$	3.37[a]	3.37[a]	3.70[b]	1.06	13	80.3	8.2
[**1**(CNMe)(THF)]$^+$	3.34[a]	3.48[b]	3.59[c]	1.07	13	80.1	8.2
[**1**(CNMe)(THF)$_2$]$^+$	3.35[a]	3.46[b]	3.67[b]	1.08	13	80.6	8.3
cis-(α,β)-[**1**(THF)$_2$]$^+$	3.45[b]	3.45[b]	3.59[c]	1.15	13	80.7	8.8
cis-(α,β)-**1**(CNMe)$_2$	3.36[a]	3.36[a]	3.31[c]	0.71	12	83.7	6.0

[a]Fe(CNMe) edge site; [b]Fe(THF) edge site; [c]κ^4-Fe edge site.

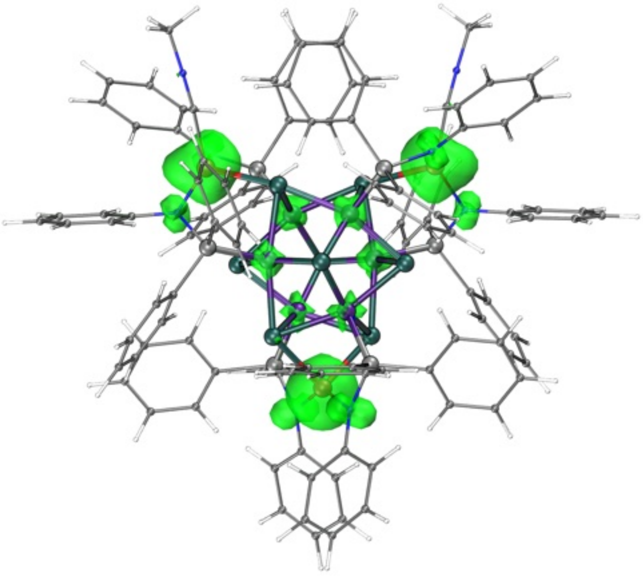

Figure S66. **Mulliken spin density (α−β; 0.008 a.u.) plot** for *cis*-(α,β)-[**1**$(CNMe)_2]^+$ calculated at B3LYP+/cc-pVTZ. All other compounds calculated herein exhibit comparable spin density distributions. Spin density contributions of the edge Fe atoms and Co_6Se_8 core for *cis*-(α,β)-[**1**$(CNMe)_2]^+$ and all other calculated structures are tabulated in Table S14.

Table S15. Hirshfeld charges calculated for [**1**$(CNMe)_2]^+$, [**1**$(CNMe)_2(THF)]^+$, [**1**$(CNMe)(THF)]^+$, [**1**$(CNMe)(THF)_2]^+$, and [**1**$(THF)_2]^+$ at the B3LYP+/cc-pVTZ level of theory.

Compound	Hirshfeld Charge (a.u.)		
	Fe1	Fe2	Fe3
cis-(α,β)-[**1**$(CNMe)_2]^+$	+0.25[c]	+0.19[a]	+0.19[a]
[**1**$(CNMe)_2(THF)]^+$	+0.29[b]	+0.19[a]	+0.19[a]
[**1**$(CNMe)(THF)]^+$	+0.22[b]	+0.25[c]	+0.19[a]
[**1**$(CNMe)(THF)_2]^+$	+0.22[b]	+0.29[b]	+0.19[a]
[**1**$(THF)_2]^+$	+0.22[b]	+0.22[b]	+0.25[c]
cis-(α,β)-**1**$(CNMe)_2$	+0.19[a]	+0.19[a]	+0.16[c]

[a]Fe(CNMe) edge site; [b]Fe(THF) edge site; [c]κ^4-Fe edge site.

S7.1 DFT Input Files

Geometry Optimization input files:

1. *cis*-(α,β)-[**1**$(CNMe)_2]^+$

```
%mem=115GB
%LindaWorker=
```

```
%usessh
%nprocshared=28
%chk=Fe3CNMe2+_cis_ab_ubpv86_def2SVP_opt.chk
#p opt ubvp86 pop=(full,nbo) def2svp gfinput scf=(xqc,maxconventional=256)

Fe3CNMe2+_cis_ab_ubpv86_def2SVP_opt

1 14
 C                 -4.30397156    1.11291904   -5.93978289
 C                 -1.33364484   -3.50405616   -6.72912193
 C                 -3.06786910    1.75766648   -6.10139696
 C                 -2.65243997   -3.36044473   -6.27142145
 C                  2.07340822    2.07211837   -6.78244702
 C                 -4.87278149    1.02320617   -4.65803461
 C                  1.50617743    0.83032398   -6.44495433
 C                 -0.27935300   -3.55873424   -5.80011608
 C                  2.95216243    2.69987043   -5.88401767
 C                 -2.40167981    2.30742895   -4.99286241
 C                 -2.92077802   -3.27244735   -4.89418829
 C                 -5.49692234    5.77677434   -3.44416665
 C                 -4.20790428    1.56799832   -3.54869317
 C                 -6.58250163    6.06730948   -2.60192649
 C                  1.81757127    0.22014714   -5.21894740
 C                 -2.96372137    2.21916391   -3.70262257
 C                 -0.54334794   -3.45913374   -4.42630330
 C                 -4.45603481    4.93530832   -3.02332308
 C                 -1.86849264   -3.31608378   -3.95652571
 C                 -6.15305212   -2.28144123   -2.14809251
 C                  3.26671888    2.08956937   -4.65635412
 C                 -2.42755911   -7.68160002   -3.55786592
 C                 -1.40658169   -8.58132264   -3.20954295
 C                 -4.76428029   -2.23919165   -2.35121163
 C                  0.75284677    5.26289229   -3.95065372
 C                  2.71165864    0.83940412   -4.31641189
 C                  0.46295988    6.54172731   -3.44464794
 C                 -0.02145783    4.15668262   -3.56572705
 C                 -6.60944226    5.48811043   -1.32075750
 C                 -6.73649787   -3.36012482   -1.46003670
 C                  4.97854474   -3.02818825   -4.85380970
 C                 -2.47208949   -6.39115458   -3.00827685
 C                  4.28174348   -1.84843771   -4.54082447
 C                 -4.46190069    4.34263419   -1.72845404
 C                 -0.59541734    6.70364575   -2.53577438
 C                 -1.12217571    4.32067730   -2.69459405
 C                 -0.41516999   -8.16436573   -2.30265764
 C                 -3.94292868   -3.28693299   -1.87664758
 C                 -1.38163965    5.60116369   -2.16167278
 C                 -5.57717434    4.64260419   -0.89518897
 C                  7.72356293    0.63797127   -3.45782519
 C                 -1.47921485   -5.95680536   -2.08950283
 C                  6.33204400    0.78405381   -3.36136654
```

C	-0.44486234	-6.87469378	-1.75928527
C	5.43919863	-3.87043636	-3.82980751
C	-5.92134900	-4.39179791	-0.96513154
C	4.02634550	-1.50153425	-3.19661430
C	-4.53051938	-4.35343375	-1.16536584
C	8.52053705	0.55439685	-2.30247147
C	5.69334515	0.84628785	-2.09413467
C	5.19924255	-3.52733040	-2.48837607
C	4.49273232	-2.35745589	-2.17349521
C	7.90228456	0.60574252	-1.04130051
C	-7.71430879	1.83386191	1.03753188
C	6.51014008	0.73889533	-0.93780350
C	-6.31843366	1.74771633	0.93486989
C	-8.33450008	1.86719518	2.29836701
C	-5.49601014	1.71413577	2.09182872
C	-0.61975922	-6.87189454	1.74446906
C	-0.84795747	-8.14111458	2.28884829
C	-4.81334927	-1.63404339	2.16535250
C	-7.53553234	1.81291734	3.45409739
C	-5.69202973	-2.68112215	2.47930228
C	-6.13820288	1.74008128	3.35865269
C	6.23205976	3.70950204	0.89857988
C	0.53666553	-6.11827745	2.08530155
C	3.79895761	-5.01161807	1.16931394
C	5.16714123	-5.26474566	0.96927463
C	7.38527426	4.38010924	1.32519058
C	2.24429167	5.30862832	2.19742487
C	1.64070472	6.51668918	2.58464671
C	-4.21315522	-0.86761180	3.18980085
C	5.08515017	3.58441631	1.73352950
C	0.06030068	-8.69970535	3.20663020
C	3.38309535	-3.86371964	1.87526588
C	1.78522567	4.07861762	2.71470867
C	0.56847089	6.51285121	3.49168793
C	6.13198737	-4.36917040	1.45992825
C	-5.97496635	-2.99080921	3.82046196
C	0.67056408	4.07996908	3.58361736
C	0.07976539	5.28972520	3.98220673
C	1.44368917	-6.69424974	3.01494979
C	4.35670942	-2.95365820	2.34596255
C	5.72226474	-3.21088033	2.14381231
C	7.45229503	4.94982484	2.60914096
C	-2.55343225	1.23987389	4.31517490
C	-2.91309477	2.55833832	4.66034408
C	1.20086902	-7.96224389	3.56497239
C	5.17536807	4.16347684	3.03147805
C	-4.51177095	-1.17888972	4.53384804
C	-5.38244013	-2.23715862	4.84562534
C	6.33638293	4.82892336	3.45321241
C	1.32424244	-3.56381853	3.95110489
C	3.27866542	1.70818934	3.69971862

C	-0.00951323	-3.51317903	4.41540183
C	4.41610278	0.88574610	3.54011442
C	-2.50950535	3.10950549	5.88993784
C	-1.76296420	0.48938355	5.21490102
C	2.72890495	1.85912104	4.98948108
C	2.36859256	-3.66380974	4.89325922
C	-0.29026598	-3.56383565	5.78855340
C	4.99063415	0.23645826	4.64373579
C	-1.73517927	2.35304663	6.78527277
C	-1.36231621	1.04107341	6.44273491
C	3.30371399	1.20441870	6.09210805
C	2.08514248	-3.70234903	6.26978047
C	0.75763110	-3.65303082	6.72216680
C	4.43490938	0.39057803	5.92504515
H	-4.82625919	0.68699136	-6.81018744
H	-1.12664834	-3.58058139	-7.80788553
H	-3.48668500	-3.32483663	-6.98920509
H	1.82996838	2.54899599	-7.74456582
H	-2.61314261	1.83958443	-7.10075994
H	0.81195473	0.33041591	-7.13762746
H	-5.85035268	0.53537164	-4.52128424
H	-5.45087175	6.21264584	-4.45489973
H	0.75871765	-3.68367542	-6.14493925
H	3.40586853	3.66963178	-6.14306189
H	-3.96257161	-3.18193791	-4.55508583
H	-1.44304704	2.81910453	-5.15096507
H	-6.78488923	-1.46242596	-2.52499678
H	-3.62572240	4.74147673	-3.71363470
H	-3.21176266	-7.98655836	-4.26892411
H	-4.31870040	-1.38215655	-2.87982255
H	1.35782909	-0.74749191	-4.96694561
H	-4.66430448	1.50334117	-2.55002635
H	1.59376918	5.11275502	-4.64428334
H	5.16773414	-3.28325407	-5.90825599
H	3.94496695	-1.19187642	-5.35553052
H	1.06292631	7.41137540	-3.75677126
H	0.25620566	3.15840511	-3.93619931
H	0.28997500	-3.50037835	-3.70656230
H	3.95005497	2.58151301	-3.94804652
H	-7.82585903	-3.39078967	-1.30379493
H	-7.45362819	5.68439275	-0.64056799
H	8.19195904	0.59346928	-4.45387936
H	-3.28884570	-5.71393433	-3.29182861
H	0.39919125	-8.84897064	-2.01818831
H	-0.82095663	7.69807024	-2.11990606
H	5.72703813	0.85935549	-4.27575254
H	-2.21208301	5.73250379	-1.45397492
H	5.98605786	-4.79379563	-4.07604646
H	-5.63283538	4.18313360	0.10264285
H	0.34662886	-6.54954526	-1.06478056
H	-6.36850399	-5.23846441	-0.42129263

H	-8.32244542	1.86268344	0.11983049
H	-3.89019245	-5.15656242	-0.77222842
H	-5.83171114	1.68530553	-0.05011232
H	8.50714188	0.52928502	-0.12417178
H	5.55432278	-4.17503124	-1.67324967
H	4.30674806	-2.10522017	-1.11794183
H	6.01900554	0.74429362	0.04695678
H	-1.34614578	-6.43422193	1.04082466
H	-1.75260019	-8.69676990	1.99617082
H	-4.59738435	-1.40784914	1.10951533
H	6.21279755	3.25167169	-0.10137049
H	-6.14865360	-3.26055226	1.66338768
H	3.04297483	-5.70937071	0.78043825
H	8.24851735	4.44457468	0.64365956
H	5.47803352	-6.17256580	0.42912229
H	3.08699730	5.31700583	1.49212633
H	2.02103402	7.46799324	2.18083410
H	-8.00622910	1.83236154	4.44987453
H	-5.53034544	1.71068426	4.27374798
H	-3.51468750	3.15108054	3.95508165
H	0.11273004	7.46245984	3.81458118
H	7.20347875	-4.56819981	1.30388315
H	-6.65802623	-3.81885236	4.06551402
H	-0.83729945	-3.44041718	3.69184388
H	0.23761555	3.13359328	3.94132289
H	4.86238749	0.76842005	2.54173097
H	-0.77552499	5.26561613	4.67409406
H	4.04947903	-2.03548175	2.87033858
H	6.47299316	-2.49767414	2.51769990
H	2.34928959	-6.14602184	3.30719200
H	4.32652288	4.09849008	3.72349374
H	-4.07210938	-0.58788037	5.34968036
H	1.92315797	-8.37941156	4.28458688
H	-2.81164415	4.13564661	6.15287813
H	-1.45491294	-0.53586686	4.95963395
H	-5.60307799	-2.46576245	5.90003009
H	6.36162654	5.26126202	4.46619164
H	1.85248702	2.50035806	5.15212656
H	3.41387606	-3.72525348	4.55844332
H	-1.33685245	-3.53701132	6.12917903
H	5.88831527	-0.38534332	4.50283898
H	-1.42203967	2.78419982	7.74880278
H	-0.75168196	0.43915820	7.13300282
H	2.86065462	1.33742245	7.09119517
H	2.91322395	-3.78061714	6.99129936
H	0.53781841	-3.69129060	7.80043699
H	4.88643304	-0.11741005	6.79100662
N	-3.44596847	3.50663315	-1.22255030
N	-1.46788113	-4.68975478	-1.48180719
N	4.30228684	1.04067382	-1.93204885
N	-4.09182900	1.68938796	1.93009534

N	0.72098335	-4.86456842	1.47790351
N	3.94952485	2.92099999	1.22634646
P	-2.15785953	2.87206893	-2.15117278
P	-2.10722834	-3.23731152	-2.11237255
P	3.12152301	0.03840009	-2.68566319
P	-3.08084132	0.51527210	2.68229256
P	1.57704897	-3.52823681	2.10746941
P	2.58020973	2.48997126	2.15555624
Fe	-3.25494006	3.02985993	0.72864428
Fe	3.68237440	2.48990131	-0.72621400
Fe	-0.31574788	-4.07419219	-0.00258577
Co	-0.99250419	1.41999547	-1.05024710
Co	-1.08084624	-1.66468360	-1.03154868
Co	1.41983368	-0.19298047	-1.35728181
Co	-1.43453552	0.02686148	1.35363321
Co	1.19899871	1.24449831	1.05095149
Co	0.80740090	-1.81660229	1.02331109
Se	-0.46444511	-0.14218794	-2.72897095
Se	-2.73003513	-0.03340915	-0.57142401
Se	1.29721046	2.13992612	-1.15006617
Se	1.14732467	-2.57623805	-1.25202216
Se	-0.95191508	2.31283416	1.15354152
Se	-1.53813296	-2.36876022	1.23887703
Se	2.68908210	-0.46057251	0.56719739
Se	0.43371044	-0.22075581	2.72420432
H	-1.38020537	-9.59301217	-3.64177790
H	-0.12184757	-9.69497176	3.63941928
H	9.61236989	0.44270491	-2.38512020
H	8.36049914	5.47093328	2.94787281
H	-9.43042658	1.92730375	2.38040273
H	-7.39662596	6.72619188	-2.93987294
C	4.02852373	4.25887884	-1.52545479
C	-3.31756422	4.82646681	1.53797768
C	4.52672818	6.71800837	-2.19270239
H	3.58454296	7.29990834	-2.26217918
H	5.18195689	7.18122187	-1.42621558
H	5.04433661	6.76605269	-3.17248733
C	-3.40336883	7.32728511	2.23064022
H	-2.37668210	7.73478467	2.33603102
H	-3.94466218	7.90716717	1.45472783
H	-3.93440367	7.45289359	3.19628144
N	-3.35583623	5.96242840	1.87753922
N	4.25092646	5.37636962	-1.85545427

$NBO PLOT FILE=Fe3CNMe2+_cis_ab_ubpv86_def2SVP_opt $END

2. (α,α,β)-[**1**$(CNMe)_2$(THF)]$^+$

%mem=243GB
%LindaWorker=
%usessh

```
%nprocshared=28
%chk=Fe3CNMe2THF+_aab_1_14_ubpv86_def2SVP_opt.chk
#p ubvp86 def2svp gfinput scf=(xqc,maxconventional=256) guess=(fragment=4,only)

Fe3CNMe2THF+_aab_1_14_ubpv86_def2SVP_opt

1 14 0 5 0 5 0 5 1 2
 C(Fragment=1)    -0.92763192    4.08945428    6.18539564
 C(Fragment=2)     3.22923536   -0.00600983    6.94855825
 C(Fragment=1)    -1.77211983    2.96865291    6.21195436
 C(Fragment=2)     3.41545491    1.34411768    6.61495676
 C(Fragment=3)    -2.88891457   -2.09795267    6.56514689
 C(Fragment=1)    -0.64692446    4.71227020    4.95761912
 C(Fragment=3)    -1.53548610   -1.78955712    6.33879476
 C(Fragment=2)     3.05687550   -0.95418262    5.92464657
 C(Fragment=3)    -3.62676156   -2.75368217    5.56584815
 C(Fragment=1)    -2.33112521    2.47079940    5.02274229
 C(Fragment=2)     3.43182800    1.74755315    5.26829831
 C(Fragment=1)    -5.11267128    6.19734658    3.46408139
 C(Fragment=1)    -1.20058409    4.21469826    3.76778242
 C(Fragment=1)    -5.15816929    7.36170502    2.68017989
 C(Fragment=3)    -0.92469963   -2.13613744    5.12294888
 C(Fragment=1)    -2.05219680    3.08787433    3.78576991
 C(Fragment=2)     3.05788603   -0.55139963    4.58111741
 C(Fragment=1)    -4.42091540    5.05613754    3.03087920
 C(Fragment=2)     3.24684327    0.80582778    4.23438553
 C(Fragment=2)     2.99846645    5.36624466    2.95692992
 C(Fragment=3)    -3.01557860   -3.10474585    4.34825083
 C(Fragment=2)     7.50746430    0.28906774    4.25652706
 C(Fragment=2)     7.96778712   -1.02053709    4.03245278
 C(Fragment=2)     2.71384665    3.99145646    2.95831130
 C(Fragment=1)    -5.66971406   -0.07105404    3.56528236
 C(Fragment=3)    -1.65681820   -2.80965998    4.11906252
 C(Fragment=1)    -6.84301284    0.46344621    3.00568407
 C(Fragment=1)    -4.42584637    0.52644843    3.30652166
 C(Fragment=1)    -4.48485502    7.36358646    1.44545888
 C(Fragment=2)     4.21125839    5.84024301    2.42542758
 C(Fragment=3)     1.61615788   -5.85908236    4.66221861
 C(Fragment=2)     6.41322323    0.79919875    3.54275216
 C(Fragment=3)     0.60800184   -4.93473498    4.33862562
 C(Fragment=1)    -3.73752214    5.03492583    1.78137055
 C(Fragment=1)    -6.76131496    1.58863905    2.16929510
 C(Fragment=1)    -4.34213413    1.69258528    2.51249644
 C(Fragment=2)     7.30438317   -1.82485020    3.08969923
 C(Fragment=2)     3.64947999    3.06842235    2.43850611
 C(Fragment=1)    -5.51983095    2.19823196    1.92257341
 C(Fragment=1)    -3.78792943    6.23025310    1.00820556
 C(Fragment=3)    -2.38052278   -7.70509110    2.93288492
 C(Fragment=2)     5.73989365    0.00274279    2.57816502
 C(Fragment=3)    -2.26644138   -6.30716796    2.92175484
 C(Fragment=2)     6.20092102   -1.32693696    2.38200910
```

C(Fragment=3)	2.48874663	-6.32995539	3.66881360
C(Fragment=2)	5.13715646	4.92898329	1.89154314
C(Fragment=3)	0.46820846	-4.45852614	3.01690553
C(Fragment=2)	4.85677440	3.55058404	1.89462139
C(Fragment=3)	-2.31160942	-8.44204689	1.73745374
C(Fragment=3)	-2.07450683	-5.60271455	1.70353552
C(Fragment=3)	2.34867942	-5.87122909	2.34788424
C(Fragment=3)	1.35234463	-4.93834931	2.02512505
C(Fragment=3)	-2.11241335	-7.75995657	0.52482535
C(Fragment=1)	-0.53096097	8.00942882	-0.58746318
C(Fragment=3)	-1.98546238	-6.36334615	0.50815825
C(Fragment=1)	-0.67968241	6.61505736	-0.58009059
C(Fragment=1)	-0.38471406	8.70344891	-1.80099468
C(Fragment=1)	-0.70817225	5.87139748	-1.78943537
C(Fragment=2)	7.17578209	-1.70141376	-1.10467208
C(Fragment=2)	8.44353753	-2.10511784	-1.54225811
C(Fragment=1)	2.50935727	4.59035307	-1.61201571
C(Fragment=1)	-0.38903196	7.97956846	-3.00638049
C(Fragment=1)	3.69768080	5.32244286	-1.75214747
C(Fragment=1)	-0.54992551	6.58620259	-3.00686210
C(Fragment=3)	-4.72517703	-5.41005160	-1.51005769
C(Fragment=2)	5.99574106	-1.99128531	-1.84973234
C(Fragment=2)	4.16989496	-4.56030916	-1.18149357
C(Fragment=2)	4.12060580	-5.96186223	-1.09012007
C(Fragment=3)	-5.55954549	-6.39390746	-2.05538909
C(Fragment=3)	-5.52128333	-1.08203704	-2.55849018
C(Fragment=3)	-6.57085248	-0.23956844	-2.96143948
C(Fragment=1)	1.73377620	4.25373800	-2.74373762
C(Fragment=3)	-4.37740930	-4.23419399	-2.23478327
C(Fragment=2)	8.59404273	-2.82798906	-2.73887448
C(Fragment=2)	3.19105731	-3.85506366	-1.91386252
C(Fragment=3)	-4.20067014	-0.84087724	-2.99260786
C(Fragment=3)	-6.31486547	0.85734257	-3.80043066
C(Fragment=2)	3.08693511	-6.67865218	-1.71506412
C(Fragment=1)	4.13946535	5.71754602	-3.02747447
C(Fragment=3)	-3.94317183	0.29887602	-3.78787494
C(Fragment=3)	-4.99606213	1.12955342	-4.20350780
C(Fragment=2)	6.16910625	-2.73503331	-3.05093932
C(Fragment=2)	2.13432494	-4.58110601	-2.50984361
C(Fragment=2)	2.09003030	-5.98183523	-2.41845069
C(Fragment=3)	-6.07777062	-6.25699174	-3.35552225
C(Fragment=1)	-0.49344908	3.04734060	-4.18472528
C(Fragment=1)	-1.71899367	3.60899132	-4.59618907
C(Fragment=2)	7.44239863	-3.13968816	-3.47975688
C(Fragment=3)	-4.90619408	-4.11898337	-3.55217431
C(Fragment=1)	2.18252670	4.65814444	-4.01932649
C(Fragment=1)	3.38082618	5.37943292	-4.15987093
C(Fragment=3)	-5.73621904	-5.11216259	-4.09382158
C(Fragment=2)	3.29600785	-1.54889170	-3.79156131
C(Fragment=3)	-2.10690655	-2.70302964	-3.97008993
C(Fragment=2)	3.73533826	-0.24096881	-4.09715025

C(Fragment=3)	-1.52190823	-3.98348022	-3.85292484
C(Fragment=1)	-2.21280258	3.37192258	-5.89189521
C(Fragment=1)	0.21949963	2.22382139	-5.08530117
C(Fragment=3)	-2.09061749	-2.06571859	-5.22784531
C(Fragment=2)	2.92165520	-2.39821723	-4.85324263
C(Fragment=2)	3.79565033	0.20500992	-5.42582870
C(Fragment=3)	-0.94391390	-4.61179965	-4.96701990
C(Fragment=1)	-1.49090425	2.56595105	-6.78775604
C(Fragment=1)	-0.27348977	1.99215551	-6.37946918
C(Fragment=3)	-1.50770707	-2.69483439	-6.34097672
C(Fragment=2)	2.98101867	-1.94945123	-6.18424791
C(Fragment=2)	3.41607305	-0.64778039	-6.47764151
C(Fragment=3)	-0.93282023	-3.96935606	-6.21671331
H(Fragment=1)	-0.49384782	4.48047998	7.11860507
H(Fragment=2)	3.22479273	-0.32138113	8.00358062
H(Fragment=2)	3.55866188	2.09556374	7.40708504
H(Fragment=3)	-3.36670610	-1.82669340	7.51938794
H(Fragment=1)	-2.00415198	2.47306140	7.16733610
H(Fragment=3)	-0.94785024	-1.27048296	7.11142921
H(Fragment=1)	-0.00012891	5.60281605	4.92621757
H(Fragment=1)	-5.62651811	6.16789115	4.43816506
H(Fragment=2)	2.92296578	-2.01904660	6.17010427
H(Fragment=3)	-4.68566334	-3.00438550	5.73720628
H(Fragment=2)	3.59814297	2.80730487	5.02883261
H(Fragment=1)	-2.99919883	1.60097406	5.07483973
H(Fragment=2)	2.26432189	6.07504578	3.37003500
H(Fragment=1)	-4.41699409	4.16835673	3.67521784
H(Fragment=2)	8.01178551	0.93017026	4.99700653
H(Fragment=2)	1.75623449	3.63574265	3.36938508
H(Fragment=3)	0.13281273	-1.87904609	4.95875016
H(Fragment=1)	-0.98277972	4.71248325	2.81147808
H(Fragment=1)	-5.71128638	-0.96777659	4.20165265
H(Fragment=3)	1.71196761	-6.21697139	5.69929512
H(Fragment=3)	-0.07597220	-4.58838392	5.12635808
H(Fragment=1)	-7.82027537	0.00149812	3.21849298
H(Fragment=1)	-3.51582513	0.05960935	3.71283088
H(Fragment=2)	2.92012869	-1.30450358	3.78907744
H(Fragment=3)	-3.59182427	-3.61466535	3.56170496
H(Fragment=1)	-4.48810087	8.26559490	0.81278756
H(Fragment=3)	-2.53052748	-8.22546369	3.89222616
H(Fragment=2)	6.07721123	1.82979108	3.72372170
H(Fragment=2)	7.63736520	-2.85954581	2.91201424
H(Fragment=1)	-7.67196826	2.00484217	1.71078765
H(Fragment=3)	-2.33581712	-5.74554272	3.86394839
H(Fragment=1)	-5.46081636	3.08153914	1.27151308
H(Fragment=3)	3.27756125	-7.05504988	3.92267108
H(Fragment=1)	-3.24981993	6.26520626	0.04926687
H(Fragment=2)	5.65176664	-1.96870637	1.67552528
H(Fragment=2)	6.09036510	5.29046050	1.47439676
H(Fragment=1)	-0.51763961	8.55593679	0.36859746
H(Fragment=2)	5.57748984	2.83385992	1.47372032

H(Fragment=1)	-0.75826622	6.06483954	0.36972378
H(Fragment=3)	-2.04221164	-8.32029421	-0.42068324
H(Fragment=3)	3.02214716	-6.23132638	1.55609060
H(Fragment=3)	1.26188206	-4.58102151	0.98782481
H(Fragment=3)	-1.79385717	-5.82864996	-0.43430938
H(Fragment=2)	7.08210740	-1.16373347	-0.14896888
H(Fragment=2)	9.32387119	-1.86031218	-0.92667571
H(Fragment=1)	2.17744672	4.28324954	-0.60819760
H(Fragment=3)	-4.31083332	-5.55361341	-0.50092019
H(Fragment=1)	4.27625442	5.58171066	-0.85267520
H(Fragment=2)	4.97977390	-4.01027352	-0.68368135
H(Fragment=3)	-5.79394080	-7.28774038	-1.45541237
H(Fragment=2)	4.90407573	-6.49521734	-0.52944468
H(Fragment=3)	-5.72521718	-1.94280827	-1.90615936
H(Fragment=3)	-7.59758351	-0.45116746	-2.62412014
H(Fragment=1)	-0.27112006	8.50786130	-3.96590540
H(Fragment=1)	-0.56550357	6.03906544	-3.95993238
H(Fragment=1)	-2.28446084	4.23459123	-3.88923809
H(Fragment=3)	-7.14264464	1.50107605	-4.13839834
H(Fragment=2)	3.05021226	-7.77666973	-1.64425018
H(Fragment=1)	5.06795777	6.30033903	-3.13722183
H(Fragment=2)	4.03757965	0.43364904	-3.28153443
H(Fragment=3)	-2.91280605	0.56077985	-4.07234163
H(Fragment=3)	-1.53642002	-4.49731436	-2.88059565
H(Fragment=3)	-4.77351164	2.00009054	-4.83885079
H(Fragment=2)	1.32338169	-4.05331714	-3.03340086
H(Fragment=2)	1.26188229	-6.53174025	-2.89148555
H(Fragment=2)	5.30012158	-2.99896181	-3.66477626
H(Fragment=3)	-4.67081379	-3.24002753	-4.16492480
H(Fragment=1)	1.59038983	4.41939472	-4.91431153
H(Fragment=2)	7.52828415	-3.71203454	-4.41726890
H(Fragment=1)	-3.16518123	3.82839502	-6.20499092
H(Fragment=1)	1.16702132	1.75511008	-4.77815803
H(Fragment=1)	3.71249747	5.69068276	-5.16291825
H(Fragment=3)	-6.12204694	-4.98062518	-5.11735709
H(Fragment=3)	-2.54596063	-1.07514913	-5.35777751
H(Fragment=2)	2.59463100	-3.42778823	-4.65501962
H(Fragment=2)	4.14651564	1.22664951	-5.64008727
H(Fragment=3)	-0.51127121	-5.61869571	-4.86046323
H(Fragment=1)	-1.87660156	2.38269502	-7.80270330
H(Fragment=1)	0.29749511	1.35098711	-7.06831127
H(Fragment=3)	-1.50949082	-2.18083129	-7.31464672
H(Fragment=2)	2.68617359	-2.63113404	-6.99697754
H(Fragment=2)	3.46739068	-0.30069550	-7.52139929
H(Fragment=3)	-0.48471232	-4.46487901	-7.09182918
N(Fragment=1)	-3.04119334	3.92516460	1.26303302
N(Fragment=2)	4.66795587	0.47037664	1.78815675
N(Fragment=3)	-2.00561555	-4.19217665	1.62607624
N(Fragment=1)	-0.92231961	4.47538650	-1.72890556
N(Fragment=2)	4.75932077	-1.52910574	-1.34928370
N(Fragment=3)	-3.55968218	-3.27277520	-1.60692101

P(Fragment=1)	-2.70283366	2.49312993	2.13980103
P(Fragment=2)	3.27206709	1.24713798	2.42446383
P(Fragment=3)	-0.85217931	-3.25725351	2.49953039
P(Fragment=1)	0.14169712	3.33318825	-2.45882971
P(Fragment=2)	3.22782210	-1.99887162	-1.97290469
P(Fragment=3)	-2.82343589	-1.95427562	-2.41621162
Fe(Fragment=1)	-2.44369318	3.78680383	-0.65848613
Fe(Fragment=3)	-3.23337714	-3.25517122	0.38660414
Fe(Fragment=2)	4.72119122	0.09322532	-0.15760492
Co(Fragment=4)	-1.38550248	1.15638117	1.07377835
Co(Fragment=4)	1.53116125	0.71274136	1.26786128
Co(Fragment=4)	-0.23737745	-1.55702514	1.31670751
Co(Fragment=4)	0.26672129	1.55327497	-1.25013329
Co(Fragment=4)	-1.41157685	-0.88761259	-1.18108332
Co(Fragment=4)	1.55514676	-1.06112419	-0.96574272
Se(Fragment=4)	-0.12997290	0.23407379	2.83891714
Se(Fragment=4)	0.33883665	2.68546296	0.81273005
Se(Fragment=4)	-2.49330965	-0.97871530	0.93567393
Se(Fragment=4)	2.10731255	-1.55867941	1.25966229
Se(Fragment=4)	-2.06441457	1.42343614	-1.18638360
Se(Fragment=4)	2.56205132	1.07301333	-0.83649913
Se(Fragment=4)	-0.08080483	-2.71738305	-0.70582779
Se(Fragment=4)	0.23215807	-0.26467649	-2.74817334
H(Fragment=3)	-6.72739264	-7.03290545	-3.78813383
H(Fragment=3)	-2.40479450	-9.53867916	1.75245482
H(Fragment=2)	8.82925302	-1.41278253	4.59405188
H(Fragment=2)	4.43030376	6.91923653	2.42496977
H(Fragment=1)	-0.26170079	9.79721288	-1.80771034
H(Fragment=1)	-5.70060568	8.25389744	3.02792735
H(Fragment=2)	9.58878028	-3.15156948	-3.08092623
C(Fragment=1)	-6.56830687	4.74620379	-2.33391067
H(Fragment=1)	-7.15548576	3.81982085	-2.50085412
H(Fragment=1)	-7.06557897	5.35427097	-1.55023820
H(Fragment=1)	-6.55357130	5.33047487	-3.27647422
C(Fragment=3)	-7.61768774	-3.48237080	1.58874233
H(Fragment=3)	-8.04869960	-2.46616832	1.70257283
H(Fragment=3)	-8.13506942	-4.00240786	0.75597455
H(Fragment=3)	-7.80054611	-4.04987909	2.52405017
N(Fragment=3)	-6.23302483	-3.40328226	1.33259421
C(Fragment=3)	-5.07780797	-3.34515800	1.06784687
C(Fragment=1)	-4.15796924	4.16635855	-1.56335139
N(Fragment=1)	-5.25143746	4.42664416	-1.94191430
O(Fragment=2)	5.87584616	1.25115525	-0.95658568
C(Fragment=2)	6.71553260	2.17568260	-0.25935038
C(Fragment=2)	6.07007042	1.36347114	-2.36925075
C(Fragment=2)	7.53287816	2.96628725	-1.27541746
H(Fragment=2)	6.04051958	2.83421667	0.34537663
H(Fragment=2)	7.36305232	1.57162423	0.42682464
C(Fragment=2)	7.11489293	2.44031915	-2.64173922
H(Fragment=2)	6.39689098	0.35544945	-2.73268503
H(Fragment=2)	5.07402873	1.61835645	-2.81415333

```
 H(Fragment=2)      8.62712959    2.81606615   -1.10513422
 H(Fragment=2)      7.32371407    4.06040713   -1.18540400
 H(Fragment=2)      7.98879759    2.01282676   -3.19172936
 H(Fragment=2)      6.68538090    3.25716644   -3.27200011

--link1--
%mem=243GB
%LindaWorker=
%usessh
%nprocshared=28
%chk=Fe3CNMe2THF+_aab_1_14_ubpv86_def2SVP_opt.chk
#p opt ubvp86 pop=(full,nbo) chkbasis gfinput scf=(xqc,maxconventional=256) geom=allcheck
guess=read

$NBO PLOT FILE=Fe3CNMe2THF+_aab_1_14_ubpv86_def2SVP_opt $END
```

3. *trans*-(α,β)-[**1**(CNMe)(THF)]$^{+}$

```
%mem=243GB
%LindaWorker=
%usessh
%nprocshared=28
%chk=Fe3CNMeTHF+_trans_ab_ubpv86_def2SVP_fragmented_opt.chk
#p ubvp86 def2svp gfinput scf=(xqc,maxconventional=256) guess=(fragment=4,only)

Fe3CNMeTHF+_trans_ab_ubpv86_def2SVP_fragmented_opt

1 14 1 2 0 5 0 5 0 5
 C(Fragment=4)     -0.46632528    4.33281731    6.53820505
 C(Fragment=2)      2.55340441   -1.76726423    7.07518957
 C(Fragment=4)     -1.71631544    4.69887369    6.01502370
 C(Fragment=2)      3.47917892   -0.75186332    6.79057868
 C(Fragment=3)     -3.55704557   -0.11667630    6.45618464
 C(Fragment=4)      0.50013001    3.75989778    5.69360106
 C(Fragment=3)     -2.38590747   -0.88040026    6.30574267
 C(Fragment=2)      1.86517609   -2.39032612    6.01989419
 C(Fragment=3)     -4.45543134   -0.00554760    5.38280770
 C(Fragment=4)     -2.00280186    4.49419873    4.65450727
 C(Fragment=2)      3.71899751   -0.36203133    5.46157758
 C(Fragment=4)     -0.69311242    8.23496745    2.89106893
 C(Fragment=4)      0.21258211    3.54701181    4.33779130
 C(Fragment=4)      0.60611498    8.74080782    2.71553601
 C(Fragment=3)     -2.11724186   -1.52929457    5.09115295
 C(Fragment=4)     -1.04190148    3.91084025    3.80143989
 C(Fragment=2)      2.08979956   -1.98957559    4.69441328
 C(Fragment=4)     -1.04290520    6.96622292    2.40474732
 C(Fragment=2)      3.02165764   -0.97037220    4.39529167
 C(Fragment=2)      5.09610340    3.13514234    3.41584383
 C(Fragment=3)     -4.18949246   -0.65943224    4.16608983
 C(Fragment=2)      6.50404475   -3.52726520    4.41738848
 C(Fragment=2)      6.20444428   -4.88365621    4.19941485
```

C(Fragment=2)	4.17839824	2.07612405	3.33404321
C(Fragment=4)	-5.44375706	3.58051637	2.17633357
C(Fragment=3)	-3.02408403	-1.43496161	4.01290407
C(Fragment=4)	-5.82170249	4.54488749	1.22509384
C(Fragment=4)	-4.08583323	3.31534683	2.41383970
C(Fragment=4)	1.56058521	7.95144084	2.04923386
C(Fragment=2)	6.38841698	2.99196033	2.87980379
C(Fragment=3)	-2.82105243	-5.89929496	4.34665882
C(Fragment=2)	5.83814721	-2.52070688	3.70269688
C(Fragment=3)	-2.99493257	-4.54771299	4.00862078
C(Fragment=4)	-0.08986179	6.15868410	1.72987455
C(Fragment=4)	-4.83501778	5.23754441	0.50452222
C(Fragment=4)	-3.08962939	4.03357434	1.71534500
C(Fragment=2)	5.21380350	-5.22266925	3.26176153
C(Fragment=2)	4.54177850	0.85387305	2.72411042
C(Fragment=4)	-3.47470921	4.98301054	0.74713824
C(Fragment=4)	1.22266824	6.67820783	1.57283890
C(Fragment=3)	-7.11450241	-4.35048344	2.24867020
C(Fragment=2)	4.84185677	-2.84810004	2.74526709
C(Fragment=3)	-6.19065440	-3.29413210	2.28413314
C(Fragment=2)	4.53449698	-4.22133359	2.55258640
C(Fragment=3)	-1.98754821	-6.72096498	3.56940519
C(Fragment=2)	6.75201515	1.78605593	2.25755253
C(Fragment=3)	-2.33269537	-3.99840941	2.88696789
C(Fragment=2)	5.83224169	0.72505673	2.17381936
C(Fragment=3)	-6.87106183	-5.48220899	1.45158731
C(Fragment=3)	-4.99955001	-3.34235981	1.51501676
C(Fragment=3)	-1.33401667	-6.18591900	2.44720188
C(Fragment=3)	-1.50590776	-4.83432521	2.10779134
C(Fragment=3)	-5.69375146	-5.54396572	0.68474723
C(Fragment=4)	3.44178899	7.20308695	-3.55354340
C(Fragment=3)	-4.77082510	-4.48881032	0.71077114
C(Fragment=4)	3.14153000	5.89399292	-3.14690587
C(Fragment=4)	2.67677435	8.29477527	-3.11037877
C(Fragment=4)	2.04988254	5.63581142	-2.27511886
C(Fragment=2)	5.35434542	-4.98456510	-0.88258843
C(Fragment=2)	6.25686738	-5.97599375	-1.28819860
C(Fragment=4)	4.64852629	2.78616316	-1.37198252
C(Fragment=4)	1.59002509	8.05391406	-2.25020523
C(Fragment=4)	6.05117084	2.78225776	-1.38447868
C(Fragment=4)	1.27792472	6.75077809	-1.84543073
C(Fragment=3)	-6.64562830	-2.18813405	-1.76455580
C(Fragment=2)	4.21098651	-4.64469891	-1.66251032
C(Fragment=2)	1.35042363	-5.93673362	-1.00598811
C(Fragment=2)	0.61789155	-7.13093363	-0.89584713
C(Fragment=3)	-7.82064786	-2.66840739	-2.35787358
C(Fragment=3)	-5.05791593	2.06924400	-2.84179186
C(Fragment=3)	-5.45087958	3.33873910	-3.29767960
C(Fragment=4)	3.91871295	2.70860657	-2.57869253
C(Fragment=3)	-5.78902781	-1.27188692	-2.43926185
C(Fragment=2)	6.05391187	-6.68536591	-2.48504245

C(Fragment=2)	0.86806629	-4.86774121	-1.79204395
C(Fragment=3)	-3.80012267	1.53790359	-3.19667531
C(Fragment=3)	-4.59233342	4.09678675	-4.11018503
C(Fragment=2)	-0.61094424	-7.27411243	-1.56066020
C(Fragment=4)	6.74899376	2.68458043	-2.60166383
C(Fragment=3)	-2.92263309	2.32772431	-3.97487637
C(Fragment=3)	-3.32505599	3.58894624	-4.44230919
C(Fragment=2)	4.01619071	-5.38484212	-2.86269155
C(Fragment=2)	-0.38844051	-5.00608683	-2.42471259
C(Fragment=2)	-1.11373092	-6.20424240	-2.31981337
C(Fragment=3)	-8.18325746	-2.27002503	-3.65719166
C(Fragment=4)	1.49582114	2.82130801	-4.22462775
C(Fragment=4)	0.75206702	3.90085682	-4.74607885
C(Fragment=2)	4.92217592	-6.38042572	-3.25877059
C(Fragment=3)	-6.16441292	-0.89367971	-3.75895725
C(Fragment=4)	4.62612794	2.60084215	-3.79591289
C(Fragment=4)	6.03201108	2.58652700	-3.80564140
C(Fragment=3)	-7.33820357	-1.38643790	-4.34900379
C(Fragment=2)	2.15455989	-2.92940947	-3.66865970
C(Fragment=3)	-3.00335127	-1.19738721	-4.01760982
C(Fragment=2)	3.27384700	-2.11429302	-3.95256309
C(Fragment=3)	-3.21369292	-2.58509433	-3.85263465
C(Fragment=4)	0.28738722	3.86692643	-6.07278360
C(Fragment=4)	1.73483728	1.69432395	-5.04425046
C(Fragment=3)	-2.58706719	-0.72233125	-5.27821576
C(Fragment=2)	1.36418687	-3.38652157	-4.74344338
C(Fragment=2)	3.59646933	-1.77211541	-5.27421653
C(Fragment=3)	-3.01960371	-3.47094668	-4.92397191
C(Fragment=4)	0.54755202	2.75348307	-6.88865549
C(Fragment=4)	1.26923128	1.66499699	-6.36844525
C(Fragment=3)	-2.38715924	-1.61207923	-6.34769902
C(Fragment=2)	1.68887537	-3.04236951	-6.06716697
C(Fragment=2)	2.80470201	-2.23566235	-6.33994911
C(Fragment=3)	-2.60307947	-2.98852398	-6.17666528
H(Fragment=4)	-0.24387147	4.49364457	7.60447974
H(Fragment=2)	2.37243104	-2.07764296	8.11598284
H(Fragment=2)	4.03191551	-0.26158068	7.60713547
H(Fragment=3)	-3.76414761	0.39626816	7.40805793
H(Fragment=4)	-2.47742893	5.15434602	6.66748326
H(Fragment=3)	-1.66880182	-0.96358179	7.13678956
H(Fragment=4)	1.48182405	3.46659217	6.09605941
H(Fragment=4)	-1.45404519	8.83939788	3.40989545
H(Fragment=2)	1.14666254	-3.19832317	6.22770622
H(Fragment=3)	-5.37327838	0.59365038	5.49135291
H(Fragment=2)	4.46460940	0.42007569	5.26066320
H(Fragment=4)	-2.98339806	4.79588217	4.25921561
H(Fragment=2)	4.79847153	4.07870938	3.89850963
H(Fragment=4)	-2.07052346	6.59923908	2.53289508
H(Fragment=2)	7.27410629	-3.24538159	5.15300211
H(Fragment=2)	3.17118831	2.19600553	3.76075922
H(Fragment=3)	-1.19033499	-2.11303090	4.98203647

H(Fragment=4)	0.96649265	3.09108052	3.67745947
H(Fragment=4)	-6.21096904	3.02364859	2.73586418
H(Fragment=3)	-3.34326645	-6.31182694	5.22394540
H(Fragment=3)	-3.65228767	-3.91572140	4.62337461
H(Fragment=4)	-6.88734212	4.75440408	1.04238978
H(Fragment=4)	-3.80202308	2.54772014	3.15079881
H(Fragment=2)	1.54113548	-2.48524266	3.87817445
H(Fragment=3)	-4.88527442	-0.56877887	3.31813632
H(Fragment=4)	2.58601668	8.32617046	1.90480181
H(Fragment=3)	-8.03187102	-4.29080951	2.85579803
H(Fragment=2)	6.09503249	-1.46613816	3.87538048
H(Fragment=2)	4.95386887	-6.27874706	3.08834370
H(Fragment=4)	-5.12243550	5.98452155	-0.25117688
H(Fragment=3)	-6.37484248	-2.41278210	2.91558860
H(Fragment=4)	-2.69693026	5.52282636	0.18618644
H(Fragment=3)	-1.84868256	-7.77973811	3.83844035
H(Fragment=4)	1.97872271	6.05343546	1.07086846
H(Fragment=2)	3.73017169	-4.47945310	1.84646664
H(Fragment=2)	7.76376506	1.66399369	1.83961111
H(Fragment=4)	4.29601210	7.36770806	-4.22952333
H(Fragment=2)	6.11234754	-0.21952180	1.68401832
H(Fragment=4)	3.76423576	5.06446817	-3.50348269
H(Fragment=3)	-5.49347782	-6.42362436	0.05320981
H(Fragment=3)	-0.68246510	-6.81788433	1.82519672
H(Fragment=3)	-0.98996367	-4.42584361	1.22633115
H(Fragment=3)	-3.85576004	-4.52744916	0.10172258
H(Fragment=2)	5.51716400	-4.46818616	0.07579033
H(Fragment=2)	7.12289326	-6.20480682	-0.64677390
H(Fragment=4)	4.11334671	2.85970253	-0.41179442
H(Fragment=3)	-6.36169422	-2.54706055	-0.76283270
H(Fragment=4)	6.59611424	2.85731606	-0.43156477
H(Fragment=2)	2.30931508	-5.83666731	-0.47991014
H(Fragment=3)	-8.44808427	-3.38109568	-1.79951200
H(Fragment=2)	1.02001009	-7.95809477	-0.29024437
H(Fragment=3)	-5.73143835	1.47885372	-2.20427849
H(Fragment=3)	-6.43875783	3.73521579	-3.01608606
H(Fragment=4)	0.97025458	8.89005554	-1.89053009
H(Fragment=4)	0.41386345	6.57226168	-1.18595585
H(Fragment=4)	0.53765146	4.77395600	-4.11470386
H(Fragment=3)	-4.90304870	5.08900919	-4.47253651
H(Fragment=2)	-1.18154770	-8.21202015	-1.47833456
H(Fragment=4)	7.85054425	2.69950725	-2.61220236
H(Fragment=2)	3.90409036	-1.75426693	-3.12525316
H(Fragment=3)	-1.90462869	1.97499133	-4.20144492
H(Fragment=3)	-3.55270176	-2.96933270	-2.87950185
H(Fragment=3)	-2.63032770	4.18252798	-5.05533692
H(Fragment=2)	-0.82712379	-4.16706519	-2.98438097
H(Fragment=2)	-2.08678590	-6.29546171	-2.82673190
H(Fragment=2)	3.14859874	-5.18022049	-3.50129663
H(Fragment=3)	-5.53432215	-0.20102306	-4.33131677
H(Fragment=4)	4.08171407	2.55545156	-4.75002351

H(Fragment=2)	4.73225309	-6.92668608	-4.19646796
H(Fragment=4)	-0.27604003	4.72484521	-6.47176642
H(Fragment=4)	2.27811968	0.82207603	-4.64948248
H(Fragment=4)	6.56672178	2.51592650	-4.76600153
H(Fragment=3)	-7.59326293	-1.06734162	-5.37216399
H(Fragment=3)	-2.42839169	0.35111021	-5.44562839
H(Fragment=2)	0.49328747	-4.03032497	-4.56414152
H(Fragment=2)	4.47925969	-1.14443361	-5.47254357
H(Fragment=3)	-3.21234520	-4.54578670	-4.78250682
H(Fragment=4)	0.18363747	2.73109051	-7.92761426
H(Fragment=4)	1.46512419	0.77739306	-6.98917308
H(Fragment=3)	-2.06614123	-1.21888002	-7.32467706
H(Fragment=2)	1.05993682	-3.41490092	-6.89027562
H(Fragment=2)	3.06226643	-1.97550829	-7.37845396
H(Fragment=3)	-2.45808341	-3.68294669	-7.01875932
N(Fragment=4)	-0.38694913	4.88911250	1.19756189
N(Fragment=2)	4.17356457	-1.88459024	1.95613138
N(Fragment=3)	-4.09741160	-2.24577967	1.50231904
N(Fragment=4)	1.67627485	4.36191544	-1.81386459
N(Fragment=2)	3.36482530	-3.61524227	-1.19454115
N(Fragment=3)	-4.65113637	-0.79512578	-1.75871215
P(Fragment=4)	-1.29445303	3.66650040	1.97932660
P(Fragment=2)	3.31871856	-0.54296010	2.60505425
P(Fragment=3)	-2.61870739	-2.23543001	2.38052527
P(Fragment=4)	2.05960565	2.82871417	-2.45919339
P(Fragment=2)	1.81668320	-3.26936870	-1.85807002
P(Fragment=3)	-3.27733620	-0.11402100	-2.52105074
Fe(Fragment=4)	0.54413420	3.94561881	-0.25887604
Fe(Fragment=3)	-4.57395314	-0.81257789	0.24622592
Fe(Fragment=2)	4.13089685	-2.19240661	0.00166213
Co(Fragment=1)	-0.63551438	1.82622879	1.05229946
Co(Fragment=1)	1.58698480	-0.12428822	1.37081540
Co(Fragment=1)	-1.08017810	-1.16919582	1.30048020
Co(Fragment=1)	1.07232478	1.41386011	-1.14670693
Co(Fragment=1)	-1.57631233	0.04006095	-1.18373647
Co(Fragment=1)	0.80462991	-1.61704415	-0.88953432
Se(Fragment=1)	-0.14529334	0.34055263	2.85181945
Se(Fragment=1)	1.72329050	2.25719932	1.03823548
Se(Fragment=1)	-2.63584240	0.52722446	0.86003221
Se(Fragment=1)	0.93214814	-2.35739422	1.32742154
Se(Fragment=1)	-1.09943285	2.41468623	-1.23052168
Se(Fragment=1)	2.73423357	-0.27836225	-0.67063100
Se(Fragment=1)	-1.45511542	-2.23372260	-0.74336304
Se(Fragment=1)	0.19990868	-0.18886513	-2.65629478
H(Fragment=3)	-9.10131146	-2.65366939	-4.12763947
H(Fragment=3)	-7.59474749	-6.31131099	1.42711612
H(Fragment=2)	6.73312749	-5.66850848	4.76144672
H(Fragment=2)	7.10908432	3.82161807	2.94678368
H(Fragment=4)	2.92030511	9.31829356	-3.43297805
H(Fragment=4)	0.87321391	9.73842425	3.09591550
H(Fragment=2)	6.75815196	-7.46941286	-2.80200217

```
 C(Fragment=2)      5.87899001  -1.73886358  -0.77845287
 C(Fragment=2)      8.06857491  -1.18005336  -2.05621078
 H(Fragment=2)      8.23975253  -1.93866565  -2.84716634
 H(Fragment=2)      7.98581080  -0.17837027  -2.52692898
 H(Fragment=2)      8.94237895  -1.18467407  -1.37292413
 N(Fragment=2)      6.89076918  -1.48621985  -1.34252170
 O(Fragment=3)     -6.16500215  -0.03614024   0.66825389
 C(Fragment=3)     -6.80498991  -0.13095865   1.94394442
 C(Fragment=3)     -6.94968462   0.75392231  -0.22954071
 C(Fragment=3)     -8.11346870   0.65106653   1.90126303
 H(Fragment=3)     -6.96958316  -1.22089486   2.14382582
 H(Fragment=3)     -6.08804516   0.28432895   2.69795430
 C(Fragment=3)     -8.20717052   1.22409485   0.49376605
 H(Fragment=3)     -6.30487139   1.60967677  -0.55650259
 H(Fragment=3)     -7.18662901   0.10407795  -1.11076913
 H(Fragment=3)     -8.11236179   1.46525918   2.66679424
 H(Fragment=3)     -8.98115614  -0.01820674   2.12067619
 H(Fragment=3)     -8.25545894   2.34036625   0.51731724
 H(Fragment=3)     -9.12425239   0.85690046  -0.02880259

--link1--
%mem=243GB
%LindaWorker=n2181,n2182
%usessh
%nprocshared=28
%chk=Fe3CNMeTHF+_trans_ab_ubpv86_def2SVP_fragmented_opt.chk
#p opt ubvp86 pop=(full,nbo) chkbasis gfinput scf=(xqc,maxconventional=256) geom=allcheck guess=read

$NBO PLOT FILE=Fe3CNMeTHF+_trans_ab_ubpv86_def2SVP_opt $END
```

4. Transition state geometry optimization of *trans*-(α,β)-[**1**(CNMe)(THF)$_2$]$^+$ (note: specification of read-write file necessary to restart the calculation in case of time-out):

```
%mem=115GB
%LindaWorker=
%usessh
%nprocshared=28
%RWF=Fe3CNMeTHF2+_aab_ubpv86_def2SVP_optimized_optts_calcFC_retry.rwf
%NoSave
#p opt=(calcfc,TS,NoEigenTest) ubvp86/def2svp gfinput scf=(xqc,maxconventional=256)

Fe3CNMeTHF2+_aab_ubpv86_def2SVP_optimized_optts_calcFC_retry

1 14
 C             0.89724620    3.86616871    6.76070588
 C             2.41510444   -3.38594019    6.69091819
 C            -0.11381358    4.71359336    6.27954194
 C             3.66202557   -2.81082238    6.39547473
 C            -4.05780303   -0.09217490    6.20249199
 C             1.40128826    2.84997596    5.93286845
```

C	-2.85535170	-0.81988585	6.16987033
C	1.38036536	-3.32606775	5.74275285
C	-4.87330412	-0.04655122	5.05981470
C	-0.62822561	4.53727792	4.98508288
C	3.87333210	-2.17602710	5.16106203
C	-0.44091021	8.12158418	2.83156347
C	0.89309713	2.67582577	4.63532431
C	0.86103632	8.49038183	3.21204712
C	-2.47873916	-1.50999063	5.00675555
C	-0.13852644	3.50513065	4.14925024
C	1.58727928	-2.68441164	4.51073313
C	-0.69814348	6.84522951	2.30520187
C	2.83389960	-2.09897695	4.20507047
C	5.11721637	1.87578720	4.23560739
C	-4.50096426	-0.73845451	3.89419123
C	6.93382225	-4.50869912	3.03177362
C	6.30108951	-5.66134057	3.52799064
C	4.21256870	0.82981098	3.99577637
C	-4.65690597	3.79427774	3.68045152
C	-3.31170319	-1.49484495	3.86536087
C	-5.26769874	4.45149914	2.59937619
C	-3.29005160	3.47066419	3.63534374
C	1.90496717	7.55966028	3.07182694
C	6.22536647	2.05845638	3.39028721
C	-2.54555968	-5.97389038	4.36313464
C	6.18013044	-3.48537422	2.43575881
C	-2.77097898	-4.61675449	4.07506539
C	0.34817457	5.90049005	2.14605182
C	-4.51014575	4.75729303	1.45477225
C	-2.51134634	3.82317199	2.51168921
C	4.90400214	-5.78401502	3.42025522
C	4.40767832	-0.05224828	2.91115764
C	-3.14456225	4.43701324	1.40974008
C	1.65094170	6.28095246	2.55370492
C	-6.81327953	-4.78415488	2.53018325
C	4.77127466	-3.59639348	2.31982559
C	-5.83336551	-3.78033773	2.52406723
C	4.14614166	-4.76703836	2.82207621
C	-2.03349774	-6.83077646	3.37627520
C	6.42051910	1.18986440	2.30369010
C	-2.47156500	-4.09887159	2.79609912
C	5.51453696	0.14297008	2.06122739
C	-7.13872650	-5.47823518	1.35163423
C	-5.14641063	-3.44499473	1.32780785
C	-1.75222276	-6.32683774	2.09469787
C	-1.96594478	-4.97133589	1.80607011
C	-6.45506109	-5.16776934	0.16319779
C	5.37620685	6.15655104	-2.50033905
C	-5.46498729	-4.17411964	0.15248562
C	4.31295985	5.24090069	-2.51678916
C	5.89820777	6.63227223	-1.28502040

C	3.74053026	4.77084372	-1.30457460
C	4.33557272	-5.73583497	-0.79749672
C	5.15466900	-6.83445488	-1.09460174
C	5.07870059	1.64042987	-1.55520154
C	5.35005316	6.16462247	-0.07723345
C	6.43152588	1.34609798	-1.77923572
C	4.29656097	5.24087951	-0.08572351
C	-6.62532710	-1.63817323	-2.05526212
C	3.54074802	-5.10891377	-1.79962616
C	0.49576561	-6.00484947	-1.50085931
C	-0.40465942	-7.08364947	-1.53662651
C	-7.82699274	-1.99240417	-2.68035029
C	-4.95473701	2.70960583	-2.60880690
C	-5.30672519	4.04233384	-2.88223295
C	4.23199223	1.97757467	-2.63484935
C	-5.75252029	-0.66673403	-2.61430249
C	5.18515326	-7.36667712	-2.39443798
C	0.23407571	-4.83311154	-2.24241599
C	-3.68720186	2.21225328	-2.97785184
C	-4.39768131	4.89516439	-3.52938251
C	-1.58318405	-7.00154857	-2.29579237
C	6.95712055	1.37253349	-3.08324769
C	-2.76091621	3.08907514	-3.58849830
C	-3.12367173	4.41455351	-3.87839434
C	3.57752079	-5.66434852	-3.10674611
C	-0.97001629	-4.74513143	-2.97907719
C	-1.86648378	-5.82522041	-3.01086803
C	-8.20532955	-1.39311475	-3.89544865
C	1.74514836	2.97401516	-3.83547717
C	1.23282686	4.27933134	-3.99043959
C	4.38352488	-6.77699951	-3.38835758
C	-6.14369722	-0.08056090	-3.84966218
C	4.76647413	2.00469684	-3.94029239
C	6.12026414	1.69706839	-4.16268129
C	-7.34934693	-0.44046997	-4.47124201
C	1.82216863	-2.99117496	-3.92384283
C	-2.97957063	-0.33591852	-4.27489480
C	2.82795656	-2.02001600	-4.13152078
C	-3.17205462	-1.73354132	-4.35402599
C	0.64245556	4.66834024	-5.20613420
C	1.62661908	2.05518613	-4.90286966
C	-2.63162975	0.36764618	-5.44655234
C	1.25468331	-3.62407010	-5.05026513
C	3.25551974	-1.69334711	-5.42709835
C	-3.01830457	-2.40842321	-5.57459990
C	0.54978170	3.75910744	-6.27325971
C	1.03920031	2.45031479	-6.11598251
C	-2.47400867	-0.31229877	-6.66631198
C	1.67756723	-3.28811754	-6.34880029
C	2.67742695	-2.32324619	-6.54373670
C	-2.66467647	-1.70089175	-6.73589016

H	1.29390277	3.99962298	7.77888249
H	2.25177064	-3.88571492	7.65810461
H	4.48252359	-2.86122230	7.12749890
H	-4.35394269	0.44347304	7.11746892
H	-0.50827335	5.52183076	6.91414855
H	-2.20186297	-0.84864484	7.05505083
H	2.19354124	2.18136596	6.30336613
H	-1.27071769	8.83702267	2.94405086
H	0.40412587	-3.78668694	5.95848941
H	-5.81098946	0.53011461	5.07378017
H	4.85981144	-1.74308534	4.94060726
H	-1.41700007	5.21365207	4.62732661
H	4.95585062	2.55216968	5.08891897
H	-1.72147873	6.57002120	2.01374255
H	8.02692131	-4.40234816	3.11283167
H	3.35361289	0.68655681	4.66895143
H	-1.52697805	-2.06171759	4.99525930
H	1.30409507	1.88505100	3.99113604
H	-5.24535628	3.53503721	4.57386072
H	-2.78412492	-6.36304151	5.36492939
H	-3.19434259	-3.96499475	4.85228810
H	-6.33469470	4.71868631	2.64508864
H	-2.82980704	2.94857945	4.48701750
H	0.77189957	-2.65042067	3.77275383
H	-5.12243713	-0.68575468	2.98813757
H	2.92767834	7.82713927	3.37978189
H	-7.33435995	-5.02289494	3.47050411
H	6.67362124	-2.57952810	2.05527817
H	4.39827306	-6.68288994	3.80557401
H	-4.98276724	5.24940881	0.59104900
H	-5.60319960	-3.23416951	3.44976909
H	-2.55849083	4.66060870	0.50575405
H	-1.86018227	-7.89392890	3.60305915
H	2.46168968	5.54273385	2.46983528
H	3.05377750	-4.86013586	2.73187039
H	7.29077733	1.32700016	1.64320068
H	5.79724503	6.50870993	-3.45510576
H	5.65990962	-0.53670970	1.20749974
H	3.90542746	4.89861906	-3.47825442
H	-6.68100628	-5.71710415	-0.76402670
H	-1.36177451	-6.98974803	1.30847335
H	-1.74416673	-4.59059202	0.79701790
H	-4.89755416	-3.96201299	-0.76633159
H	4.27066661	-5.37206233	0.24163107
H	5.75474760	-7.28948730	-0.29153849
H	4.68238965	1.62912604	-0.52773440
H	-6.33575654	-2.13455524	-1.11818090
H	7.08279781	1.10638957	-0.92526448
H	1.40927835	-6.07911706	-0.89589137
H	-8.46851702	-2.75699769	-2.21475337
H	-0.17380165	-8.00001968	-0.97171111

H	-5.67751249	2.04161035	-2.12044120
H	-6.30554560	4.40940181	-2.60019921
H	5.75746128	6.51302616	0.88472462
H	3.88982808	4.84678537	0.85766204
H	1.29120181	4.98196650	-3.14470216
H	-4.68062438	5.93365783	-3.76015619
H	-2.28447678	-7.84930762	-2.32497122
H	8.02416667	1.16144198	-3.25592090
H	3.29381255	-1.51989887	-3.26758529
H	-1.74420405	2.74719686	-3.83559111
H	-3.46776065	-2.29252540	-3.45386975
H	-2.39955572	5.07262610	-4.38220943
H	-1.21797073	-3.82300608	-3.52642943
H	-2.79634646	-5.74559347	-3.59476417
H	2.98100081	-5.21585716	-3.90999570
H	-5.49995262	0.66232286	-4.33748416
H	4.13399158	2.29170818	-4.79229299
H	4.38770077	-7.18638913	-4.41087029
H	0.26122017	5.69493533	-5.32263194
H	1.98852570	1.02144923	-4.79400370
H	6.52493016	1.73483825	-5.18588638
H	-7.61777183	0.03498284	-5.42751085
H	-2.49765651	1.45734695	-5.42614007
H	0.49287618	-4.40569035	-4.92475745
H	4.04995526	-0.94295366	-5.56132591
H	-3.19245021	-3.49437082	-5.62243514
H	0.09469490	4.06847046	-7.22669772
H	0.96202114	1.72658927	-6.94150680
H	-2.20953739	0.25556444	-7.57152295
H	1.22319004	-3.79624415	-7.21323963
H	3.01169215	-2.06818308	-7.56107440
H	-2.55020468	-2.22950024	-7.69444469
N	0.13517689	4.61858786	1.57552656
N	4.00582184	-2.58831654	1.66582306
N	-4.17426315	-2.42288014	1.26186897
N	2.64968344	3.88235747	-1.25397136
N	2.82238470	-3.95178926	-1.41665552
N	-4.56422992	-0.35208862	-1.92381406
P	-0.71461934	3.37057137	2.39024254
P	3.15849877	-1.37546672	2.52733802
P	-2.81548014	-2.34993193	2.29755173
P	2.49649069	2.48390163	-2.21187512
P	1.38263723	-3.38444604	-2.14671671
P	-3.21732283	0.45829293	-2.60439091
Fe	-3.98379412	-1.03513887	-0.14157613
Fe	3.99241100	-2.82720550	-0.27648551
Co	-0.39492993	1.57862936	1.20472569
Co	1.50446896	-0.58537404	1.37079116
Co	-1.33013354	-1.24130913	1.17188426
Co	1.26080606	1.14361477	-1.04120096
Co	-1.60093777	0.25845128	-1.16736833

Co	0.54117718	-1.70009365	-1.05811671
Se	-0.19552732	-0.06879737	2.86654999
Se	1.94696362	1.76885818	1.16614030
Se	-2.64601868	0.73794488	0.93878845
Se	0.53452638	-2.67073381	1.06910005
Se	-0.68910534	2.49424159	-0.98282606
Se	2.63646272	-0.76443267	-0.67915554
Se	-1.80402237	-2.09131960	-0.98344555
Se	0.14904759	-0.09746948	-2.69961414
H	-9.14739804	-1.67250570	-4.39003165
H	-7.91104186	-6.26183620	1.36212560
H	6.89384254	-6.46113679	3.99713464
H	6.93734583	2.87603653	3.57988050
H	6.72825811	7.35442693	-1.27914316
H	1.05876686	9.49219961	3.62205697
H	5.81440937	-8.23841366	-2.62830494
C	5.74590590	-2.57567514	-1.13595962
C	8.00656365	-2.59685328	-2.41032500
H	8.04698935	-3.50022740	-3.05200657
H	8.10285242	-1.69513546	-3.04858374
H	8.85803556	-2.62937236	-1.70090867
N	6.78517768	-2.56738945	-1.70147350
O	-6.07242371	-0.07341071	0.77043485
C	-7.30480651	-0.67767376	1.21821852
C	-6.32271365	1.34324700	0.75328242
C	-8.43960066	0.16710840	0.59630681
H	-7.29719578	-1.73889518	0.91036749
H	-7.34236063	-0.64407982	2.33212794
C	-7.73586244	1.48538590	0.16872080
H	-6.26837831	1.74034918	1.79377139
H	-5.51881160	1.82189062	0.16381990
H	-9.25192616	0.33992937	1.32934589
H	-8.88145869	-0.34340991	-0.28019165
H	-8.24972784	2.39213892	0.54388596
H	-7.69257913	1.55583876	-0.93629048
Fe	1.10647460	3.91034403	0.01308453
O	0.58916907	6.00645219	-1.01195491
C	1.40471675	7.21389068	-1.01417705
C	-0.79972272	6.35933160	-1.24745014
C	0.60617813	8.21346147	-1.84638481
H	1.54039548	7.56577334	0.03226219
H	2.39295045	6.95299900	-1.43443501
C	-0.83624144	7.88474985	-1.42623304
H	-1.14224284	5.80859451	-2.14963273
H	-1.40290228	6.01625641	-0.38377966
H	0.75853069	8.02588348	-2.93029353
H	0.89409427	9.26275286	-1.63942889
H	-1.59650368	8.20580184	-2.16499542
H	-1.07017177	8.37794045	-0.46077531

5. *cis*-(α,β)-[**1**(THF)$_2$]$^+$

```
%mem=243GB
%LindaWorker=n2181,n2182
%usessh
%nprocshared=28
%chk=Fe3THF2+_cis_ab_ubpv86_def2SVP_opt.chk
#p ubvp86 guess=(fragment=4,only) def2svp gfinput scf=(xqc,maxconventional=256)

Fe3THF2+_cis_ab_ubpv86_def2SVP_opt

1 14 0 5 0 5 0 5 1 2
 C(Fragment=2)     -4.30397156    1.11291904   -5.93978289
 C(Fragment=1)     -1.33364484   -3.50405616   -6.72912193
 C(Fragment=2)     -3.06786910    1.75766648   -6.10139696
 C(Fragment=1)     -2.65243997   -3.36044473   -6.27142145
 C(Fragment=3)      2.07340822    2.07211837   -6.78244702
 C(Fragment=2)     -4.87278149    1.02320617   -4.65803461
 C(Fragment=3)      1.50617743    0.83032398   -6.44495433
 C(Fragment=1)     -0.27935300   -3.55873424   -5.80011608
 C(Fragment=3)      2.95216243    2.69987043   -5.88401767
 C(Fragment=2)     -2.40167981    2.30742895   -4.99286241
 C(Fragment=1)     -2.92077802   -3.27244735   -4.89418829
 C(Fragment=2)     -5.49692234    5.77677434   -3.44416665
 C(Fragment=2)     -4.20790428    1.56799832   -3.54869317
 C(Fragment=2)     -6.58250163    6.06730948   -2.60192649
 C(Fragment=3)      1.81757127    0.22014714   -5.21894740
 C(Fragment=2)     -2.96372137    2.21916391   -3.70262257
 C(Fragment=1)     -0.54334794   -3.45913374   -4.42630330
 C(Fragment=2)     -4.45603481    4.93530832   -3.02332308
 C(Fragment=1)     -1.86849264   -3.31608378   -3.95652571
 C(Fragment=1)     -6.15305212   -2.28144123   -2.14809251
 C(Fragment=3)      3.26671888    2.08956937   -4.65635412
 C(Fragment=1)     -2.42755911   -7.68160002   -3.55786592
 C(Fragment=1)     -1.40658169   -8.58132264   -3.20954295
 C(Fragment=1)     -4.76428029   -2.23919165   -2.35121163
 C(Fragment=2)      0.75284677    5.26289229   -3.95065372
 C(Fragment=3)      2.71165864    0.83940412   -4.31641189
 C(Fragment=2)      0.46295988    6.54172731   -3.44464794
 C(Fragment=2)     -0.02145783    4.15668262   -3.56572705
 C(Fragment=2)     -6.60944226    5.48811043   -1.32075750
 C(Fragment=1)     -6.73649787   -3.36012482   -1.46003670
 C(Fragment=3)      4.97854474   -3.02818825   -4.85380970
 C(Fragment=1)     -2.47208949   -6.39115458   -3.00827685
 C(Fragment=3)      4.28174348   -1.84843771   -4.54082447
 C(Fragment=2)     -4.46190069    4.34263419   -1.72845404
 C(Fragment=2)     -0.59541734    6.70364575   -2.53577438
 C(Fragment=2)     -1.12217571    4.32067730   -2.69459405
 C(Fragment=1)     -0.41516999   -8.16436573   -2.30265764
 C(Fragment=1)     -3.94292868   -3.28693299   -1.87664758
 C(Fragment=2)     -1.38163965    5.60116369   -2.16167278
 C(Fragment=2)     -5.57717434    4.64260419   -0.89518897
```

C(Fragment=3)	7.72356293	0.63797127	-3.45782519
C(Fragment=1)	-1.47921485	-5.95680536	-2.08950283
C(Fragment=3)	6.33204400	0.78405381	-3.36136654
C(Fragment=1)	-0.44486234	-6.87469378	-1.75928527
C(Fragment=3)	5.43919863	-3.87043636	-3.82980751
C(Fragment=1)	-5.92134900	-4.39179791	-0.96513154
C(Fragment=3)	4.02634550	-1.50153425	-3.19661430
C(Fragment=1)	-4.53051938	-4.35343375	-1.16536584
C(Fragment=3)	8.52053705	0.55439685	-2.30247147
C(Fragment=3)	5.69334515	0.84628785	-2.09413467
C(Fragment=3)	5.19924255	-3.52733040	-2.48837607
C(Fragment=3)	4.49273232	-2.35745589	-2.17349521
C(Fragment=3)	7.90228456	0.60574252	-1.04130051
C(Fragment=2)	-7.71430879	1.83386191	1.03753188
C(Fragment=3)	6.51014008	0.73889533	-0.93780350
C(Fragment=2)	-6.31843366	1.74771633	0.93486989
C(Fragment=2)	-8.33450008	1.86719518	2.29836701
C(Fragment=2)	-5.49601014	1.71413577	2.09182872
C(Fragment=1)	-0.61975922	-6.87189454	1.74446906
C(Fragment=1)	-0.84795747	-8.14111458	2.28884829
C(Fragment=2)	-4.81334927	-1.63404339	2.16535250
C(Fragment=2)	-7.53553234	1.81291734	3.45409739
C(Fragment=2)	-5.69202973	-2.68112215	2.47930228
C(Fragment=2)	-6.13820288	1.74008128	3.35865269
C(Fragment=3)	6.23205976	3.70950204	0.89857988
C(Fragment=1)	0.53666553	-6.11827745	2.08530155
C(Fragment=1)	3.79895761	-5.01161807	1.16931394
C(Fragment=1)	5.16714123	-5.26474566	0.96927463
C(Fragment=3)	7.38527426	4.38010924	1.32519058
C(Fragment=3)	2.24429167	5.30862832	2.19742487
C(Fragment=3)	1.64070472	6.51668918	2.58464671
C(Fragment=2)	-4.21315522	-0.86761180	3.18980085
C(Fragment=3)	5.08515017	3.58441631	1.73352950
C(Fragment=1)	0.06030068	-8.69970535	3.20663020
C(Fragment=1)	3.38309535	-3.86371964	1.87526588
C(Fragment=3)	1.78522567	4.07861762	2.71470867
C(Fragment=3)	0.56847089	6.51285121	3.49168793
C(Fragment=1)	6.13198737	-4.36917040	1.45992825
C(Fragment=2)	-5.97496635	-2.99080921	3.82046196
C(Fragment=3)	0.67056408	4.07996908	3.58361736
C(Fragment=3)	0.07976539	5.28972520	3.98220673
C(Fragment=1)	1.44368917	-6.69424974	3.01494979
C(Fragment=1)	4.35670942	-2.95365820	2.34596255
C(Fragment=1)	5.72226474	-3.21088033	2.14381231
C(Fragment=3)	7.45229503	4.94982484	2.60914096
C(Fragment=2)	-2.55343225	1.23987389	4.31517490
C(Fragment=2)	-2.91309477	2.55833832	4.66034408
C(Fragment=1)	1.20086902	-7.96224389	3.56497239
C(Fragment=3)	5.17536807	4.16347684	3.03147805
C(Fragment=2)	-4.51177095	-1.17888972	4.53384804
C(Fragment=2)	-5.38244013	-2.23715862	4.84562534

C(Fragment=3)	6.33638293	4.82892336	3.45321241
C(Fragment=1)	1.32424244	-3.56381853	3.95110489
C(Fragment=3)	3.27866542	1.70818934	3.69971862
C(Fragment=1)	-0.00951323	-3.51317903	4.41540183
C(Fragment=3)	4.41610278	0.88574610	3.54011442
C(Fragment=2)	-2.50950535	3.10950549	5.88993784
C(Fragment=2)	-1.76296420	0.48938355	5.21490102
C(Fragment=3)	2.72890495	1.85912104	4.98948108
C(Fragment=1)	2.36859256	-3.66380974	4.89325922
C(Fragment=1)	-0.29026598	-3.56383565	5.78855340
C(Fragment=3)	4.99063415	0.23645826	4.64373579
C(Fragment=2)	-1.73517927	2.35304663	6.78527277
C(Fragment=2)	-1.36231621	1.04107341	6.44273491
C(Fragment=3)	3.30371399	1.20441870	6.09210805
C(Fragment=1)	2.08514248	-3.70234903	6.26978047
C(Fragment=1)	0.75763110	-3.65303082	6.72216680
C(Fragment=3)	4.43490938	0.39057803	5.92504515
H(Fragment=2)	-4.82625919	0.68699136	-6.81018744
H(Fragment=1)	-1.12664834	-3.58058139	-7.80788553
H(Fragment=1)	-3.48668500	-3.32483663	-6.98920509
H(Fragment=3)	1.82996838	2.54899599	-7.74456582
H(Fragment=2)	-2.61314261	1.83958443	-7.10075994
H(Fragment=3)	0.81195473	0.33041591	-7.13762746
H(Fragment=2)	-5.85035268	0.53537164	-4.52128424
H(Fragment=2)	-5.45087175	6.21264584	-4.45489973
H(Fragment=1)	0.75871765	-3.68367542	-6.14493925
H(Fragment=3)	3.40586853	3.66963178	-6.14306189
H(Fragment=1)	-3.96257161	-3.18193791	-4.55508583
H(Fragment=2)	-1.44304704	2.81910453	-5.15096507
H(Fragment=1)	-6.78488923	-1.46242596	-2.52499678
H(Fragment=2)	-3.62572240	4.74147673	-3.71363470
H(Fragment=1)	-3.21176266	-7.98655836	-4.26892411
H(Fragment=1)	-4.31870040	-1.38215655	-2.87982255
H(Fragment=3)	1.35782909	-0.74749191	-4.96694561
H(Fragment=2)	-4.66430448	1.50334117	-2.55002635
H(Fragment=2)	1.59376918	5.11275502	-4.64428334
H(Fragment=3)	5.16773414	-3.28325407	-5.90825599
H(Fragment=3)	3.94496695	-1.19187642	-5.35553052
H(Fragment=2)	1.06292631	7.41137540	-3.75677126
H(Fragment=2)	0.25620566	3.15840511	-3.93619931
H(Fragment=1)	0.28997500	-3.50037835	-3.70656230
H(Fragment=3)	3.95005497	2.58151301	-3.94804652
H(Fragment=1)	-7.82585903	-3.39078967	-1.30379493
H(Fragment=2)	-7.45362819	5.68439275	-0.64056799
H(Fragment=3)	8.19195904	0.59346928	-4.45387936
H(Fragment=1)	-3.28884570	-5.71393433	-3.29182861
H(Fragment=1)	0.39919125	-8.84897064	-2.01818831
H(Fragment=2)	-0.82095663	7.69807024	-2.11990606
H(Fragment=3)	5.72703813	0.85935549	-4.27575254
H(Fragment=2)	-2.21208301	5.73250379	-1.45397492
H(Fragment=3)	5.98605786	-4.79379563	-4.07604646

H(Fragment=2)	-5.63283538	4.18313360	0.10264285
H(Fragment=1)	0.34662886	-6.54954526	-1.06478056
H(Fragment=1)	-6.36850399	-5.23846441	-0.42129263
H(Fragment=2)	-8.32244542	1.86268344	0.11983049
H(Fragment=1)	-3.89019245	-5.15656242	-0.77222842
H(Fragment=2)	-5.83171114	1.68530553	-0.05011232
H(Fragment=3)	8.50714188	0.52928502	-0.12417178
H(Fragment=3)	5.55432278	-4.17503124	-1.67324967
H(Fragment=3)	4.30674806	-2.10522017	-1.11794183
H(Fragment=3)	6.01900554	0.74429362	0.04695678
H(Fragment=1)	-1.34614578	-6.43422193	1.04082466
H(Fragment=1)	-1.75260019	-8.69676990	1.99617082
H(Fragment=2)	-4.59738435	-1.40784914	1.10951533
H(Fragment=3)	6.21279755	3.25167169	-0.10137049
H(Fragment=2)	-6.14865360	-3.26055226	1.66338768
H(Fragment=1)	3.04297483	-5.70937071	0.78043825
H(Fragment=3)	8.24851735	4.44457468	0.64365956
H(Fragment=1)	5.47803352	-6.17256580	0.42912229
H(Fragment=3)	3.08699730	5.31700583	1.49212633
H(Fragment=3)	2.02103402	7.46799324	2.18083410
H(Fragment=2)	-8.00622910	1.83236154	4.44987453
H(Fragment=2)	-5.53034544	1.71068426	4.27374798
H(Fragment=2)	-3.51468750	3.15108054	3.95508165
H(Fragment=3)	0.11273004	7.46245984	3.81458118
H(Fragment=1)	7.20347875	-4.56819981	1.30388315
H(Fragment=2)	-6.65802623	-3.81885236	4.06551402
H(Fragment=1)	-0.83729945	-3.44041718	3.69184388
H(Fragment=3)	0.23761555	3.13359328	3.94132289
H(Fragment=3)	4.86238749	0.76842005	2.54173097
H(Fragment=3)	-0.77552499	5.26561613	4.67409406
H(Fragment=1)	4.04947903	-2.03548175	2.87033858
H(Fragment=1)	6.47299316	-2.49767414	2.51769990
H(Fragment=1)	2.34928959	-6.14602184	3.30719200
H(Fragment=3)	4.32652288	4.09849008	3.72349374
H(Fragment=2)	-4.07210938	-0.58788037	5.34968036
H(Fragment=1)	1.92315797	-8.37941156	4.28458688
H(Fragment=2)	-2.81164415	4.13564661	6.15287813
H(Fragment=2)	-1.45491294	-0.53586686	4.95963395
H(Fragment=2)	-5.60307799	-2.46576245	5.90003009
H(Fragment=3)	6.36162654	5.26126202	4.46619164
H(Fragment=3)	1.85248702	2.50035806	5.15212656
H(Fragment=1)	3.41387606	-3.72525348	4.55844332
H(Fragment=1)	-1.33685245	-3.53701132	6.12917903
H(Fragment=3)	5.88831527	-0.38534332	4.50283898
H(Fragment=2)	-1.42203967	2.78419982	7.74880278
H(Fragment=2)	-0.75168196	0.43915820	7.13300282
H(Fragment=3)	2.86065462	1.33742245	7.09119517
H(Fragment=1)	2.91322395	-3.78061714	6.99129936
H(Fragment=1)	0.53781841	-3.69129060	7.80043699
H(Fragment=3)	4.88643304	-0.11741005	6.79100662
N(Fragment=2)	-3.44596847	3.50663315	-1.22255030

N(Fragment=1)	-1.46788113	-4.68975478	-1.48180719
N(Fragment=3)	4.30228684	1.04067382	-1.93204885
N(Fragment=2)	-4.09182900	1.68938796	1.93009534
N(Fragment=1)	0.72098335	-4.86456842	1.47790351
N(Fragment=3)	3.94952485	2.92099999	1.22634646
P(Fragment=2)	-2.15785953	2.87206893	-2.15117278
P(Fragment=1)	-2.10722834	-3.23731152	-2.11237255
P(Fragment=3)	3.12152301	0.03840009	-2.68566319
P(Fragment=2)	-3.08084132	0.51527210	2.68229256
P(Fragment=1)	1.57704897	-3.52823681	2.10746941
P(Fragment=3)	2.58020973	2.48997126	2.15555624
Fe(Fragment=2)	-3.25494006	3.02985993	0.72864428
Fe(Fragment=3)	3.68237440	2.48990131	-0.72621400
Fe(Fragment=1)	-0.31574788	-4.07419219	-0.00258577
Co(Fragment=4)	-0.99250419	1.41999547	-1.05024710
Co(Fragment=4)	-1.08084624	-1.66468360	-1.03154868
Co(Fragment=4)	1.41983368	-0.19298047	-1.35728181
Co(Fragment=4)	-1.43453552	0.02686148	1.35363321
Co(Fragment=4)	1.19899871	1.24449831	1.05095149
Co(Fragment=4)	0.80740090	-1.81660229	1.02331109
Se(Fragment=4)	-0.46444511	-0.14218794	-2.72897095
Se(Fragment=4)	-2.73003513	-0.03340915	-0.57142401
Se(Fragment=4)	1.29721046	2.13992612	-1.15006617
Se(Fragment=4)	1.14732467	-2.57623805	-1.25202216
Se(Fragment=4)	-0.95191508	2.31283416	1.15354152
Se(Fragment=4)	-1.53813296	-2.36876022	1.23887703
Se(Fragment=4)	2.68908210	-0.46057251	0.56719739
Se(Fragment=4)	0.43371044	-0.22075581	2.72420432
H(Fragment=1)	-1.38020537	-9.59301217	-3.64177790
H(Fragment=1)	-0.12184757	-9.69497176	3.63941928
H(Fragment=3)	9.61236989	0.44270491	-2.38512020
H(Fragment=3)	8.36049914	5.47093328	2.94787281
H(Fragment=2)	-9.43042658	1.92730375	2.38040273
H(Fragment=2)	-7.39662596	6.72619188	-2.93987294
O(Fragment=2)	-3.32846339	5.13915015	1.67883491
C(Fragment=2)	-3.93431100	5.44497906	2.93795308
C(Fragment=2)	-2.77438270	6.31844468	1.08873362
C(Fragment=2)	-3.77653724	6.93631652	3.21457884
H(Fragment=2)	-5.00959949	5.13980525	2.86293273
H(Fragment=2)	-3.41863233	4.81485434	3.70738327
C(Fragment=2)	-3.02539532	7.50195309	2.01706879
H(Fragment=2)	-1.68155629	6.12302978	0.93857595
H(Fragment=2)	-3.27291982	6.44806165	0.09391502
H(Fragment=2)	-3.20718158	7.10452084	4.16141060
H(Fragment=2)	-4.77515110	7.42477354	3.32916695
H(Fragment=2)	-2.06006568	7.96833906	2.33261763
H(Fragment=2)	-3.62803435	8.28859133	1.50037222
O(Fragment=3)	4.08777749	4.56169197	-1.66226863
C(Fragment=3)	4.67681927	4.77360484	-2.94838057
C(Fragment=3)	3.78482986	5.81184744	-1.03673320
C(Fragment=3)	4.77728207	6.27321562	-3.20640489

```
H(Fragment=3)      5.68180589    4.27896069   -2.93011843
H(Fragment=3)      4.01913312    4.25874756   -3.69495633
C(Fragment=3)      4.19965116    6.94555782   -1.96846800
H(Fragment=3)      2.68330659    5.81369521   -0.83265735
H(Fragment=3)      4.34639361    5.83391338   -0.06762890
H(Fragment=3)      4.20218676    6.55604907   -4.12194820
H(Fragment=3)      5.84082517    6.57596903   -3.36816639
H(Fragment=3)      3.32005026    7.58282416   -2.23141688
H(Fragment=3)      4.95868785    7.60274382   -1.47763327

--link1--
%mem=243GB
%LindaWorker=n2181,n2182
%usessh
%nprocshared=28
%chk=Fe3THF2+_cis_ab_ubpv86_def2SVP_opt.chk
#p opt ubvp86 chkbasis gfinput scf=(xqc,maxconventional=256) geom=allcheck guess=read
```

6. (α,α)-[**1**$(CNMe)_2$]$^+$

```
%mem=115GB
%LindaWorker=
%usessh
%nprocshared=28
%chk=Fe3CNMe2+_aa_1_14_ubpy86_def2svp_opt.chk
#p ubvp86 def2svp gfinput scf=(xqc,maxconventional=256) guess=(fragment=4,only)

Fe3CNMe2+_aa_1_14_ubpy86_def2svp_opt

1 14 0 5 0 5 0 5 1 2
C(Fragment=3)     -0.11669659   -0.08078994    0.00000000
C(Fragment=2)     -5.94126294   -0.60548992   -0.50336028
C(Fragment=3)     -0.19771166   -1.48192473   -0.01052052
C(Fragment=2)     -5.26338901    0.58699908   -0.20822548
C(Fragment=1)     -2.44444066   -6.31732952   -0.12955862
C(Fragment=3)      0.12753182    0.58636090    1.21217051
C(Fragment=1)     -3.21387663   -5.15251510    0.03955833
C(Fragment=2)     -6.31137261   -1.46828851    0.54332771
C(Fragment=1)     -2.38356843   -7.26627288    0.90478086
C(Fragment=3)     -0.03829888   -2.21298317    1.17898295
C(Fragment=2)     -4.95630194    0.91875492    1.12297294
C(Fragment=3)      4.55761420   -1.15610812    2.50364444
C(Fragment=3)      0.28177800   -0.14079303    2.40270535
C(Fragment=3)      5.36872682   -0.26635400    3.22676415
C(Fragment=1)     -3.92272248   -4.93979382    1.23326646
C(Fragment=3)      0.20273247   -1.55120999    2.40090795
C(Fragment=2)     -5.99230335   -1.14648528    1.87074085
C(Fragment=3)      3.31933764   -1.58303209    3.00665436
C(Fragment=2)     -5.31039114    0.05269461    2.17897077
C(Fragment=2)     -2.16580282    3.41927230    3.25247324
C(Fragment=1)     -3.09285797   -7.05737460    2.10049054
```

C(Fragment=2)	-8.92345589	2.38952297	2.31813968
C(Fragment=2)	-10.07991144	1.62019952	2.53632995
C(Fragment=2)	-2.82747788	2.18164135	3.28702564
C(Fragment=3)	0.95468470	-6.31030859	2.66182718
C(Fragment=1)	-3.88028035	-5.89955775	2.27004418
C(Fragment=3)	2.19710684	-6.66268451	3.21469455
C(Fragment=3)	0.40988625	-5.03761303	2.89593793
C(Fragment=3)	4.90879908	0.19838191	4.47183004
C(Fragment=2)	-2.74936890	4.55387510	3.84435674
C(Fragment=1)	-8.41249522	-5.64766544	1.77100951
C(Fragment=2)	-7.73126013	2.10334596	2.99962570
C(Fragment=1)	-7.03527060	-5.68043103	2.05178929
C(Fragment=3)	2.83821936	-1.12890547	4.26747874
C(Fragment=3)	2.88736623	-5.73575758	4.01204837
C(Fragment=3)	1.11948925	-4.08314295	3.66035327
C(Fragment=2)	-10.02404731	0.54409558	3.43894363
C(Fragment=2)	-4.09439880	2.06723002	3.90278069
C(Fragment=3)	2.35250180	-4.45627760	4.23597670
C(Fragment=3)	3.67150192	-0.21877274	4.97838775
C(Fragment=1)	-6.51095931	-9.96373865	3.40837462
C(Fragment=2)	-7.66246050	1.02678839	3.92322713
C(Fragment=1)	-5.80236353	-8.75360934	3.45493075
C(Fragment=2)	-8.83208518	0.24418190	4.11462694
C(Fragment=1)	-9.34358659	-5.51376519	2.81290236
C(Fragment=2)	-3.99967168	4.44153353	4.47400901
C(Fragment=1)	-6.57454744	-5.56701354	3.38062753
C(Fragment=2)	-4.66803548	3.20421631	4.50556620
C(Fragment=1)	-6.75732280	-10.70200531	4.57803639
C(Fragment=1)	-5.32229388	-8.23863939	4.68878832
C(Fragment=1)	-8.89194185	-5.41241152	4.14008812
C(Fragment=1)	-7.51866592	-5.43154926	4.42273191
C(Fragment=1)	-6.29014119	-10.20335492	5.80756255
C(Fragment=3)	2.42774276	3.23195044	6.65790438
C(Fragment=1)	-5.59461560	-8.98947941	5.86399250
C(Fragment=3)	1.62699244	2.08082315	6.68805480
C(Fragment=3)	2.88736173	3.81056464	7.85345251
C(Fragment=3)	1.27704464	1.46170688	7.91645893
C(Fragment=2)	-9.73394599	0.61907182	7.60276051
C(Fragment=2)	-10.96936219	1.10123133	8.05431938
C(Fragment=3)	-2.04666202	2.59547173	7.91472953
C(Fragment=3)	2.53233438	3.22097551	9.07955896
C(Fragment=3)	-2.45526348	3.92693400	8.08122651
C(Fragment=3)	1.74105882	2.06338145	9.11616460
C(Fragment=1)	-2.45054792	-9.53915916	7.15616670
C(Fragment=2)	-8.94382477	-0.27324409	8.38464681
C(Fragment=2)	-9.13677206	-3.43270584	7.84917244
C(Fragment=2)	-9.97673997	-4.55874051	7.82702014
C(Fragment=1)	-2.29596443	-10.90580915	7.41679824
C(Fragment=1)	0.55191605	-6.98623464	8.64338270
C(Fragment=1)	1.92270432	-7.05514810	8.94411267
C(Fragment=3)	-1.57241114	1.84594449	9.01400762

C(Fragment=1)	-2.29169141	-8.56729212	8.18225527
C(Fragment=2)	-11.48178823	0.70648310	9.30308563
C(Fragment=2)	-7.92299217	-3.45626082	8.57009357
C(Fragment=1)	-0.24192938	-5.94369415	9.16719002
C(Fragment=1)	2.51869696	-6.08207696	9.76268131
C(Fragment=2)	-9.61180452	-5.73157398	8.50893875
C(Fragment=3)	-2.41043660	4.52742418	9.35140686
C(Fragment=1)	0.37715014	-4.93510712	9.94094714
C(Fragment=1)	1.74434427	-5.01463647	10.24926321
C(Fragment=2)	-9.48764382	-0.67112016	9.63840556
C(Fragment=2)	-7.54696398	-4.65287414	9.22408266
C(Fragment=2)	-8.39038152	-5.77606199	9.20091552
C(Fragment=1)	-1.96446973	-11.35501930	8.70819052
C(Fragment=3)	-0.62781235	-0.53211250	10.40476575
C(Fragment=3)	0.67202993	-0.95709979	10.74513465
C(Fragment=2)	-10.72803514	-0.18721005	10.08141510
C(Fragment=1)	-1.95061073	-9.03506681	9.48052229
C(Fragment=3)	-1.52205303	2.45984446	10.28420782
C(Fragment=3)	-1.94463740	3.78987799	10.45140441
C(Fragment=1)	-1.79019546	-10.40676246	9.72990237
C(Fragment=2)	-6.46205461	-1.52148903	10.29535018
C(Fragment=1)	-2.93926895	-6.00163565	10.37945813
C(Fragment=2)	-5.93275216	-0.22998014	10.51728747
C(Fragment=1)	-4.22434556	-6.58848483	10.34867448
C(Fragment=3)	0.94065074	-1.47346691	12.02598559
C(Fragment=3)	-1.66104224	-0.65296290	11.36129431
C(Fragment=1)	-2.42425422	-5.56427511	11.61747121
C(Fragment=2)	-6.69590887	-2.35157148	11.41163974
C(Fragment=2)	-5.64088311	0.21551969	11.81524397
C(Fragment=1)	-4.97079425	-6.73841431	11.52741902
C(Fragment=3)	-0.08730257	-1.57456426	12.97787804
C(Fragment=3)	-1.38924463	-1.16280094	12.64075588
C(Fragment=1)	-3.17601560	-5.71198733	12.79568263
C(Fragment=2)	-6.40157953	-1.90499810	12.71189926
C(Fragment=2)	-5.87152403	-0.62231292	12.92083897
C(Fragment=1)	-4.45081730	-6.29809654	12.75672798
H(Fragment=3)	-0.23675480	0.48972760	-0.93380411
H(Fragment=2)	-6.18742511	-0.86025740	-1.54594445
H(Fragment=2)	-4.97518401	1.27516491	-1.01812618
H(Fragment=1)	-1.89112289	-6.48360257	-1.06682488
H(Fragment=3)	-0.38252197	-2.01834625	-0.95407626
H(Fragment=1)	-3.26213355	-4.39837322	-0.76086301
H(Fragment=3)	0.21243514	1.68401152	1.23025996
H(Fragment=3)	4.89034916	-1.53327349	1.52352149
H(Fragment=2)	-6.85672918	-2.40037717	0.32934897
H(Fragment=1)	-1.78649826	-8.18315452	0.77980020
H(Fragment=2)	-4.44115077	1.86655034	1.33354016
H(Fragment=3)	-0.08958116	-3.30888560	1.13768613
H(Fragment=2)	-1.18271774	3.49803076	2.76320899
H(Fragment=3)	2.72272373	-2.28423078	2.41006524
H(Fragment=2)	-8.94882385	3.23188085	1.60858718

H(Fragment=2)	-2.35323924	1.29976478	2.82863346
H(Fragment=1)	-4.50731986	-4.01516026	1.35401408
H(Fragment=3)	0.48137508	0.38847971	3.34587741
H(Fragment=3)	0.38808536	-7.03405225	2.05716151
H(Fragment=1)	-8.75653412	-5.73775647	0.72882630
H(Fragment=1)	-6.32034551	-5.80858492	1.22623973
H(Fragment=3)	2.61850476	-7.66407566	3.03630999
H(Fragment=3)	-0.59054089	-4.80724410	2.49925278
H(Fragment=2)	-6.28798733	-1.83223462	2.68043058
H(Fragment=1)	-3.04236491	-7.79716920	2.91307470
H(Fragment=3)	5.51379415	0.90891306	5.05741815
H(Fragment=1)	-6.87085253	-10.33577618	2.43601430
H(Fragment=2)	-6.84033141	2.72327059	2.82581814
H(Fragment=2)	-10.91416318	-0.08159697	3.61008041
H(Fragment=3)	3.85390958	-6.00757852	4.46417176
H(Fragment=1)	-5.61129144	-8.20451233	2.52313262
H(Fragment=3)	2.89555678	-3.73491298	4.86238295
H(Fragment=1)	-10.42188541	-5.49093541	2.59172563
H(Fragment=3)	3.32248328	0.17936098	5.94305486
H(Fragment=2)	-8.77235457	-0.62276484	4.79034237
H(Fragment=2)	-4.46504363	5.32428050	4.94022849
H(Fragment=3)	2.68498614	3.68608453	5.68816248
H(Fragment=2)	-5.64567153	3.11066978	5.00165121
H(Fragment=3)	1.23744610	1.64348483	5.75643749
H(Fragment=1)	-6.47687363	-10.76054248	6.73889199
H(Fragment=1)	-9.60918388	-5.31096013	4.96811420
H(Fragment=1)	-7.17698170	-5.34348538	5.46609090
H(Fragment=1)	-5.25136287	-8.59301468	6.83259227
H(Fragment=2)	-9.37157514	0.91570106	6.60680438
H(Fragment=2)	-11.54500993	1.78311459	7.40799845
H(Fragment=3)	-2.09483023	2.13482340	6.91627670
H(Fragment=1)	-2.69369740	-9.19689017	6.13757923
H(Fragment=3)	-2.81390571	4.49088407	7.20705482
H(Fragment=2)	-9.42949063	-2.52377375	7.30630105
H(Fragment=1)	-2.43204569	-11.62732068	6.59609593
H(Fragment=2)	-10.92994732	-4.51228589	7.27749548
H(Fragment=1)	0.09805125	-7.75165514	7.99980902
H(Fragment=1)	2.52404461	-7.88307461	8.53794448
H(Fragment=3)	2.88105266	3.66547969	10.02529365
H(Fragment=3)	1.48326647	1.60669786	10.08221341
H(Fragment=3)	1.47383993	-0.88032516	9.99523155
H(Fragment=1)	3.58817804	-6.15315680	10.01735511
H(Fragment=2)	-10.27132141	-6.61282019	8.49168977
H(Fragment=3)	-2.73295408	5.57233197	9.48239772
H(Fragment=2)	-5.75219666	0.43746016	9.66045344
H(Fragment=1)	-0.19893653	-4.06513317	10.29131101
H(Fragment=1)	-4.63212262	-6.94516072	9.39124738
H(Fragment=1)	2.20264309	-4.22784704	10.86750397
H(Fragment=2)	-6.57919908	-4.72339267	9.74259360
H(Fragment=2)	-8.08285675	-6.69783160	9.71837043
H(Fragment=2)	-8.93303352	-1.36246166	10.28339595

H(Fragment=1)	-1.80928338	-8.31938279	10.30134977
H(Fragment=3)	-1.14527763	1.90145550	11.15297386
H(Fragment=2)	-11.10778480	-0.52076258	11.06041710
H(Fragment=3)	1.96380361	-1.79022184	12.28320108
H(Fragment=3)	-2.68862089	-0.34766335	11.11027530
H(Fragment=3)	-1.89760367	4.25284755	11.44956187
H(Fragment=1)	-1.52543134	-10.73565993	10.74738180
H(Fragment=1)	-1.42347440	-5.11685438	11.67962717
H(Fragment=2)	-7.13086294	-3.35156441	11.28080922
H(Fragment=2)	-5.23373836	1.22814142	11.96205272
H(Fragment=1)	-5.96208836	-7.21591031	11.48663529
H(Fragment=3)	0.12428769	-1.97498755	13.98154238
H(Fragment=3)	-2.20556914	-1.24532200	13.37449686
H(Fragment=1)	-2.75422098	-5.36752035	13.75256474
H(Fragment=2)	-6.59591144	-2.56987375	13.56782341
H(Fragment=2)	-5.64721831	-0.27256657	13.94057062
H(Fragment=1)	-5.03500081	-6.41948421	13.68192995
N(Fragment=3)	1.61967903	-1.52488166	4.85495464
N(Fragment=2)	-6.50612975	0.72029912	4.67639119
N(Fragment=1)	-4.58764798	-7.04531162	4.80670961
N(Fragment=3)	0.51963089	0.26611442	7.89253289
N(Fragment=2)	-7.69779043	-0.68918506	7.86726717
N(Fragment=1)	-2.47026550	-7.21485021	7.84325676
P(Fragment=3)	0.39854980	-2.41220135	4.04585001
P(Fragment=2)	-4.97028566	0.42788591	3.97067143
P(Fragment=1)	-4.78475780	-5.62862844	3.86709324
P(Fragment=3)	-0.99355487	0.10861291	8.69630287
P(Fragment=2)	-6.78224242	-1.99263982	8.50689462
P(Fragment=1)	-2.04497317	-5.83406854	8.74999096
Fe(Fragment=3)	1.20111096	-1.23988278	6.80137387
Fe(Fragment=1)	-3.42026757	-6.47081269	6.28184453
Fe(Fragment=2)	-6.69235164	0.56319604	6.64486907
Co(Fragment=4)	-1.42286537	-2.56456941	5.21306986
Co(Fragment=4)	-3.90999312	-1.07977193	5.09989752
Co(Fragment=4)	-3.99386930	-4.03781828	5.10785374
Co(Fragment=4)	-2.30862449	-1.16762628	7.55082842
Co(Fragment=4)	-2.58768043	-4.14601621	7.51454015
Co(Fragment=4)	-4.93764364	-2.35327351	7.40707012
Se(Fragment=4)	-3.03870430	-2.55761419	3.53007282
Se(Fragment=4)	-1.73035176	-0.28062219	5.44406494
Se(Fragment=4)	-1.77505950	-4.94866272	5.37232731
Se(Fragment=4)	-5.77183174	-2.47740389	5.23196240
Se(Fragment=4)	-0.61473448	-2.76863296	7.42605739
Se(Fragment=4)	-4.39290031	-0.06707849	7.18771090
Se(Fragment=4)	-4.87708635	-4.72668577	7.23264470
Se(Fragment=4)	-3.35534462	-2.57753522	9.11395842
H(Fragment=1)	-1.83920970	-12.42893690	8.91292704
H(Fragment=1)	-7.31071153	-11.65219942	4.53363024
H(Fragment=2)	-11.01311620	1.85206596	2.00077691
H(Fragment=2)	-2.22618063	5.52221877	3.81719119
H(Fragment=3)	3.51144326	4.71694208	7.83099878

```
 H(Fragment=3)      6.33772228    0.06565826    2.82432751
 H(Fragment=2)    -12.45566926    1.07814177    9.65620362
 C(Fragment=3)      2.80166242   -2.05432551    7.61858967
 C(Fragment=2)     -7.00574231    2.31927146    7.45293653
 C(Fragment=3)      5.07354101   -3.15061576    8.23198855
 H(Fragment=3)      4.95109775   -4.24431402    8.37257811
 H(Fragment=3)      5.79686958   -2.97043931    7.41006644
 H(Fragment=3)      5.48677981   -2.71802949    9.16591521
 C(Fragment=2)     -7.70809632    4.44947981    8.75817609
 H(Fragment=2)     -8.67492991    4.23844450    9.26057991
 H(Fragment=2)     -6.95766377    4.72941346    9.52625324
 H(Fragment=2)     -7.84708593    5.31055973    8.07280016
 N(Fragment=2)     -7.27904602    3.31726386    8.03447902
 N(Fragment=3)      3.83142643   -2.55415608    7.92906993

--link1--
%mem=115GB
%LindaWorker=
%usessh
%nprocshared=28
%chk=Fe3CNMe2+_aa_1_14_ubpy86_def2svp_opt.chk
#p opt ubvp86 chkbasis gfinput scf=(xqc,maxconventional=256) geom=allcheck guess=read
```

7. *trans*-(α,β)-[**1**$(CNMe)_2$]$^+$

```
%mem=115GB
%LindaWorker=n2095,n2096,n2097,n2098
%usessh
%nprocshared=28
%chk=Fe3CNMe2+_trans_ab_ubpv86_def2SVP_opt_modified.chk
#p ubvp86 def2svp gfinput scf=(xqc,maxconventional=256) guess=(fragment=4,only)

Fe3CNMe2+_trans_ab_ubpv86_def2SVP_opt_modified

1 14 0 5 0 5 0 5 1 2
 C(Fragment=1)      0.24416048    3.83747289   -6.81948324
 C(Fragment=3)     -3.07850111   -2.60954062   -6.64367214
 C(Fragment=1)      1.55282107    4.14304919   -6.41544471
 C(Fragment=3)     -4.20007145   -1.83614634   -6.30206013
 C(Fragment=2)      3.95365936   -0.49789150   -6.30435919
 C(Fragment=1)     -0.69017971    3.39245437   -5.86829962
 C(Fragment=2)      2.60165103   -0.87287260   -6.21704404
 C(Fragment=3)     -2.02731695   -2.75267528   -5.72308783
 C(Fragment=2)      4.81721121   -0.75881716   -5.22803541
 C(Fragment=1)      1.93011986    4.00308284   -5.06897268
 C(Fragment=3)     -4.26955340   -1.20579620   -5.04947846
 C(Fragment=1)      1.03795134    7.95439852   -3.47650667
 C(Fragment=1)     -0.31480166    3.24253909   -4.52545040
 C(Fragment=1)     -0.20939307    8.56511526   -3.26325489
 C(Fragment=2)      2.11661998   -1.50524391   -5.06126921
 C(Fragment=1)      1.00171523    3.54275902   -4.11067523
```

C(Fragment=3)	-2.09249987	-2.11765061	-4.47206992
C(Fragment=1)	1.32224485	6.69272269	-2.93290058
C(Fragment=3)	-3.21166949	-1.33439453	-4.11963021
C(Fragment=3)	-4.73710105	2.97646718	-4.06030693
C(Fragment=2)	4.33525412	-1.39756625	-4.07203060
C(Fragment=3)	-7.56339940	-3.20796972	-2.84458217
C(Fragment=3)	-7.08579947	-4.42549847	-3.35871201
C(Fragment=3)	-4.00369205	1.80482585	-3.81790187
C(Fragment=1)	5.49678851	3.14128798	-2.78593377
C(Fragment=2)	2.98316626	-1.78595068	-3.98081582
C(Fragment=1)	5.98100040	4.12710058	-1.90844279
C(Fragment=1)	4.11569115	2.92447958	-2.91466804
C(Fragment=1)	-1.18037650	7.89212653	-2.50042285
C(Fragment=3)	-5.85794590	3.28501882	-3.26906896
C(Fragment=2)	1.24223751	-5.94996664	-4.62420739
C(Fragment=3)	-6.67704809	-2.28338845	-2.27000333
C(Fragment=2)	1.74753655	-4.68022082	-4.29426250
C(Fragment=1)	0.35178479	6.00451963	-2.16003939
C(Fragment=1)	5.07785856	4.89186646	-1.15198219
C(Fragment=1)	3.20210412	3.71526318	-2.18057584
C(Fragment=3)	-5.71079546	-4.71412448	-3.28845423
C(Fragment=3)	-4.38505363	0.92379871	-2.78181730
C(Fragment=1)	3.69454740	4.68635503	-1.28458085
C(Fragment=1)	-0.90872003	6.62714308	-1.96430393
C(Fragment=2)	6.34615639	-5.19510542	-3.68554554
C(Fragment=3)	-5.28886258	-2.56152279	-2.19267097
C(Fragment=2)	5.54316520	-4.06164748	-3.48848939
C(Fragment=3)	-4.82153451	-3.79757246	-2.70982135
C(Fragment=2)	0.64845219	-6.75335656	-3.63811419
C(Fragment=3)	-6.23515519	2.41734278	-2.23119241
C(Fragment=2)	1.65162060	-4.19611571	-2.97171233
C(Fragment=3)	-5.49731531	1.24657344	-1.98112601
C(Fragment=2)	6.43659692	-6.19286131	-2.69959660
C(Fragment=2)	4.80116367	-3.89763559	-2.28919265
C(Fragment=2)	0.56492915	-6.28465543	-2.31545061
C(Fragment=2)	1.05895374	-5.01497466	-1.98414551
C(Fragment=2)	5.69522930	-6.05181195	-1.51349317
C(Fragment=1)	-2.79359316	7.65741355	3.09337150
C(Fragment=2)	4.88129224	-4.92730109	-1.31490754
C(Fragment=1)	-2.62633670	6.30170955	2.77245234
C(Fragment=1)	-1.91593192	8.63388772	2.59367338
C(Fragment=1)	-1.55801673	5.88413520	1.93685996
C(Fragment=3)	-5.26615205	-4.67227756	0.92320910
C(Fragment=3)	-6.26788856	-5.59399033	1.25991098
C(Fragment=1)	-4.41452932	3.09291301	1.50441589
C(Fragment=1)	-0.85240182	8.23318338	1.76524067
C(Fragment=1)	-5.80727151	3.18858919	1.64093146
C(Fragment=1)	-0.67120660	6.88172856	1.44797310
C(Fragment=2)	6.79100947	-2.08132980	2.02334222
C(Fragment=3)	-4.30296460	-4.23915446	1.87929811
C(Fragment=3)	-1.49536143	-5.68042709	1.41429989

C(Fragment=3)	-0.80828102	-6.90629450	1.38270970
C(Fragment=2)	8.03512931	-2.48392598	2.52620279
C(Fragment=2)	5.57967063	1.79490612	2.21505689
C(Fragment=2)	6.22012343	3.03730004	2.35231814
C(Fragment=1)	-3.58205479	3.10363312	2.64550687
C(Fragment=2)	6.18969212	-0.85508171	2.42149075
C(Fragment=3)	-6.32323023	-6.14227347	2.55199367
C(Fragment=3)	-0.98227225	-4.59393773	2.15464104
C(Fragment=2)	4.29150821	1.59302405	2.75345660
C(Fragment=2)	5.58013329	4.09461300	3.01931572
C(Fragment=3)	0.40447562	-7.06025719	2.07377059
C(Fragment=1)	-6.39193797	3.27542061	2.91697336
C(Fragment=2)	3.63635822	2.67338152	3.38898933
C(Fragment=2)	4.28430051	3.91087721	3.53075307
C(Fragment=3)	-4.36646183	-4.81232747	3.17799075
C(Fragment=3)	0.25585090	-4.74691831	2.82099982
C(Fragment=3)	0.93836419	-5.97350979	2.78733201
C(Fragment=2)	8.72537771	-1.68453556	3.45554381
C(Fragment=1)	-1.01097104	3.20992799	4.06283199
C(Fragment=1)	-0.14612895	4.27037065	4.40725385
C(Fragment=3)	-5.35871548	-5.75034183	3.49760628
C(Fragment=2)	6.89794117	-0.06311158	3.36749198
C(Fragment=1)	-4.17527993	3.18432717	3.92488805
C(Fragment=1)	-5.57241021	3.26518727	4.05757655
C(Fragment=2)	8.13998756	-0.47734010	3.87146616
C(Fragment=3)	-2.14793770	-2.53127855	3.93597148
C(Fragment=2)	3.07706690	-0.68154355	4.22065701
C(Fragment=3)	-3.04012516	-1.46587585	4.19446487
C(Fragment=2)	2.89249203	-2.07757862	4.33826830
C(Fragment=1)	0.44507575	4.31596117	5.68213432
C(Fragment=1)	-1.24659866	2.18152759	5.00390742
C(Fragment=2)	3.00046769	0.11432603	5.38207352
C(Fragment=3)	-1.58627785	-3.22065089	5.03150371
C(Fragment=3)	-3.36299180	-1.10237156	5.51035233
C(Fragment=2)	2.63594997	-2.66171927	5.58751960
C(Fragment=1)	0.18820635	3.30310778	6.62057191
C(Fragment=1)	-0.65584090	2.23305803	6.27613303
C(Fragment=2)	2.73884223	-0.47414003	6.63142602
C(Fragment=3)	-1.90472182	-2.84917717	6.34985166
C(Fragment=3)	-2.79232733	-1.79062633	6.59598560
C(Fragment=2)	2.55431723	-1.86096174	6.73964023
H(Fragment=1)	-0.05057144	3.95055185	-7.87397236
H(Fragment=3)	-3.02618025	-3.10445134	-7.62560905
H(Fragment=3)	-5.03332231	-1.72488780	-7.01262907
H(Fragment=2)	4.33166259	-0.00100871	-7.21085823
H(Fragment=1)	2.28922947	4.50299140	-7.15021150
H(Fragment=2)	1.91520818	-0.66596810	-7.05198345
H(Fragment=1)	-1.72004641	3.15697190	-6.17743878
H(Fragment=1)	1.80857825	8.46916706	-4.07114651
H(Fragment=3)	-1.14931572	-3.36716160	-5.97424048
H(Fragment=2)	5.87784026	-0.46948517	-5.28922005

H(Fragment=3)	-5.15969878	-0.61253355	-4.79495890
H(Fragment=1)	2.95534274	4.26233798	-4.76949379
H(Fragment=3)	-4.43502910	3.65308637	-4.87434042
H(Fragment=1)	2.31109114	6.24214811	-3.09290424
H(Fragment=3)	-8.63786380	-2.97217204	-2.89512805
H(Fragment=3)	-3.13537691	1.56235732	-4.45057144
H(Fragment=2)	1.05195144	-1.77674354	-5.00656291
H(Fragment=1)	-1.05374521	2.89674638	-3.78581923
H(Fragment=1)	6.19810151	2.53337726	-3.37779818
H(Fragment=2)	1.32572990	-6.31523479	-5.65939854
H(Fragment=2)	2.23032141	-4.07343202	-5.07350670
H(Fragment=1)	7.06427370	4.29891299	-1.81354243
H(Fragment=1)	3.75237930	2.14107752	-3.59767768
H(Fragment=3)	-1.26550912	-2.24640723	-3.75789862
H(Fragment=2)	5.01062774	-1.60103362	-3.22850919
H(Fragment=1)	-2.16580343	8.35155536	-2.32806718
H(Fragment=2)	6.91277492	-5.29686820	-4.62438969
H(Fragment=3)	-7.04892227	-1.32656006	-1.87627812
H(Fragment=3)	-5.32663241	-5.66633870	-3.68575440
H(Fragment=1)	5.44920928	5.65968456	-0.45668605
H(Fragment=2)	5.49333185	-3.28775384	-4.26727976
H(Fragment=1)	2.98555803	5.28830972	-0.69678271
H(Fragment=2)	0.25662384	-7.74862419	-3.89813654
H(Fragment=1)	-1.68083046	6.09312004	-1.38733311
H(Fragment=3)	-3.74706877	-4.02311181	-2.64270342
H(Fragment=3)	-7.12046973	2.64793832	-1.61802420
H(Fragment=1)	-3.63179806	7.95238510	3.74379072
H(Fragment=3)	-5.78269546	0.56819144	-1.16300608
H(Fragment=1)	-3.33559894	5.56338266	3.16557848
H(Fragment=2)	5.73614211	-6.83438219	-0.73979291
H(Fragment=2)	0.11191608	-6.90843042	-1.53062388
H(Fragment=2)	0.99067671	-4.66188471	-0.94353139
H(Fragment=2)	4.26361898	-4.83884218	-0.40822759
H(Fragment=3)	-5.19050636	-4.30850357	-0.11514308
H(Fragment=3)	-6.99422264	-5.90182168	0.49208179
H(Fragment=1)	-3.96944259	3.02435461	0.49914359
H(Fragment=2)	6.25348316	-2.73534358	1.32084412
H(Fragment=1)	-6.43685797	3.20370377	0.73890742
H(Fragment=3)	-2.43838042	-5.57181600	0.86214918
H(Fragment=2)	8.45989406	-3.44574882	2.19785205
H(Fragment=3)	-1.23384883	-7.75026125	0.81814269
H(Fragment=2)	6.08360438	0.96998810	1.69196417
H(Fragment=2)	7.23013669	3.17708180	1.93774335
H(Fragment=1)	-0.14902255	8.97937982	1.36495808
H(Fragment=1)	0.17675844	6.57389705	0.81500429
H(Fragment=1)	0.06148193	5.06950126	3.68274216
H(Fragment=2)	6.08674155	5.06514944	3.13369055
H(Fragment=3)	0.93817752	-8.02251506	2.05035632
H(Fragment=1)	-7.48439916	3.36764537	3.02136210
H(Fragment=3)	-3.50050000	-0.92375635	3.35340566
H(Fragment=2)	2.60770874	2.56413147	3.76473505

H(Fragment=2)	2.97661847	-2.71145208	3.44236154
H(Fragment=2)	3.76606037	4.73918822	4.03731258
H(Fragment=3)	0.70088960	-3.89799929	3.36102064
H(Fragment=3)	1.89766882	-6.07892079	3.31691337
H(Fragment=3)	-3.64183461	-4.51570268	3.94557021
H(Fragment=2)	6.47691603	0.88950070	3.71376796
H(Fragment=1)	-3.54685829	3.21628014	4.82627066
H(Fragment=3)	-5.37941223	-6.17864848	4.51208653
H(Fragment=1)	1.10395390	5.15813090	5.94393736
H(Fragment=1)	-1.88786595	1.32478373	4.74651917
H(Fragment=1)	-6.01809620	3.34123828	5.06152377
H(Fragment=2)	8.65713676	0.16176903	4.60410606
H(Fragment=2)	3.16557010	1.19857818	5.33035491
H(Fragment=3)	-0.91607877	-4.07525131	4.86770588
H(Fragment=3)	-4.07213260	-0.27902052	5.68780964
H(Fragment=2)	2.51375015	-3.75308519	5.66467883
H(Fragment=1)	0.64772546	3.34572314	7.61999152
H(Fragment=1)	-0.85087788	1.42432383	6.99639560
H(Fragment=2)	2.69046663	0.16203404	7.52837014
H(Fragment=3)	-1.45952768	-3.40398170	7.18993546
H(Fragment=3)	-3.04801360	-1.50934418	7.62905320
H(Fragment=2)	2.35940048	-2.31953058	7.72103114
N(Fragment=1)	0.58543437	4.74693201	-1.56367319
N(Fragment=3)	-4.39508630	-1.65460096	-1.55440983
N(Fragment=2)	4.00198252	-2.76603636	-2.01927344
N(Fragment=1)	-1.31876798	4.54847177	1.55375851
N(Fragment=3)	-3.39542043	-3.23634201	1.46328968
N(Fragment=2)	4.95276931	-0.49881464	1.85494137
P(Fragment=1)	1.38324010	3.41575350	-2.30152037
P(Fragment=3)	-3.37274165	-0.59454427	-2.42397473
P(Fragment=2)	2.35145988	-2.57212557	-2.42210544
P(Fragment=1)	-1.74239346	3.10539507	2.36712578
P(Fragment=3)	-1.84899646	-2.95732260	2.13772533
P(Fragment=2)	3.44402996	-0.03522772	2.51288722
Fe(Fragment=1)	-0.32535798	3.97580521	-0.02810794
Fe(Fragment=2)	4.75648767	-0.93580864	-0.78007768
Fe(Fragment=3)	-4.39296663	-1.89684717	0.38986410
Co(Fragment=4)	0.67068465	1.68272125	-1.16043690
Co(Fragment=4)	-1.58398920	-0.11469597	-1.29576993
Co(Fragment=4)	1.10263007	-1.23297585	-1.24008627
Co(Fragment=4)	-0.93865970	1.52942619	1.10101353
Co(Fragment=4)	1.78938688	0.09493460	1.11071874
Co(Fragment=4)	-0.75454525	-1.42732040	1.04810444
Se(Fragment=4)	0.11380793	0.16205697	-2.85920186
Se(Fragment=4)	-1.66818405	2.27764775	-1.09040136
Se(Fragment=4)	2.75461489	0.46373590	-1.04332365
Se(Fragment=4)	-0.97698157	-2.33133813	-1.08573654
Se(Fragment=4)	1.25898867	2.44079248	1.02965742
Se(Fragment=4)	-2.66802503	-0.11192872	0.79784298
Se(Fragment=4)	1.57365355	-2.16405414	0.83456633
Se(Fragment=4)	0.00949518	0.04503416	2.67319669

```
H(Fragment=2)      9.69856419   -2.00347776    3.85743943
H(Fragment=2)      7.07030808   -7.07806863   -2.85933763
H(Fragment=3)     -7.78188636   -5.14744885   -3.81184588
H(Fragment=3)     -6.43710642    4.20026673   -3.46449785
H(Fragment=1)     -2.05611012    9.69474723    2.84897329
H(Fragment=1)     -0.42479416    9.55584038   -3.69057903
H(Fragment=3)     -7.09809797   -6.87739295    2.81596228
C(Fragment=3)     -6.01826443   -1.26049210    1.28880291
C(Fragment=3)     -8.17451789   -0.61831075    2.58388479
H(Fragment=3)     -8.34462964   -1.35266938    3.39706513
H(Fragment=3)     -8.07049810    0.39315772    3.02679014
H(Fragment=3)     -9.05529812   -0.62945495    1.91030954
N(Fragment=3)     -7.00766983   -0.96155136    1.86556129
C(Fragment=2)      6.22003888    0.17418850   -1.73724058
C(Fragment=2)      8.61609720    0.88684075   -2.43246530
H(Fragment=2)      8.74687989    1.98668384   -2.39337934
H(Fragment=2)      9.31746367    0.41188868   -1.71621482
H(Fragment=2)      8.84982727    0.53321383   -3.45635387
N(Fragment=2)      7.28777654    0.53801257   -2.09006924

--link1--
%mem=115GB
%LindaWorker=n2095,n2096,n2097,n2098
%usessh
%nprocshared=28
%chk=Fe3CNMe2+_trans_ab_ubpv86_def2SVP_opt_modified.chk
#p opt ubvp86 chkbasis gfinput scf=(xqc,maxconventional=256) geom=allcheck guess=read
```

8. *cis*-(α,β)-**1**(CNMe)$_2$

```
%mem=128GB
%nprocshared=28
%chk=Fe3CNMe2_cis_ab_0_13_ubpv86_def2svp_opt.chk
# opt ubvp86 pop=(full,nbo) def2svp gfinput scf=maxcycle=5000

Fe3CNMe2_cis_ab_0_13_ubpv86_def2svp_opt

0 13
 C              -2.32543641    2.58383560   -6.58719660
 C              -2.14763533   -2.84258812   -6.88973501
 C              -0.93001785    2.71135166   -6.51057893
 C              -3.27597772   -2.06527525   -6.58652041
 C               3.26848841    0.51858352   -6.51987356
 C              -3.09403425    2.69554677   -5.41609342
 C               2.21767761   -0.37795639   -6.25571801
 C              -1.35343744   -3.34795510   -5.84502588
 C               4.23631155    0.76279833   -5.53166611
 C              -0.30371418    2.94655408   -5.27454831
 C              -3.61259021   -1.79276954   -5.24900844
 C              -2.19532579    7.26705553   -3.93690474
 C              -2.47097948    2.92507376   -4.18012280
```

C	-3.29073113	7.85688893	-3.28523148
C	2.13726586	-1.02823472	-5.01395768
C	-1.06669795	3.05423456	-4.09382370
C	-1.68052320	-3.06736135	-4.51032373
C	-1.56656813	6.12724023	-3.41343479
C	-2.81573735	-2.28660455	-4.19556893
C	-6.25379461	0.73731466	-2.88725089
C	4.16028190	0.10645191	-4.29001117
C	-5.43914931	-5.82311683	-3.91276421
C	-5.01658153	-7.08596621	-3.46568501
C	-5.00140370	0.10343899	-2.93171177
C	3.52644171	4.28304465	-3.58715061
C	3.11891010	-0.80448321	-4.02203397
C	3.68266808	5.60741910	-3.14227565
C	2.32336274	3.59610507	-3.35946349
C	-3.75581507	7.27091038	-2.09470164
C	-7.34619134	0.10412673	-2.26880020
C	3.43580977	-5.31308606	-4.31203540
C	-4.91445096	-4.64664889	-3.35610567
C	3.33133051	-3.93110044	-4.07841173
C	-2.01571185	5.52546341	-2.20295077
C	2.63003635	6.23742478	-2.45874073
C	1.24491774	4.23614256	-2.70657229
C	-4.04478110	-7.15603224	-2.45081252
C	-4.83068616	-1.18115809	-2.36735577
C	1.41951930	5.55690506	-2.24123749
C	-3.13669931	6.12992305	-1.57028073
C	7.41491152	-3.11854988	-2.85136916
C	-3.93425525	-4.70146664	-2.32895926
C	6.21768758	-2.38714914	-2.82249984
C	-3.50718319	-5.98757554	-1.89850778
C	3.35331555	-6.21939184	-3.24337866
C	-7.17645949	-1.16309811	-1.68620848
C	3.13162147	-3.43786510	-2.77101577
C	-5.92431160	-1.80063330	-1.72743916
C	8.04286722	-3.52079474	-1.65951430
C	5.60513946	-2.03740372	-1.58729872
C	3.16618673	-5.73695523	-1.93679626
C	3.04955072	-4.35908219	-1.70253593
C	7.44407786	-3.19454242	-0.43016209
C	-6.49580874	4.82477118	0.51755890
C	6.23988159	-2.47684942	-0.39415896
C	-5.26450060	4.15508352	0.47439629
C	-7.07492498	5.17456441	1.75010649
C	-4.55329709	3.83632113	1.66311562
C	-4.02987096	-5.73045979	1.56918352
C	-4.89634817	-6.70770830	2.07257860
C	-5.28422014	0.52992013	1.72547496
C	-6.40346377	4.83506827	2.93768759
C	-6.53275892	-0.07061795	1.94279051
C	-5.16395812	4.17857915	2.90143451

C	7.05319426	0.27792652	1.66301182
C	-2.69981431	-5.60120243	2.05465950
C	0.74937060	-6.25608738	1.50632501
C	1.83054336	-7.15249406	1.44952931
C	8.32854011	0.29050890	2.24092802
C	4.18252844	3.56677287	2.78639998
C	4.15636099	4.90748269	3.20723277
C	-4.49767049	0.96715748	2.81555267
C	5.89994669	0.71295503	2.37396408
C	-4.47440278	-7.58222186	3.09074726
C	0.88357129	-5.01941902	2.17115410
C	3.11706022	2.69421834	3.09572747
C	3.06410331	5.39639808	3.94187167
C	3.05852448	-6.81941317	2.04528551
C	-7.01227351	-0.25546786	3.25078692
C	2.00342781	3.20641818	3.79975656
C	1.98661362	4.54227334	4.23221217
C	-2.28824235	-6.48986460	3.08407726
C	2.12836530	-4.67840884	2.74682175
C	3.20514506	-5.57792546	2.68906992
C	8.51317244	0.73257893	3.56264290
C	-2.22672891	2.28516349	4.08335854
C	-2.00851121	3.64551235	4.38129333
C	-3.16767028	-7.45881482	3.59112305
C	6.10808417	1.15033896	3.71343455
C	-4.99304249	0.79039043	4.12484770
C	-6.23995361	0.17765209	4.33983478
C	7.38812488	1.15705517	4.28783450
C	-0.96351404	-3.65649143	4.01384159
C	3.22007225	-0.10104634	4.04698761
C	-2.16608084	-2.98077498	4.32206828
C	3.89174604	-1.34100221	3.96693503
C	-1.50497580	4.03098711	5.63647119
C	-1.89901011	1.31237821	5.05560137
C	2.63097861	0.27167327	5.27293896
C	-0.17758088	-4.16206882	5.06955546
C	-2.57240400	-2.81836530	5.65456085
C	3.97821178	-2.18161889	5.08780171
C	-1.20065195	3.06015050	6.60525096
C	-1.39573045	1.69935814	6.30854043
C	2.71426068	-0.57426571	6.39188024
C	-0.58164329	-3.98790167	6.40491583
C	-1.77799959	-3.31722507	6.70238901
C	3.38748260	-1.80290872	6.30505151
H	-2.81445305	2.40449878	-7.55700561
H	-1.89077705	-3.06177106	-7.93777743
H	-3.91056907	-1.67170316	-7.39575551
H	3.33002758	1.02820749	-7.49392393
H	-0.31627172	2.63257352	-7.42127207
H	1.44609071	-0.56896450	-7.01694070
H	-4.19084471	2.61348117	-5.46666742

H	-1.81537658	7.69826276	-4.87680940
H	-0.47328842	-3.97068675	-6.06813690
H	5.06278369	1.46370717	-5.72826106
H	-4.51352826	-1.20119603	-5.03265978
H	0.78856507	3.05832695	-5.24616337
H	-6.37651208	1.73602652	-3.33424174
H	-0.71580747	5.69942477	-3.95883949
H	-6.19660626	-5.74557876	-4.70890219
H	-4.15001191	0.61318242	-3.40813244
H	1.29797924	-1.71302910	-4.81899037
H	-3.07862157	3.02241539	-3.26803616
H	4.34950337	3.77173117	-4.10897866
H	3.59043435	-5.67980690	-5.33882000
H	3.41563096	-3.23559016	-4.92552159
H	4.62572453	6.14529205	-3.32593096
H	2.23802069	2.54747770	-3.68252778
H	-1.05066926	-3.46661575	-3.69911252
H	4.91265654	0.29989937	-3.51214192
H	-8.32729216	0.60239060	-2.23578659
H	-4.62270271	7.69892830	-1.56645966
H	7.86475674	-3.37459287	-3.82381433
H	-5.27223239	-3.67397084	-3.71910083
H	-3.69269608	-8.13407827	-2.08710604
H	2.74289937	7.27258586	-2.10034089
H	5.74965983	-2.07228472	-3.76569809
H	0.59328655	6.06063740	-1.72006311
H	3.43554147	-7.30177988	-3.42764739
H	-3.53200542	5.66897665	-0.65579007
H	-2.73241794	-6.05135129	-1.11732245
H	-8.02675657	-1.66682409	-1.20041311
H	-7.01642025	5.05804334	-0.42461319
H	-5.78873226	-2.78971604	-1.26587463
H	-4.83691760	3.83604248	-0.48775593
H	7.90950899	-3.51458480	0.51520310
H	3.09944625	-6.43311600	-1.08758960
H	2.89692059	-3.99595216	-0.67431924
H	5.75060594	-2.25730813	0.56682185
H	-4.37373382	-5.03734803	0.78465901
H	-5.91772425	-6.78065994	1.66725735
H	-4.92084171	0.66704211	0.69468597
H	6.93216751	-0.09412675	0.63631808
H	-7.12744767	-0.39986110	1.07800550
H	-0.21017342	-6.51422946	1.03506308
H	9.18776373	-0.05990827	1.64728216
H	1.70721991	-8.11984510	0.93814532
H	5.04846426	3.18696417	2.22687921
H	5.00405255	5.56832126	2.96735966
H	-6.84900847	5.08762970	3.91305601
H	-4.65294224	3.93105053	3.84238975
H	-2.23826431	4.40243045	3.61757005
H	3.04856474	6.44300114	4.28378394

H	3.90450330	-7.52274244	2.00269511
H	-7.98921266	-0.73411617	3.42005448
H	-2.79544173	-2.58530183	3.50887829
H	1.12667502	2.57120358	3.99882108
H	4.36578056	-1.63959507	3.02063846
H	1.11391097	4.91389758	4.78912103
H	2.26323950	-3.70287955	3.23903691
H	4.16912390	-5.30448232	3.14532099
H	-1.26944166	-6.42411713	3.48888017
H	5.25981749	1.49179731	4.31954101
H	-4.41264984	1.14331652	4.98903427
H	-2.81793952	-8.13122325	4.39058705
H	-1.35807174	5.09962635	5.85900160
H	-2.03440073	0.24173318	4.83920923
H	-6.61151578	0.04885245	5.36847081
H	7.50063733	1.50347612	5.32749042
H	2.11070132	1.23373050	5.37309058
H	0.75177136	-4.70928311	4.85709225
H	-3.52005771	-2.30216200	5.87232544
H	4.52469942	-3.13450698	5.01263167
H	-0.80949192	3.36191533	7.58921451
H	-1.15418478	0.92822076	7.05606087
H	2.24965271	-0.26404056	7.34053539
H	0.04390358	-4.39027086	7.21680571
H	-2.09649555	-3.18979352	7.74870981
H	3.45817002	-2.46074129	7.18487521
N	-1.43120120	4.39140938	-1.60871991
N	-3.37934231	-3.56987858	-1.70769439
N	4.43434920	-1.25207826	-1.48998920
N	-3.29513812	3.21234744	1.56117529
N	-1.87305159	-4.61930620	1.48363112
N	4.65073724	0.68958477	1.71827918
P	-0.35007314	3.33282184	-2.39316959
P	-3.17801484	-2.01463795	-2.38918459
P	2.99322603	-1.63189285	-2.35889588
P	-2.88039831	1.78789595	2.41422034
P	-0.54150154	-3.83705662	2.21050244
P	3.14579260	0.93181201	2.49351020
Fe	-1.83331445	3.59852679	0.22659938
Fe	4.39851215	0.29952303	-0.24285523
Fe	-2.21866295	-3.51654901	-0.11405782
Co	-0.12264462	1.59036310	-1.12986738
Co	-1.63946048	-1.07151547	-1.17945223
Co	1.26696940	-0.98768054	-1.21500684
Co	-1.43302276	0.74732056	1.17380189
Co	1.48908634	0.47501484	1.17273129
Co	-0.26357258	-2.01983528	1.05768956
Se	-0.21878600	-0.09533668	-2.77105963
Se	-2.42294213	1.19716720	-0.89855660
Se	2.23623046	1.13386359	-0.99117544
Se	-0.10628377	-2.96217559	-1.16557908

Se	0.18213217	2.50279418	1.07207171
Se	-2.58314855	-1.38550824	0.98835203
Se	2.07029164	-1.74316288	0.83685023
Se	-0.06205697	-0.34894562	2.71075779
H	-5.43519365	-8.00429369	-3.90443048
H	-5.15735600	-8.34678800	3.49083250
H	8.98336103	-4.09153151	-1.68920697
H	9.51396531	0.73996671	4.02006387
H	-8.04501816	5.69306501	1.78539216
H	-3.77913081	8.75060935	-3.70202655
C	5.77066631	1.66919091	-1.05424032
C	-1.44510112	5.48695507	1.08356207
C	7.52690872	3.42225110	-2.09274528
H	7.36419982	4.38794143	-1.66162117
H	8.52374039	3.09870085	-1.87700965
H	7.39136534	3.47742462	-3.15269050
C	-0.95012821	7.89470562	2.17619154
H	-0.01100830	8.26234716	1.81870936
H	-1.72767375	8.57706350	1.90285906
H	-0.91481751	7.80243554	3.24162074
N	-1.23349560	6.51629081	1.55067132
N	6.52147626	2.41864043	-1.49821084

$NBO PLOT FILE=Fe3CNMe2_cis_ab_0_13_ubpv86_def2svp_opt $END

Example of analytical frequency calculation input:

```
%mem=115GB
%LindaWorker=
%usessh
%nprocshared=28
%rwf=Fe3CNMeTHF2+_aab_TS_1_14_ubpv86_def2svp_freq.rwf
%NoSave
%oldchk=Fe3CNMeTHF2+_aab_ubpv86_def2SVP_optimized_optts_calcFC_retry.chk
%chk=Fe3CNMeTHF2+_aab_TS_1_14_ubpv86_def2svp_freq.chk
#p freq ubvp86 def2svp scf=(maxcycle=5000) Geom=Checkpoint Guess=Read

Fe3CNMeTHF2+_aab_TS_1_14_ubpv86_def2svp_freq

1 14

$NBO PLOT FILE=Fe3CNMeTHF2+_aab_TS_1_14_ubpv86_def2svp_freq $END
```

Example of single-point electronic calculation:

```
%mem=128GB
%nprocshared=28
%RWF=Fe3CNMe2+_cis_ab_ub3lyp+_ccPVTZ.rwf
%NoSave
%oldchk=Fe3CNMe2+_cis_ab_ubpv86_def2SVP_opt.chk
%chk=Fe3CNMe2+_cis_ab_ub3lyp+_ccPVTZ.chk
#p ub3lyp IOp(3/76=1000001500) IOp(3/77=0720008000) IOp(3/78=0810010000) cc-pVTZ
guess=read geom=check scf=maxcycle=3072 gfinput pop=full IOp(6/82=1)
```

```
Fe3CNMe2+_cis_ab_ub3lyp+_ccPVTZ

1 14

$NBO PLOT FILE=Fe3CNMe2+_cis_ab_ub3lyp+_ccPVTZ $END
```

Example of interactive batch file:

```
#!/bin/bash
#SBATCH --job-name=Fe3THF2+_cis_ab_ubpv86_def2SVP_freq
#SBATCH --nodes=2
#SBATCH --cpus-per-task=28
#SBATCH --time=48:00:00
#SBATCH --mem=115G
#SBATCH --chdir=/gscratch/EXAMPLE-DIRECTORY
#SBATCH --mail-type=FAIL,END
#SBATCH --mail-user=EXAMPLE-EMAIL@uw.edu

#SBATCH --partition=EXAMPLE-NODE
#SBATCH --account=EXAMPLE-NODE

# load Gaussian environment
module load contrib/g16.b01
export inputfile='Fe3THF2+_cis_ab_ubpv86_def2SVP_freq.gjf'


# debugging information
echo "**** Job Debugging Information ****"
echo "This job will run on $SLURM_JOB_NODELIST"
echo ""
echo "ENVIRONMENT VARIABLES"
set
echo "**********************************************"

# local scratch
export GAUSS_SCRDIR='/gscratch/EXAMPLE-DIRECTORY'


## Memory
gbmem=`expr $SLURM_MEM_PER_NODE / 1000`
gbmem=`expr $gbmem - 10`
echo "Parsed memory: $gbmem"
sed -i "/mem/s/.*/%mem=${gbmem}GB/" $inputfile


## Set number of threads
export num_threads=$(echo $SLURM_JOB_CPUS_PER_NODE| cut -f1 -d"(" )
sed -i "/nproc/s/.*/%nprocshared=${num_threads}/" $inputfile


# add linda nodes
```

```
nodes=()
nodes+=(`scontrol show hostnames $SLURM_JOB_NODELIST `)
for ((i=0; i<${#nodes[*]}-1; i++));
do
    string+=${nodes[$i]}
    string+=","
done
string+=${nodes[$SLURM_NNODES-1]}
sed -i -e "s/\%LindaWorker.*/\%LindaWorker=$string/gI" "$inputfile"

# check that the Linda nodes are correct
lindaline=(`grep -i 'lindaworker' $inputfile`)
if [[ $lindaline == *$string ]]
then
    echo "Using the correct nodes for Linda"
else
    echo "Using the wrong nodes for Linda"
    echo "Nodes assigned by scheduler = $string"
    echo "Line in Gaussian input file = $lindaline"
    exit 1
fi

# run Gaussian
g16 $inputfile

exit 0
```

S8 References

(1) Kephart, J. A.; Mitchell, B. S.; Chirila, A.; Anderton, K. J.; Rogers, D.; Kaminsky, W.; Velian, A. Atomically Defined Nano-Propeller $Fe_3Co_6Se_8(Ph_2PNTol)_6$: Functional Model for the Electronic Metal-Support Interaction Effect, and High Catalytic Activity for Carbodiimide Formation. *J. Am. Chem. Soc.* **2019**, *141* (50), 19605–19610. https://doi.org/10.1021/jacs.9b12473.

(2) Greenwood, B. P.; Forman, S. I.; Rowe, G. T.; Chen, C.-H.; Foxman, B. M.; Thomas, C. M. Multielectron Redox Activity Facilitated by Metal-Metal Interactions in Early/Late Heterobimetallics: Co/Zr Complexes Supported by Phosphinoamide Ligands. *Inorg. Chem* **2009**, *48*, 6251–6260. https://doi.org/10.1021/ic900552b.

(3) Bain, G. A.; Berry, J. F. Diamagnetic Corrections and Pascal's Constants. *J. Chem. Ed.* **2008**, *85* (4), 532. https://doi.org/10.1021/ed085p532.

(4) Chilton, N. F.; Anderson, R. P.; Turner, L. D.; Soncini, A.; Murray, K. S. PHI: A Powerful New Program for the Analysis of Anisotropic Monomeric and Exchange-Coupled Polynuclear *d* - and *f* - Block Complexes. *J. Comput. Chem.* **2013**, *34* (13), 1164–1175. https://doi.org/10.1002/jcc.23234.

(5) Bruker APEX2 (Version 2.1-4), SAINT (Version 7.34A), SADABS (Version 2007/4), 2007 BrukerAXS Inc, Madison, Wisconsin, USA.

(6) Sheldrick, G. M. *Acta Cryst.* **2015**, *A71*, 3-8.

(7) Altomare, A.; Burla, C.; Camalli, M.; Cascarano, G. L.; Giacovazzo, C.; Guagliardi, A.; Moliterni, A.G.G.; Polidori, G.; Spagna, R. *J. Appl. Crystallogr.*, **1999**, *32*, 115-119.

(8) Altomare, A.; Cascarano, G. L.; Giacovazzo, C.; Guagliardi, A. *J. Appl. Crystallogr.*, **1993**, *26*, 343-350.

(9) Sheldrick, G. M. *SHELXL-97, Program for the Refinement of Crystal Structures*, 1997, University of Göttingen, Germany.
(10) Sheldrick, G. M. *Acta Cryst.,* **2015**, *C71*, 3–8.
(11) Mackay, S.; Edwards, C.; Henderson, A.; Gilmore, C.; Stewart, N.; Shankland, K.; Donald, A. *MaXus: A Computer Program for the Solution and Refinement of Crystal Structures from Diffraction Data*, 1997, University of Glasgow, Scotland.
(12) Waasmaier, D.; Kirfel, A. *Acta Cryst*., **1995**, *51*, 416-430.
(13) Frisch, M. J.; Trucks, G. W.; Schlegel, H. B.; Scuseria, G. E.; Robb, M. A.; Cheeseman, J. R.; Scalmani, G.; Barone, V.; Petersson, G. A.; Nakatsuji, H.; Li, X.; Caricato, M.; Marenich, A. V.; Bloino, J.; Janesko, B. G.; Gomperts, R.; Mennucci, B.; Hratchian, H. P.; Ortiz, J. V.; Izmaylov, A. F.; Sonnenberg, J. L.; Williams; Ding, F.; Lipparini, F.; Egidi, F.; Goings, J.; Peng, B.; Petrone, A.; Henderson, T.; Ranasinghe, D.; Zakrzewski, V. G.; Gao, J.; Rega, N.; Zheng, G.; Liang, W.; Hada, M.; Ehara, M.; Toyota, K.; Fukuda, R.; Hasegawa, J.; Ishida, M.; Nakajima, T.; Honda, Y.; Kitao, O.; Nakai, H.; Vreven, T.; Throssell, K.; Montgomery Jr., J. A.; Peralta, J. E.; Ogliaro, F.; Bearpark, M. J.; Heyd, J. J.; Brothers, E. N.; Kudin, K. N.; Staroverov, V. N.; Keith, T. A.; Kobayashi, R.; Normand, J.; Raghavachari, K.; Rendell, A. P.; Burant, J. C.; Iyengar, S. S.; Tomasi, J.; Cossi, M.; Millam, J. M.; Klene, M.; Adamo, C.; Cammi, R.; Ochterski, J. W.; Martin, R. L.; Morokuma, K.; Farkas, O.; Foresman, J. B.; Fox, D. J. *Gaussian 16 Rev. A.03*; Wallingford, CT, 2016.
(14) Becke, A. D. Density-Functional Exchange-Energy Approximation with Correct Asymptotic Behavior. *Phys. Rev. A* **1988**, *38* (6), 3098–3100. https://doi.org/10.1103/PhysRevA.38.3098.
(15) Perdew, J. P. Density-Functional Approximation for the Correlation Energy of the Inhomogeneous Electron Gas. *Phys. Rev. B* **1986**, *33* (12), 8822–8824. https://doi.org/10.1103/PhysRevB.33.8822.
(16) Weigend, F.; Ahlrichs, R. Balanced Basis Sets of Split Valence, Triple Zeta Valence and Quadruple Zeta Valence Quality for H to Rn: Design and Assessment of Accuracy. *Phys. Chem. Chem. Phys.* **2005**, *7* (18), 3297. https://doi.org/10.1039/b508541a.
(17) Weigend, F. Accurate Coulomb-Fitting Basis Sets for H to Rn. *Phys. Chem. Chem. Phys.* **2006**, *8* (9), 1057. https://doi.org/10.1039/b515623h.
(18) Becke, A. D. Density-functional Thermochemistry. III. The Role of Exact Exchange. *J. Chem. Phys.* **1993**, *98* (7), 5648–5652. https://doi.org/10.1063/1.464913.
(19) Miehlich, B.; Savin, A.; Stoll, H.; Preuss, H. Results Obtained with the Correlation Energy Density Functionals of Becke and Lee, Yang and Parr. *Chem. Phys. Lett* **1989**, *157* (3), 200–206. https://doi.org/10.1016/0009-2614(89)87234-3.
(20) Lee, C.; Yang, W.; Parr, R. G. Development of the Colle-Salvetti Correlation-Energy Formula into a Functional of the Electron Density. *Phys. Rev. B* **1988**, *37* (2), 785–789. https://doi.org/10.1103/PhysRevB.37.785.
(21) Yanai, T.; Tew, D. P.; Handy, N. C. A New Hybrid Exchange–Correlation Functional Using the Coulomb-Attenuating Method (CAM-B3LYP). *Chem. Phys. Lett.* **2004**, *393* (1–3), 51–57. https://doi.org/10.1016/j.cplett.2004.06.011.
(22) Dunning, T. H. Gaussian Basis Sets for Use in Correlated Molecular Calculations. I. The Atoms Boron through Neon and Hydrogen. *J. Chem. Phys.* **1989**, *90* (2), 1007–1023. https://doi.org/10.1063/1.456153.
(23) Woon, D. E.; Dunning, T. H. Gaussian Basis Sets for Use in Correlated Molecular Calculations. III. The Atoms Aluminum through Argon. *J. Chem. Phys.* **1993**, *98* (2), 1358–1371. https://doi.org/10.1063/1.464303.
(24) Wilson, A. K.; van Mourik, T.; Dunning, T. H. Gaussian Basis Sets for Use in Correlated Molecular Calculations. VI. Sextuple Zeta Correlation Consistent Basis Sets for Boron through Neon. *Journal of Molecular Structure: THEOCHEM* **1996**, *388*, 339–349. https://doi.org/10.1016/S0166-1280(96)80048-0.
(25) Peterson, K. A.; Woon, D. E.; Dunning, T. H. Benchmark Calculations with Correlated Molecular Wave Functions. IV. The Classical Barrier Height of the $H+H_2 \rightarrow H_2+H$ Reaction. *J. Chem. Phys.* **1994**, *100* (10), 7410–7415. https://doi.org/10.1063/1.466884.

(26) Kendall, R. A.; Dunning, T. H.; Harrison, R. J. Electron Affinities of the First-row Atoms Revisited. Systematic Basis Sets and Wave Functions. *J. Chem. Phys.* **1992**, *96* (9), 6796–6806. https://doi.org/10.1063/1.462569.
(27) Lu, T.; Chen, F. Multiwfn: A Multifunctional Wavefunction Analyzer. *J. Comp. Chem.* **2012**, *33* (5), 580–592. https://doi.org/10.1002/jcc.22885.
